# Modular preparation of cationic bipyridines and azaarenes *via* C–H activation†

**DOI:** 10.1039/d3sc04864k

**Published:** 2023-11-15

**Authors:** Ryan P. King, Jenny Y. Yang

**Affiliations:** a Department of Chemistry, University of California, Irvine Irvine CA 92697 USA j.yang@uci.edu

## Abstract

Bipyridines are ubiquitous in organic and inorganic chemistry because of their redox and photochemical properties and their utility as ligands to transition metals. Cationic substituents on bipyridines and azaarenes are valuable as powerful electron-withdrawing functionalities that also enhance solubility in polar solvents, but there are no general methods for direct functionalization. A versatile method for the preparation of trimethylammonium- and triarylphosphonium-substituted bipyridines and azaheterocycles is disclosed. This methodology showcases a C–H activation of pyridine *N*-oxides that enables a highly modular and scalable synthesis of a diverse array of cationically charged azaarenes. The addition of trimethylammonium functionalities on bipyridine derivatives resulted in more anodic reduction potentials (up to 700 mV) and increased electrochemical reversibility compared to the neutral unfunctionalized bipyridine. Additonally, metallation of 4-triphenylphosphinated biquinoline to make the corresponding Re(CO)_3_Cl complex resulted in reduction potentials 400 mV more anodic than the neutral derivative.

## Abstract 2

A versatile method for the preparation of trimethylammonium- and triarylphosphonium-substituted bipyridines and azaheterocycles is disclosed. The impact of the cationic functionalities was investigated electrochemically.

## Graphical abstract

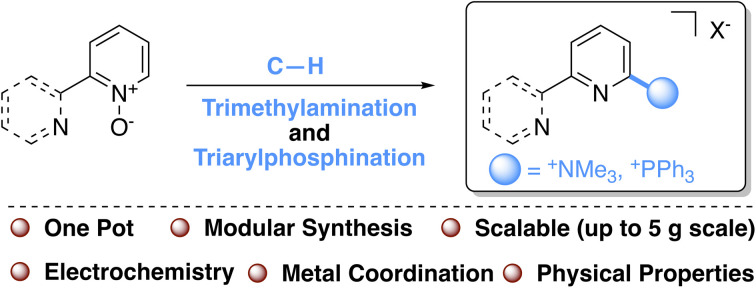

## Introduction

Bipyridines and their derivatives are a broadly useful class of small molecules for applications ranging from electrochemistry^1^ and photochemistry^2^ to medicinal chemistry.^3^ They are also ubiquitous ancillary ligands in transition metal chemistry.^4^ Functionalizing bipyridyls is critical to fine-tuning their specific redox and photophysical properties as isolated species and for their corresponding metal complexes. Among these, charged functional groups are often desirable because of their electron-withdrawing properties^5^ and their favorable impact on solubility in polar solvents.^6^ Charged functionalities impart significant electrostatic effects that can be used to create amphiphilic and phase transfer catalysts,^7^ impact regiochemical control over reactivity,^8^ and inhibit aggregation.^9^

**Fig. 1:**
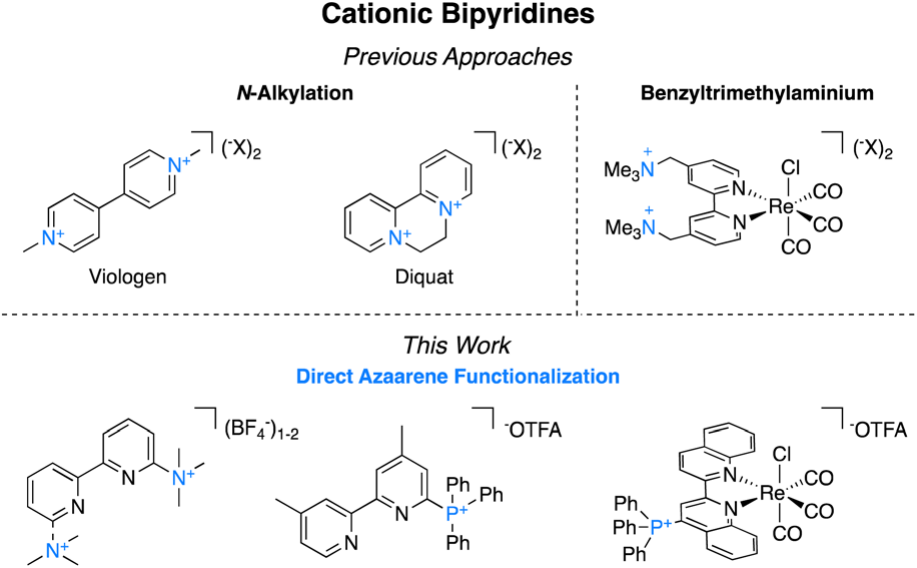
Previous approaches to accessing charged bipyridines and the approach reported herein.

Despite the utility of cationic bipyridines and related ligands, there are no general methods for the preparation of trimethylammonium groups directly bound to the azaarenes. Instead, preparation of isolated cationic bipyridines most commonly occurs through *N*,*N*-dialkylation (Fig. 1). These molecules, commonly called viologens, are widely applicable because of their multiple accessible oxidation states and unique photochemical properties.^10^ In photochemistry and photoredox catalysis, viologens are used as oxidative quenchers of photocatalysts.^11^ Electrochemically, they are common anolytes in both aqueous^12^ and nonaqueous^13^ redox flow batteries and are used as mediators for (bio)electrocatalysis because of their stability in electrochemical cycling.^14^ As these derivatives easily gain electrons, they can also act as herbicides, as they interfere with photosynthesis by disrupting electron transfer reactions and producing harmful radical oxygen species.^15^

Obtaining cationic bipyridines to use as transition metal ligands has been more challenging since they cannot be alkylated at the nitrogen metal-binding site. Instead, the charged functional group is typically tethered to the 2,2′-bipyridyl (bpy) metal complexes through a multi-step synthesis because direct attachment to the azaarene has been generally inaccessible. Meyer and coworkers investigated benzylic trimethylaminium functionalized bpy to study ion pairing in the corresponding iridium and ruthenium complexes.^16^ The same ligand was applied by Thoi and coworkers in aqueous electrocatalytic rhenium-catalyzed carbon dioxide (CO_2_) to carbon monoxide (CO).^17^ Likewise, Tsai and coworkers used this ligand for a palladium-catalyzed Mizoroki–Heck reaction under phase-transfer conditions.^18^ Ertem, Manbeck, and coworkers have shown that even when the trimethylaminium is further separated from the metal center, the impact of the charge has beneficial properties on catalysis.^19^

**Scheme 1:**
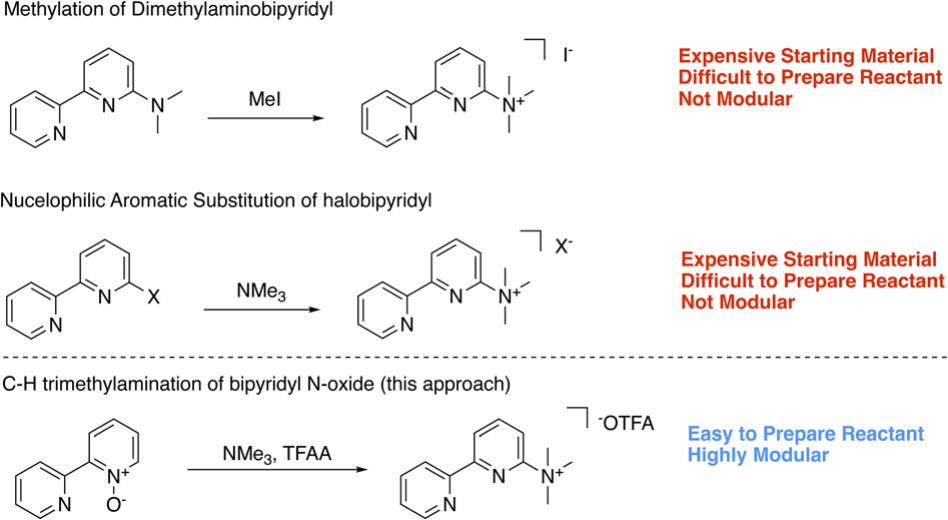
Potential approaches to trimethylaminated bipyridyls.

To expand the series of accessible non-*N* methylated cationic bipyridines and azaarenes, we sought a modular and facile preparation for the direct attachment of trimethylaminium functionalities to azaarenes. Using 6-trimethylaminium-2,2′-bipyridyl 1 as the target molecule, we initially envisioned synthetic access through either methylation of 6-dimethylamino-2,2′-bipyridyl (Scheme 1),^20^ or nucleophilic aromatic substitution of 6-halo-2,2′-bipyridyl with trimethylamine (NMe_3_) (Scheme 1).^21^ While these approaches have been used to access relatively simple trimethylaminated pyridines, their application to bipyridines would require pre-functionalized bipyridyl precursors that are either expensive‡‡For example, on Millipore-Sigma (September 2023), 6-bromo-2,2′-bipyridine is $204 per g ($48,000 per mol) and 6,6′-dibromo-2,2′-bipyridine is $228 per g ($71,600 per mol). or challenging to prepare. Keeping these limitations in mind, we opted to try a pyridine *N*-oxide C–H trimethylamination approach (Scheme 1). This method is inspired by prior work by Xiong and Hoye and coworkers for the fluorination of pyridines.^22^ In their work, a series of functionalized pyridine *N*-oxides were selectively trimethylaminated using trimethylamine (NMe_3_) as a nucleophile and trifluoroacetic anhydride (TFAA) as an activating agent. Since the goal of their study was to use the trimethylaminated pyridines as intermediates in the preparation of fluoropyridines, Xiong and Hoye only isolated two of the trimethylaminated pyridine salts.^22^ This approach was of interest because pyridine *N*-oxides are easy to access with a diverse array of functional groups and substitution patterns. While applying this method to bipyridines is a logical extension of their work, the additional pyridine ring adds complexity. Namely, pyridines are known to be competent nucleophiles under analogous conditions of using TFAA to activate pyridine *N*-oxides.^22,23^ Further, at the outset we were not certain if the products could be easily isolable without the use of reverse phase high performance liquid chromatography (HPLC), which would limit our method's scalability and broad accessibility. In this study, we demonstrate a general and facile method for isolating trimethylaminated and triarylphosphinated bipyridines. Furthermore, we show this approach can be expanded to other azaheterocyclic ligands, including phenanthrolines, terpyridines, and quaterpyridines.

Because the electrochemical properties of bipyridines and their corresponding complexes are a key factor in their widespread utility, the redox properties of the monocationic and dicationic bipyridines were also examined and compared to the neutral bipyridine. Of note is that the cationic bipyridines have both anodically-shifted and more available reduction events, as well as improved overall stability of the reduced species. Additionally, a rhenium complex with a 4-triphenylphosphinated biquinoline was prepared. The presence of the cationic group resulted in an anodic shift to the metal-based reduction potential.

## Results and discussion

### Synthesis of cationic bipyridyls

The reaction of 2,2′-bipyridyine *N*-oxide with 5 equiv. of NMe_3_ and 3 equiv. of TFAA in methylene chloride (CH_2_Cl_2_) at room temperature furnished 1 in 100% yield, as determined by ^1^H NMR spectroscopic analysis (Table 1). Fewer equivalents of amine or anhydride led to lower reaction yields. Isolation of pure 1 as the trifluoroacetate salt proved to be challenging because the concomitantly generated trimethylammonium trifluoroacetate was difficult to separate from 1. Various methods of purification were attempted, including extractions, recrystallizations, bases, and alternative activating groups.§§See ESI† for a list of the activating groups investigated. Fortunately, salt metathesis with aqueous sodium tetrafluoroborate (NaBF_4_) allowed for the isolation of spectroscopically pure 1, albeit in slightly diminished yields (70–80%) due to its partial solubility in water.¶¶Salt metathesis using a 2 M aqueous NaBF_4_ solution was optimal for obtaining our salts in highest yields and highest purities. Use of more dilute or more concentrated solutions, or using other salts, including ammonium tetrafluoroborate, ammonium hexafluorophosphate, and sodium trifluoromethanesulfonate, lead to greatly diminished yields or poor separation from ammonium trifluoroacetate. and ||||It is likely that the trifluoroacetate salt could be isolated by using preparatory reverse-phase HPLC. However, we opted for the salt metathesis approach to allow for our method to be modular, scalable, and accessible to the broadest range of chemists.

**Table 1:** Scope of trimethylaminated azaarenes accessible by C–H trimethylamination of pyridine *N*-oxides^a^

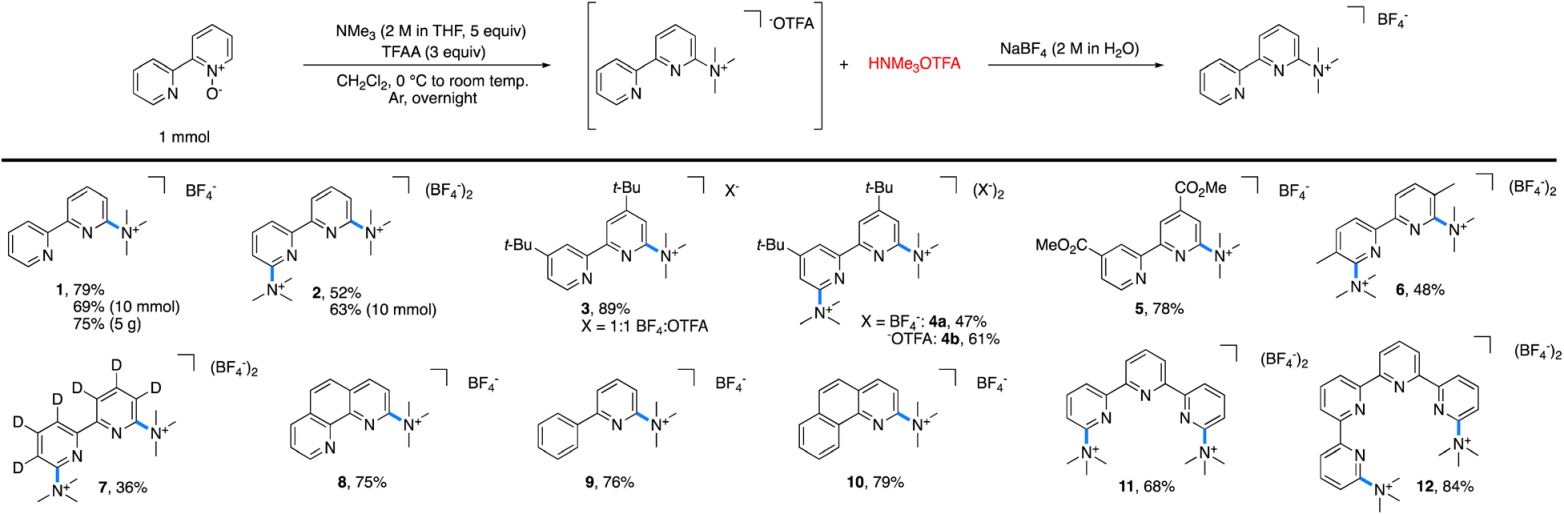

a Standard reaction conditions: pyridine *N*-oxide (1 mmol), trimethylamine (5 mmol), TFAA (3 mmol), and CH_2_Cl_2_ (0.1 M).

With these optimized conditions in hand, the scope of tolerated azaarene *N*-oxides was tested (Table 1). Each reaction was performed on 1 mmol scale or higher. As noted above, the tetrafluoroborate salt of 1 was generated in 79% yield on 1 mmol scale and 69% yield on 10 mmol scale. This approach was easily scaled, as 1 was isolated in 75% yield on 5 g scale with respect to the starting bipyridyl *N*-oxide. This method is not limited to converting mono *N*-oxides into mono trimethylaminated bipyridyls, as 2,2′-bipyridyl *N*,*N*′-bis-*N*-oxide formed the bis trimethylaminated 2 in 52% isolated yield on 1 mmol scale and 63% isolated yield on 10 mmol scale. Substitution in the 4-position of the bipyridine was also tolerated, as the mono and bis trimethylaminated analogues of 4,4′-di-*tert*-butyl-2,2′-bipyridyl, 3 and 4, were obtained in 89% and 47% yield, respectively. In the case of 4, the trifluoroacetate (^−^OTFA) salt can be isolated spectroscopically pure in 61% yield. Further, the electron-deficient 4,4′-bis-esterbipyridyl and more sterically encumbered 5,5′-dimethylbipyridyl were successful under the reaction conditions, furnishing 5 in 78% and 6 in 48% yield. In the case of 7, perdeuteration did not impact the reaction. Additionally, these conditions are not limited to the trimethylamination of bipyridyls; *N*-oxides of phenanthroline (8), 2-phenylpyridine (9), benzo[*h*]quinoline (10), terpyridine (11), and quaterpyridine (12) all afforded the desired salt in moderate to great yields.

One limitation of this method is for substrates based on 4,4′- and 6,6′-dimethylbipyridyl. Here, we postulate that a Boekelheide reaction proceeds instead of our desired salt formation, as trifluoroacetylation of the *N*-oxide causes significant acidification of the protons on the 4- or 6-methyl groups, which can be deprotonated by trimethylamine and lead to undesired reactions.^24^ In an attempt to prepare cationic salts of these substrates, we hypothesized that a heavier pnictogen congener should still have adequate nucleophilicity while having attenuated basicity. Thus, we turned to triarylphosphines, which Bugaenko, Karchava, and coworkers have shown to react with pyridine *N*-oxides using TFAA as an activating agent.^25^ Using slightly modified conditions (1.5 equiv. of tri(*para*-tolyl)phosphine and 1.5 equiv. of TFAA), complete conversion of 4,4′-dimethyl-2,2′-bipyridyl *N*,*N*′-dioxide to the corresponding bis-phosphonium salt 13 (Table 2) was achieved. Due to the lowered basicity of triarylphopshines, no triarylphosphonium trifluoroacetate forms in the reaction, allowing for easy isolation of spectroscopically pure 13 in 95% yield as the trifluoroacetate salt without the need for an aqueous salt metathesis.

**Table 2:** Scope of triarylphosphinated azaarenes accessible by C–H phosphination of pyridine *N*-oxides^a^

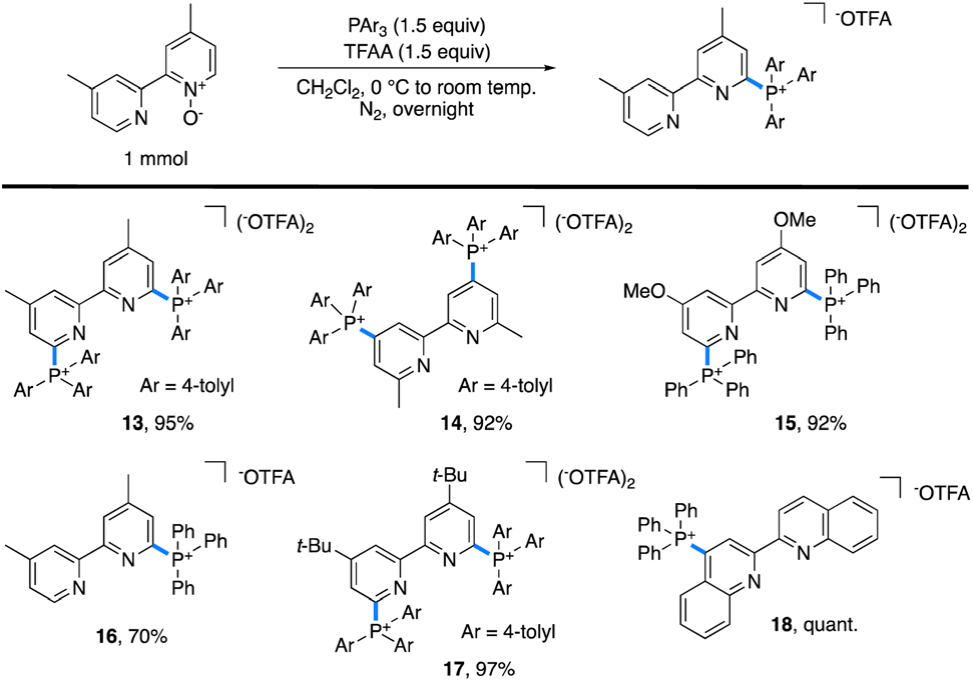

a Standard reaction conditions: pyridine *N*-oxide (1 mmol), triarylphosphine (1.5 mmol), TFAA (1.5 mmol), and CH_2_Cl_2_ (0.1 M).

The scope of accessible triarylphosphonium salts (Table 2) was expanded with 6,6′-dimethyl-2,2′-bipyridyl *N*,*N*′-dioxide and 4,4′-dimethoxy-2,2′-bipyridyl *N*,*N*′-dioxide to afford the phosphonium salts 14 and 15, each in 92% isolated yield. Monophosphonium 16 did not readily precipitate; instead, it was purified by silica gel column chromatography to obtain spectroscopically pure 16 in 70% yield. Further, as 4,4′-di-*tert*-butylbipyridyl is an important ligand in a number of catalytic transformations,^26^ the corresponding bis-phosphonium salt, 17, was synthesized with a 97% isolated yield. Finally, 2,2′-biquinoline (biq) also formed the desired phosphonium 18 in quantitative yield.

### Electrochemistry

The electrochemical behavior of the cation-bearing bipyridines was investigated using cyclic voltammetry (CV) and compared to both uncharged and *N*,*N*′-dialkyl bipyridiniums commonly utilized in electrochemical and other redox processes. All potentials are reported *versus* an internal Fe(C_5_H_5_)_2_^+/0^ standard. The neutral, parent 2,2′-bipyridine in acetonitrile exhibits one reduction event, bpy/bpy˙^−^ at −2.60 V in acetonitrile (MeCN) using tetrabutylammonium hexafluorophosphate (TBAPF_6_) as the supporting electrolyte (Fig. S1†), which matches known values in both tetrahydrofuran and *N*,*N*-dimethylformamide.^27,28^ The one electron reduced species is unstable, as the feature is irreversible at slow scan rates but becomes reversible at fast scan rates (>500 mV s^−1^) (Fig. S2†).

Incorporation of trimethylaminium group(s) results in significant changes to the bipyridine's redox (electrochemical) properties. For monocationic 1, where there is one trimethylaminium group, two different one electron redox features can be observed (Fig. S3†). The first event is the bpy/bpy˙^−^ redox cycle, which is now anodically shifted by almost 400 mV to −2.22 V (Table 3). This singly reduced species is still unstable; however, reversibility is again observed at scan rates of 500 mV s^−1^ and above (Fig. S4†). The second reduction event, which is not present within the solvent window for the parent 2,2′-bipyridine, is quasireversible for 1 with a reduction potential at −2.58 V (Fig. S5†).

**Table 3:** Summary of reduction potentials for 2,2′-bipyridine and selected new cationic derivatives. Potential values are reported *vs.* Fe(C_5_H_5_)_2_^+/0^ and are *E*_1/2_ for reversible and quasireversible events and *E*_pc_ for irreversible events

Compound	1st Reduction (V)	2nd Reduction (V)
2,2′-bipyridine (bpy)	−2.60^a^	—
1	−2.22^a^	−2.58 (quasi.)
2	−1.92	−2.38 (irrev.)^b^
19	−1.14 (irrev.)^b^	−1.60 (irrev.)^b^
20	−0.86	−1.42
21	−1.30	−1.84 (irrev.)^b^

a Reversible at fast scan rates (>500 mV s^−1^).

b Irreversible potentials were recorded as *E*_pc_, or the potential at peak cathodic current, at a scan rate of 100 mV s^−1^.

An even more dramatic effect is observed when multiple trimethylaminium groups are installed. In the case of dicationic 2, where the nitrogen of each pyridyl ring is flanked by a trimethylaminium, the first reduction occurs at −1.92 V and the peak cathodic current for the second irreversible reduction (*E*_pc_) is at −2.38 V (Fig. S6†). Most notably, not only is the first reduction shifted anodically by nearly 700 mV with respect to the unsubstituted 2,2′-bipyridine, the one electron reduced species is more stable, as the redox event is completely reversible even at scan rates as low as 5 mV s^−1^ (Fig. S7†). This redox behavior shows the impact that installing trimethylaminiums and other cationic functional groups has on accessing stable reduced states of bipyridine derivatives.

To complete the series of charged 2,2′-bipyridines, *N*,*N*-dimethylated 2,2′-bipyridinium (19) was also prepared. As expected, both redox features are at even more anodic potentials, where the first reduction proceeds at −1.14 V and the second reduction at −1.60 V (*E*_pc_, Fig. S8†). Surprisingly, while the related *N*,*N*-dialkylated 4,4′-bipyridiniums (viologens) are stable and redox events are reversible, both reduction features of 19 are irreversible.

**Fig. 2:**
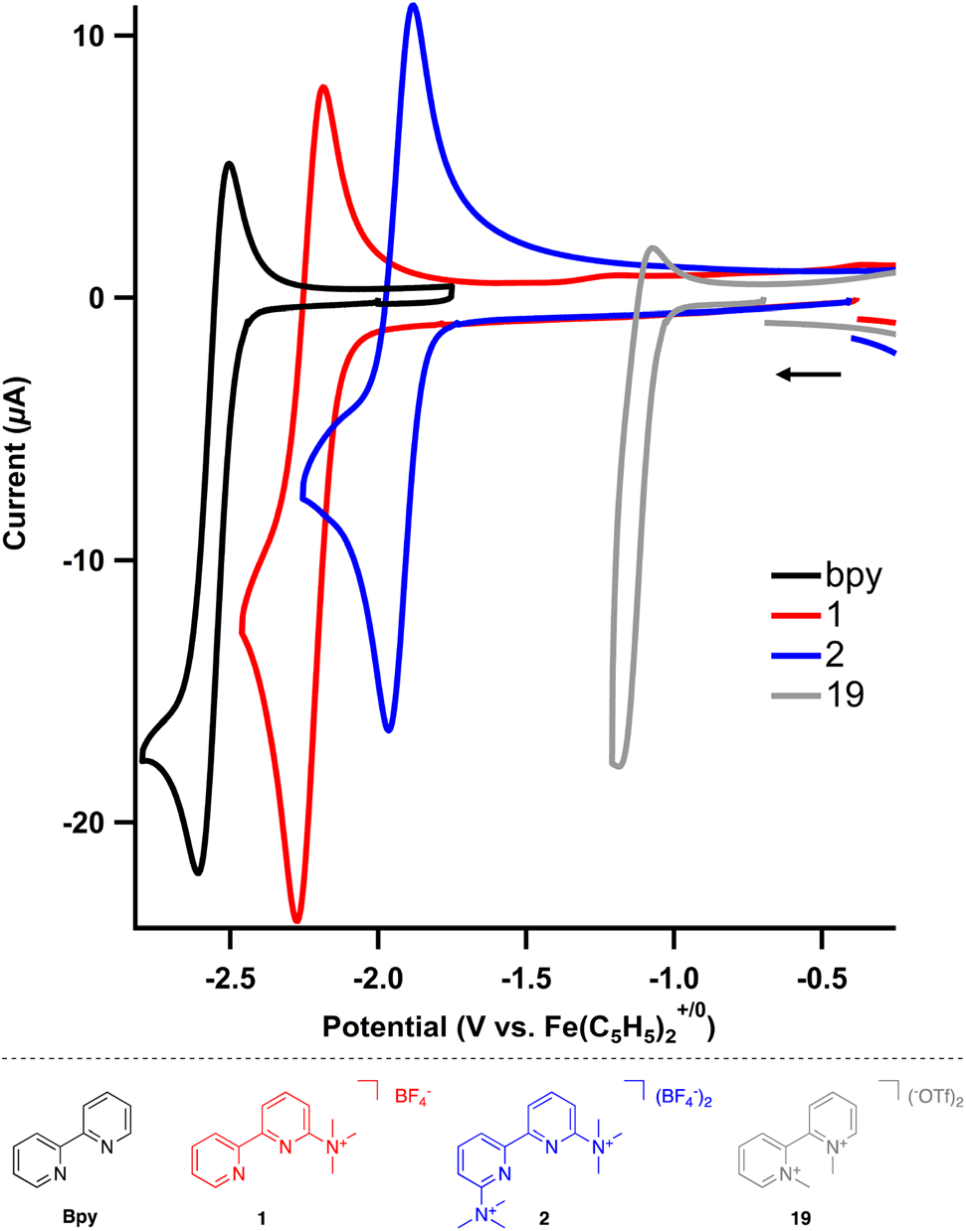
Cyclic voltammograms of substituted bipyridines (2 mM) in MeCN using TBAPF_6_ (0.2 M) as the supporting electrolyte. Scan rate = 500 mV s^−1^.

The installation of trimethylaminium(s) modifies the first reduction of the bipyridines by nearly 700 mV. For comparison, the first reduction event for dimethyl 2,2′-bipyridine-4,4′-carboxylate occurs at −2.03 V, or only a 600 mV change with respect to 2,2′-bipyridine.^28^ The profound change in reduction is clearly tuned by charge, as the reduction events become more positive with increasing charge (Fig. 2). Further, incorporating the trimethylaminium(s) improves the stability of the one electron reduced species by cyclic voltammetry, enhancing their utility as redox-flow battery anolytes or redox mediators. The potential for each reduction is summarized in Table 3.

### Ligand properties

**Scheme 2:**
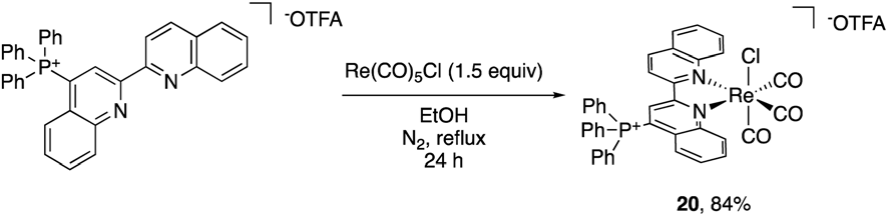
Reaction of 18 with Re(CO)_5_Cl to yield 20.

The impact of the cationic groups on transition metal complexes was also explored. As large functionalities such as *tert*-butyl and dimethylphenyl groups in the 2- or 6-position of bpy or phenanthroline ligands often inhibit metal binding at the pyridine or require C–H activation to bind,^29^ we opted to use 4-triphenylphosphinated biquinoline (18) to obtain desired pyridine binding. Since the cationic functional group is in the 4-position relative to the transition metal, it decouples the electronic impact of the phosphonium from any steric bias that it might impart. Additionally, the ligand influence has the potential to be analyzed in the context of Hammett studies.^30^ The reaction of 18 with pentacarbonylchlororhenium(i) [Re(CO)_5_Cl] readily forms the rhenium tricarbonyl complex 20 in 84% yield (Scheme 2).

The electrochemical and photochemical influence of the phosphonium group on the metal center was investigated by comparing 20 to the uncharged biquinoline bound rhenium complex (biq)Re(CO)_3_Cl (21) *via* CV and UV-visible (UV-Vis) spectroscopy. Electrochemically, complexes of the type (bpy)Re(CO)_3_Cl are known to have two one-electron reductions: a metal-centered reduction at −1.78 V followed by a ligand-centered reduction event at −2.16 V in acetonitrile.^31^ Similarly, the neutral biquinoline-ligated 21 exhibits a reversible reduction at −1.30 V and an irreversible reduction at −1.84 V in *N*,*N*-dimethylformamide (DMF) (Fig. S10†). We attribute these to metal-centered and ligand-centered events based on the similar structure of the biquinoline complex to the bipyridine congener. The more anodic values reflect the more electron-withdrawing nature of the biquinoline ligand compared to bipyridine.

**Fig. 3:**
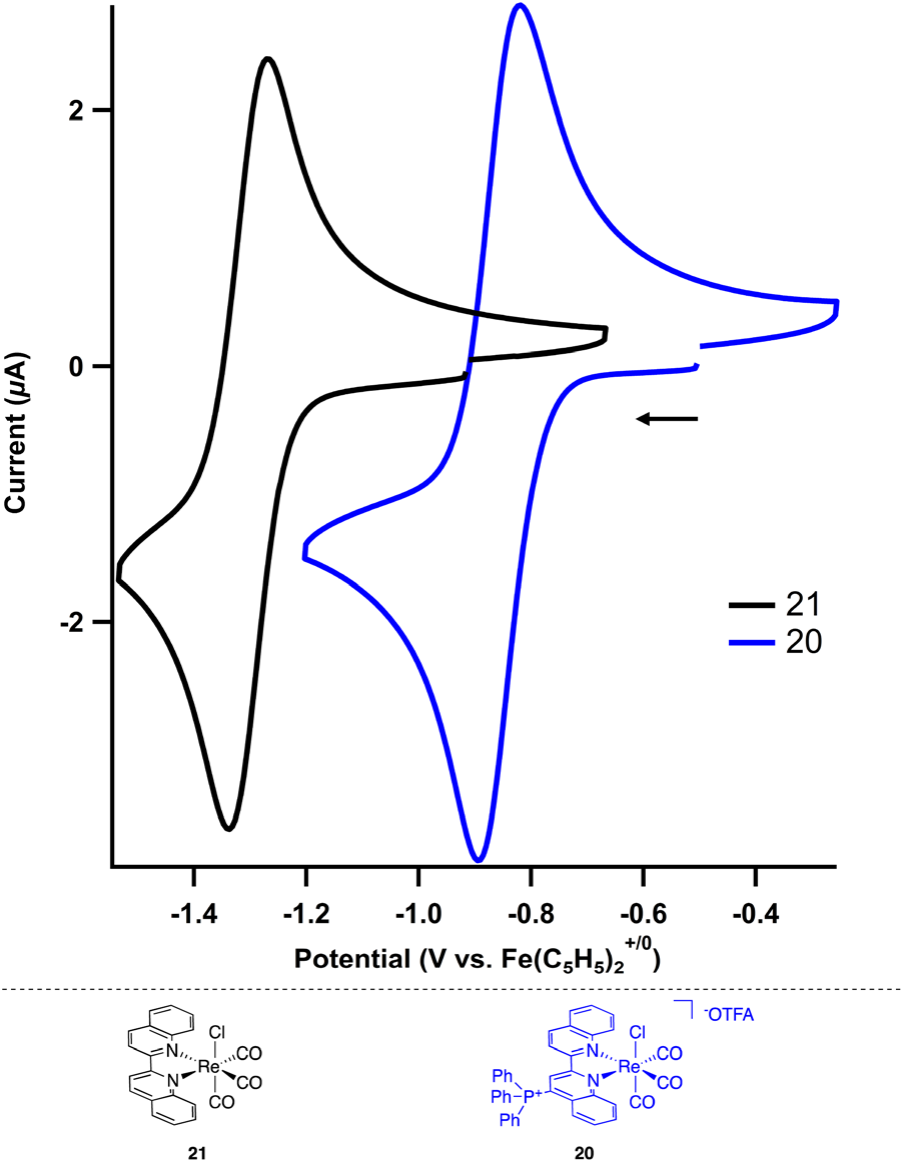
Cyclic voltammograms of the Re^I/0^ for neutral (21, black trace) and cationic (20, blue trace) Re complexes (2 mM) in DMF using TBAPF_6_ (0.2 M) as the supporting electrolyte. Scan rate = 100 mV s^−1^.

In contrast, the cationic triphenylphosphonium complex 20 shows a significant anodic shift for both reductive features compared to the neutral analogue. By installing the charged functional group on the biquinoline, the reversible first reduction occurs at −0.86 V and the second reduction, now reversible, occurs at −1.42 V (Fig. 3, S11 and S12†). This Re(I/0) reduction potential of −0.86 V for 20 is particularly noteworthy as it is 300 mV more anodic than the analogous Re(I/0) redox event (−1.19 V) for the related electron deficient (4,4′-CN-bpy)Re(CO)_3_Cl.^29^ The potential for each reduction is summarized in Table 3.

**Fig. 4:**
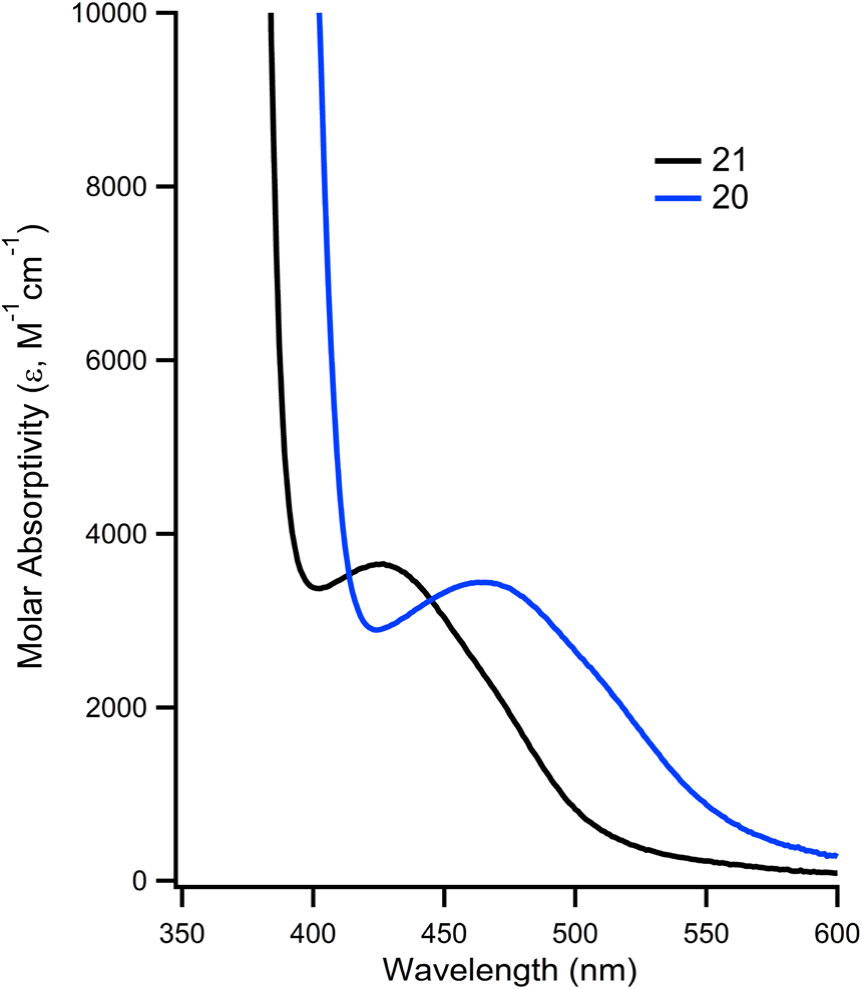
Overlay of UV-vis of 20 (blue trace) and 21 (black trace) showing d → π* transition at room temperature in DMF.

The impact of installing the phosphonium group on the metal's photophysical properties was investigated. In DMF, 21 is known to exhibit an absorption at 426 nm that is characterized as a d → π* transition.^32^ In the case of 20, this absorbance is red shifted by 45 nm, to a *λ*_max_ of 471 nm, while having a slightly higher molar absorptivity (*ε* = 3970 *vs.* 3548 for 21, Fig. 4). The *λ*_max_ and molar absorptivities of the other absorbances remained relatively constant (Fig. S13 and 14†).

Finally, the Re complexes contain carbonyl ligands that are useful infrared spectroscopic probes for the electronic effects of ligands (see ESI†). The parent complex (biquinoline)Re(CO)_3_Cl exhibits infrared vibrational spectra resonances at 2012, 1895, and 1866 cm^−1^ while the cationic derivative 21 has features at 2013, 1899, and 1884 cm^−1^. While we do not assign these resonances to specific carbonyls, we note that the first two are similar, while the third increases by 18 wavenumbers in the cationic species, indicating less π-backbonding.

## Conclusions

The synthesis of a series of previously synthetically inaccessible bipyridyls and azaheterocycles bearing cationic pnictogens is reported. As their preparation involves C–H activation of pyridine *N*-oxides, this method is highly modular and allows for facile trimethylamination and diversification of commercial and existing bipyridyls and azaarenes without requiring *de novo* syntheses for each target molecule. The method is scalable, as products can be prepared on a 5 gram scale. The impact of the cationic functionalities on the derivatives, as well as the physical properties of the corresponding complexes, were investigated. The strong electron-withdrawing nature of the cationic pnictogens resulted in significant anodic shifts in the reduction events while also improving the stability of the reduced species. This general synthetic method and the charged azaarenes will have a positive impact on a wide range of applications, from new amphiphilic metal-based photo- and electrocatalysts to organic materials with beneficial electrochemical and optical properties.

## Data availability

All additional data can be found in the ESI.†

## Author contributions

J. Y. Y. supervised the project and conceived the targets 1 and 2. R. P. K. conceived the method, performed all of the syntheses and NMR spectroscopic analyses, and prepared the manuscript with feedback from J. Y. Y.

## Conflicts of interest

There are no conflicts to declare.

## Supplementary Material

SC-014-D3SC04864K-s001
