# Supplementary material for: Modular preparation of cationic bipyridines and azaarenes via C–H activation
Source: Chem Sci. 2023 Nov 15;14(46):13530–6. doi: 10.1039/d3sc04864k (PMC10686024; doi:10.1039/d3sc04864k)

Electronic Supplementary Material (ESI) for Chemical Science.
This journal is © The Royal Society of Chemistry 2023

Modular Preparation of Cationic Bipyridines and Azaarenes via C–H Activation

Ryan P. King[1] and Jenny Y. Yang[1*]

[1]Department of Chemistry, University of California, Irvine, Irvine, 92697, USA

*Correspondence to: j.yang@uci.edu

Supporting Information

Table of Contents

I. General Information..S3

II. Investigation of Other Activating Groups...S5

III. Synthesis of Starting Materials..S6

IV. Synthesis of Trimethylaminated and Triarylphosphinated Azaheterocycles.................S7

V. Electrochemistry and UV–Vis Spectroscopy...S21

VI. References...S35

VII. Copies of NMR Spectra..S37

I. General Information

General Analytical Information
All compounds were characterized by ^{1}H NMR, ^{13}C NMR, ^{2}H NMR (when applicable), ^{19}F NMR (when applicable), ^{31}P NMR (when applicable), and Infrared (IR) spectroscopy. All new compounds were also characterized by high-resolution mass spectrometry (HRMS). Nuclear Magnetic Resonance spectra were recorded on a Bruker 400, 500, or 600 MHz instrument. All NMR spectra are recorded in δ units and parts per million (ppm). The multiplicities are abbreviated with s (singlet), br s (broad singlet), d (doublet), t (triplet), m (multiplet), and app (apparent). All ^{1}H and ^{13}C spectra were calibrated using residual solvent as an internal reference ($CDCl_3$: δ 7.26 ppm and δ 77.16 ppm respectively; CD_2Cl_2: δ 5.32 ppm and δ 53.84 ppm respectively; CD_3OD: δ 3.31 ppm and δ 49.00 ppm respectively; DMSO: δ 2.50 ppm and δ 39.52 ppm respectively; CD_3CN: δ 1.94 ppm and δ 118.26 ppm respectively).[1] All ^{19}F NMR spectra were calibrated to an external standard of trifluorotoluene ($PhCF_3$) in $CDCl_3$ (δ –63.72 ppm). All ^{31}P NMR spectra were calibrated to an external standard of triphenylphosphine in $CDCl_3$ (δ –4.90 ppm). All ^{13}C NMR and ^{31}P NMR spectra were obtained with ^{1}H NMR decoupling. All HRMS were recorded on a Waters LCT Premier ESI-LC-TOF system. All IR spectra were obtained on a Thermo Scientific Nicolet iS5 spectrometer (iD5 ATR, diamond) and were reported in wavenumbers (cm^{-1}). Flash column chromatography was performed using Fisher Chemical silica gel (40-63 µm, 230-400 mesh, Grade 60, Cat. #: S825).

Electrochemistry
All measurements were performed on a Pine Wavedriver 10 bipotentiostat using a 11 mm glassy carbon disc working electrode, a glassy carbon rod counter electrode, and a $Ag^{+/0}$ pseudoreference electrode. Potentials are referenced to a ferrocene internal standard ($Fe(C_5H_5)_2^{+/0}$) set to 0.00 V. All experiments were performed in an Ar-filled glovebox using oven dried glassware and dried and degassed acetonitrile or *N*,*N*-dimethylformamide. Analyses were performed on solutions containing 2 mM analyte and 0.2 M tetrabutylammonium hexafluorophosphate as the electrolyte.

Ultraviolet–Visible (UV–Vis) Electronic Absorption Spectroscopy
All measurements were performed in air in an Agilent Technologies Cary 60 UV–Vis spectrometer using a 1 mm pathlength quartz cuvette.

General Reagent Information
Reagent information: All reagents were purchased from commercial sources and used as received unless otherwise noted. Tetrabutylammonium hexafluorophosphate ($TBAPF_6$, electrochemical grade) was purchased from Tokyo Chemical Industry Co., recrystallized from ethanol 3x, dried in a vacuum oven at 80 °C overnight, and placed in a N_2-filled glovebox prior to use. 3-chloroperoxybenzoic acid (mCPBA, 70 – 75%, treated as 70%) was purchased from Acros and stored in a 0 °C freezer. 2,2'-Biquinoline was purchased from Combi-Blocks. 2,2'-bipyridine was purchased from Strem. Hydrogen peroxide (H_2O_2, 30% in water) was purchased from Fisher Scientific and stored in a 0 °C freezer. Trifluoroacetic acid (TFA) was purchased from Acros. Trifluoroacetic anhydride (TFAA), 2,2'-bipyridyl *N*-oxide, triphenylphosphine, methyl trifluoromethanesulfonate (MeOTf), and pentacarbonylchlororhenium (I) [$Re(CO)_5Cl$] were purchased from Millipore-Sigma. Sodium tetrafluoroborate ($NaBF_4$) was purchased from Strem. Trimethylamine (2M in THF) and 2,2'-bipyridine *N*,*N*'-dioxide were purchased from Tokyo

Chemical Industry Co. 3 Å molecular sieves (1–2 mm beads) were purchased from Fisher Scientific and activated by drying in a vacuum oven at 200 °C overnight prior to use. Deuterated solvents were purchased from Cambridge Isotope Laboratories and dried over activated molecular sieves (3 Å) overnight prior to use.

Solvent Information: Methylene chloride (CH_2Cl_2), *N*,*N*-dimethylformamide (DMF), and ethanol (EtOH) for reactions and UV–Vis analyses were purchased from Fisher Scientific, degassed by sparging with argon, and dried by passing through two columns of neutral alumina in a solvent purification system prior to use. Acetonitrile (MeCN) and *N*,*N*-dimethylformamide (DMF) for CVs were purchased from Fisher Scientific, degassed by sparging with argon, dried by passing through two columns of neutral alumina in a solvent purification system prior, brought into a N_2-filled glovebox and stored over activated molecular sieves (3 Å) overnight prior to use. Diethyl ether (stabilized, Cat. #: E138), methylene chloride (stabilized, Cat. #: D37), ethyl acetate (EtOAc, Cat. #: E145), acetone (Cat. #: A18) and methanol (Cat. #: A412) for workups and purifications were purchased from Fisher Scientific and used as received.

2,2'-bipyridine *N*-oxide,[2] 4,4'-di-*tert*-butyl-2,2'-bipyridine *N*-oxide,[3] 4,4'-bis(methoxycarbonyl)-2,2'-bipyridine *N*-oxide,[4] D_8-2,2'-bipyridine *N*,*N*'-dioxide,[5] 1,10-phenanthroline *N*-oxide,[6] 2-phenylpyridine *N*-oxide,[7] benzo[h]quinoline *N*-oxide,[8] 2,2':6',2"-terpyridine *N*,*N*"-dioxide,[9] 2,2':6',2":6",2"'-quaterpyridine,[10] 4,4'-dimethyl-2,2'-bipyridine *N*,*N*'-dioxide,[11] 4,4'-dimethyl-2,2'-bipyridine *N*-oxide,[12] 6,6'-dimethyl-2,2'-bipyridine *N*,*N*'-dioxide,[13] 4,4'-dimethoxy-2,2'-bipyridine *N*,*N*'-dioxide,[14] and 2,2'-biquinoline *N*-oxide[15] were prepared according to literature procedures.

II. Investigation of Other Activating Groups

Table 1. Activating Groups Investigated

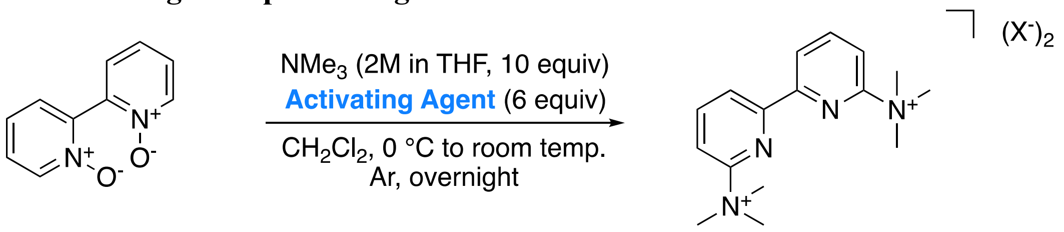

Activating Agent	% Conversion
Trifluoroacetic Anhydride	100
Pentafluoropropionic Anhydride	100
Heptafluorobutyric Anhydride	100
Oxalyl Chloride	100
Trifluoromethanesulfonic Anhydride	Intractable Mixture
Methanesulfonic Anhydride	0
Pivalic Anhydride	0
Benzoic Anhydride	0
Pentafluorophenyl Trifluoroacetate	0
Trimethylsilyl Acetate	0
Trimethylsilyl Trifluoroacetate	0
Trimethylsilyl Trifluoromethanesulfonate	0
Trimethylsilyl Methanesulfonate	0

Procedure: A flame-dried 20 mL scintillation vial equipped with a magnetic stir bar and septum cap was allowed to cool to room temperature under vacuum before being backfilled with nitrogen. At this point, the cap was removed and the vial was charged with pyridine *N*-oxide (1 mmol, 1 equiv). The cap was returned and the vial was placed under a balloon of argon before being charged with CH_2Cl_2 (5 mL) and NMe_3 (2 M in THF, 5 mL, 10 mmol, 5 equiv w.r.t. each *N*-oxide). The vial was placed in an ice/water bath and the contents were allowed to cool to 0 °C. The reaction was initiated by adding the corresponding activating agent (6 mmol, 3 equiv w.r.t. each *N*-oxide). The ice-water bath was removed and the reaction mixture was allowed to stir overnight at room temperature. At this point, the vial was opened to air and the reaction mixture was quenched with methanol (1 mL) and charged with 1,3,5-trimethoxybenzene (8.4 mg). ~ 500 µL of the mixture was transferred to an NMR tube, diluted with CD_3OD, and the conversion was determined by ^1H NMR spectroscopic analysis.

III. Synthesis of Starting Materials

4,4'-di-*tert*-butyl-2,2'-bipyridine *N,N'*-dioxide (S1)

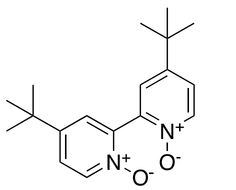

A 50 mL round bottom flask equipped with a magnetic stir bar was charged with 4,4'-di-*tert*-butyl-2,2'-bipyridine (971 mg, 3.6 mmol, 1 equiv) and CH_2Cl_2 (17 mL). The reaction was initiated by adding mCPBA (70%, 2.26 g, 9.2 mmol, 2.5 equiv) slowly to yield a yellow solution. The resulting solution was allowed to stir in air at room temperature overnight. At this time, the reaction was diluted with CH_2Cl_2 (50 mL) and quenched with a sodium carbonate solution (1 M in water, 50 mL). The organic layer was separated and the aqueous layer was further extracted with CH_2Cl_2 (3 x 50 mL). The organic layers were combined, dried over magnesium sulfate, filtered and concentrated *in vacuo*. The crude material was purified by column chromatography (150 mL SiO_2, 9:1 to 8:2 CH_2Cl_2:MeOH, UV) and dried *in vacuo* overnight to yield **S1** (973 mg, 90%) as a tan powder. The ^1H and ^{13}C NMR spectroscopic data matched that reported in the literature.[16]

^{1}H NMR (600 MHz, $CDCl_3$) δ 8.15 (d, J = 7.0 Hz, 2H), 7.49 (d, J = 2.9 Hz, 2H), 7.24 (dd, J = 6.9, 2.8 Hz, 2H), 1.23 (s, 18H).
^{13}C NMR (151 MHz, $CDCl_3$) δ 149.8, 141.3, 139.1, 125.1, 123.7, 34.5, 30.3.

5,5'-dimethyl-2,2'-bipyridine *N,N'*-dioxide (S2)

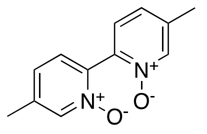

A 100 mL round bottom flask equipped with a magnetic stir bar was charged with 5,5'-dimethyl-2,2'-bipyridine (1.00 g, 5.4 mmol, 1 equiv) and CH_2Cl_2 (25 mL). The reaction was initiated by adding mCPBA (70%, 3.39 g, 13.8 mmol, 2.6 equiv) slowly to yield a yellow solution. The resulting solution was allowed to stir in air at room temperature overnight. At this time, the reaction was diluted with CH_2Cl_2 (50 mL) and quenched with a sodium carbonate solution (1 M in water, 50 mL). The organic layer was separated and the aqueous layer was further extracted with CH_2Cl_2 (3 x 50 mL). The organic layers were combined, dried over magnesium sulfate, filtered and concentrated *in vacuo* to yield an off-white solid. The solid was triturated with the assistance of sonication with cold EtOAc. The solid was collected on a fritted funnel and washed with cold EtOAc to yield **S2** (425 mg, 36%) as an off-white powder. The ^1H and ^{13}C NMR spectroscopic data matched that reported in the literature.[17]

^{1}H NMR (600 MHz, $CDCl_3$) δ 8.14 (s, 2H), 7.52 (d, J = 8.1 Hz, 2H), 7.11 (d, J = 8.1 Hz, 2H), 2.30 (s, 6H).
^{13}C NMR (151 MHz, $CDCl_3$) δ 139.9, 139.4, 137.3, 127.8, 126.3, 18.4.

2,2':6',2":6",2"'-quaterpyridine *N,N"'*-dioxide (S3)

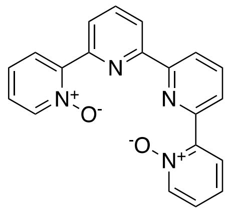

A 100 mL round bottom flask equipped with a magnetic stir bar was charged with 2,2':6',2":6",2"'-quaterpyridine (776 mg, 2.5 mmol, 1 equiv) and CH_2Cl_2 (25 mL). The reaction was initiated by adding mCPBA (70%, 1.48 g, 6 mmol, 2.4 equiv) slowly to yield a yellow solution. The resulting solution was allowed to stir in air at room temperature overnight, over which time the solution became a white slurry. At this time, acetone (50 mL) was added and the mixture was allowed to stir until a free-flowing

coarse powder forms. The resulting powder was collected on a fritted funnel and further washed with acetone (50 mL) to yield **S3** (753 mg, 88%) as a white powder.

^{1}H NMR (600 MHz, $CDCl_3$) δ 9.04 (dd, *J* = 7.9, 1.1 Hz, 2H), 8.58 (dd, *J* = 7.9, 1.1 Hz, 2H), 8.42 (dd, *J* = 8.1, 2.1 Hz, 2H), 8.36 (dd, *J* = 6.5, 1.3 Hz, 2H), 7.99 (t, *J* = 7.9 Hz, 2H), 7.43 (td, *J* = 7.7, 1.2 Hz, 2H), 7.34 – 7.29 (m, 2H).
^{13}C NMR (151 MHz, $CDCl_3$) δ 155.5, 149.0, 147.6, 141.0, 137.5, 128.2, 125.8, 125.4, 121.7.
IR (neat): 3117, 3069, 1613, 1566, 1492, 1438 1426, 1378, 1309, 1262, 1239, 1153, 1122, 1106, 1076, 1038, 990, 948, 872, 847, 803, 765, 746, 734, 642, 630, 598, 561, 549, 512, 479 cm^{-1}
HRMS (ESI) Calcd. For $C_{20}H_{14}NaN_4O_2^+$ $(M+Na)^+$: 365.1014. Found: 365.1000.

IV. Synthesis of Trimethylaminated and Triarylphosphinated Azaheterocycles

General Procedure A: Trimethylamination of *N*-Oxides
A flame-dried 20 mL scintillation vial equipped with a magnetic stir bar and septum cap was allowed to cool to room temperature under vacuum before being backfilled with nitrogen. At this point, the cap was removed and the vial was charged with pyridine *N*-oxide (1 mmol, 1 equiv). The cap was returned and the vial was placed under a balloon of argon before being charged with CH_2Cl_2 (5 – 7.5 mL) and NMe_3 (2 M in THF, 2.5 – 5 mL, 5 – 10 mmol, 5 equiv w.r.t. each *N*-oxide). The vial was placed in an ice/water bath and the contents were allowed to cool to 0 °C. The reaction was initiated by adding TFAA (420 – 840 µL, 3 – 6 mmol, 3 equiv w.r.t. each *N*-oxide). The ice-water bath was removed and the reaction mixture was allowed to stir overnight at room temperature,[I] over which time a suspension or slurry formed. At this point, the vial was opened to air and the reaction mixture was diluted with CH_2Cl_2 (1 mL) and poured into a 125 mL Erlenmeyer flask containing diethyl ether (50 mL) to form a white precipitate. The resulting precipitate was collected on a fritted funnel and washed with diethyl ether (~ 25 mL). The filter cake, a mixture of trimethylaminated product and trimethylammonium trifluoroacetate (TMAOTFA), was dissolved in minimal H_2O and charged with a sodium tetrafluoroborate solution (2 M in H_2O)[II] to form a new precipitate. The flask was scratched with a glass stir rod to assist with precipitation. The precipitate was collected on a fritted funnel and washed with H_2O and ethyl acetate and dried *in vacuo* overnight to afford the desired product.[III]

General Notes:
[I]While each reaction was allowed to stir overnight, most substrates reached full conversion within 2 h.
[II]The amount of sodium tetrafluoroborate solution used is dependent on the amount of TMAOTFA present, which was determined by ^{1}H NMR spectroscopy. Approximately 2 equiv of $NaBF_4$ solution is used relative to the sum total of product and TMAOTFA. See specific substrates for amounts of $NaBF_4$ solution used.
[III]For some substrates, a small amount of trimethylammonium tetrafluoroborate ($TMABF_4$) was still present (< 10%) after the workup. In these instances (highlighted below), the sodium tetrafluoroborate wash was repeated once more.

General Procedure B: Triarylphosphination of *N*-Oxides
A flame-dried 20 mL scintillation vial equipped with a magnetic stir bar and septum cap was allowed to cool to room temperature under vacuum before being backfilled with nitrogen. At this point, the cap was removed and the vial was charged with pyridine *N*-oxide (0.5 mmol, 1 equiv) and tri(*p*-tolyl)phosphine (228 – 457 mg, 0.75 – 1.5 mmol, 1.5 equiv w.r.t. each *N*-oxide). The cap was returned and the vial was placed under nitrogen before being charged with CH_2Cl_2 (2.5 mL). The vial was placed in an ice/water bath and the contents were allowed to cool to 0 °C. The reaction was initiated by adding TFAA (110 – 220 µL, 0.75 – 1.5 mmol, 1.5 equiv w.r.t. each *N*-oxide). The ice-water bath was removed and the reaction mixture was allowed to stir overnight at room temperature. At this point, the vial was opened to air and the contents were transferred to a 50 mL round bottom flask. The solution was concentrated *in vacuo* with the assistance of a rotary evaporator and a high vacuum to form an oil. The oil was layered with diethyl ether (25 mL), the flask was scratched with a glass stir rod, and the contents were triturated with the assistance of sonication to form a precipitate. The mixture was allowed to sit for 10 min, then the precipitate was collected on a fritted funnel and washed with diethyl ether (25 mL) and dried *in vacuo* overnight to afford the desired product.

***N,N,N*-trimethyl-2,2'-bipyridin-6-aminium tetrafluoroborate (1)**

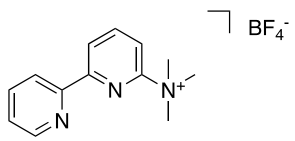

Compound **1** was prepared according to a modified General Procedure **A** using 2,2'-bipyridine *N*-oxide (172 mg, 1 mmol, 1 equiv), trimethylamine (2 M in THF, 2.5 mL, 5 mmol, 5 equiv) and TFAA (420 µL, 3 mmol, 3 equiv) and CH_2Cl_2 (7.5 mL). After stirring at room temperature overnight, the flask was opened to air and the reaction mixture was charged with CH_2Cl_2 (1 mL). The cloudy solution was slowly filtered through a fine fritted funnel, and the white filter cake (TMAOTFA) was washed with CH_2Cl_2. The filtrate was concentrated *in vacuo* to yield a yellow oil. The oil was diluted with water (2 mL) and subsequently charged with a $NaBF_4$ solution (2 M in water, 2 mL). The flask was scratched, sealed with a plastic stopper, and placed in a 0 °C freezer overnight. Colorless crystals formed overnight, which were collected on a fritted funnel and washed with water (5 mL) and diethyl ether (5 mL) to yield **1** (239 mg, 79%) as colorless crystals.

5 mmol scale: Following a modified General Procedure **A** using a flame-dried 100 mL round bottom flask, 2,2'-bipyridine *N*-oxide (860 mg, 5 mmol, 1 equiv) was allowed to react with trimethylamine (2 M in THF, 12.5 mL, 25 mmol, 5 equiv) and TFAA (2.1 mL, 15 mmol, 3 equiv) in CH_2Cl_2 (37.5 mL). After stirring at room temperature overnight, the flask was opened to air and the reaction mixture was charged with CH_2Cl_2 (5 mL). The cloudy solution was slowly filtered through a fine fritted funnel, and the white filter cake (TMAOTFA) was washed with CH_2Cl_2. The filtrate was collected and poured into a 500 mL Erlenmeyer flask containing diethyl ether (300 mL), resulting in the formation of a white precipitate. The resulting precipitate was collected on a fritted funnel and washed with diethyl ether. The filter cake was dissolved in water (3 mL) and a precipitate formed following the addition of a $NaBF_4$ solution (2 M in water, 5 mL). The precipitate was collected on a fritted funnel and washed with water (5 mL) and EtOAc (5 mL) to yield **1** (822 mg, 55%) as a shimmery white powder.

10 mmol scale: Following a modified General Procedure **A** using a flame-dried 200 mL round bottom flask, 2,2'-bipyridine *N*-oxide (1.72 g, 10 mmol, 1 equiv) was allowed to react with

trimethylamine (2 M in THF, 25 mL, 50 mmol, 5 equiv) and TFAA (4.2 mL, 30 mmol, 3 equiv) in CH_2Cl_2 (75 mL). After stirring at room temperature overnight, the flask was opened to air and the reaction mixture was charged with CH_2Cl_2 (10 mL). The cloudy solution was slowly filtered through a fine fritted funnel, and the white filter cake (TMAOTFA) was washed with CH_2Cl_2. The filtrate was collected and poured into a 1 L Erlenmeyer flask containing diethyl ether (600 mL), resulting in the formation of a white precipitate. The resulting precipitate was collected on a fritted funnel and washed with diethyl ether. The filter cake was dissolved in water (6 mL) and a precipitate formed following the addition of a $NaBF_4$ solution (2 M in water, 10 mL). The precipitate was collected on a fritted funnel and washed with water (10 mL) and EtOAc (10 mL) to yield **1** (2.07 g, 69%) as a shimmery white powder.

5 g scale: Following a modified General Procedure **A** using a flame-dried 500 mL round bottom flask, 2,2'-bipyridine *N*-oxide (5.00 g, 29 mmol, 1 equiv) was allowed to react with trimethylamine (2 M in THF, 75 mL, 150 mmol, 5.2 equiv) and TFAA (12.6 mL, 90 mmol, 3.1 equiv) in CH_2Cl_2 (225 mL). After stirring at room temperature overnight, the flask was opened to air and the reaction mixture was charged with CH_2Cl_2 (30 mL). The cloudy solution is slowly filtered through a fine fritted funnel, and the white filter cake (TMAOTFA) was washed with CH_2Cl_2. The filtrate was concentrated *in vacuo* to yield a yellow oil. The oil was diluted with water (60 mL) and subsequently charged with a $NaBF_4$ solution (2 M in water, 60 mL). The flask was scratched, triturated with the assistance of sonication, sealed with a plastic stopper, and placed in a 0 °C freezer for 2 h. Colorless crystals formed over this period, which were collected on a fritted funnel and washed with water (50 mL) and EtOAc (30 mL) to yield **1** (6.53 g, 75%) as a shimmery white powder.

^{1}H NMR (600 MHz, CD_3CN) δ 8.72 (d, J = 5.8 Hz, 1H), 8.64 (d, J = 7.8 Hz, 1H), 8.46 (dd, J = 7.8, 1.2 Hz, 1H), 8.24 (td, J = 8.0, 1.1 Hz, 1H), 7.97 (t, J = 7.8 Hz, 1H), 7.85 (d, J = 8.1 Hz, 1H), 7.51 – 7.46 (m, 1H), 3.62 (s, 9H).
^{13}C NMR (151 MHz, CD_3CN) δ 156.9, 156.7, 154.4, 150.6, 143.0, 138.4, 126.2, 123.7, 122.3, 115.1, 56.0.
^{19}F NMR (565 MHz, CD_3CN) δ –151.66, –151.71.
IR (neat): 3091, 3053, 1604, 1588, 1561, 1497, 1473, 1470, 1458, 1438, 1422, 1408, 1309, 1288, 1268, 1255, 1185, 1057, 1020, 992, 945, 866, 796, 778, 748, 737, 701, 661, 635, 619, 569, 521 cm^{-1}
HRMS (ESI) Calcd. For $C_{13}H_{16}N_3^+$ $(M–BF_4)^+$: 214.1339. Found: 214.1342.

***N,N,N,N',N',N'*-hexamethyl-2,2'-bipyridin-6,6'-diaminium bis(tetrafluoroborate) (2)**

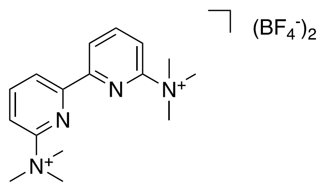

Compound **2** was prepared according to General Procedure **A** using 2,2'-bipyridine *N*,*N*'-dioxide (188 mg, 1 mmol, 1 equiv), trimethylamine (2 M in THF, 5 mL, 10 mmol, 10 equiv), TFAA (840 µL, 6 mmol, 6 equiv), and CH_2Cl_2 (5 mL). The filter cake was dissolved in water (4 mL) and a precipitate formed following addition of a $NaBF_4$ solution (2 M in water, 40 mL). The precipitate was collected on a fritted funnel and washed with water (5 mL) and EtOAc (5 mL) to yield **2** (232 mg, 52%) as a white powder.

10 mmol: Following General Procedure **A** using a flame-dried 200 mL round bottom flask, 2,2'-bipyridine *N,N'*-dioxide (1.88 g, 10 mmol, 1 equiv), trimethylamine (2 M in THF, 50 mL, 100 mmol, 10 equiv), TFAA (8.4 mL, 60 mmol, 6 equiv), and CH_2Cl_2 (50 mL). The filter cake was dissolved in water (40 mL) and a precipitate formed following addition of a $NaBF_4$ solution (2 M in water, 400 mL). The precipitate was collected on a fritted funnel and washed with water (40 mL) and EtOAc (10 mL) to yield **2** (2.80 g, 63%) as a white powder.

^{1}H NMR (600 MHz, CD_3CN) δ 8.70 (d, J = 7.8 Hz, 2H), 8.36 – 8.30 (m, 2H), 7.96 (d, J = 8.3 Hz, 2H), 3.64 (s, 18H).
^{13}C NMR (151 MHz, CD_3CN) δ 157.1, 154.1, 143.5, 124.6, 116.7, 56.0.
^{19}F NMR (565 MHz, CD_3CN) δ –151.63, –151.68.
IR (neat): 3118, 3060, 1600, 1563, 1493, 1468, 1428, 1289, 1178, 1158, 1029, 994, 946, 845, 810, 746, 632, 605, 521, 497 cm^{-1}
HRMS (ESI) Calcd. For $C_{16}H_{24}BF_4N_4^+$ (M–BF_4)$^+$: 359.2025. Found: 359.2032.

***N,N,N*-trimethyl-4,4'-di-*tert*-butyl-2,2'-bipyridin-6-aminium tetrafluoroborate (3)**

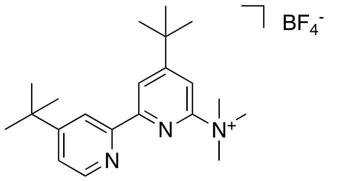

Compound **3** was prepared according to a modified General Procedure **A** using 4,4'-di-*tert*-butyl-2,2'-bipyridine *N*-oxide (284 mg, 1.0 mmol, 1 equiv), trimethylamine (2 M in THF, 2.5 mL, 5 mmol, 5 equiv), TFAA (420 µL, 3 mmol, 3 equiv), and CH_2Cl_2 (7.5 mL). After stirring at room temperature overnight, the flask was opened to air and the reaction mixture was charged with CH_2Cl_2 (1 mL). The cloudy solution is slowly filtered through a fine fritted funnel, and the white filter cake (TMAOTFA) was washed with CH_2Cl_2. The filtrate was concentrated *in vacuo* and was subsequently dissolved in water (2 mL). A yellow precipitate forms upon addition of a $NaBF_4$ solution (2 M in water, 10 mL). The precipitate was collected on a fritted funnel and washed with water (10 mL) and diethyl ether to yield **3** (252 mg) as a white powder. Off-white crystals formed in the filtrate overnight, which were collected on a fritted funnel to yield a second crop of **3** (129 mg). Combined total yield 381 mg, 89%.
Note: **3** was determined to be a 1:1 mixture of the $^-BF_4$ to $^-$OTFA salt. The yield is based off of this salt ratio.

^{1}H NMR (600 MHz, CD_3CN) δ 8.87 (d, J = 5.8 Hz, 1H), 8.55 (d, J = 1.3 Hz, 1H), 8.54 (d, J = 1.9 Hz, 1H), 7.90 (dd, J = 5.9, 1.9 Hz, 1H), 7.87 (d, J = 1.2 Hz, 1H), 3.67 (s, 916H), 1.47 (s, 9H), 1.47 (s, 9H).
^{13}C NMR (151 MHz, CD_3CN) δ 169.9, 169.4, 157.8, 151.0, 150.0, 146.0, 124.9, 122.4, 122.1, 114.4, 56.1, 37.3, 37.0, 30.5, 30.3.
^{19}F NMR (376 MHz, CD_3CN) δ –76.51, –151.56, –151.61.
IR (neat): 2974, 2879, 1679, 1631, 1604, 1557, 1502, 1485, 1473, 1408, 1373, 1295, 1274, 1251, 1175, 1129, 1055, 1022, 973, 947, 901, 877, 856, 818, 795, 769, 746, 716, 672, 609, 555, 533, 517, 486, 447 cm^{-1}
HRMS (ESI) Calcd. For $C_{21}H_{32}N_3^+$ (M–BF_4)$^+$: 326.2591. Found: 326.2582.

***N,N,N,N',N',N'*-hexamethyl-4,4'-di-*tert*-butyl-2,2'-bipyridin-6,6'-diaminium bis(tetrafluoroborate) (4a)**

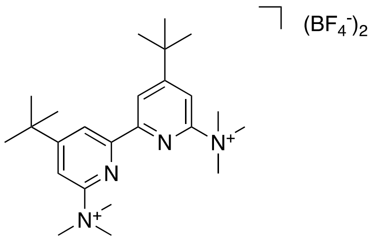

Compound **4a** was prepared according to General Procedure **A** using **S1** (150 mg, 0.5 mmol, 1 equiv), trimethylamine (2 M in THF, 2.5 mL, 5 mmol, 10 equiv), TFAA (420 µL, 3 mmol, 6 equiv), and CH_2Cl_2 (2.5 mL). The filter cake was dissolved in water (5 mL) and a precipitate formed following addition of a $NaBF_4$ solution (2 M in water, 20 mL). The precipitate was collected on a fritted funnel and washed with water (2 mL) and EtOAc (2 mL) to yield **4a** (130 mg, 47%) as a white powder.

^{1}H NMR (600 MHz, CD_3CN) δ 8.59 (d, *J* = 1.3 Hz, 2H), 7.85 (d, *J* = 1.3 Hz, 2H), 3.65 (s, 18H), 1.47 (s, 18H).
^{13}C NMR (151 MHz, CD_3CN) δ 168.8, 157.7, 154.0, 121.4, 113.7, 56.1, 37.0, 30.4.
^{19}F NMR (376 MHz, CD_3CN) δ –151.64, –151.69.
IR (neat): 2968, 2879, 1603, 1546, 1479, 1432, 1387, 1367, 1270, 1209, 1188, 1034, 998, 965, 942, 911, 897, 834, 750, 709, 664, 570, 545, 520 cm^{-1}
HRMS (ESI) Calcd. For $C_{24}H_{40}BF_4N_3^+$ $(M–BF_4)^+$: 471.3277. Found: 471.3289.

***N,N,N,N',N',N'*-hexamethyl-4,4'-di-*tert*-butyl-2,2'-bipyridin-6,6'-diaminium bis(trifluoroacetate) (4b)**

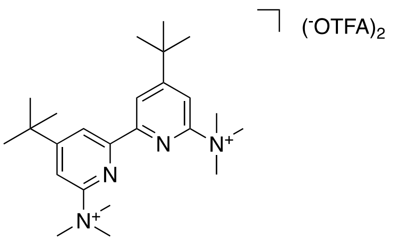

Compound **4b** was prepared according to a modified General Procedure **A** using **S1** (300 mg, 0.5 mmol, 1 equiv), trimethylamine (2 M in THF, 5 mL, 10 mmol, 10 equiv), TFAA (840 µL, 6 mmol, 6 equiv), and CH_2Cl_2 (5 mL). The filter cake was suspended in water (15 mL) and the insoluble solid was collected on a fritted funnel and washed with water and diethyl ether to yield **4b** (270 mg) as a white powder. A second crop of **4b** was obtained by concentrating the aqueous filtrate, suspending the resulting crystalline solid in water (2 mL), and collecting the insoluble solid on a fritted funnel, washing it with water (1 mL) and EtOAc (5 mL) to yield **4b** (100 mg) as a white crystalline solid. Combined total yield 370 mg, 61%.

^{1}H NMR (600 MHz, CD_3OD) δ 8.68 (s, 2H), 8.10 (s, 2H), 3.80 (s, 18H), 1.52 (s, 18H).
^{13}C NMR (151 MHz, CD_3OD) δ 169.5, 162.7 (q, *J* = 34.0 Hz), 158.5, 154.7, 121.6, 118.3 (q, *J* = 293.6 Hz), 113.9, 56.0, 37.3, 30.7.
^{19}F NMR (565 MHz, CD_3OD) δ –76.92.
IR (neat): 3496, 3443, 3281, 3044, 2999, 2972, 2879, 1685, 1671, 1600, 1557, 1539, 1498, 1486, 1469, 1417, 1387, 1367, 1270, 1197, 1174, 1115, 999, 968, 952, 900, 878, 825, 803, 748, 717, 665, 594, 566, 547, 499 cm^{-1}
HRMS (ESI) Calcd. For $C_{26}H_{40}F_3N_4O_2^+$ $(M–OTFA)^+$: 497.3103. Found: 497.3102.

***N,N,N*-trimethyl-4,4'-bis(methoxycarbonyl)-2,2'-bipyridin-6-aminium tetrafluoroborate (5)**

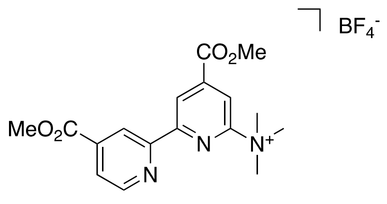

Compound **5** was prepared according to General Procedure **A** using 4,4'-bis(methoxycarbonyl)-2,2'-bipyridine *N*-oxide (288 mg, 1.0 mmol, 1 equiv), trimethylamine (2 M in THF, 2.5 mL, 5 mmol, 5 equiv), TFAA (420 µL, 3 mmol, 3 equiv) and CH_2Cl_2 (7.5 mL). The filter cake was dissolved in water (5 mL) and a precipitate formed following addition of a $NaBF_4$ solution (2 M in water, 40 mL). The precipitate was collected on a fritted funnel and washed with water (10 mL) and EtOAc (7 mL). The precipitate contained ~ 5% $TMABF_4$, so it was further washed with $NaBF_4$ (2 M in water, 40 mL), followed by water (5 mL) and EtOAc (5 mL) to yield **5** (324 mg, 78%) as a white powder.

^{1}H NMR (600 MHz, DMSO) δ 9.01 (dd, *J* = 5.0, 0.9 Hz, 1H), 9.00 (d, *J* = 1.0 Hz, 1H), 8.80 (dd, *J* = 1.6, 0.9 Hz, 1H), 8.58 (d, *J* = 1.0 Hz, 1H), 8.03 (dd, *J* = 5.0, 1.6 Hz, 1H), 4.03 (s, 3H), 3.97 (s, 3H), 3.76 (s, 9H).
^{13}C NMR (151 MHz, DMSO) δ 164.9, 163.7, 157.5, 154.7, 153.6, 151.2, 142.9, 138.8, 124.4, 121.6, 120.0, 115.4, 54.8, 53.5, 53.1.
^{19}F NMR (376 MHz, DMSO) δ –148.72, –148.78.
IR (neat): 3078, 2971, 1729, 1598, 1561, 1495, 1468, 1438, 1416, 1366, 1311, 1288, 1263, 1203, 1147, 1109, 1069, 1042, 1033, 992, 960, 948, 924, 903, 882, 875, 836, 800, 767, 743, 696, 670, 662, 592, 551, 522, 507, 477 cm^{-1}
HRMS (ESI) Calcd. For $C_{17}H_{20}N_3O_4^+$ $(M–BF_4)^+$: 330.1448. Found: 330.1451.

***N,N,N,N',N',N'*-hexamethyl-5,5'-dimethyl-2,2'-bipyridin-6,6'-diaminium bis(tetrafluoroborate) (6)**

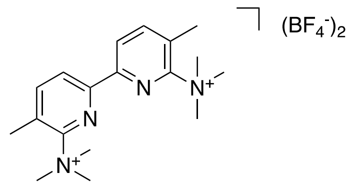

Compound **6** was prepared according to General Procedure **A** using **S2** (216 mg, 1.0 mmol, 1 equiv), trimethylamine (2 M in THF, 5 mL, 10 mmol, 10 equiv), TFAA (840 µL, 6 mmol, 6 equiv) and CH_2Cl_2 (5 mL). The filter cake was dissolved in water (3 mL) and a precipitate formed following addition of a $NaBF_4$ solution (2 M in water, 40 mL). The precipitate was collected on a fritted funnel and washed with water (5 mL) and EtOAc (5 mL). The precipitate contained ~ 10% $TMABF_4$, so it was further washed with $NaBF_4$ (2 M in water, 40 mL), followed by water (5 mL) and EtOAc (5 mL) to yield **6** (227 mg, 48%) as a white powder.

^{1}H NMR (600 MHz, CD_3CN) δ 8.49 (d, *J* = 7.9 Hz, 2H), 8.10 (d, *J* = 7.8 Hz, 2H), 3.69 (s, 18H), 2.76 (s, 6H).
^{13}C NMR (151 MHz, CD_3CN) δ 153.9, 150.8, 148.3, 128.4, 124.4, 56.0, 21.5.
^{19}F NMR (376 MHz, CD_3CN) δ –151.91, –151.96.
IR (neat): 1609, 1543, 1492, 1462, 1437, 1370, 1287, 1258, 1226, 1155, 1030, 942, 862, 847, 807, 696, 615, 596, 551, 520 cm^{-1}
HRMS (ESI) Calcd. For $C_{18}H_{28}BF_4N_4^+$ $(M–BF_4)^+$: 387.2338. Found: 387.2351.

***N,N,N,N',N',N'*-hexamethyl-2,2'-bipyridin-6,6'-diaminium bis(tetrafluoroborate)-d_6 (7)**

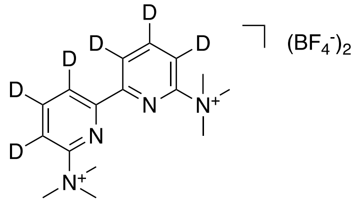

Compound **7** was prepared according to General Procedure **A** using D_8-2,2'-bipyridine *N,N'*-dioxide (196 mg, 1.0 mmol, 1 equiv), trimethylamine (2 M in THF, 5 mL, 10 mmol, 10 equiv), TFAA (840 µL, 6 mmol, 6 equiv) and CH_2Cl_2 (5 mL). The filter cake was dissolved in water (3 mL) and a precipitate formed following addition of a $NaBF_4$ solution (2 M in water, 40 mL). The precipitate was collected on a fritted funnel and washed with water (5 mL) and EtOAc (5 mL). The precipitate contained ~ 1.3 equiv $TMABF_4$, so it was further washed with 40 mL of $NaBF_4$ (2 M in water), followed by water (5 mL) and EtOAc (5 mL) to yield **7** (161 mg, 36%) as a white powder.

^{1}H NMR (600 MHz, CD_3CN) δ 3.64 (s, 18H).
^{2}H NMR (92 MHz, CD_3CN) δ 8.74 (br s, 2H), 8.37 (br s, 2H), 8.00 (br s, 2H).
^{13}C NMR (151 MHz, CD_3CN) δ 157.1, 154.0, 143.8 – 142.6 (m), 124.8 – 123.7 (m), 117.1 – 115.8 (m), 56.0.
^{19}F NMR (376 MHz, CD_3CN) δ –151.61, –151.66.
IR (neat): 3056, 2338, 2322, 2293, 1577, 1534, 1494, 1473, 1419, 1367, 1318, 1290, 1261, 1106, 1032, 969, 946, 932, 860, 846, 830, 767, 733, 720, 613, 604, 571, 521, 436 cm^{-1}
HRMS (ESI) Calcd. For $C_{16}H_{18}D_6BF_4N_4^+$ $(M–BF_4)^+$: 364.2443. Found: 364.2444.

***N,N,N*-trimethyl-1,10-phenanthrolin-2-aminium tetrafluoroborate (8)**

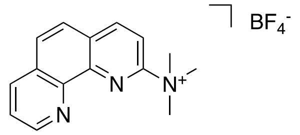

Compound **8** was prepared according to a modified General Procedure **A** using 1,10-phenanthroline *N*-oxide (196 mg, 1.0 mmol, 1 equiv), trimethylamine (2 M in THF, 2.5 mL, 5 mmol, 5 equiv), TFAA (420 µL, 3 mmol, 3 equiv), and CH_2Cl_2 (7.5 mL). The filter cake was dissolved in water (2 mL) and a precipitate formed following addition of a $NaBF_4$ solution (2 M in water, 20 mL). The precipitate was collected on a fritted funnel and washed with water (5 mL) and EtOAc (5 mL) to yield **8** (208 mg) as a white powder. Colorless crystals formed in the filtrate overnight, which were collected on a fritted funnel and washed with diethyl ether to yield a second crop of **8** (37 mg) as colorless needles. Combined total yield: 245 mg, 75%.

^{1}H NMR (600 MHz, CD_3CN) δ 9.16 (dd, J = 4.3, 1.7 Hz, 1H), 8.78 (d, J = 8.8 Hz, 1H), 8.48 (dd, J = 8.1, 1.7 Hz, 1H), 8.16 (d, J = 8.8 Hz, 1H), 8.09 (d, J = 8.9 Hz, 1H), 8.04 (d, J = 8.8 Hz, 1H), 7.81 (dd, J = 8.1, 4.3 Hz, 1H), 3.74 (s, 9H).
^{13}C NMR (151 MHz, CD_3CN) δ 155.9, 151.6, 145.5, 144.7, 142.8, 137.8, 130.8, 130.5, 130.3, 126.5, 125.4, 114.4, 56.2.
^{19}F NMR (376 MHz, CD_3CN) δ –151.68, –151.73.
IR (neat): 3074, 3025, 1622, 1590, 1569, 1496, 1463, 1454, 1412, 1289, 1173, 1155, 1135, 1033, 965, 951, 884, 848, 829, 779, 743, 715, 663, 624, 578, 521 cm^{-1}
HRMS (ESI) Calcd. For $C_{15}H_{16}N_3^+$ $(M–BF_4)^+$: 238.1339. Found: 238.1344.

***N,N,N*-trimethyl-6-phenylpyridin-2-aminium tetrafluoroborate (9)**

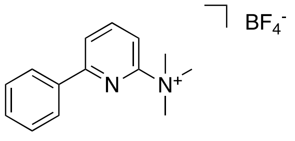

Compound **9** was prepared according to a modified General Procedure **A** using 2-phenylpyridine *N*-oxide (171 mg, 1 mmol, 1 equiv), trimethylamine (2 M in THF, 2.5 mL, 5 mmol, 5 equiv), TFAA (420 µL, 3 mmol, 3 equiv), and CH_2Cl_2 (7.5 mL). After stirring at room temperature overnight, the flask was opened to air and the reaction mixture was charged with CH_2Cl_2 (1 mL). The cloudy solution was slowly filtered through a fine fritted funnel, and the white filter cake (TMAOTFA) was washed with CH_2Cl_2. The filtrate was concentrated *in vacuo* to yield an off-white oil. The oil was diluted with water (4 mL) and subsequently charged with a $NaBF_4$ solution (2 M in water, 2 mL) to form a white cloudy solution. The flask was scratched to assist with precipitation of a white powder. The powder was collected on a fritted funnel and washed with water (6 mL) and EtOAc (5 mL) to yield **9** (229 mg, 76%) as a white powder.

^{1}H NMR (600 MHz, CD_3CN) δ 8.20 – 8.14 (m, 3H), 8.12 (d, *J* = 7.8 Hz, 1H), 7.77 (d, *J* = 8.1 Hz, 1H), 7.59 – 7.50 (m, 3H), 3.61 (s, 9H).
^{13}C NMR (151 MHz, CD_3CN) δ 157.5, 157.1, 142.8, 137.5, 131.4, 130.0, 128.0, 123.3, 113.4, 55.8.
^{19}F NMR (376 MHz, CD_3CN) δ –151.45, –151.50.
IR (neat): 3101, 3064, 1601, 1558, 1490, 1456, 1443, 1408, 1305, 1288, 1194, 1024, 992, 965, 942, 917, 860, 816, 792, 769, 742, 700, 665, 621, 535, 521, 498 cm^{-1}
HRMS (ESI) Calcd. For $C_{14}H_{17}N_2^+$ $(M–BF_4)^+$: 213.1386. Found: 213.1391.

***N,N,N*-trimethyl-benzo[h]quinolin-2-aminium tetrafluoroborate (10)**

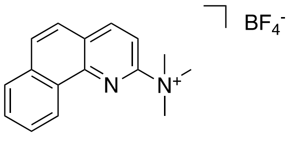

Compound **10** was prepared according to General Procedure **A** using benzo[h]quinoline *N*-oxide (195 mg, 1 mmol, 1 equiv), trimethylamine (2 M in THF, 2.5 mL, 5 mmol, 5 equiv), TFAA (420 µL, 3 mmol, 3 equiv), and CH_2Cl_2 (7.5 mL). The filter cake was dissolved in water (5 mL) and a precipitate formed following addition of a $NaBF_4$ solution (2 M in water, 40 mL). The precipitate was collected on a fritted funnel and washed with water (5 mL) and EtOAc (5 mL) to yield **10** (255 mg, 79%) as a white powder.

^{1}H NMR (600 MHz, CD_3CN) δ 9.22 – 9.16 (m, 1H), 8.68 (d, *J* = 8.7 Hz, 1H), 8.09 – 8.03 (m, 3H), 7.92 (d, *J* = 8.9 Hz, 1H), 7.87 – 7.81 (m, 2H), 3.74 (s, 9H).
^{13}C NMR (151 MHz, CD_3CN) δ 155.1, 145.1, 142.3, 135.2, 131.0, 131.0, 130.6, 129.2, 128.8, 128.1, 125.3, 125.3, 113.1, 56.1.
^{19}F NMR (376 MHz, CD_3CN) δ –151.70, –151.75.
IR (neat): 3129, 3064, 1624, 1591, 1575, 1520, 1491, 1464, 1442, 1406, 1344, 1285, 1233, 1172, 1158, 1031, 960, 946, 886, 850, 824, 803, 762, 743, 717, 666, 624, 572, 520, 453, 432 cm^{-1}
HRMS (ESI) Calcd. For $C_{16}H_{17}N_2^+$ $(M–BF_4)^+$: 237.1386. Found: 237.1392.

***N,N,N,N'',N'',N''*-hexamethyl-2,2':6',2"-terpyridin-6,6''-diaminium bis(tetrafluoroborate) (11)**

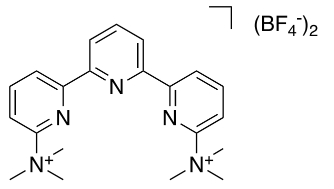

Compound **11** was prepared according to General Procedure **A** using 2,2':6',2"-terpyridine *N,N*"-dioxide (265 mg, 1.0 mmol, 1 equiv), trimethylamine (2 M in THF, 5 mL, 10 mmol, 10 equiv), TFAA (840 µL, 6 mmol, 6 equiv) and CH_2Cl_2 (5 mL). The filter cake was dissolved in water (2 mL) and a precipitate formed following addition of a $NaBF_4$ solution (2 M in water, 40 mL). The precipitate was collected on a fritted funnel and washed with water (5 mL) and EtOAc (5 mL). The precipitate contained $TMABF_4$, so it was further washed with $NaBF_4$ (2 M in water, 2 x 40 mL), followed by water (10 mL) and EtOAc (10 mL) to yield **11** (355 mg, 68%) as a white powder.

^{1}H NMR (600 MHz, DMSO) δ 8.89 (d, J = 7.7 Hz, 2H), 8.65 (d, J = 7.8 Hz, 2H), 8.43 (t, J = 8.0 Hz, 2H), 8.26 (t, J = 7.8 Hz, 1H), 8.19 (d, J = 8.2 Hz, 2H), 3.71 (s, 18H).
^{13}C NMR (151 MHz, DMSO) δ 156.5, 154.0, 153.2, 142.5, 139.4, 122.7, 122.7, 115.6, 54.7.
^{19}F NMR (376 MHz, DMSO) δ –148.73, –148.78.
IR (neat): 3640, 3101, 3063, 1691, 1638, 1604, 1561, 1494, 1477, 1432, 1018, 995, 948, 857, 820, 799, 745, 718, 652, 632, 577, 550, 519 cm^{-1}
HRMS (ESI) Calcd. For $C_{21}H_{27}BF_4N_5^+$ $(M–BF_4)^+$: 436.2290. Found: 436.2289.

***N,N,N,N''',N''',N'''*-hexamethyl-2,2':6',2":6",2"'-quaterpyridin-6,6'''-diaminium bis(tetrafluoroborate) (12)**

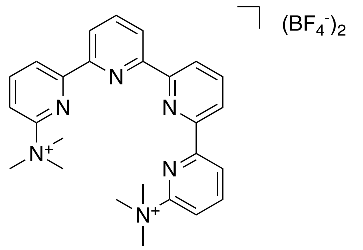

Compound **12** was prepared according to General Procedure **A** using **S3** (342 mg, 0.5 mmol, 1 equiv), trimethylamine (2 M in THF, 5 mL, 10 mmol, 10 equiv), TFAA (840 µL, 6 mmol, 6 equiv), and CH_2Cl_2 (5 mL). The filter cake was dissolved in water (5 mL) and a precipitate formed following addition of a $NaBF_4$ solution (2 M in water, 40 mL). The precipitate was collected on a fritted funnel and washed with water (10 mL) and EtOAc (5 mL) to yield **12** (507 mg, 84%) as a white powder.

^{1}H NMR (600 MHz, CD_3CN) δ 8.89 (d, J = 7.8 Hz, 2H), 8.78 (d, J = 7.9 Hz, 2H), 8.56 (d, J = 7.8 Hz, 2H), 8.31 (t, J = 8.0 Hz, 2H), 8.17 (t, J = 7.8 Hz, 2H), 7.90 (d, J = 8.2 Hz, 2H), 3.66 (s, 18H).
^{13}C NMR (151 MHz, CD_3CN) δ 157.0, 156.4, 156.2, 154.0, 143.1, 139.7, 123.9, 123.1, 122.8, 115.4, 56.0.
^{19}F NMR (376 MHz, CD_3CN) δ –151.65, –151.70.
IR (neat): 3094, 3059, 1600, 1585, 1565, 1490, 1467, 1422, 1372, 1293, 1275, 1183, 1157, 1055, 1023, 991, 951, 932, 868, 800, 746, 703, 676, 636, 521 cm^{-1}
HRMS (ESI) Calcd. For $C_{26}H_{30}BF_4N_6$ $(M-BF_4)^+$: 513.2566. Found: 513.2573.

***P,P,P,P',P',P'*-hexa(*p*-tolyl)-4,4'-dimethyl-2,2'-bipyridin-6,6'-diphosphinium bis(trifluoroacetate) (13)**

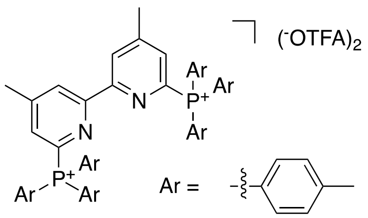

Compound **13** was prepared according to General Procedure **B** using 4,4'-dimethyl-2,2'-bipyridine *N,N'*-dioxide (108 mg, 0.5 mmol, 1 equiv), tri(*p*-tolyl)phosphine (457 mg, 1.5 mmol, 3 equiv), TFAA (220 µL, 1.5 mmol, 3 equiv), and CH_2Cl_2 (2.5 mL). The precipitate was collected on a fritted funnel to yield **13** (482 mg, 95%) as a tan powder.

^{1}H NMR (600 MHz, CD_3OD) δ 8.35 – 8.31 (m, 2H), 7.81 – 7.76 (m, 2H), 7.70 (dd, *J* = 12.8, 8.3 Hz, 12H), 7.61 (dd, *J* = 8.2, 3.4 Hz, 12H), 2.54 (s, 18H), 2.52 (s, 6H).
^{13}C NMR (151 MHz, CD_3OD) δ 161.6, 161.4, 161.1, 160.9, 157.6, 157.5, 153.4, 153.4, 148.5, 148.5, 147.0, 146.2, 136.1, 136.0, 134.3, 134.1, 132.2, 132.1, 127.3, 127.3, 120.3, 118.3, 116.4, 116.0, 115.3, 21.8, 21.8, 21.6, 21.6.
^{19}F NMR (376 MHz, CD_3OD) δ –77.44.
^{31}P NMR (162 MHz, CD_3OD) δ 18.13.
IR (neat): 3063, 2933, 1776, 1731, 1598, 1540, 1499, 1447, 1402, 1385, 1313, 1187, 1136, 1109, 1039, 1016, 988, 881, 856, 835, 804, 789, 761, 732, 704, 662, 637, 614, 585, 550, 514, 496, 465, 438 cm^{-1}
HRMS (ESI) Calcd. For $C_{56}H_{52}F_3N_2O_2P_2^+$ $(M–OTFA)^+$: 903.3451. Found: 903.3474.

***P,P,P,P',P',P'*-hexa(*p*-tolyl)-6,6'-dimethyl-2,2'-bipyridin-4,4'-diphosphinium bis(trifluoroacetate) (14)**

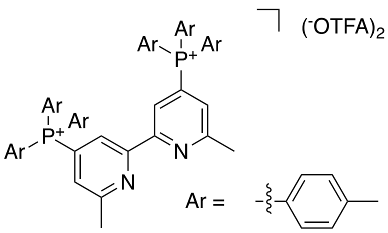

Compound **14** was prepared according to General Procedure **B** using 6,6'-dimethyl-2,2'-bipyridine *N,N'*-dioxide (108 mg, 0.5 mmol, 1 equiv), tri(*p*-tolyl)phosphine (457 mg, 1.5 mmol, 3 equiv), TFAA (220 µL, 1.5 mmol, 3 equiv), and CH_2Cl_2 (2.5 mL). The precipitate was collected on a fritted funnel to yield **13** (468 mg, 92%) as a white powder.

^{1}H NMR (600 MHz, CD_3OD) δ 8.52 (d, *J* = 15.0 Hz, 2H), 7.69 – 7.63 (m, 24H), 7.62 (d, *J* = 13.0 Hz, 2H), 2.61 (s, 6H), 2.55 (s, 18H).
^{13}C NMR (151 MHz, CD_3OD) δ 162.4, 162.3, 161.7, 161.4, 161.2, 156.4, 156.4, 156.4, 149.0, 149.0, 135.9, 135.9, 135.7, 133.2, 132.7, 132.5, 132.4, 129.1, 129.0, 123.1, 123.0, 118.3, 116.4, 115.0, 114.4, 24.7, 21.8.
^{19}F NMR (376 MHz, CD_3OD) δ –77.48.
^{31}P NMR (162 MHz, CD_3OD) δ 23.41.
IR (neat): 3066, 3044, 2984, 2930, 2870, 1780, 1737, 1597, 1570, 1553, 1499, 1446, 1401, 1385, 1362, 1316, 1184, 1137, 1107, 1038, 1016, 870, 838, 806, 790, 703, 660, 611, 587, 552, 517, 472, 442 cm^{-1}
HRMS (ESI) Calcd. For $C_{56}H_{52}F_3N_2O_2P_2^+$ $(M–OTFA)^+$: 903.3451. Found: 903.3433.

***P,P,P,P',P',P'*-hexaphenyl-4,4'-dimethoxy-2,2'-bipyridin-6,6'-diphosphinium bis(trifluoroacetate) (15)**

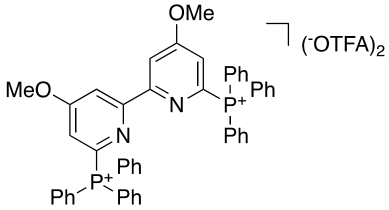

Compound **15** was prepared according to General Procedure **B** using 4,4'-dimethoxy-2,2'-bipyridine *N,N*'-dioxide (124 mg, 0.5 mmol, 1 equiv), triphenylphosphine (393 mg, 1.5 mmol, 3 equiv), TFAA (220 µL, 1.5 mmol, 3 equiv), and CH_2Cl_2 (5 mL). The precipitate was collected on a fritted funnel to yield **15** (444 mg, 92%) as a white powder.

^{1}H NMR (600 MHz, CD_3OD) δ 8.01 – 7.94 (m, 8H), 7.88 (ddd, *J* = 13.0, 8.4, 1.4 Hz, 12H), 7.81 (td, *J* = 7.9, 3.7 Hz, 10H), 7.49 (dd, *J* = 7.4, 2.3 Hz, 2H), 3.93 (s, 6H).
^{13}C NMR (151 MHz, CD_3OD) δ 169.5, 169.4, 161.7, 161.4, 161.2, 160.9, 159.5, 159.4, 147.5, 146.7, 136.9, 136.9, 136.3, 136.2, 131.6, 131.5, 121.9, 121.7, 120.2, 118.9, 118.3, 118.3, 116.4, 114.5, 111.0, 111.0, 57.1.
^{19}F NMR (376 MHz, CD_3OD) δ –77.46.
^{31}P NMR (162 MHz, CD_3OD) δ 19.73.
IR (neat): 3093, 3066, 2990, 2949, 1778, 1732, 1689, 1577, 1548, 1485, 1402, 1378, 1295, 1266, 1191, 1137, 1111, 1037, 998, 985, 871, 789, 757, 728, 704, 687, 615, 589, 565, 543, 526, 481 cm^{-1}
HRMS (ESI) Calcd. For $C_{50}H_{40}F_3N_2O_4P_2^+$ $(M\text{-}OTFA)^+$: 851.2415. Found: 851.2411.

***P,P,P*-triphenyl-4,4'-dimethyl-2,2'-bipyridin-6-phosphinium trifluoroacetate (16)**

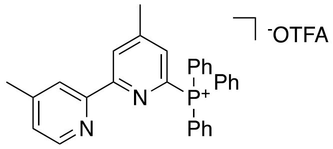

Compound **16** was prepared according to a modified General Procedure **B** using 4,4'-dimethyl-2,2'-bipyridine *N*-oxide (401 mg, 2 mmol, 1 equiv), triphenylphosphine (787 mg, 3 mmol, 1.5 equiv), TFAA (440 µL, 3 mmol, 1.5 equiv), and CH_2Cl_2 (10 mL). After the solution was opened to air, transferred to a 50 mL round bottom flask, and concentrated *in vacuo*, the resulting crude material was purified by column chromatography (70 mL SiO_2, 9:1 CH_2Cl_2:MeOH, UV). Upon combining the fractions containing **16** and concentrating *in vacuo*, the resulting oil was layered with diethyl ether (25 mL) and triturated with the assistance of sonication to form a cloudy solution. The flask was scratched with a glass stir rod and placed in a 0 °C freezer overnight to yield a tan precipitate. The precipitate was collected on a fritted funnel and washed with diethyl ether to yield **16** (787 mg, 70%) as a tan powder.

^{1}H NMR (600 MHz, CD_3OD) δ 8.62 (dt, *J* = 2.4, 1.2 Hz, 1H), 8.54 (d, *J* = 5.0 Hz, 1H), 8.00 – 7.94 (m, 4H), 7.87 (ddd, *J* = 12.9, 8.5, 1.4 Hz, 6H), 7.82 (ddd, *J* = 8.3, 7.4, 3.8 Hz, 6H), 7.79 – 7.76 (m, 1H), 7.32 (ddd, *J* = 5.0, 1.7, 0.9 Hz, 1H), 2.56 (s, 3H), 2.40 (s, 3H).
^{13}C NMR (151 MHz, CD_3OD) δ 163.0, 162.7, 159.6, 159.5, 155.0, 153.0, 152.9, 150.7, 150.4, 145.7, 144.9, 136.8, 136.7, 136.2, 136.2, 133.7, 133.5, 131.6, 131.5, 127.2, 127.1, 123.6, 119.3, 118.7, 21.5, 21.5, 21.2.
^{19}F NMR (376 MHz, CD_3OD) δ −77.04.
^{31}P NMR (162 MHz, CD_3OD) δ 18.20.
IR (neat): 3055, 3022, 1686, 1651, 1586, 1540, 1482, 1439, 1410, 1396, 1372, 1322, 1196, 1151, 1109, 1039, 996, 984, 877, 846, 816, 797, 753, 736, 726, 715, 692, 633, 615, 545, 535, 523, 490 cm^{-1}
HRMS (ESI) Calcd. For $C_{30}H_{26}N_2P^+$ $(M\text{–}OTFA)^+$: 445.1833. Found: 445.1847.

***P,P,P,P',P',P'*-hexa(*p*-tolyl)-4,4'-di-*tert*-butyl-2,2'-bipyridin-6,6'-diphosphinium bis(trifluoroacetate) (17)**

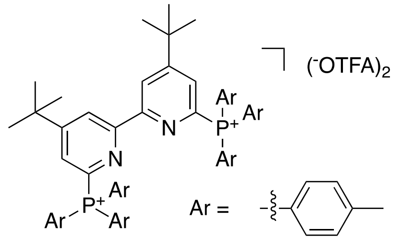

Compound **17** was prepared according to General Procedure **B** using 4,4'-di-*tert*-butyl-2,2'-bipyridine *N*,*N*'-dioxide (150 mg, 0.5 mmol, 1 equiv), tri(*p*-tolyl)phosphine (457 mg, 1.5 mmol, 3 equiv), TFAA (220 µL, 1.5 mmol, 3 equiv), and CH_2Cl_2 (5 mL). The precipitate was collected on a fritted funnel to yield **17** (532 mg, 97%) as a white powder.

^{1}H NMR (600 MHz, CD_3OD) δ 8.45 (dd, *J* = 2.3, 1.7 Hz, 2H), 7.92 (dd, *J* = 6.5, 1.7 Hz, 2H), 7.71 (dd, *J* = 12.8, 8.4 Hz, 12H), 7.63 (dd, *J* = 8.5, 3.2 Hz, 12H), 2.55 (s, 18H), 1.30 (s, 18H).
^{13}C NMR (151 MHz, CD_3OD) δ 165.5, 165.5, 161.6, 161.4, 161.1, 160.9, 157.9, 157.7, 148.6, 148.6, 147.2, 146.4, 136.1, 136.0, 132.3, 132.2, 130.6, 130.4, 123.5, 123.5, 120.2, 118.3, 116.4, 115.9, 115.3, 114.5, 36.7, 36.7, 30.4, 21.8, 21.8.
^{19}F NMR (376 MHz, CD_3OD) δ –77.44.
^{31}P NMR (162 MHz, CD_3OD) δ 19.65.
IR (neat): 2962, 2930, 2870, 1782, 1740, 1598, 1588, 1531, 1499, 1486, 1448, 1400, 1378, 1314, 1283, 1268, 1181, 1136, 1107, 1043, 1019, 989, 916, 876, 788, 740, 701, 662, 638, 613, 588, 528, 516, 493, 473, 443 cm^{-1}
HRMS (ESI) Calcd. For $C_{62}H_{64}F_3N_2O_2P_2^+$ (M–OTFA)$^+$: 987.4390. Found: 987.4393.

[2,2'-biquinolin]-4-yltriphenylphosphonium trifluoroacetate (18)

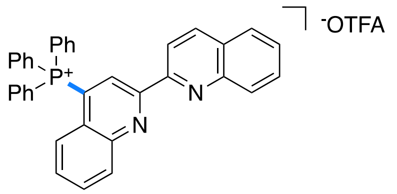

Compound **18** was prepared according to a modified General Procedure **B** using 2,2'-biquinoline *N*-oxide (272 mg, 1 mmol, 1 equiv), triphenylphosphine (393 mg, 1.5 mmol, 1.5 equiv), TFAA (210 µL, 1.5 mmol, 1.5 equiv), and CH_2Cl_2 (10 mL). Upon concentration *in vacuo*, the resulting yellow oil was diluted with CH_2Cl_2 (~ 1 mL) before adding diethyl ether (~ 50 mL). The flask was scratched with a glass stir rod and the solution was triturated with the assistance of sonication to form a shimmery white precipitate. The precipitate was collected on a fritted funnel and washed with diethyl ether to yield **18** (630 mg, quant.) as an off-white powder.

^{1}H NMR (600 MHz, CD_2Cl_2) δ 9.06 (d, *J* = 18.4 Hz, 1H), 8.85 (d, *J* = 8.5 Hz, 1H), 8.51 (d, *J* = 8.4 Hz, 1H), 8.42 (d, *J* = 8.6 Hz, 1H), 8.04 – 7.98 (m, 3H), 7.97 – 7.91 (m, 2H), 7.84 – 7.80 (m, 7H), 7.80 – 7.73 (m, 7H), 7.66 – 7.59 (m, 1H), 7.55 – 7.49 (m, 1H), 7.46 (d, *J* = 8.2 Hz, 1H).
^{13}C NMR (151 MHz, CD_2Cl_2) δ 160.3 (q, *J* = 36.2 Hz), 156.2 (d, *J* = 12.2 Hz), 153.8, 149.2 (d, *J* = 7.2 Hz), 147.9, 137.9, 136.6 (d, *J* = 3.1 Hz), 134.9, 134.9, 132.6 (d, *J* = 2.4 Hz), 132.0, 131.4, 131.3, 130.5, 130.2 (d, *J* = 3.0 Hz), 130.1 (d, *J* = 3.3 Hz), 129.2, 128.3, 128.2, 126.5 (d, *J* = 6.7 Hz), 126.2 (d, *J* = 6.0 Hz), 125.9, 125.4, 118.9, 117.6, 117.0, 116.8 (q, *J* = 291.2 Hz).
^{19}F NMR (376 MHz, CD_2Cl_2) δ –76.07.
^{31}P NMR (162 MHz, CD_2Cl_2) δ 23.19.

IR (neat): 3059, 1787, 1737, 1690, 1616, 1582, 1548, 1497, 1484, 1438, 1397, 1319, 1299, 1193, 1137, 1105, 1069, 1027, 996, 951, 838, 813, 796, 756, 725, 709, 689, 628, 615, 588, 573, 541, 524, 508, 495, 476, 441 cm^{-1}
HRMS (ESI) Calcd. For $C_{36}H_{26}N_2P^+$ $(M–OTFA)^+$: 517.1833. Found: 517.1833.

1,1'-dimethyl-[2,2'-bipyridine]-1,1'-diium bis(trifluoromethanesulfonate) (19)

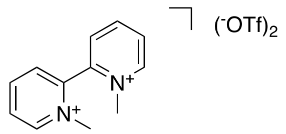

An oven-dried 20 mL scintillation vial equipped with a magnetic stir bar was brought into a N_2-filled glovebox and charged with 2,2'-bipyridine (156 mg, 1.0 mmol, 1 equiv) and CH_2Cl_2 (10 mL). The reaction is initiated by slow addition of MeOTf (340 µL, 3.0 mmol, 3 equiv) at room temperature. The flask was capped, sealed with electrical tape, removed from the glovebox, and allowed to stir at room temperature for 48 h, over which time the colorless solution turned into a viscous white slurry. After 48 h, the flask is opened to air and the white precipitate was collected on a fritted funnel and washed with CH_2Cl_2 and diethyl ether to yield **19** (459 mg, 95%) as a white powder.

^{1}H NMR (600 MHz, CD_3CN) δ 9.09 – 9.03 (m, 2H), 8.77 (td, J = 7.9, 1.4 Hz, 2H), 8.36 – 8.27 (m, 4H), 4.12 (s, 6H).
^{13}C NMR (151 MHz, CD_3CN) δ 150.3, 148.3, 143.9, 132.0, 131.6, 121.8 (q, J = 320.5 Hz), 48.6.
^{19}F NMR (565 MHz, CD_3CN) δ –79.39.
IR (neat): 3091, 3063, 1625, 1586, 1512, 1461, 1247, 1225, 1166, 1147, 1075, 1031, 1024, 815, 798, 790, 760, 634, 572, 538, 516, 445 cm^{-1}
HRMS (ESI) Calcd. For $C_{13}H_{14}F_3N_2O_3S^+$ $(M–OTf)^+$: 335.0677. Found: 335.0672.

([2,2'-biquinolin]-4-yltriphenylphosphonium)chlororhenium tricarbonyl trifluoroacetate (20)

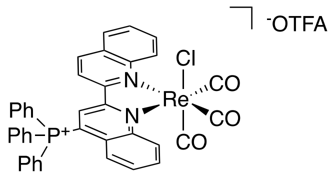

An oven-dried 100 mL round bottom flask equipped with a magnetic stir bar, a reflux condenser, and a rubber septum was allowed to cool to room temperature under vacuum before being backfilled with nitrogen. At this point, the condenser was removed and the vial was charged with **18** (158 mg, 0.25 mmol, 1 equiv) and $Re(CO)_5Cl$ (136 mg, 0.375 mmol, 1.5 equiv). The reflux condenser was returned, and the flask was evacuated and backfilled with nitrogen three times. Under nitrogen, the flask was charged with EtOH (25 mL). The flask was placed in a preheated oil bath and the reaction was heated to reflux for 24 h under N_2, over which time the colorless suspension became deep red. After 24 h, the oil bath was removed and the reaction mixture was allowed to cool to room temperature under N_2. At room temperature, the flask was opened to air and the solution was filtered through a plug of Celite®. The flask and Celite® were further washed with methanol. The red filtrate was concentrated *in vacuo*. The resulting red oil was diluted with CH_2Cl_2 (~ 5 mL) and charged with diethyl ether (~ 50 mL) to yield a red brown precipitate. The contents of the flask were triturated with the assistance of sonication to assist with precipitation. The precipitate was collected on a fritted funnel and washed with diethyl ether to yield **20** (198 mg, 84%) as a red brown powder.

^{1}H NMR (600 MHz, CD_3CN) δ 9.20 (dd, *J* = 9.0, 2.1 Hz, 1H), 8.90 (d, *J* = 8.7 Hz, 1H), 8.72 (d, *J* = 8.5 Hz, 1H), 8.32 (d, *J* = 16.7 Hz, 1H), 8.16 (ddd, *J* = 14.4, 7.0, 4.1 Hz, 2H), 8.11 (ddd, *J* = 8.6, 6.8, 1.5 Hz, 1H), 8.00 (td, *J* = 7.3, 1.9 Hz, 3H), 7.90 – 7.76 (m, 14H), 7.69 – 7.62 (m, 2H).
^{13}C NMR (151 MHz, CD_3CN) δ 198.1, 197.9, 189.5, 160.7 (d, *J* = 13.4 Hz), 159.5, 149.6 (d, *J* = 8.2 Hz), 149.0, 142.8, 137.2, 137.2, 136.0 (d, *J* = 10.8 Hz), 135.1, 134.6, 133.1, 132.5, 131.8 (d, *J* = 13.4 Hz), 131.3 (d, *J* = 11.0 Hz), 131.1, 130.8, 130.8, 130.4, 130.2, 130.1, 128.4 (d, *J* = 6.0 Hz), 128.0 (d, *J* = 7.0 Hz), 121.7, 117.4, 116.8.
^{19}F NMR (565 MHz, CD_3CN) δ –75.30.
^{31}P NMR (243 MHz, CD_3CN) δ 24.52 (d, *J* = 23.9 Hz).
UV–Vis [λ_{max} (ε)] 471 nm (3970), 393 nm (19710), 375 nm (21320), 359 nm (13630), 317 nm (12970), 269 nm (44260)
IR (neat): 3061, **2013**, **1899**, **1884**, 1688, 1585, 1508, 1486, 1437, 1411, 1379, 1355, 1340, 1289, 1211, 1197, 1154, 1104, 996, 962, 862, 823, 800, 790, 754, 727, 712, 688, 643, 592, 555, 545, 516, 494 cm^{-1}
HRMS (ESI) Calcd. For $C_{39}H_{26}ClN_2O_3PRe^+$ (M–OTFA)$^+$: 823.0920. Found: 823.0934.

(2,2'-biquinoline)chlororhenium tricarbonyl (21)

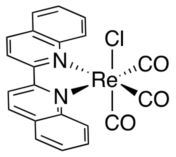

Compound **21** was made according to a literature procedure.[18]

UV–Vis [λ_{max} (ε)] 426 nm (3550), 376 nm (31840), 358 nm (20680), 305(s) nm (14102), 269 nm (55110)
IR (neat): 3088, 3063, **2012**, **1895**, **1866**, 1616, 1594, 1508, 1454, 1432, 1378, 1359, 1337, 1288, 1248, 1211, 1181, 1155, 1142, 1101, 980, 955, 867, 822, 779, 746, 696, 656, 631, 569, 535, 490, 479 cm^{-1}

V. Electrochemistry and UV–Vis Spectroscopy

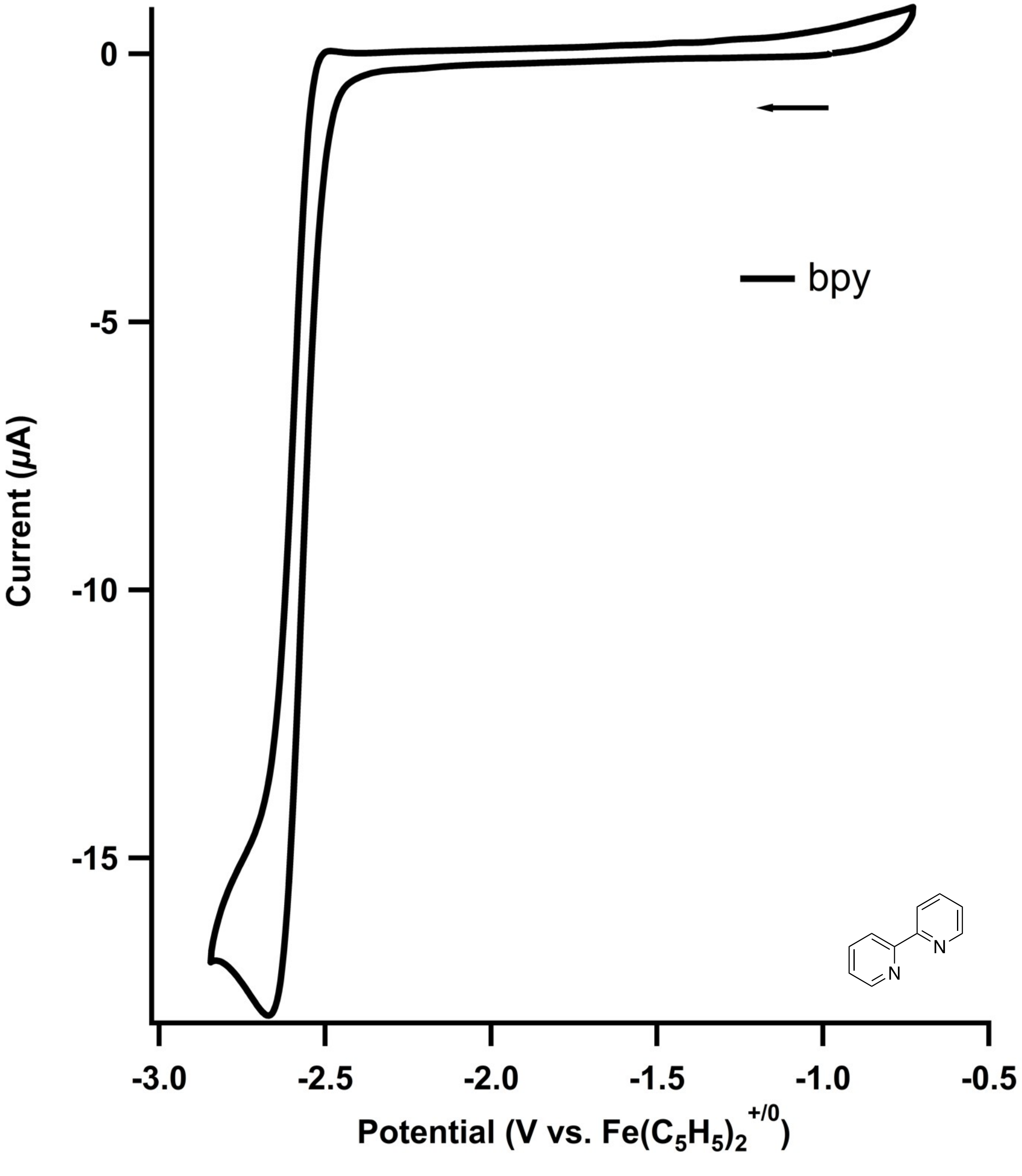

Figure S1. CV of 2,2'-bipyridine (2 mM) in MeCN using $TBAPF_6$ (0.2 M) as the supporting electrolyte. Scan rate = 100 mV/s

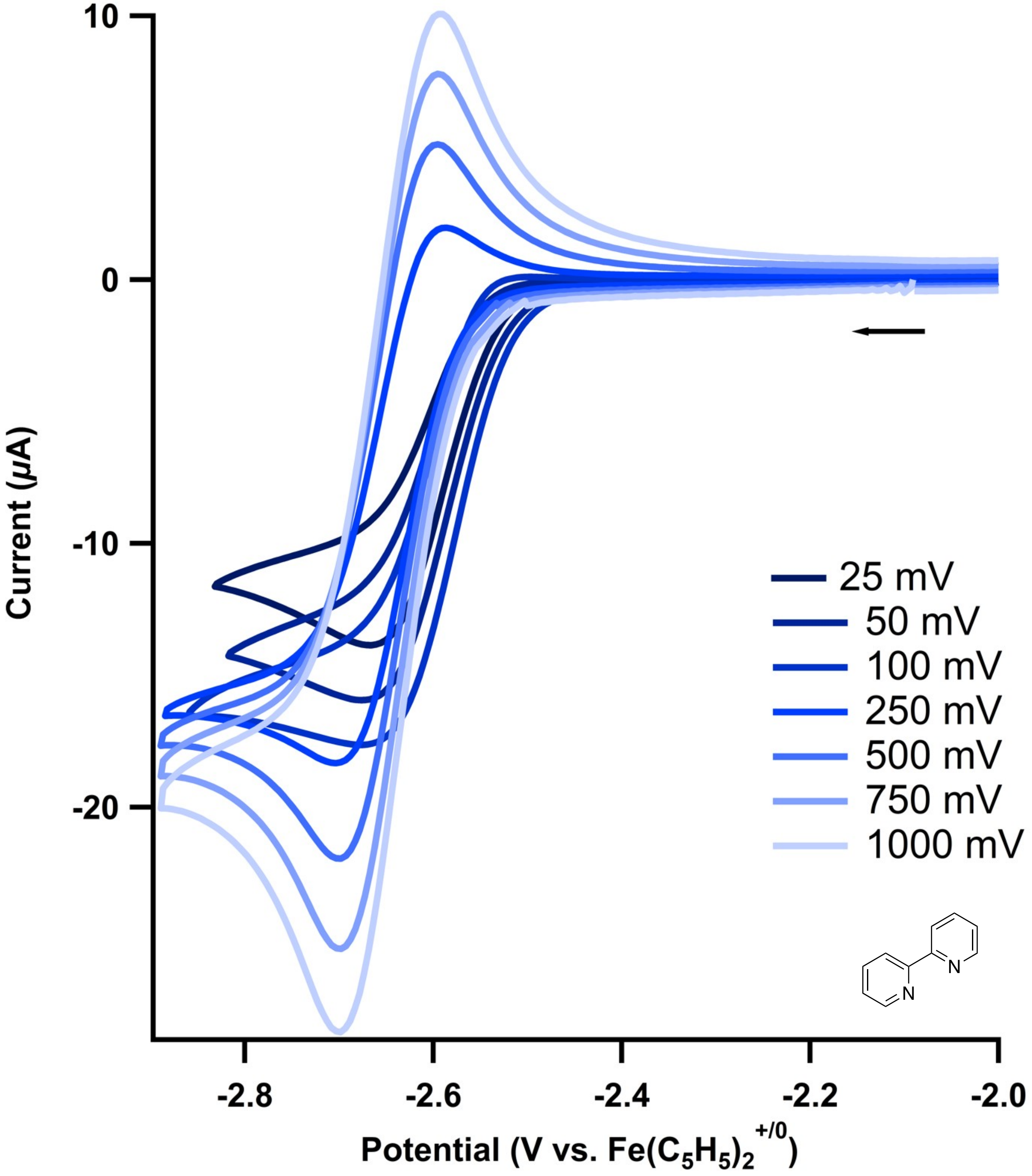

Figure S2. Scan rate dependent CV of 2,2'-bipyridine (2 mM) in MeCN using $TBAPF_6$ (0.2 M) as the supporting electrolyte. The bpy/bpy$^{\bullet-}$ feature is found to be reversible at scan rates of 500 mV/s and above.

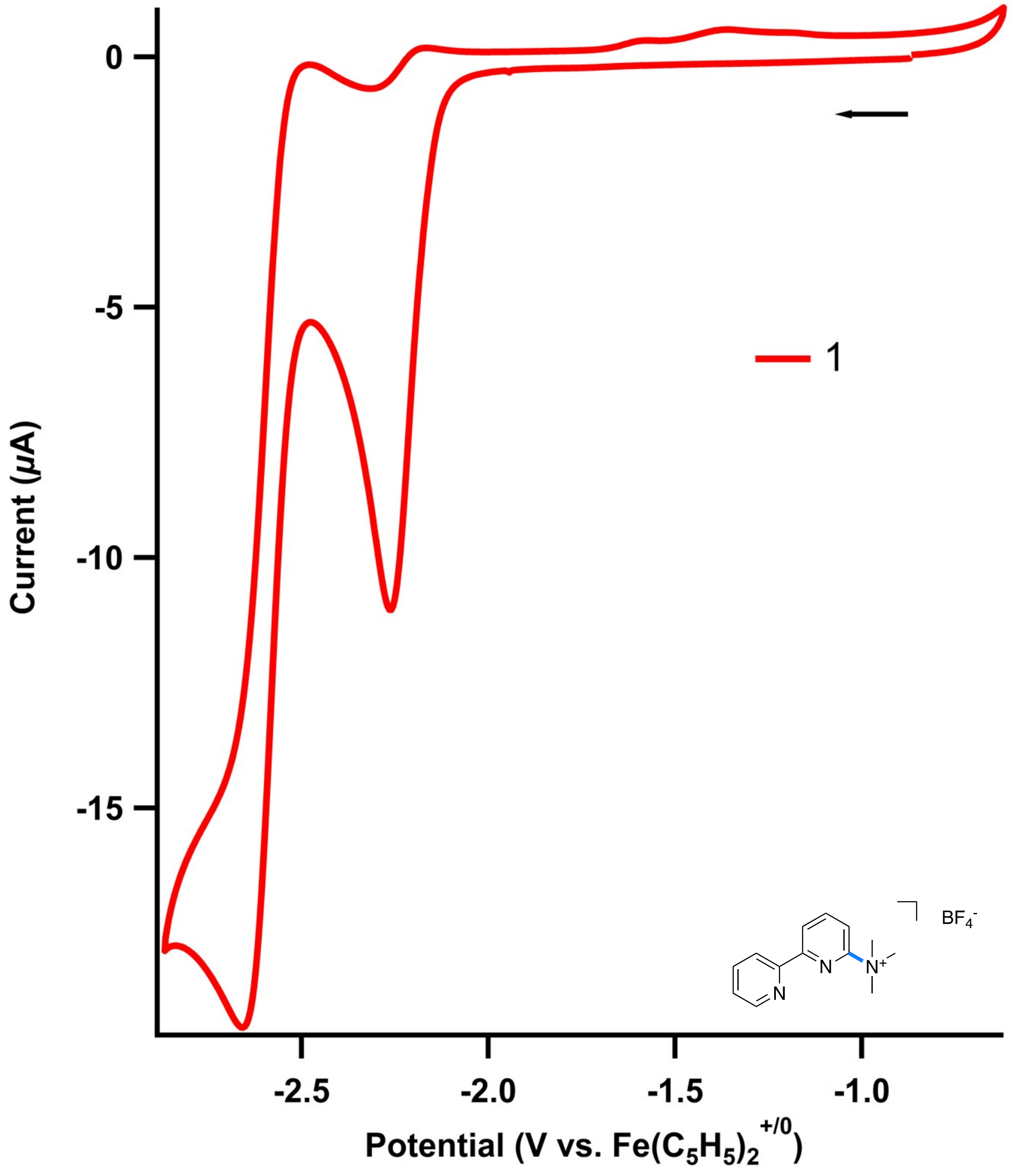

Figure S3. CV of **1** (2 mM) in MeCN using $TBAPF_6$ (0.2 M) as the supporting electrolyte. Scan rate = 100 mV/s

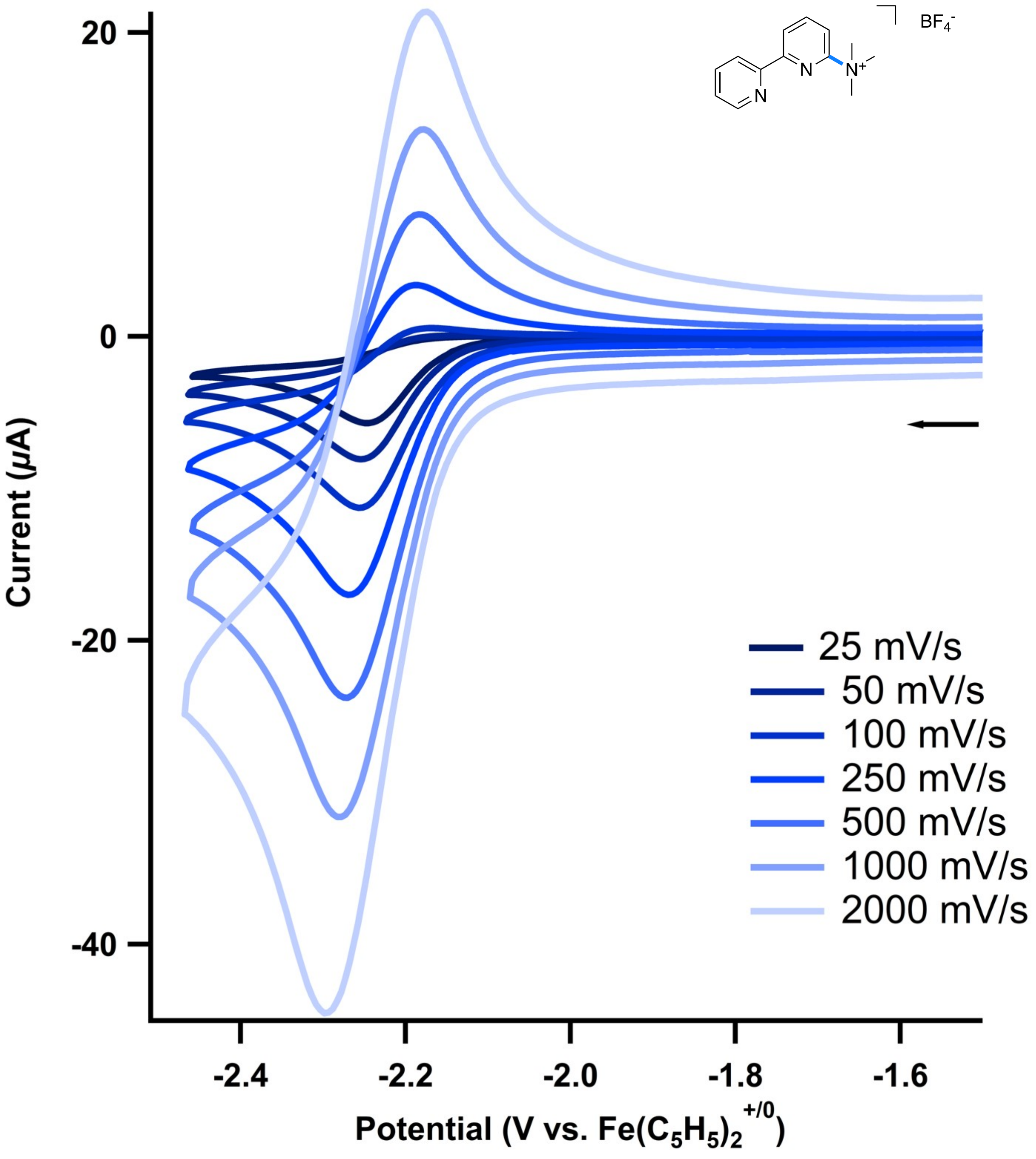

Figure S4. Scan rate dependent CV of the first redox feature of **1** (2 mM) in MeCN using $TBAPF_6$ (0.2 M) as the supporting electrolyte. This feature is found to be reversible at scan rates of 500 mV/s and above.

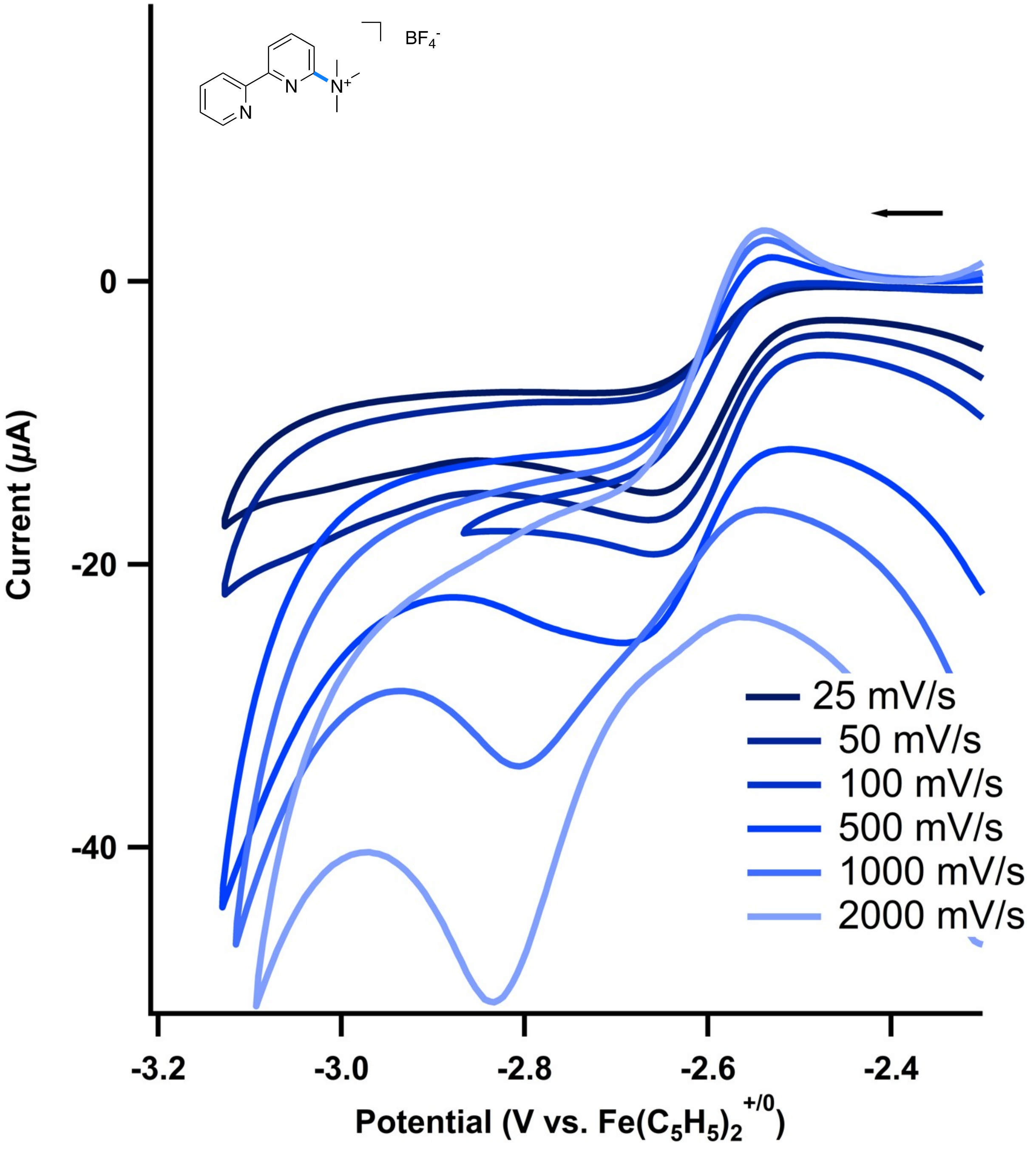

Figure S5. Scan rate dependent CV of the second redox feature of **1** (2 mM) in MeCN using $TBAPF_6$ (0.2 M) as the supporting electrolyte. This feature is found to be quasireversible.

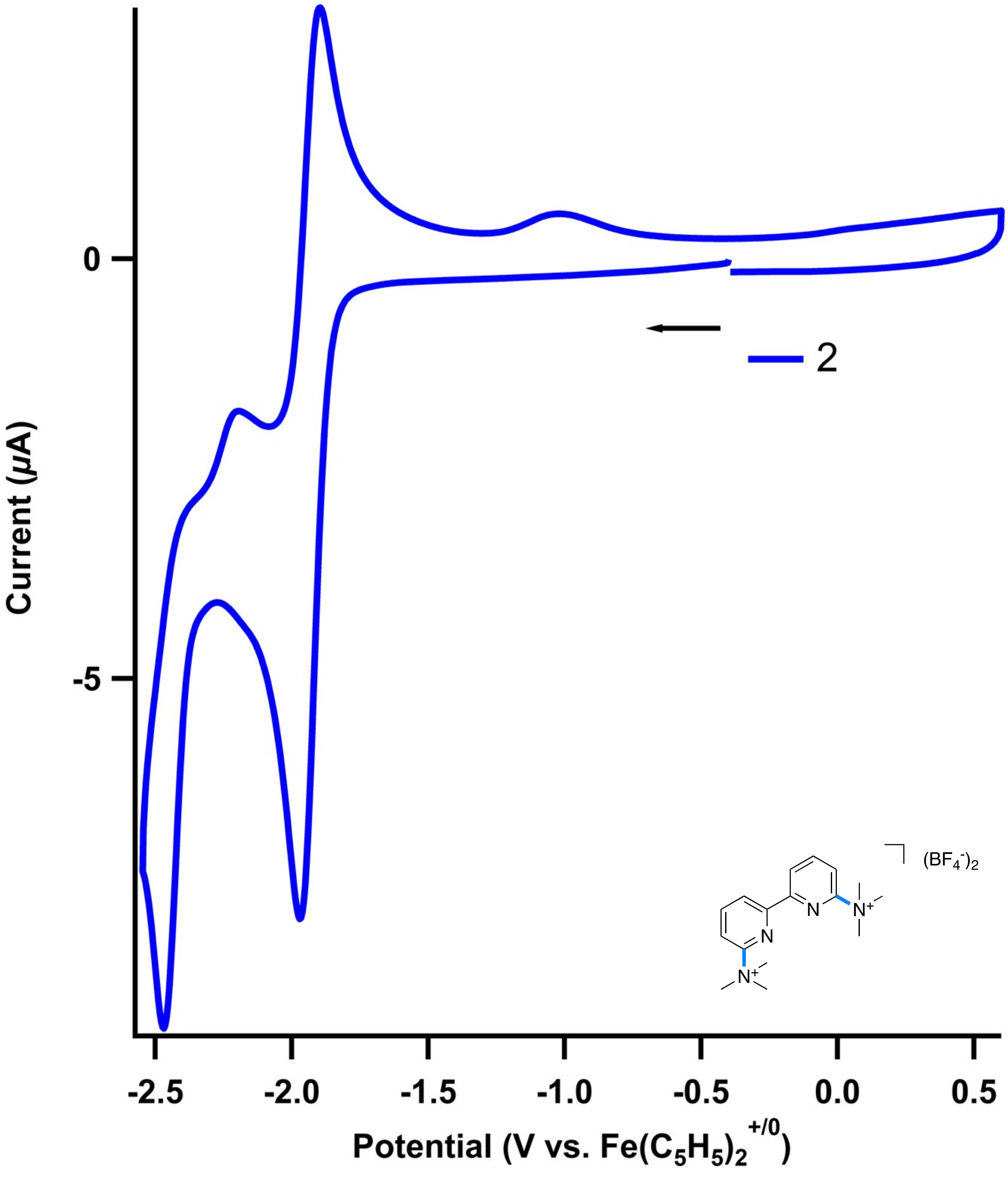

Figure S6. CV of **2** (2 mM) in MeCN using $TBAPF_6$ (0.2 M) as the supporting electrolyte. Scan rate = 100 mV/s

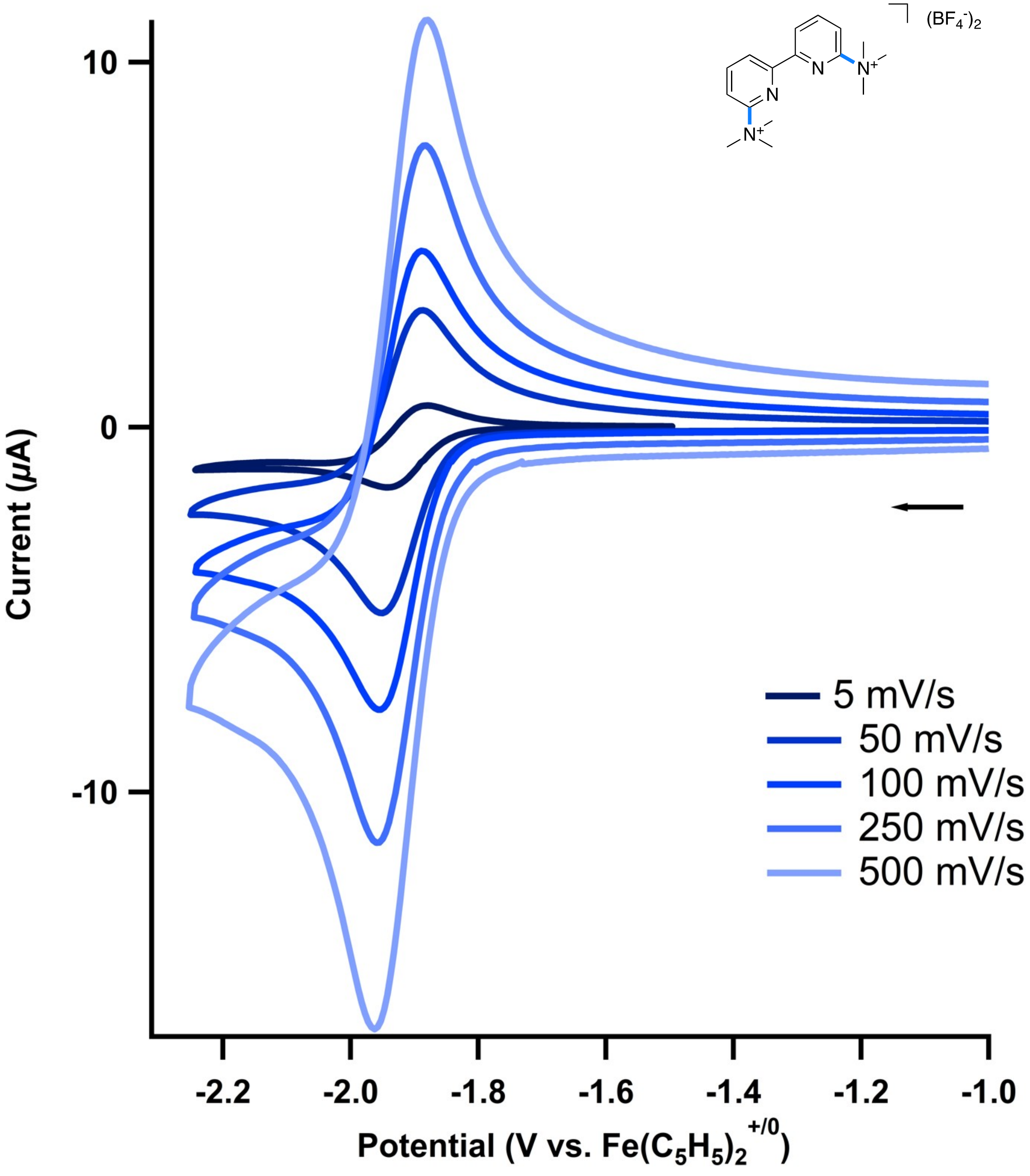

Figure S7. Scan rate dependent CV of the first redox feature of **2** (2 mM) in MeCN using $TBAPF_6$ (0.2 M) as the supporting electrolyte. This feature is found to be reversible at scan rates of 500 mV/s and above.

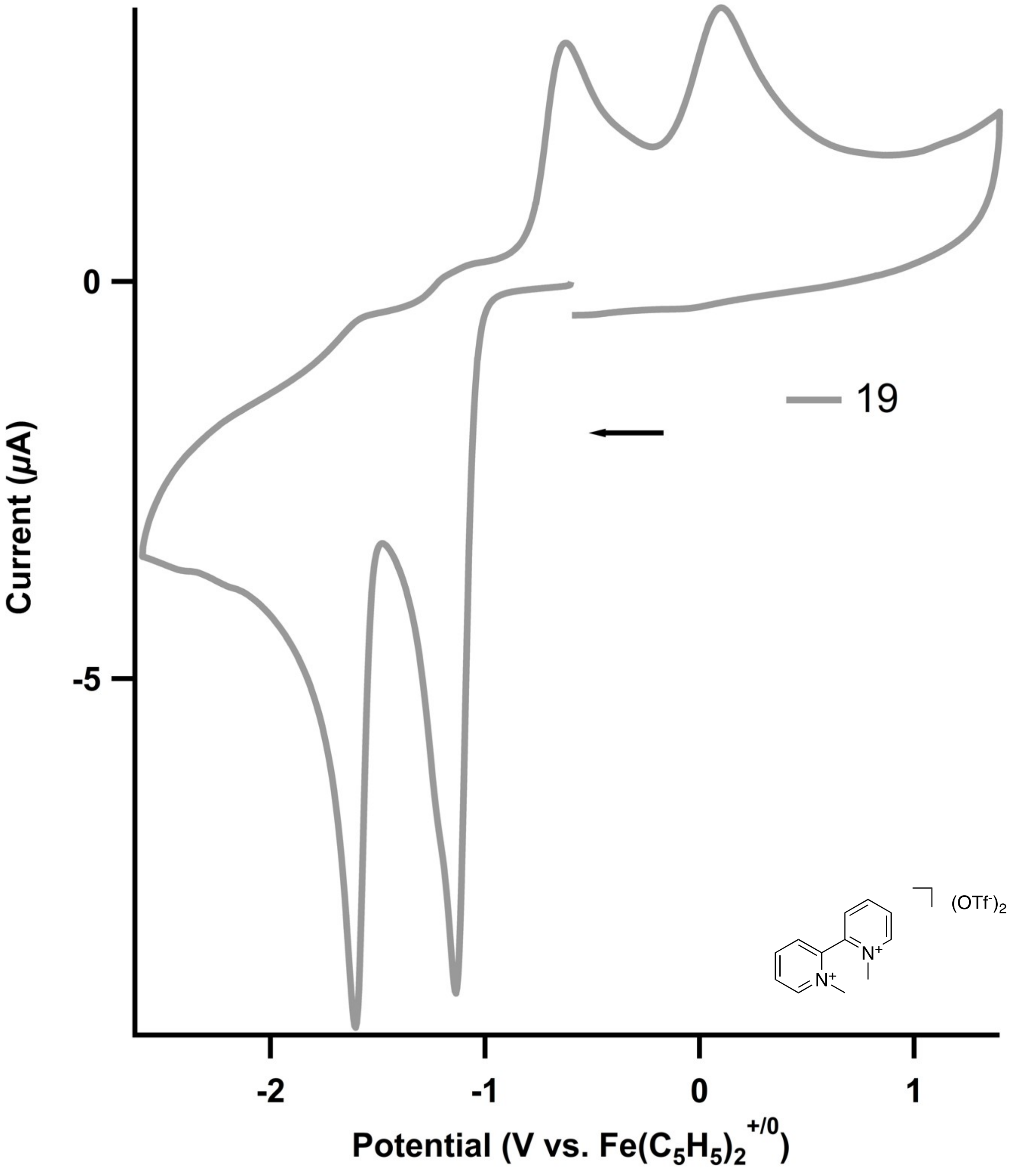

Figure S8. CV of **19** (2 mM) in MeCN using $TBAPF_6$ (0.2 M) as the supporting electrolyte. Scan rate = 100 mV/s

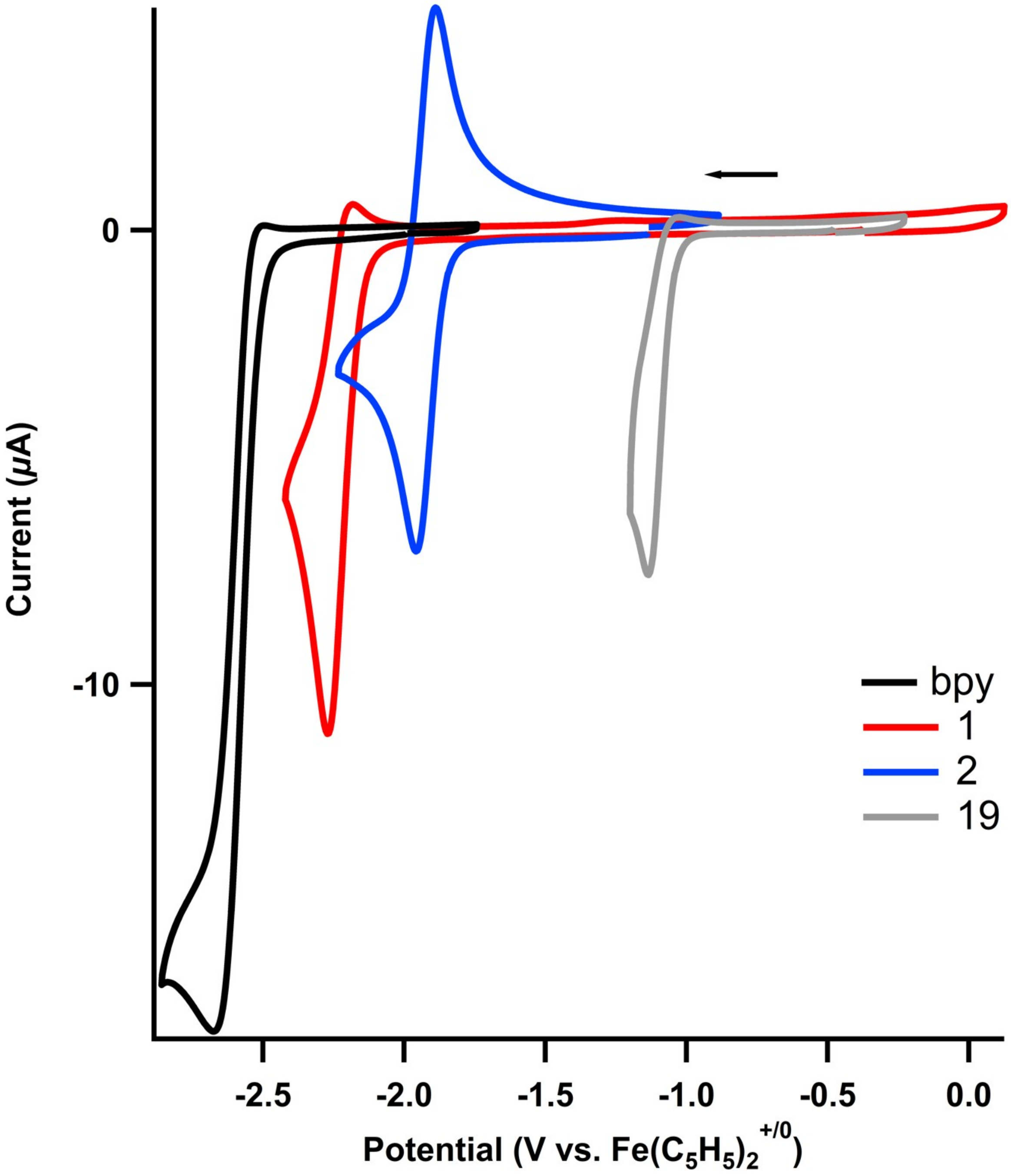

Figure S9. CV of 2,2'-bipyridine (black trace), **1** (red trace), **2** (blue trace), and **19** (grey trace), all 2 mM in MeCN with $TBAPF_6$ (0.2 M) as the supporting electrolyte. Scan rate = 100 mV/s

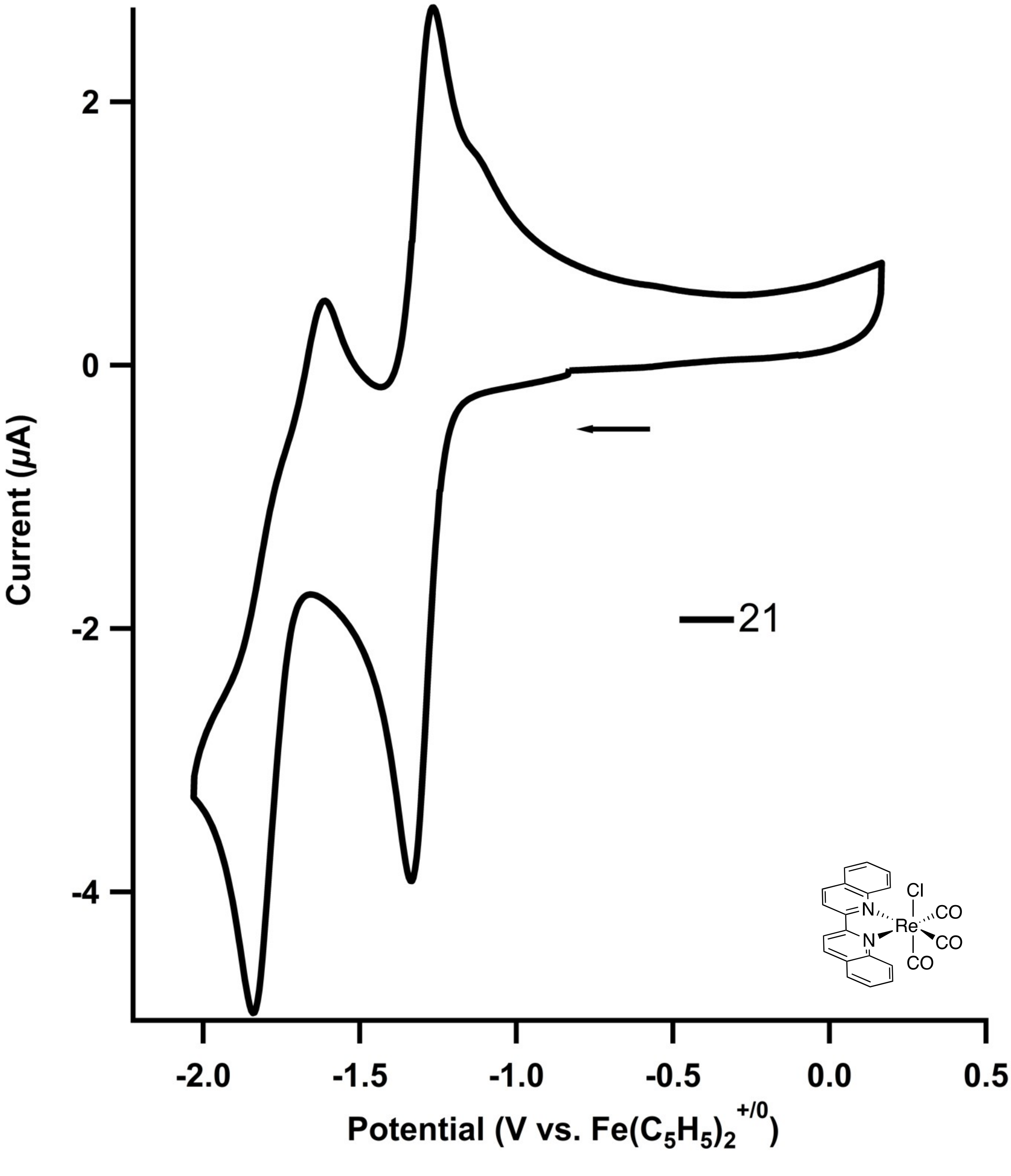

Figure S10. CV of **21** (2 mM) in DMF using $TBAPF_6$ (0.2 M) as the supporting electrolyte. Scan rate = 100 mV/s

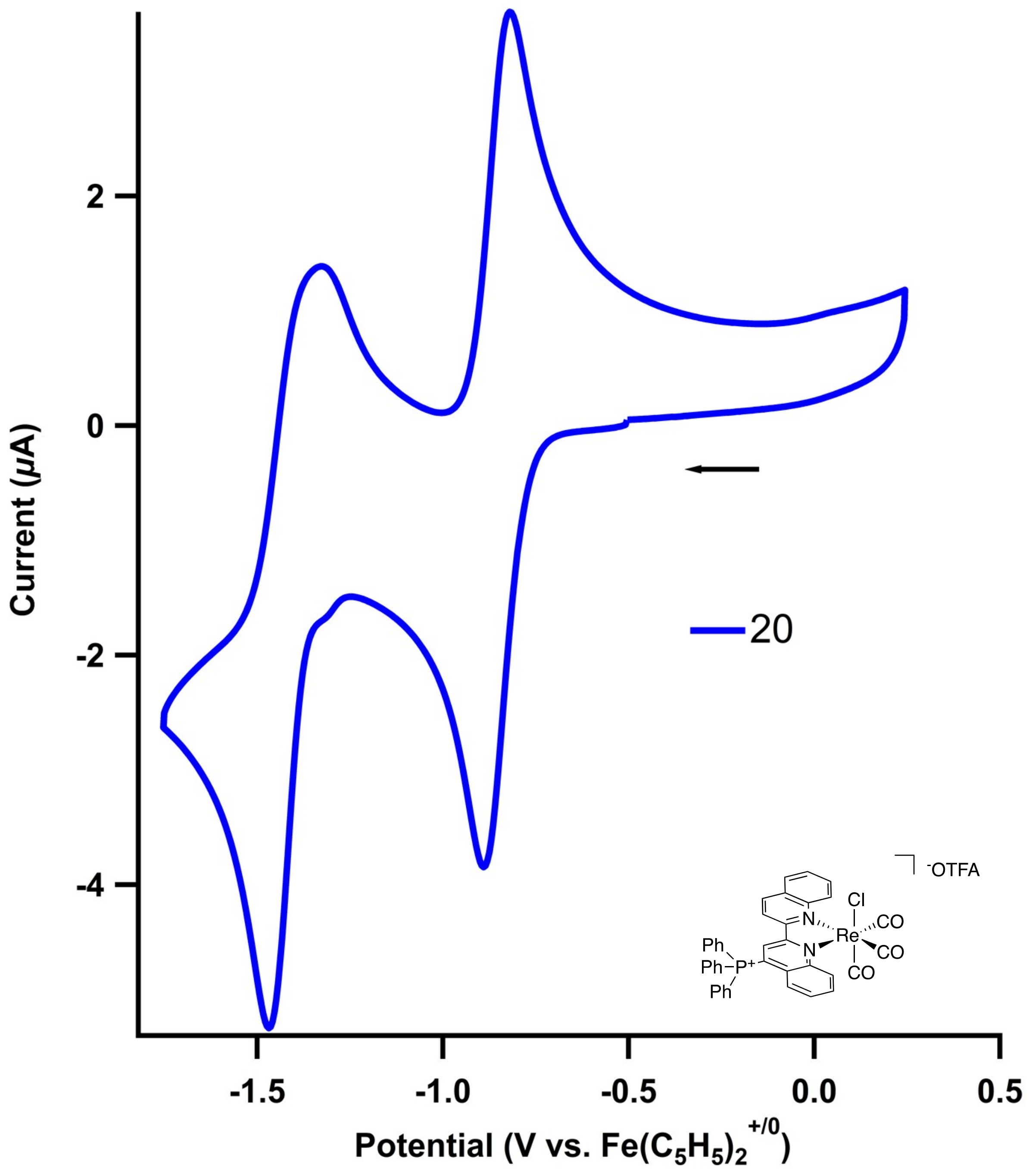

Figure S11. CV of **20** (2 mM) in DMF using $TBAPF_6$ (0.2 M) as the supporting electrolyte. Scan rate = 100 mV/s

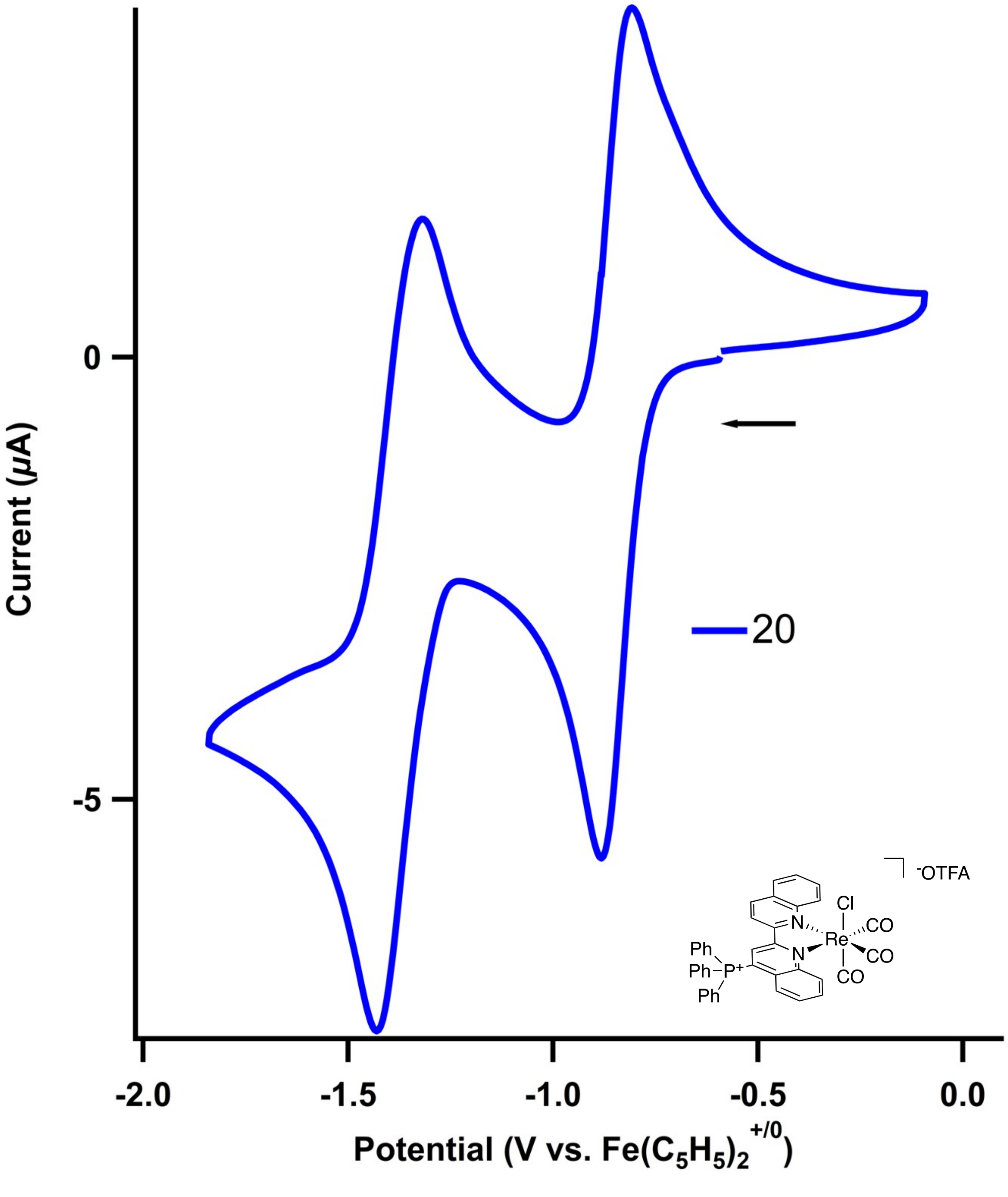

Figure S12. CV of **20** (2 mM) in MeCN using $TBAPF_6$ (0.2 M) as the supporting electrolyte. Scan rate = 100 mV/s

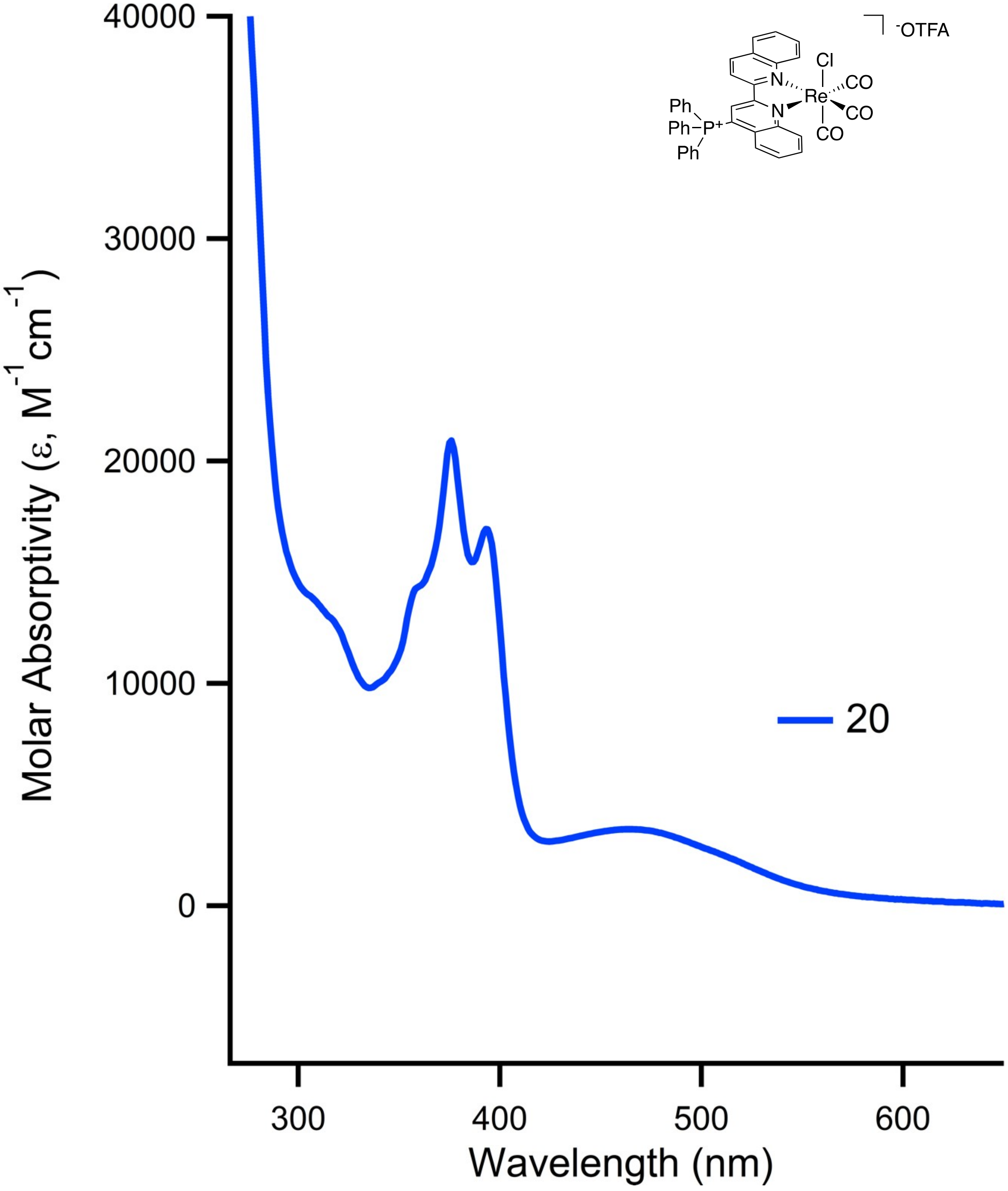

Figure S13. UV–Vis spectra of **20** in DMF

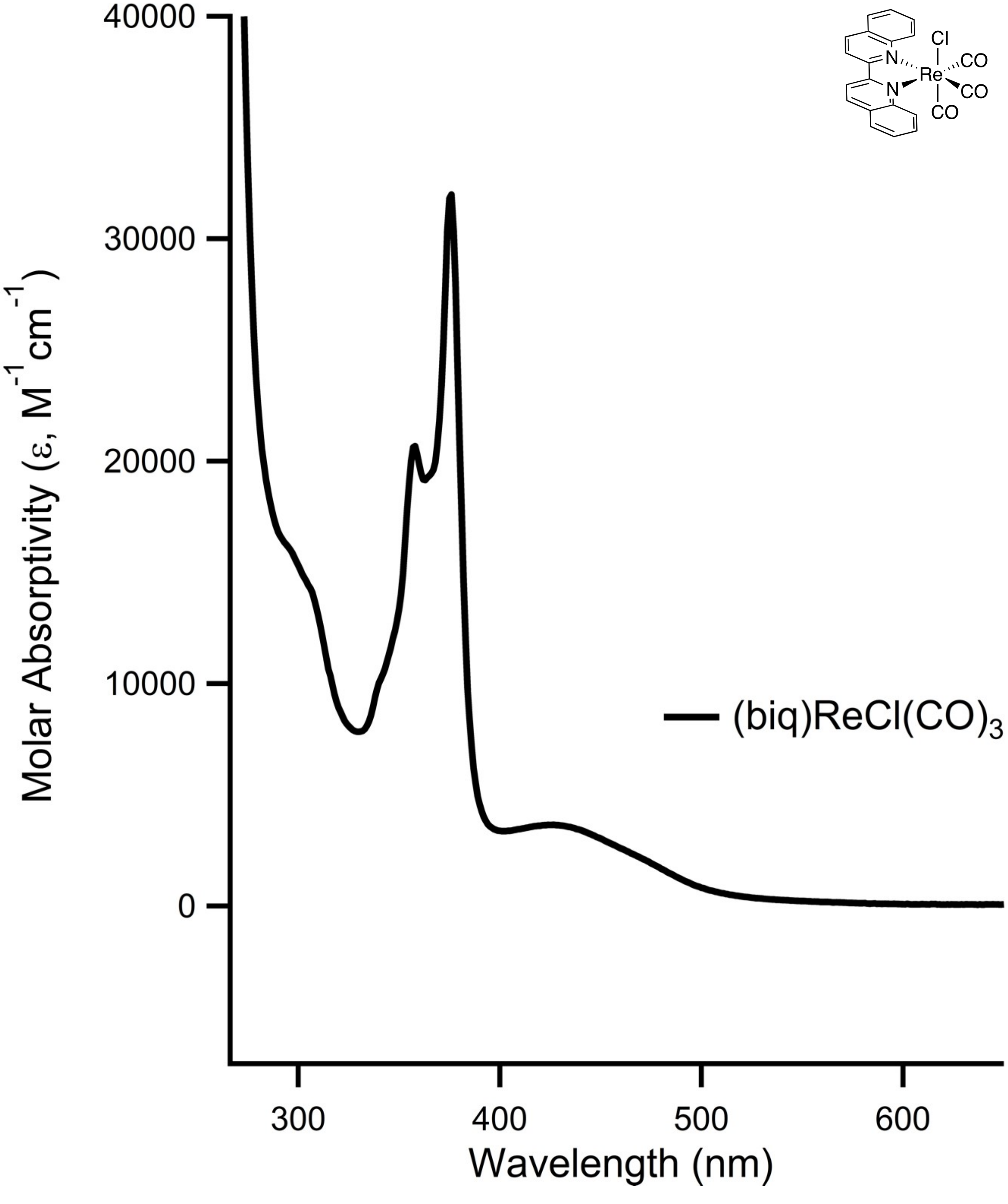

Figure S14. UV–Vis spectra of **21** in DMF

VI. References

1. Fulmer, G. R.; Miller, A. J. M.; Sherden, M. H.; Gottlieb, H. E.; Nudelman, A.; Stoltz, B. M.; Bercaw, J. E.; Goldberg, K. I. NMR chemical shifts of trace impurities: common laboratory solvents, organics, and gases in deuterated solvents relevant to the organometallic chemist. *Organometallics* **2010**, *29*, 2176–2179.

2. Zalas, M.; Gierczyk, B.; Klein, M.; Siuzdak, K.; Pędziński, T.; Łuczak, T. Synthesis of a novel dinuclear ruthenium polypyridine dye for dye-sensitized solar cells application. *Polyhedron* **2014**, *67*, 381–387.

3. Kajiwara, R.; Hirano, K.; Miura, M. Copper-mediated Regioselective C–H Cyanation of Phenols with Assistance of Bipyridine-type Bidentate Auxiliary. *Chem. Lett.* **2021**, *50*, 1814–1817.

4. Kim, J.-J.; Choi, H.; Paek, S.; Kim, C.; Lim, K.; Ju, M.-J.; Kang, H., S.; Kang, M.-S.; Ko, J. A New Class of Cyclometalated Ruthenium Sensitizers of the Type ĈN̂N for Efficient Dye-Sensitized Solar Cells. *Inorg. Chem.* **2011**, *50*, 11340–11347.

5. Cook, M. J.; Lewis, A. P.; McAuliffe, G. S. G.; Skarda, V.; Thomson, A. J.; Glasper, J. L.; Robbins, D. J. Luminescent metal complexes. Part 1. Tris-chelates of substituted 2,2'-bipyridyls with ruthenium (II) as dye for luminescent solar collectors. *J. Chem. Soc., Perkins Trans. 2* **1984**, 1293–1301.

6. Klenner, M. A.; Pascali, G.; Zhang, B.; Sia, T. R.; Spare, L. K.; Krause-Heuer, A. M.; Aldrich-Wright, J. R.; Greguric, I.; Guastella, A. J.; Massi, M.; Fraser, B. H. A Fluorine-18 Radiolabeling Method Enabled by Rhenium(I) Complexation Circumvents the Requirement of Anhydrous Conditions. *Chem. Eur. J.* **2017**, *23*, 6499–6503.

7. Malkov, A. V.; Hand, J. B.; Kočovský, P. A long-range chiral relay *via* tertiary amide group in asymmetric catalysis: new amino acid-derived N,P-ligands for copper-catalysed conjugate addition. *Chem. Commun.* **2003**, 1948–1949.

8. Zhang, Y.; Zhang, S.; Xu, G.; Li, M.; Tang, C.; Fan, W. Cu-Catalyzed carbamoylation *versus* amination of quinoline *N*-oxide with formamides. *Org. Biomol. Chem.* **2019**, *17*, 309–314.

9. Thummel, R. P.; Jahng, Y. N-Oxides of 2,2':6',2"-terpyridine. *J. Org. Chem.* **1985**, *50*, 3635–3636.

10. Muramatsu, W.; Hattori, T.; Yamamoto, H. Substrate-Directed Lewis-Acid Catalysis for Peptide Synthesis. *J. Am. Chem. Soc.* **2019**, *141*, 12288–12295.

11. Ward, R. S.; Branciard, D.; Dignan, R. A.; Pritchard, M. C. Synthesis of C_2 Symmetric 2,2'-Bipyridyl Imidazolidinone and Oxazaborolidine Derivatives. *Heterocycles* **2002**, *56*, 157–170.

12. Zavozin, A. G.; Ignat'ev, N. V.; Schulte, M.; Zlotin, S. G. Synthesis of novel tridentate pyrazole–bipyridine ligands for Co-complexes as redox-couples in dye-sensitized solar cells. *Tetrahedron* **2015**, *71*, 8551–8556.

13. Davies, J.; Janssen-Müller, D.; Zimin, D. P.; Day, C. S.; Yanagi, T.; Elfert, J.; Martin, R. Ni-Catalyzed Carboxylation of Aziridines en Route to β-Amino Acids. *J. Am. Chem. Soc.* **2021**, *143*, 4949–4954.

14. ten Brink, G.-J.; Arends, I. W. C. E.; Hoogenraad, M.; Verspui, G.; Sheldon, R. A. Catalytic Conversions in Water. Part 22: Electronic Effects in the (Diimine)palladium(II)-Catalysed Aerobic Oxidation of Alcohols. *Adv. Synth. Catal.* **2003**, *345*, 497–505.

15. Thummel, R. P.; Lefoulon, F. Polyaza cavity shaped molecules. 2. Annelated derivatives of 2,2'-biquinoline and the corresponding N-oxides. *J. Org. Chem.* **1985**, *50*, 666–670.

16. Lewis, F. W.; Harwood, L. M.; Hudson, M. J.; Distler, P.; John, J.; Stamberg, K.; Núñez, A.; Galán, H.; Espartero, A. G. Synthesis and Evaluation of Lipophilic BtBP Ligands for An/Ln

Separation in Nuclear Waste Treatment: The Effect of AlkylSubstitution on Extraction Properties and Implications for Ligand Design. *Eur. J. Org. Chem.* **2012**, *8*, 1509–1519.
17. Liu, W.; Li, Y.; Wang, Y.; Kuang, C. Pd-Catalyzed Oxidative CH/CH Direct Coupling of Heterocyclic *N*-Oxides. *Org. Lett.* **2013**, *15*, 4682–4685.
18. Coe, B. J.; Curati, N. R. M.; Fitzgerald, E. C.; Coles, S. J.; Horton, P. N.; Light, M. E. Hursthouse, M. B. Syntheses and Properties of Bimetallic Chromophore-Quencher Assemblies Containing Ruthenium(II) and Rhenium(I) Centers. *Organometallics*, **2007**, *26*, 2318–2329.

VII. Copies of NMR Spectra

4,4’-di-*tert*-butyl-2,2’-bipyridine *N*,*N*’-dioxide (S1)

^{1}H NMR (600 MHz, $CDCl_3$)

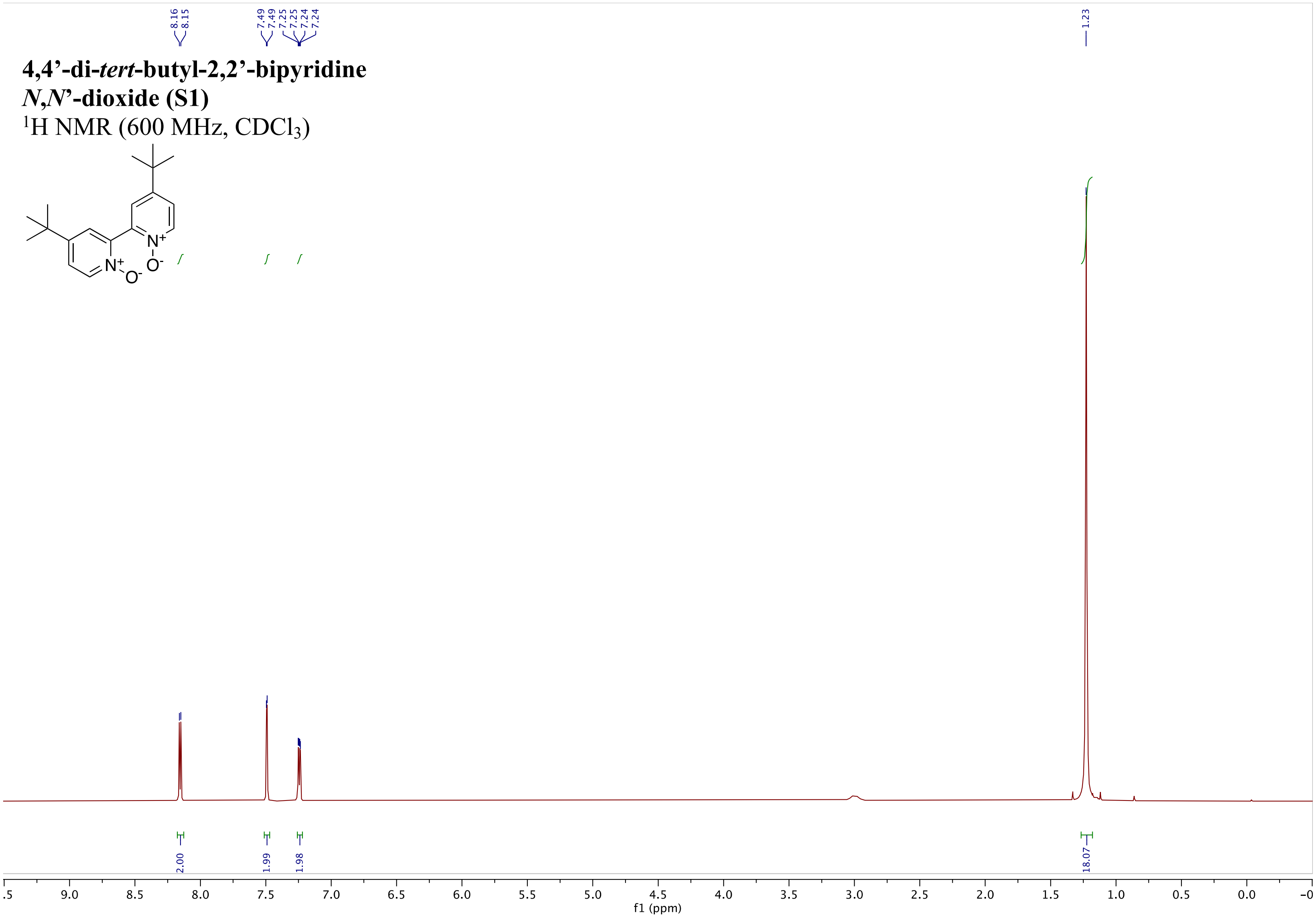

4,4'-di-*tert*-butyl-2,2'-bipyridine *N*,*N*'-dioxide (S1)

^{13}C NMR (151 MHz, CDCl$_3$)

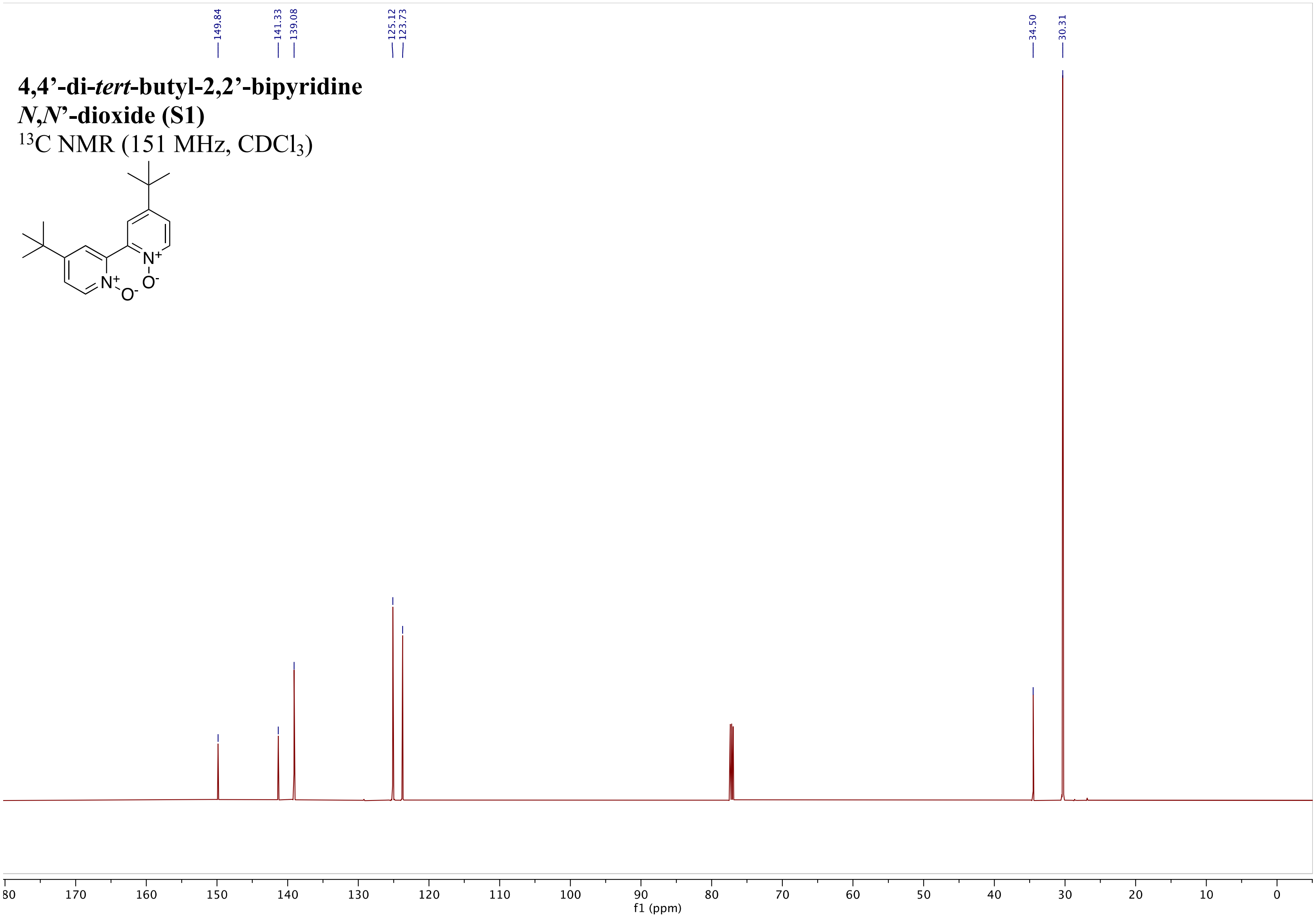

5,5'-dimethyl-2,2'-bipyridine *N*,*N*'-dioxide (S2)
^{1}H NMR (600 MHz, $CDCl_3$)

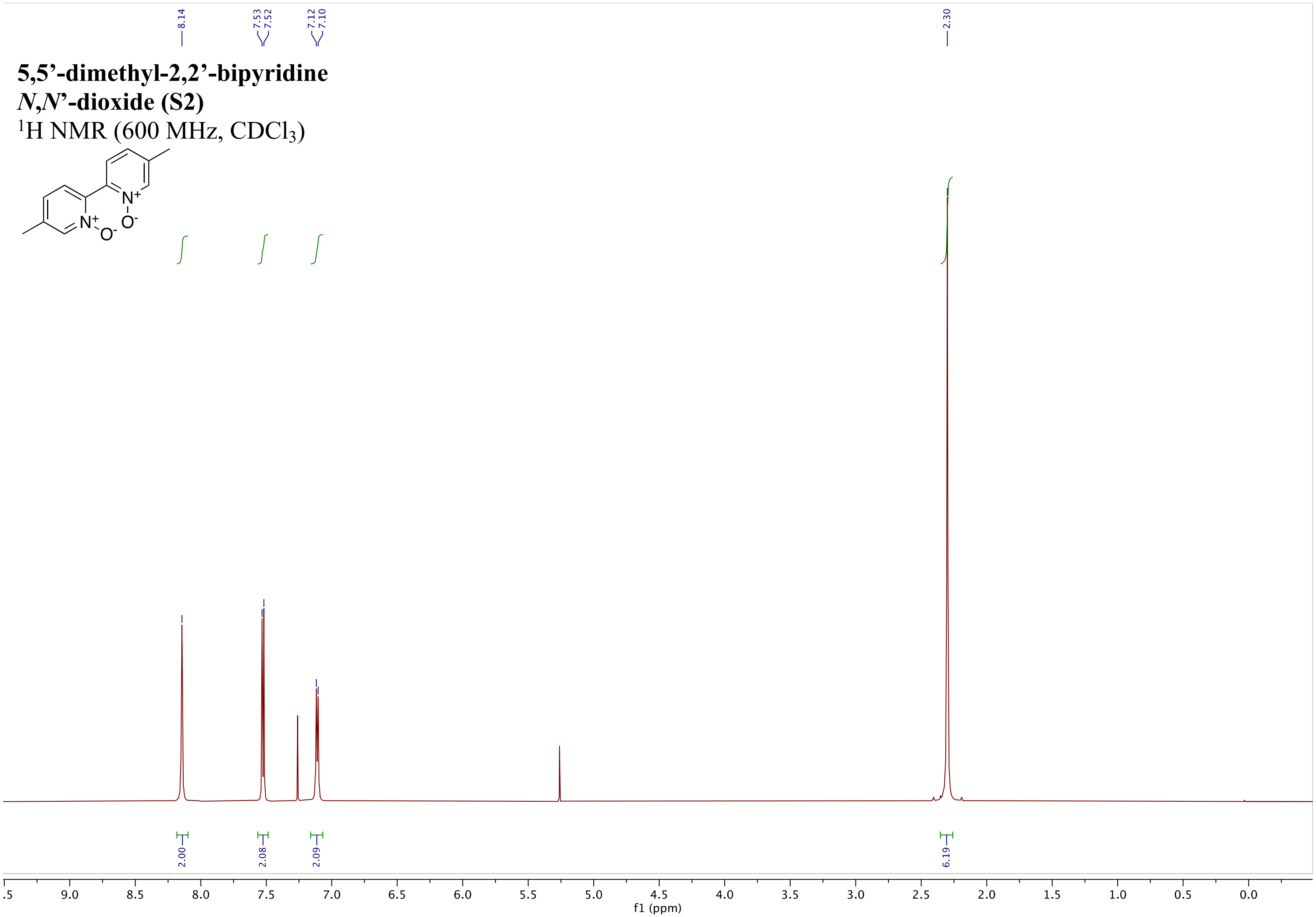

5,5'-dimethyl-2,2'-bipyridine *N*,*N*'-dioxide (S2)

^{13}C NMR (151 MHz, $CDCl_3$)

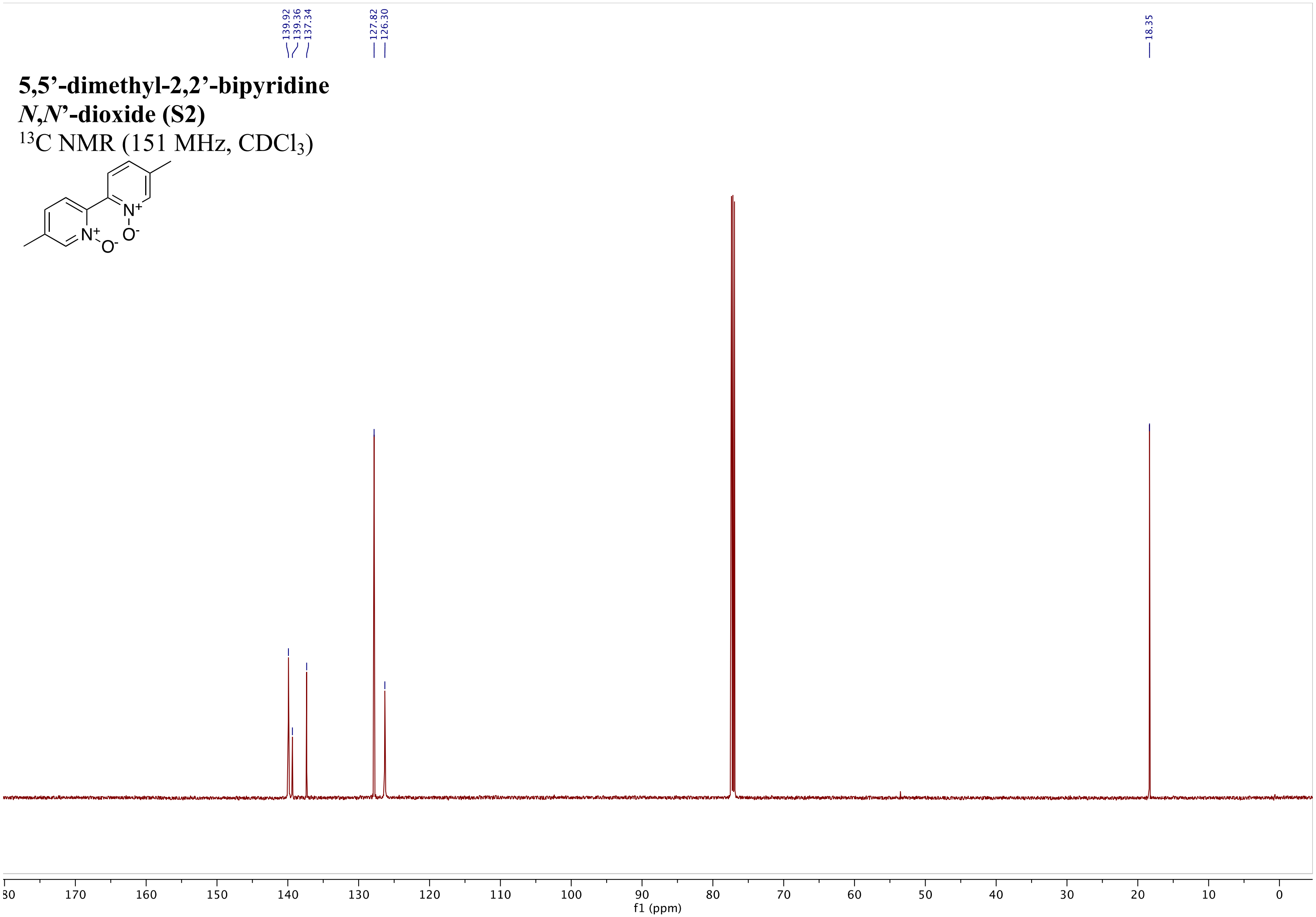

2,2':6',2":6",2"'-quaterpyridine
***N*,*N*"'-dioxide (S3)**
^{1}H NMR (600 MHz, $CDCl_3$)

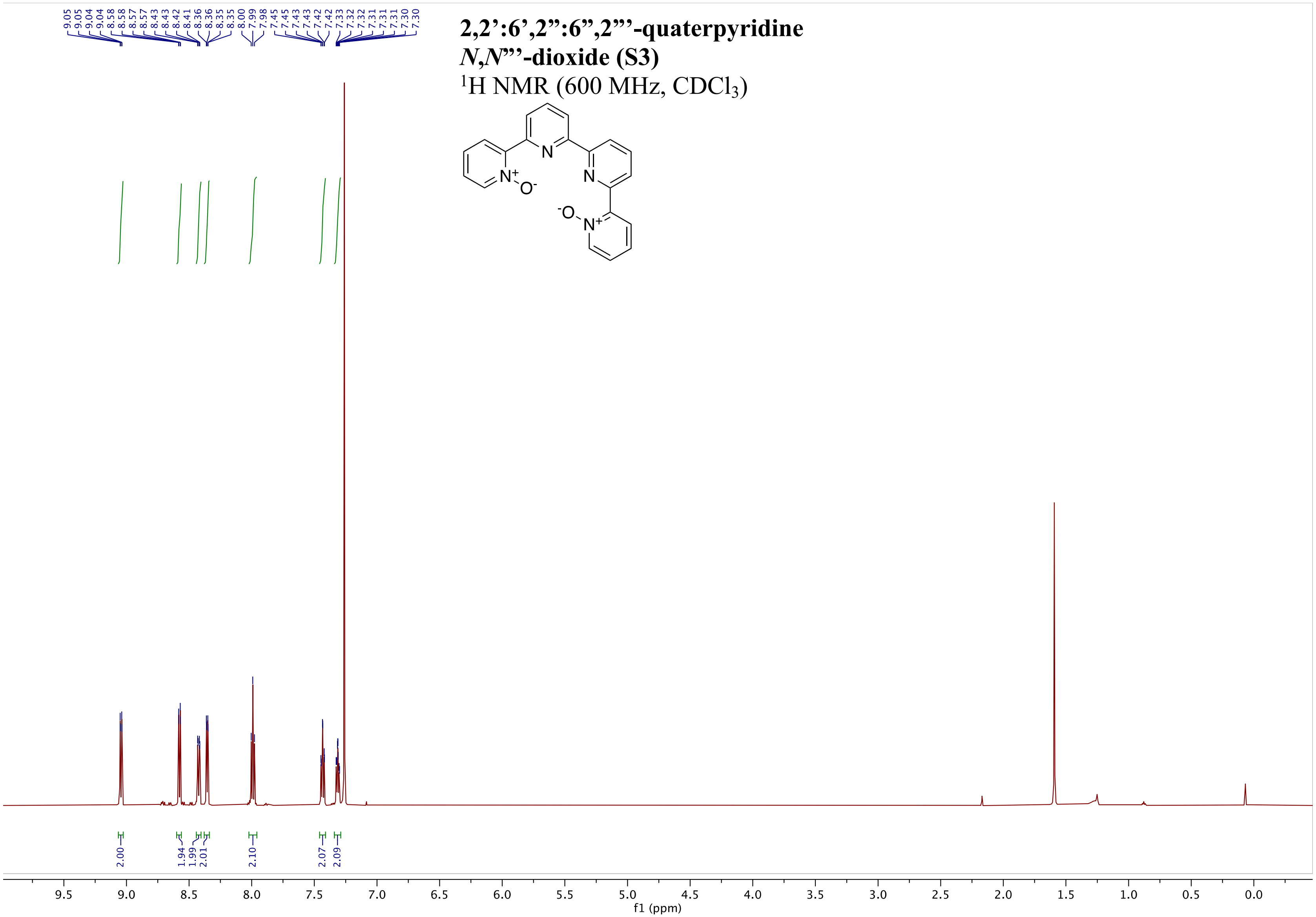

2,2’:6’,2”:6”,2”’-quaterpyridine
***N*,*N*”’-dioxide (S3)**
^{13}C NMR (151 MHz, $CDCl_3$)

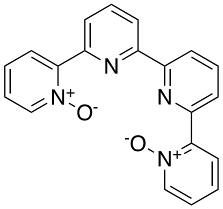

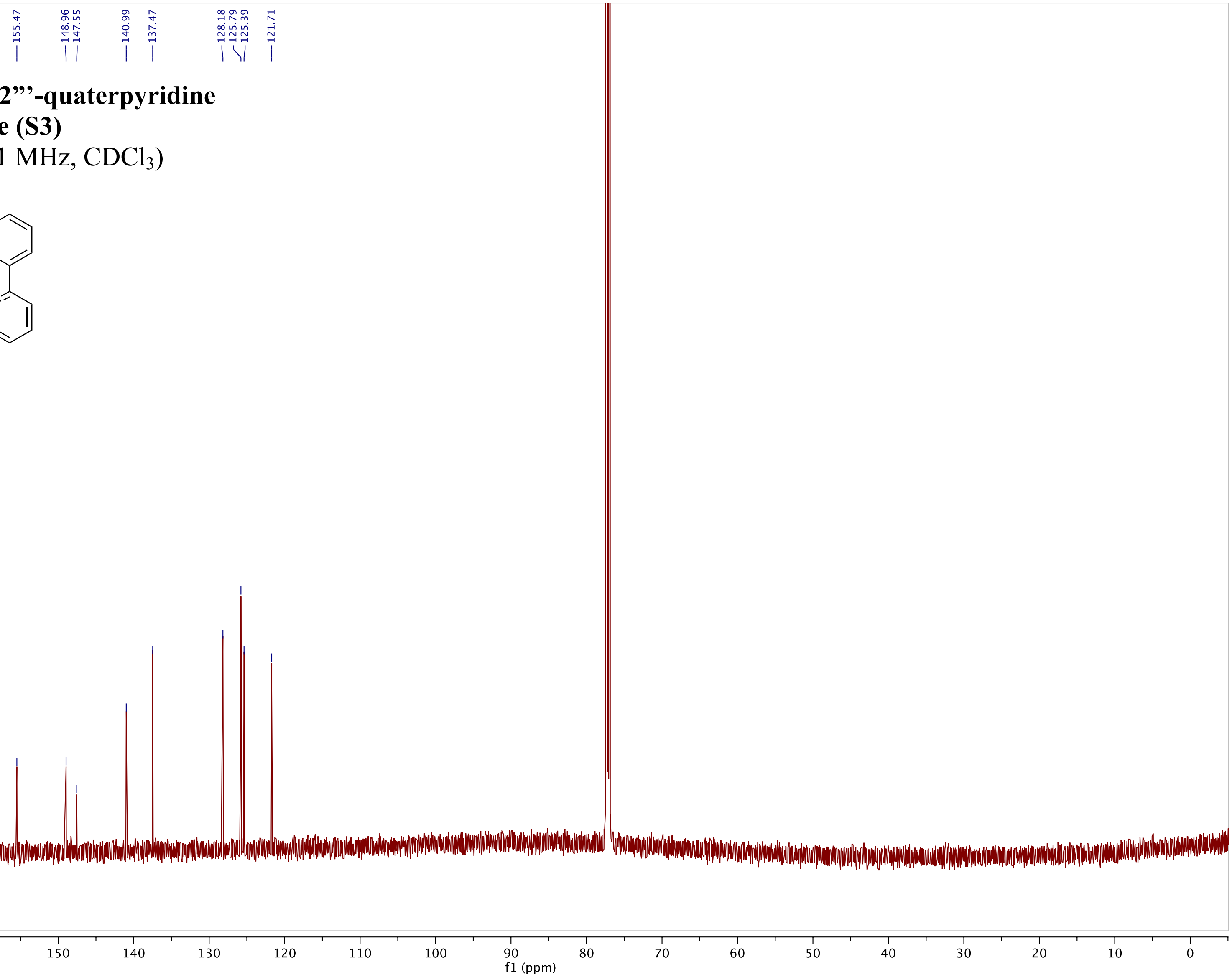

***N,N,N*-trimethyl-2,2'-bipyridin-6-aminium tetrafluoroborate (1)**

^{1}H NMR (600 MHz, CD_3CN)

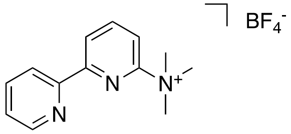

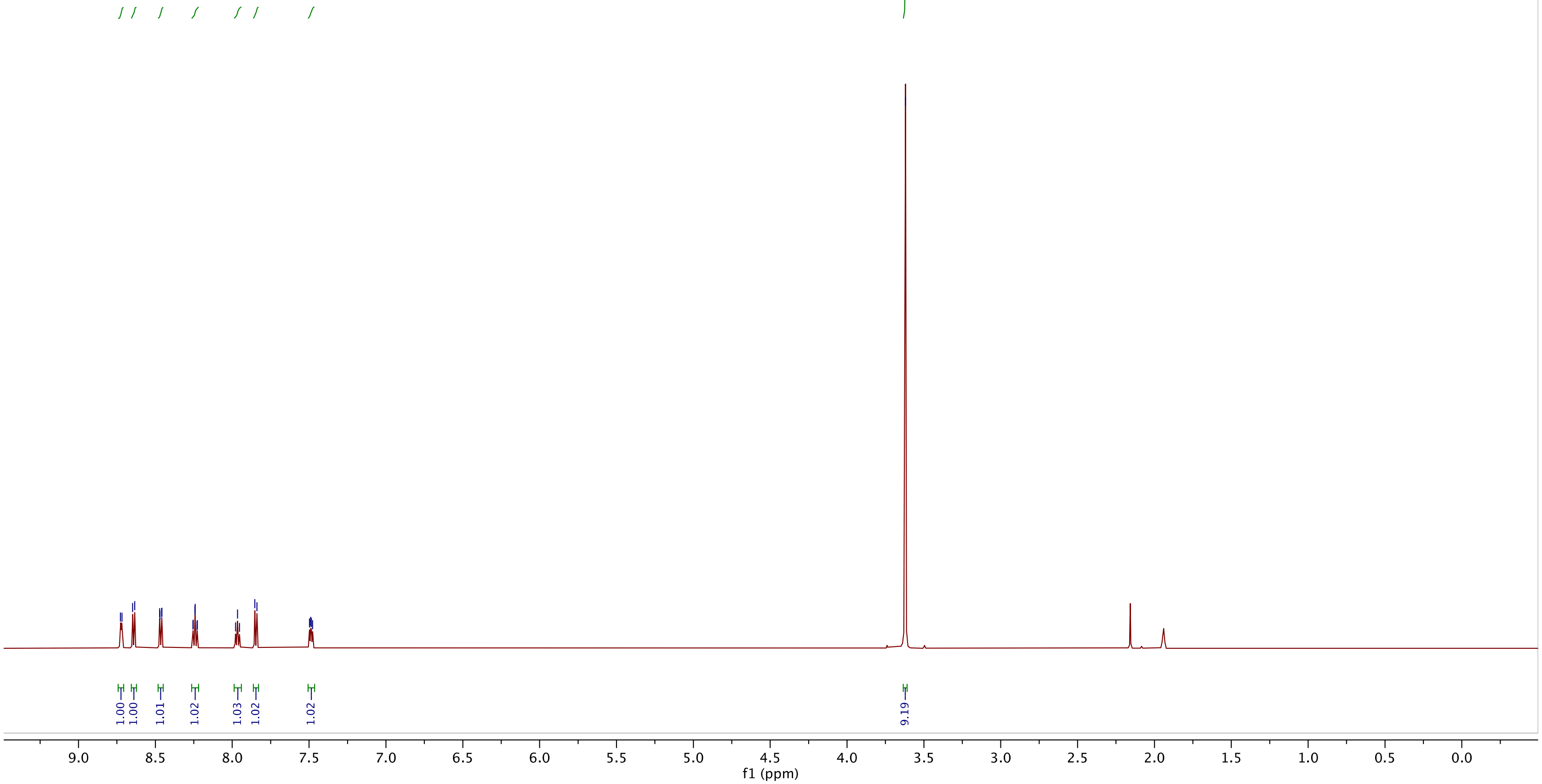

_N,N,N_-trimethyl-2,2’-bipyridin-6-aminium tetrafluoroborate (1)

^{13}C NMR (151 MHz, CD_3CN)

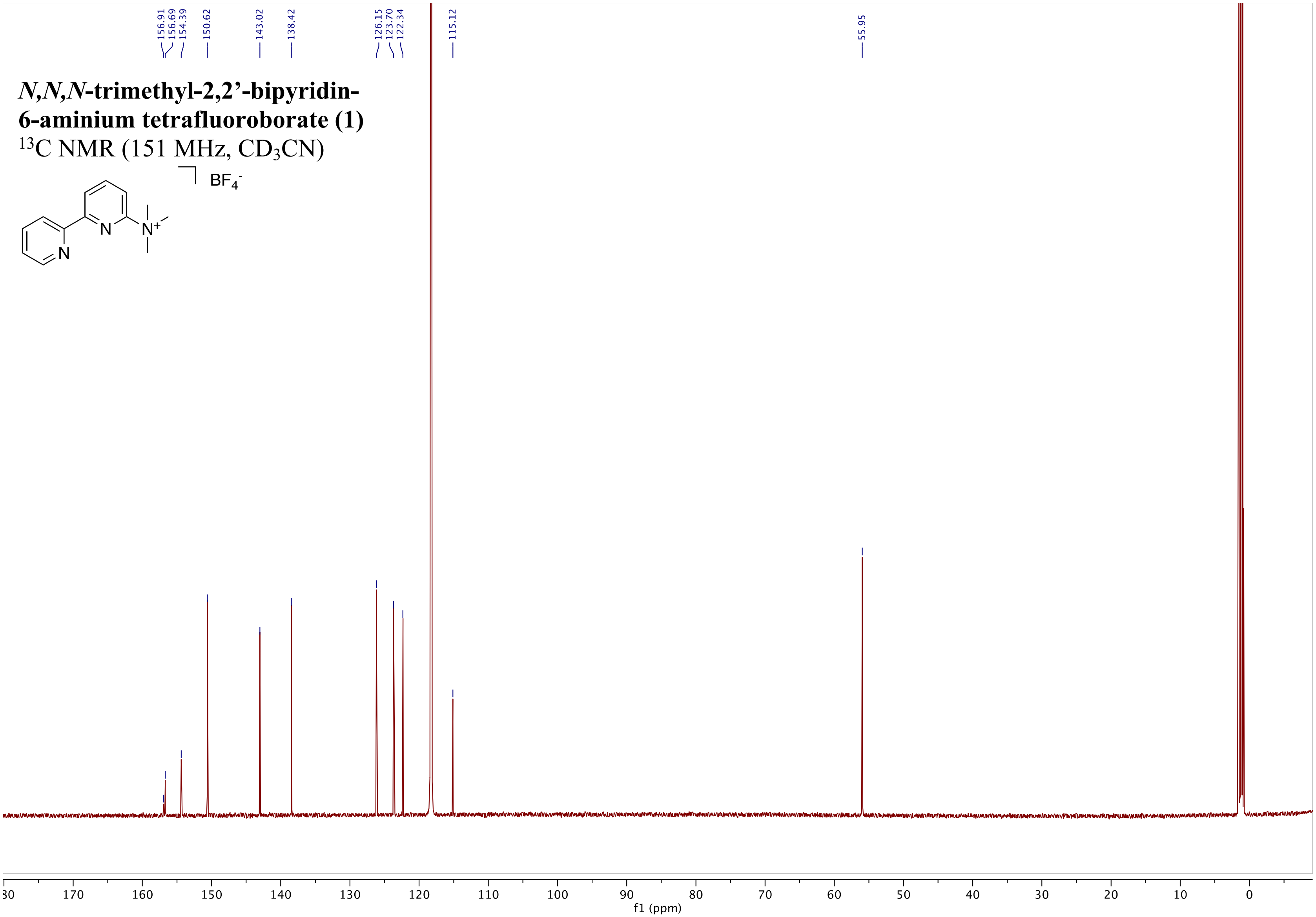

***N*,*N*,*N*-trimethyl-2,2’-bipyridin-6-aminium tetrafluoroborate (1)**

^{19}F NMR (565 MHz, CD_3CN)

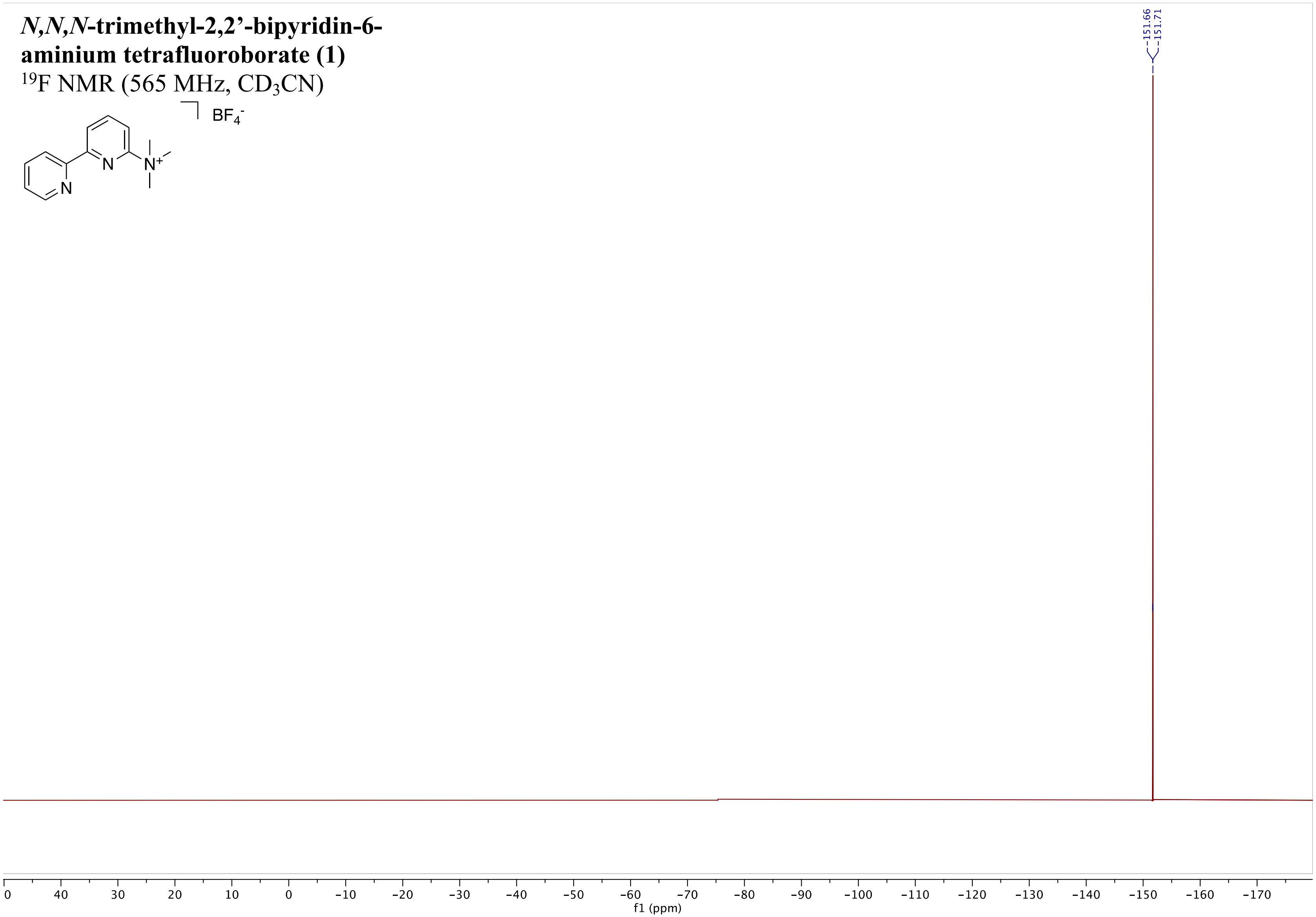

***N,N,N,N',N',N'*-hexamethyl-2,2'-bipyridin-6,6'-diaminium bis(tetrafluoroborate) (2)**

^{1}H NMR (600 MHz, CD_3CN)

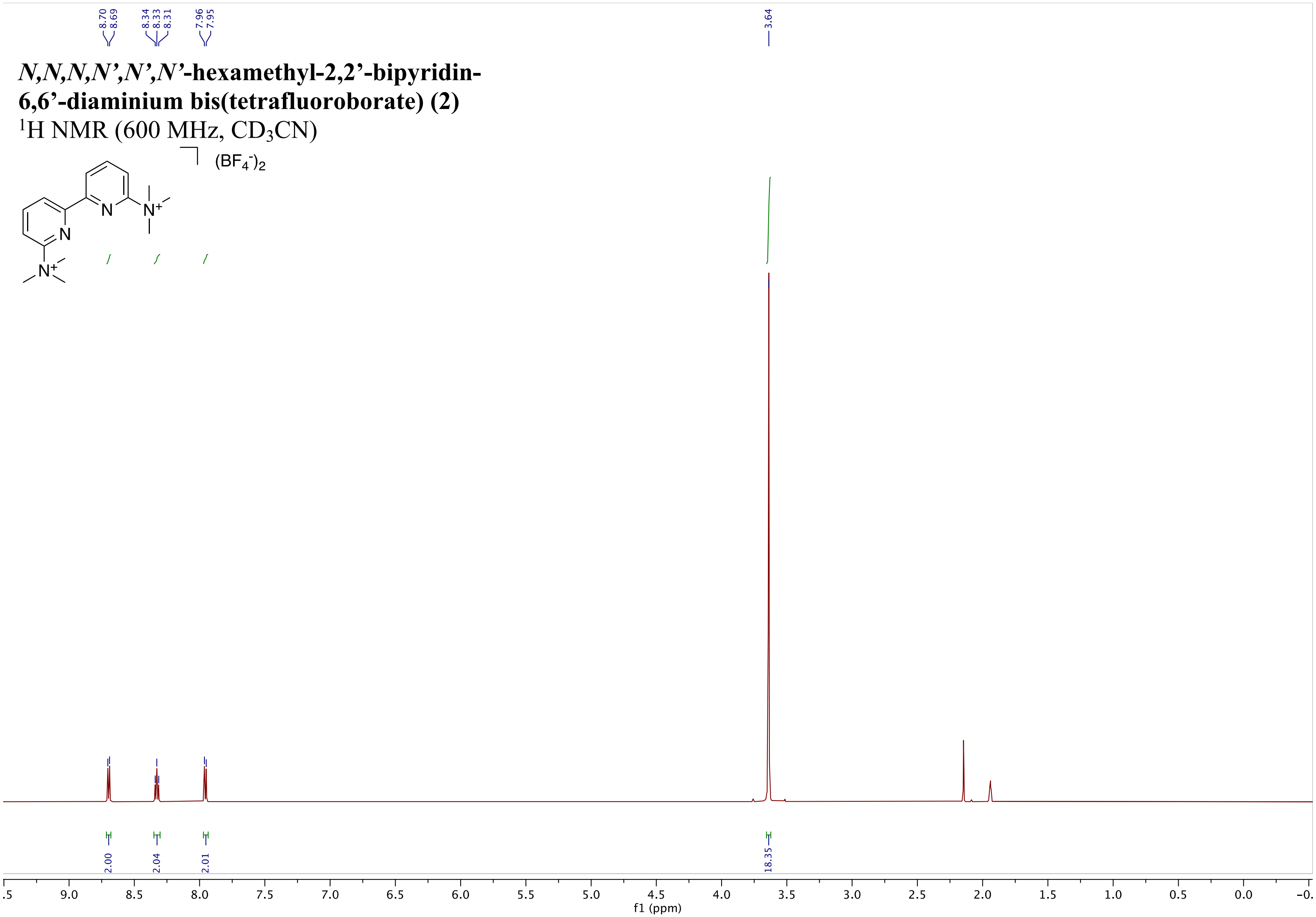

_N,N,N,N',N',N'_-hexamethyl-2,2'-bipyridin-6,6'-diaminium bis(tetrafluoroborate) (2)

^{13}C NMR (151 MHz, CD_3CN)

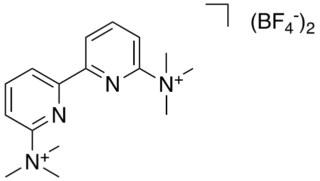

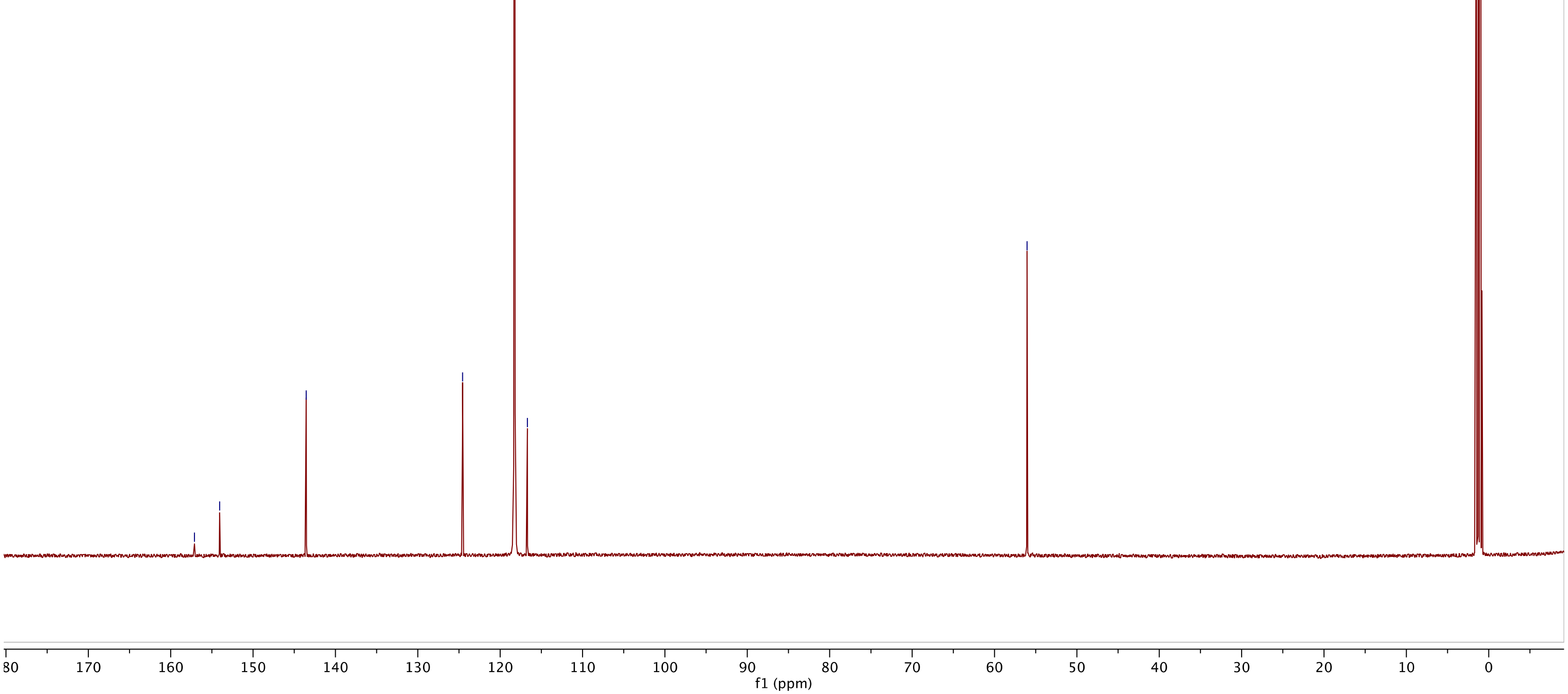

***N,N,N,N',N',N'*-hexamethyl-2,2'-bipyridin-6,6'-diaminium bis(tetrafluoroborate) (2)**

^{19}F NMR (565 MHz, CD_3CN)

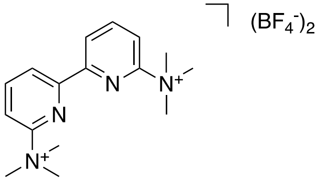

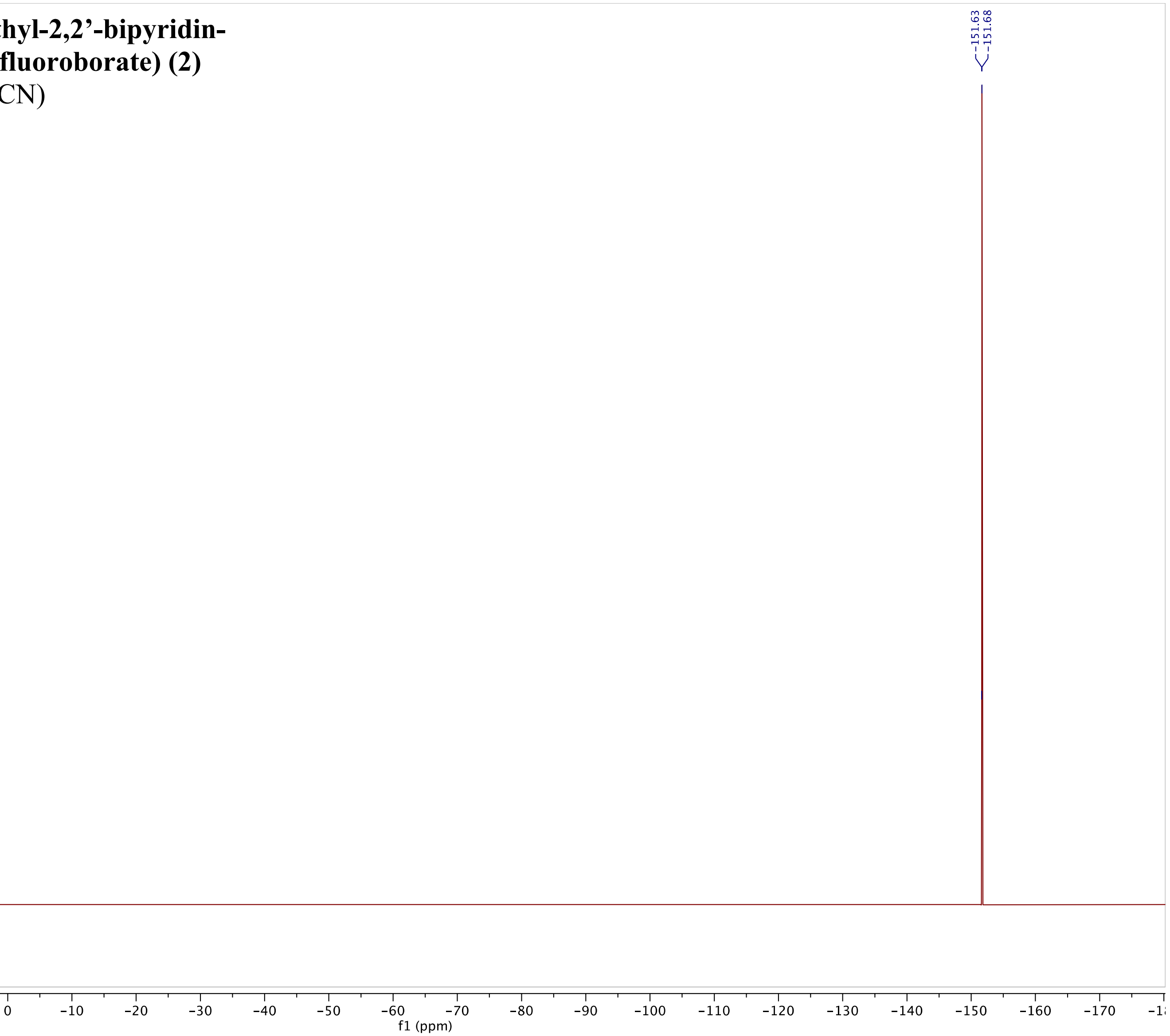

***N,N,N*-trimethyl-4,4'-di-*tert*-butyl-2,2'-bipyridin-6-aminium tetrafluoroborate (3)**

^{1}H NMR (600 MHz, CD_3CN)

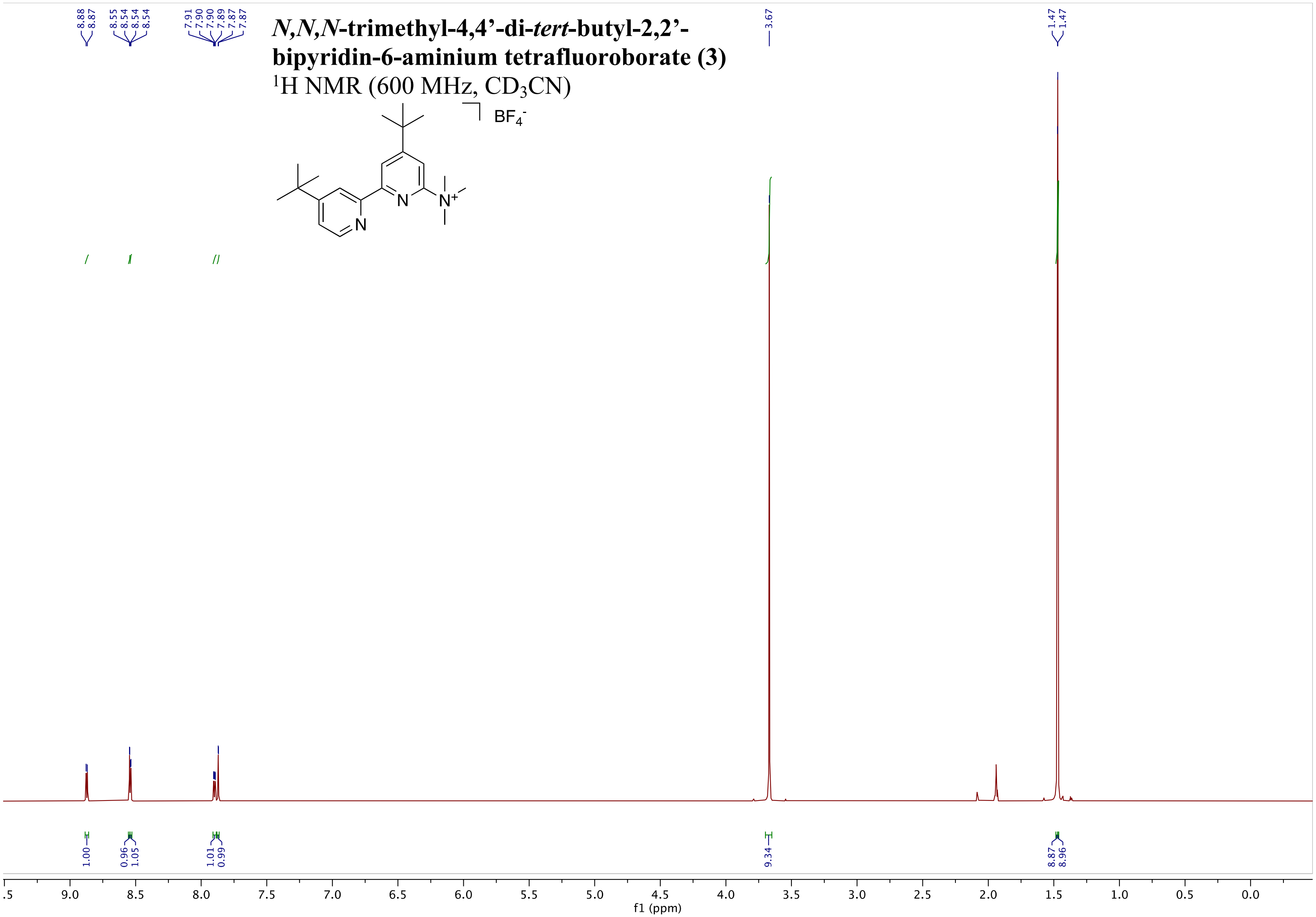

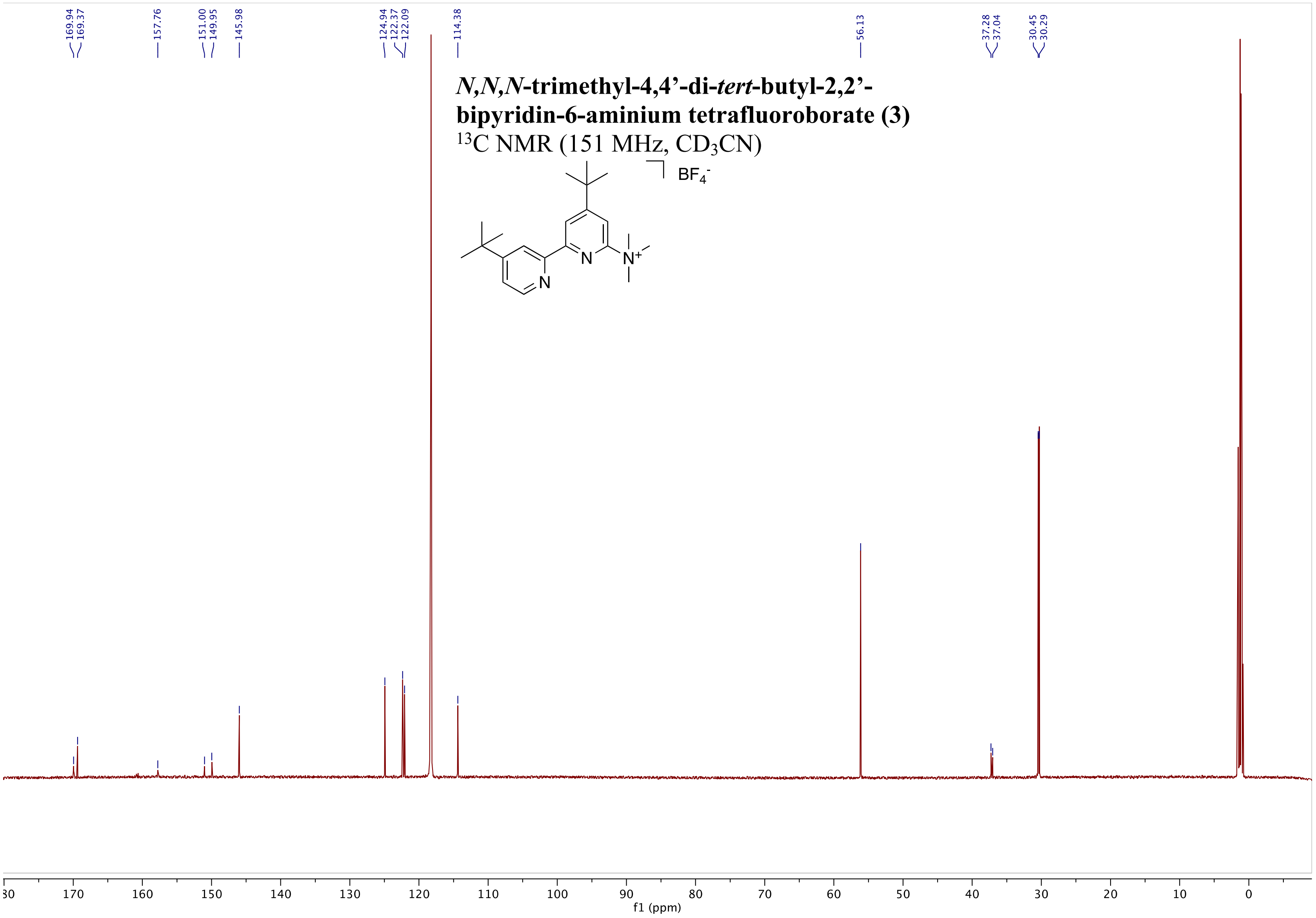
169.94
169.37
157.76
151.00
149.95
145.98
124.94
122.37
122.09
114.38
56.13
37.28
37.04
30.45
30.29
N,N,N-trimethyl-4,4'-di-*tert*-butyl-2,2'-bipyridin-6-aminium tetrafluoroborate (3)
^{13}C NMR (151 MHz, CD_3CN)
BF_4^-
N
N
N^+
170
160
150
140
130
120
110
100
90
80
70
60
50
40
30
20
10
0
f1 (ppm)

***N,N,N*-trimethyl-4,4'-di-*tert*-butyl-2,2'-bipyridin-6-aminium tetrafluoroborate (3)**

^{19}F NMR (376 MHz, CD_3CN)

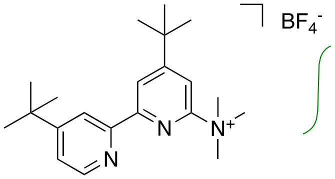

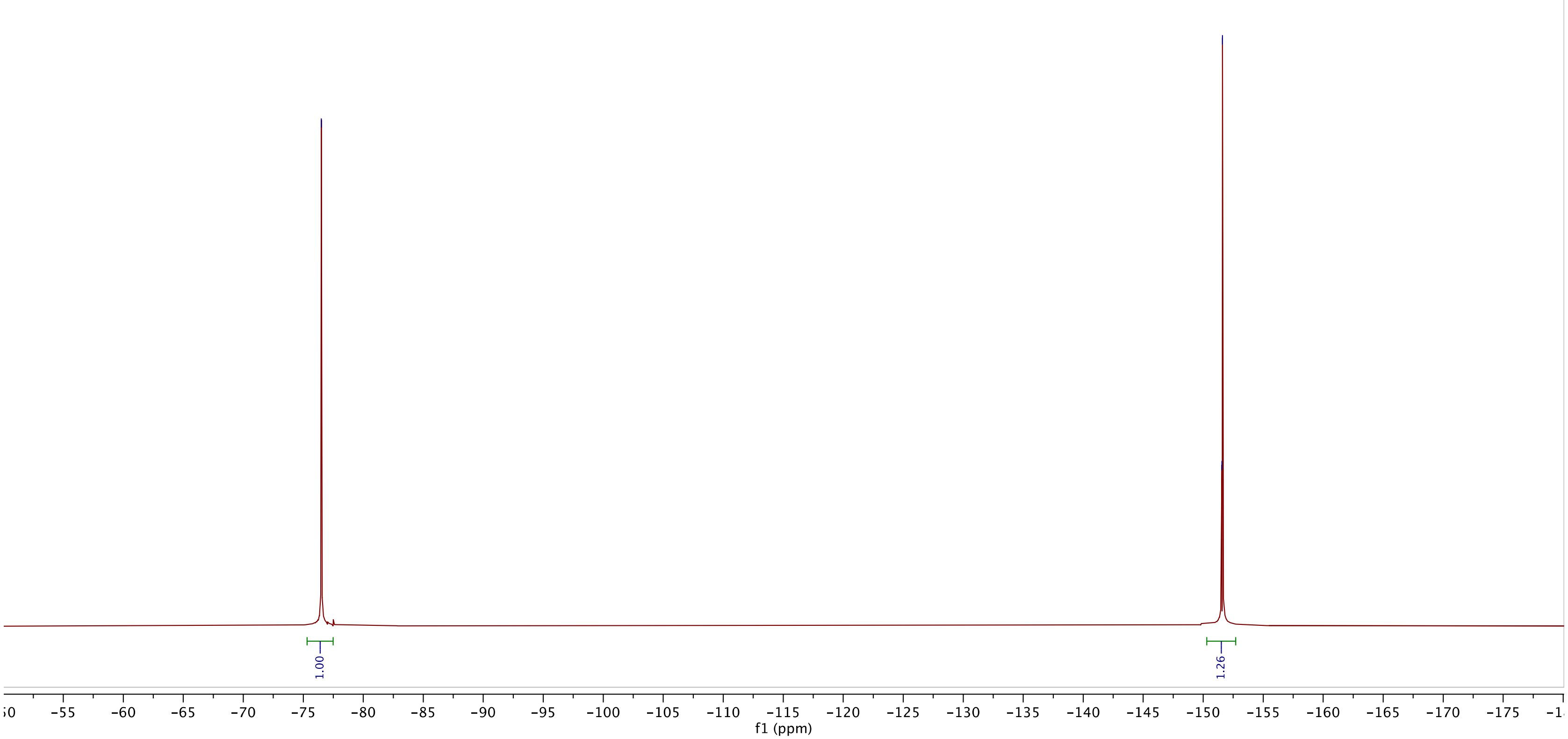

***N,N,N,N',N',N'*-hexamethyl-4,4'-di-*tert*-butyl-2,2'-bipyridin-6,6'-diaminium bis(tetrafluoroborate) (4a)**

^{1}H NMR (600 MHz, CD_3CN)

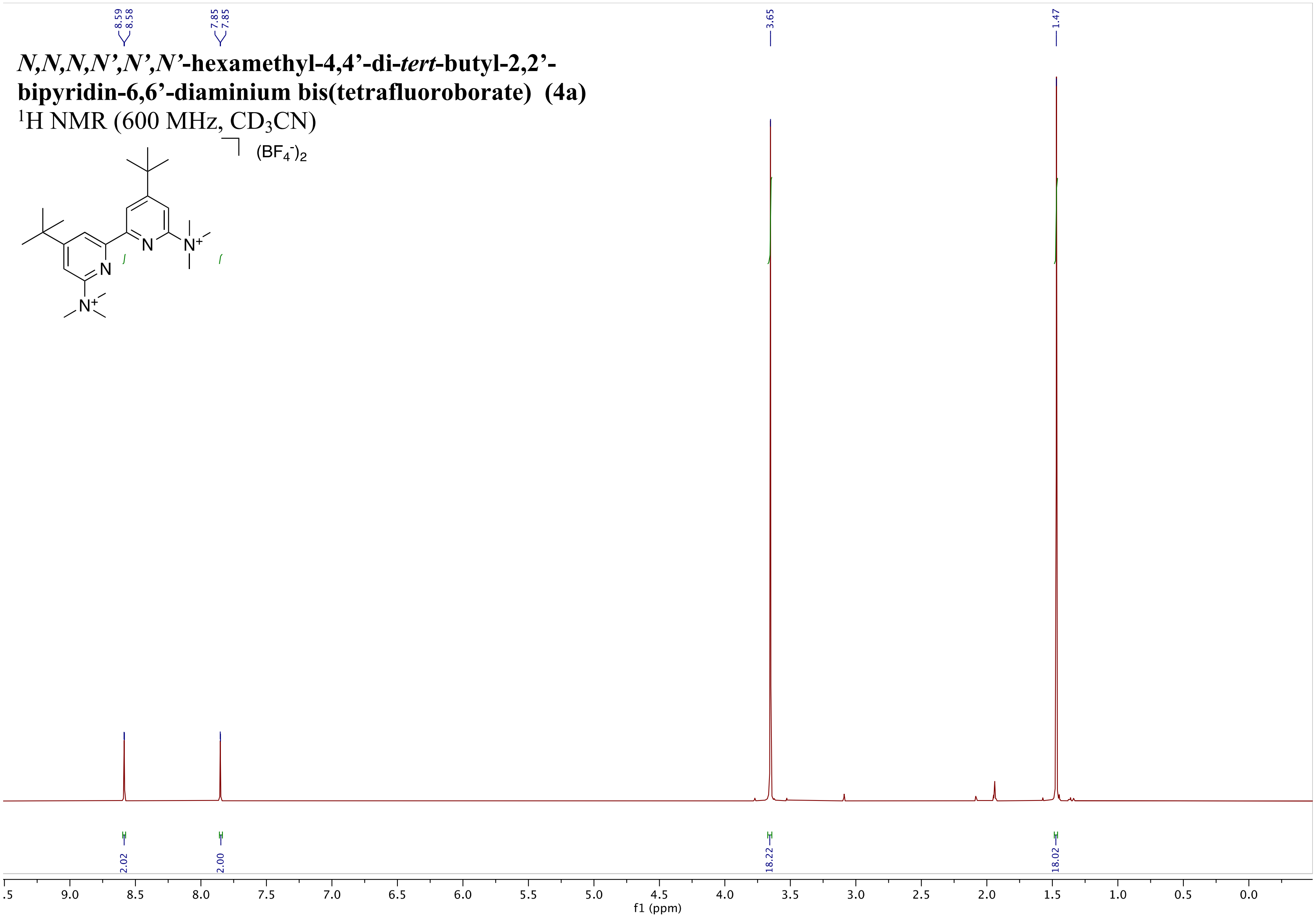

***N,N,N,N',N',N'*-hexamethyl-4,4'-di-*tert*-butyl-2,2'-bipyridin-6,6'-diaminium bis(tetrafluoroborate) (4a)**

^{13}C NMR (151 MHz, CD_3CN)

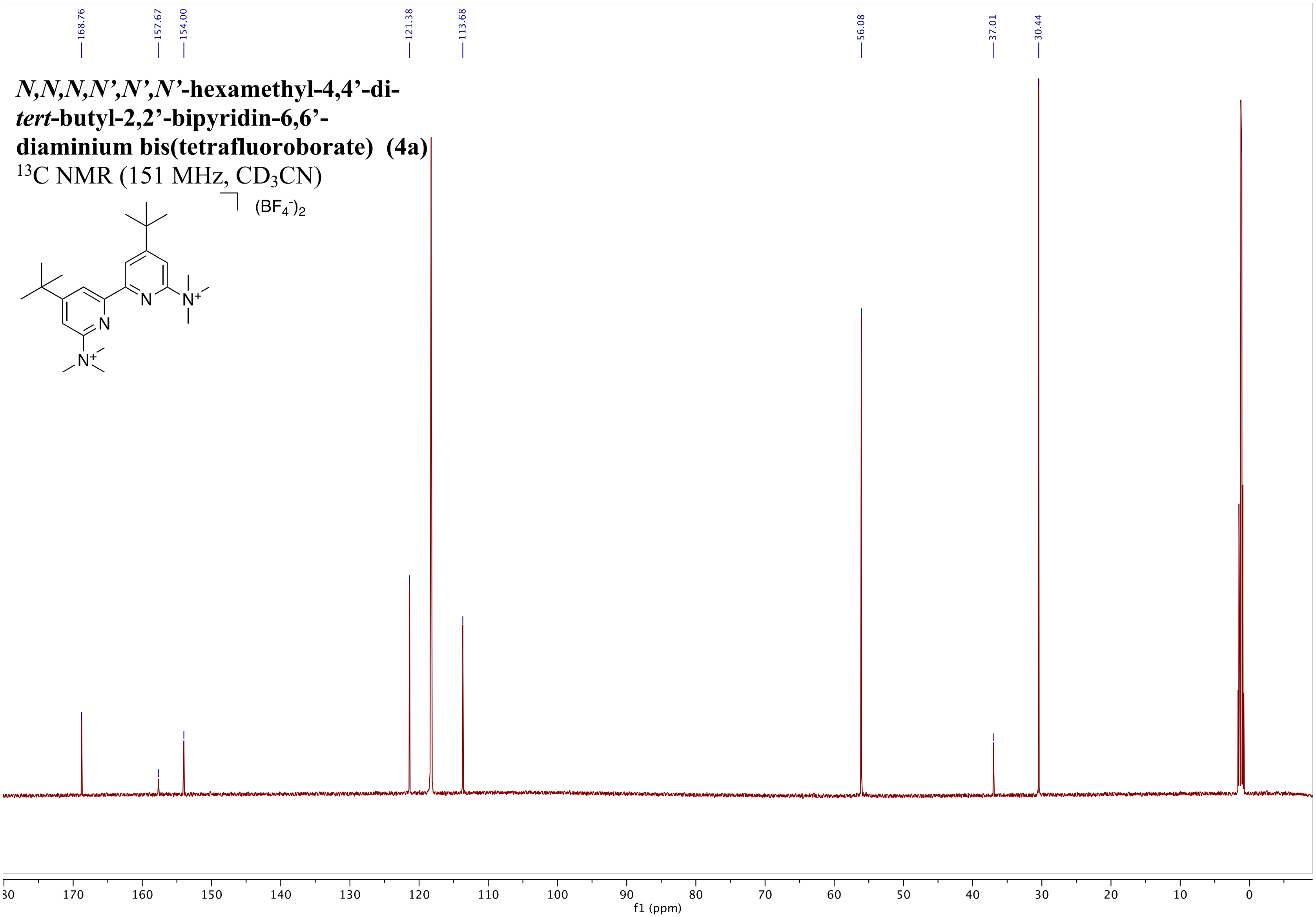

***N,N,N,N',N',N'*-hexamethyl-4,4'-di-*tert*-butyl-2,2'-bipyridin-6,6'-diaminium bis(tetrafluoroborate) (4a)**

^{19}F NMR (376 MHz, CD_3CN)

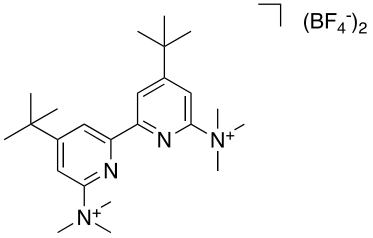

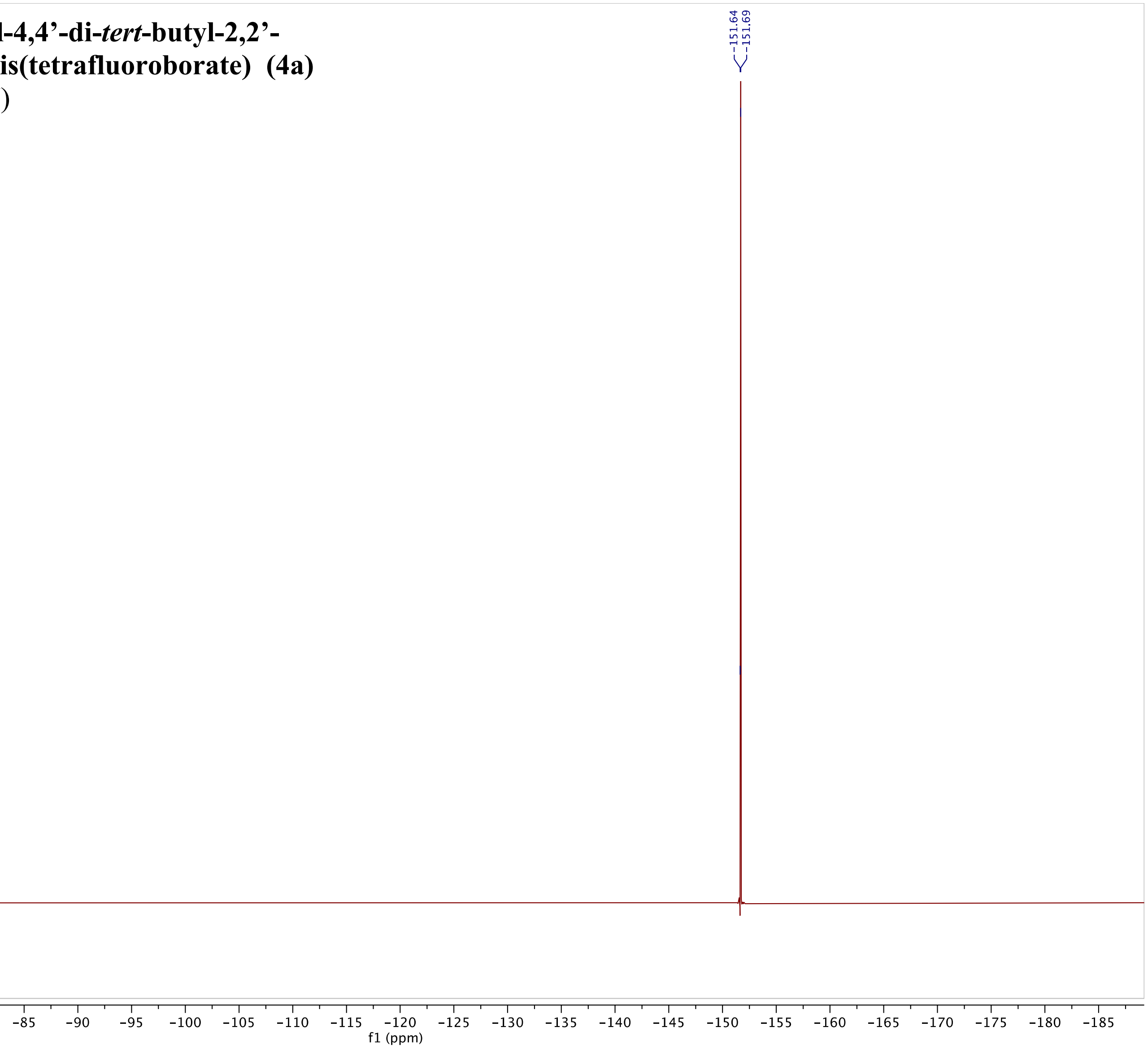

***N,N,N,N',N',N'*-hexamethyl-4,4'-di-*tert*-butyl-2,2'-bipyridin-6,6'-diaminium bis(trifluoroacetate) (4b)**

^{1}H NMR (600 MHz, CD_3OD)

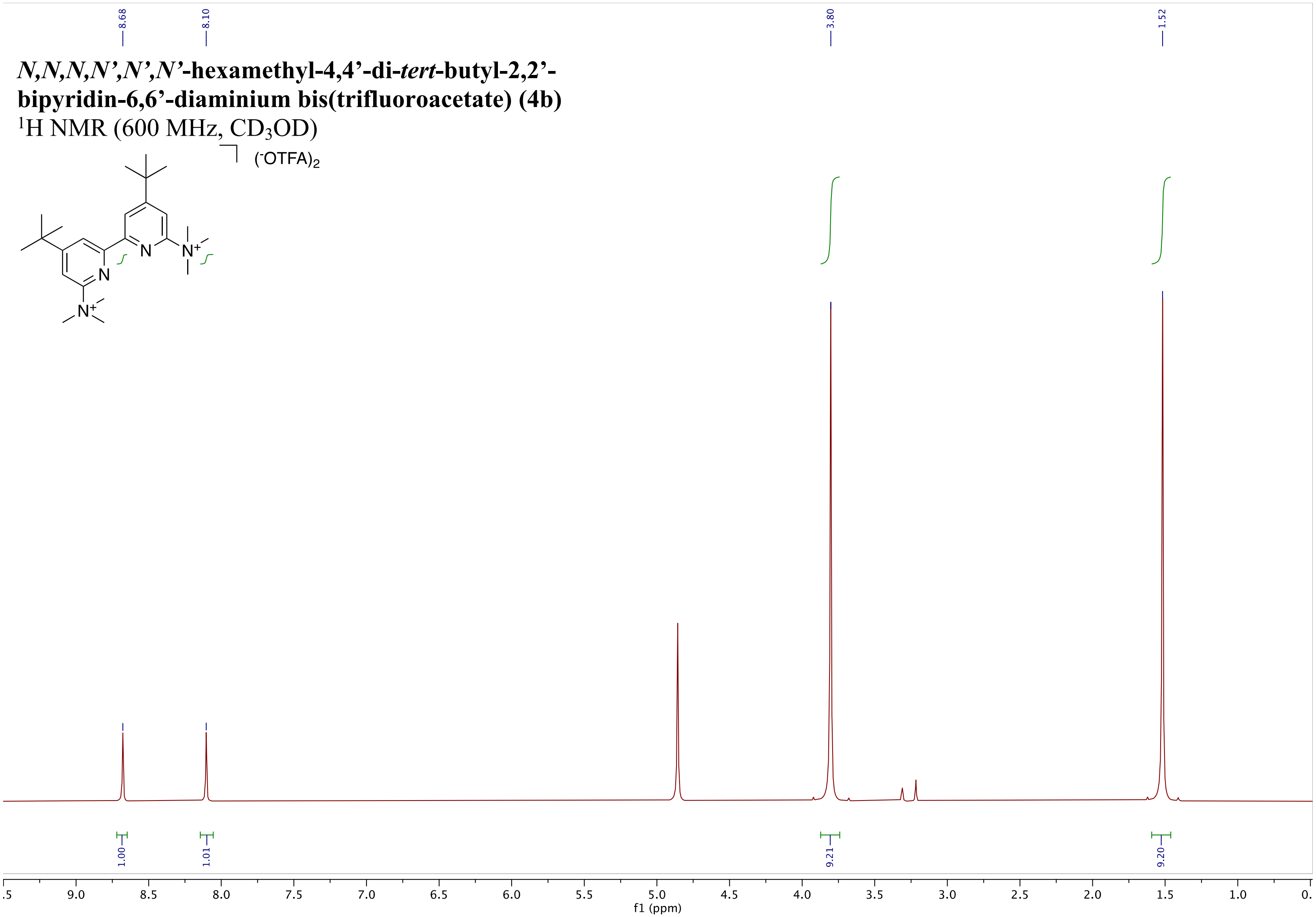

***N,N,N,N',N',N'*-hexamethyl-4,4'-di-*tert*-butyl-2,2'-bipyridin-6,6'-diaminium bis(trifluoroacetate) (4b)**

^{13}C NMR (151 MHz, CD_3OD)

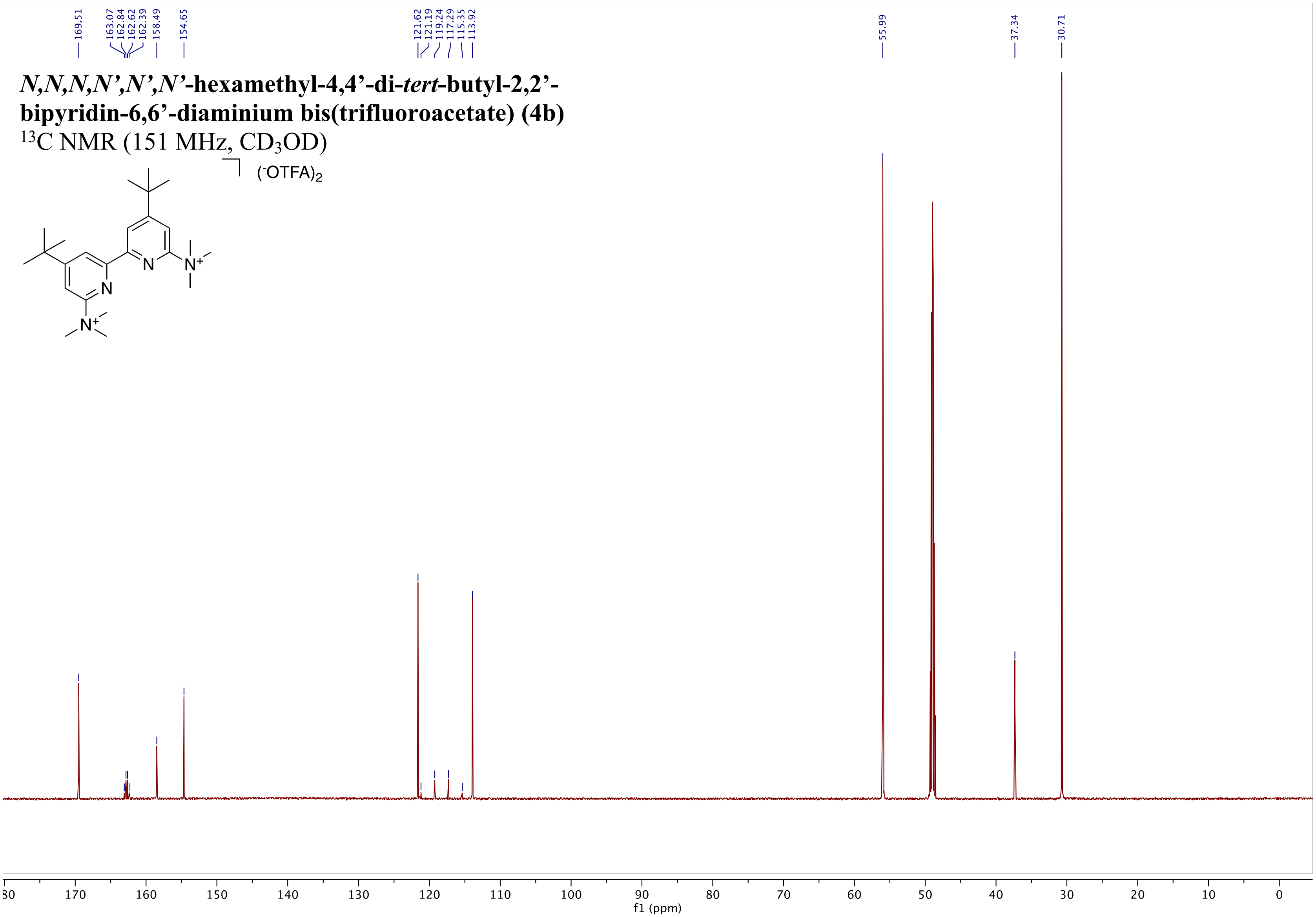

_N,N,N,N',N',N'_-hexamethyl-4,4'-di-_tert_-butyl-2,2'-bipyridin-6,6'-diaminium bis(trifluoroacetate) (4b)

^{19}F NMR (565 MHz, CD_3OD)

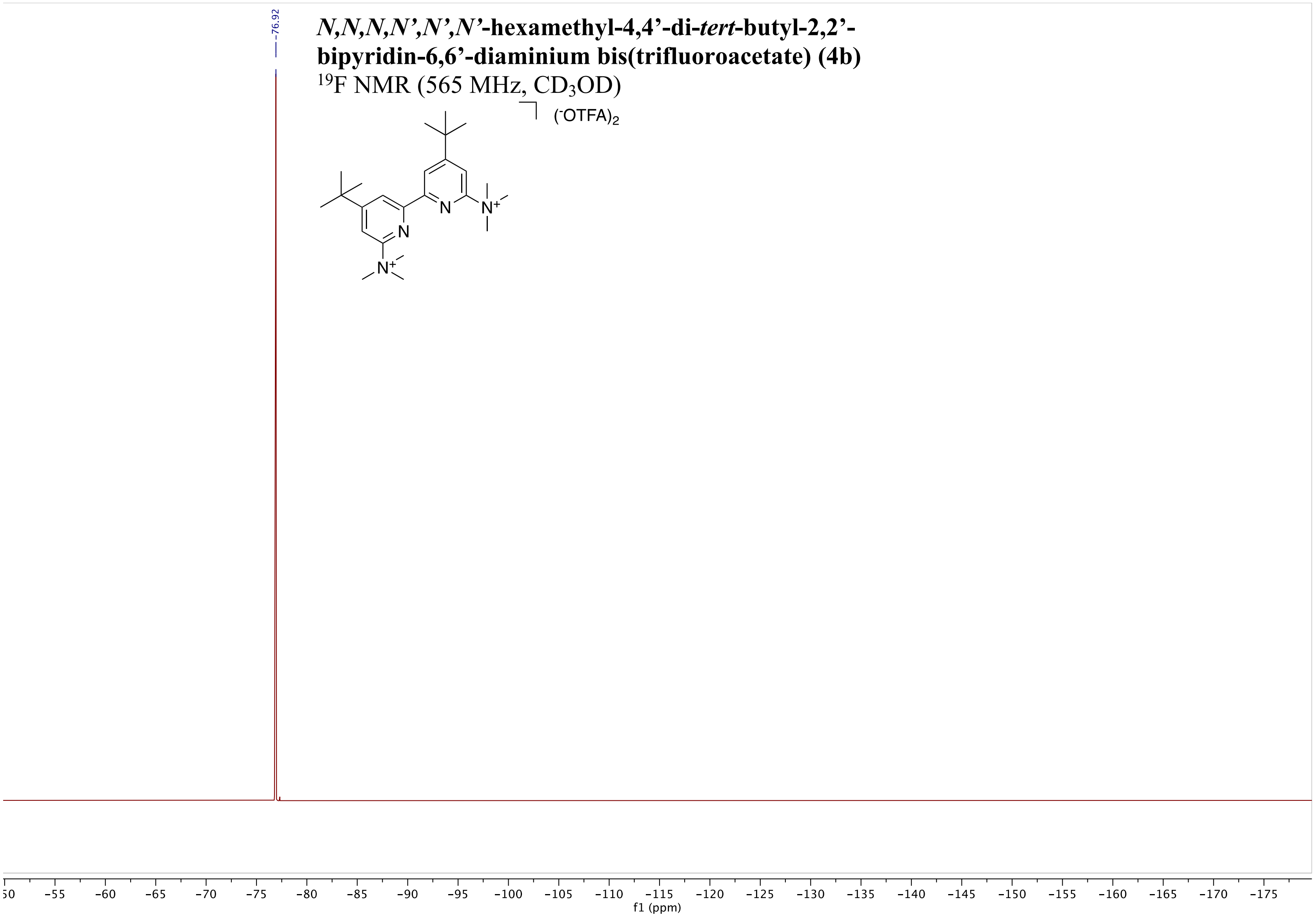

N,N,N-trimethyl-4,4'-bis(methoxycarbonyl)-2,2'-bipyridin-6-aminium tetrafluoroborate (5)

^{1}H NMR (600 MHz, DMSO-d$_6$)

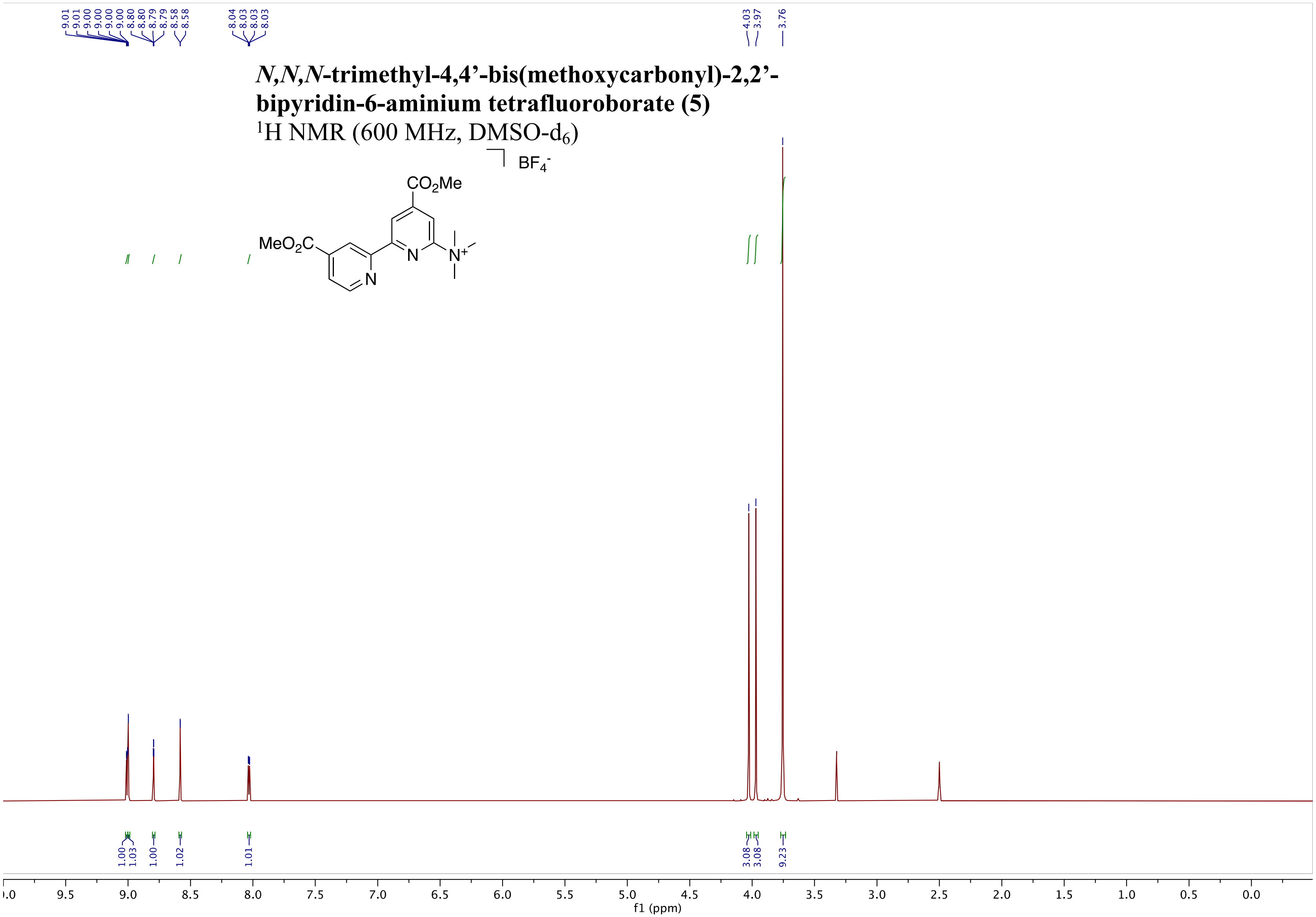

***N,N,N*-trimethyl-4,4'-bis(methoxycarbonyl)-2,2'-bipyridin-6-aminium tetrafluoroborate (5)**

^{13}C NMR (151 MHz, DMSO-d$_6$)

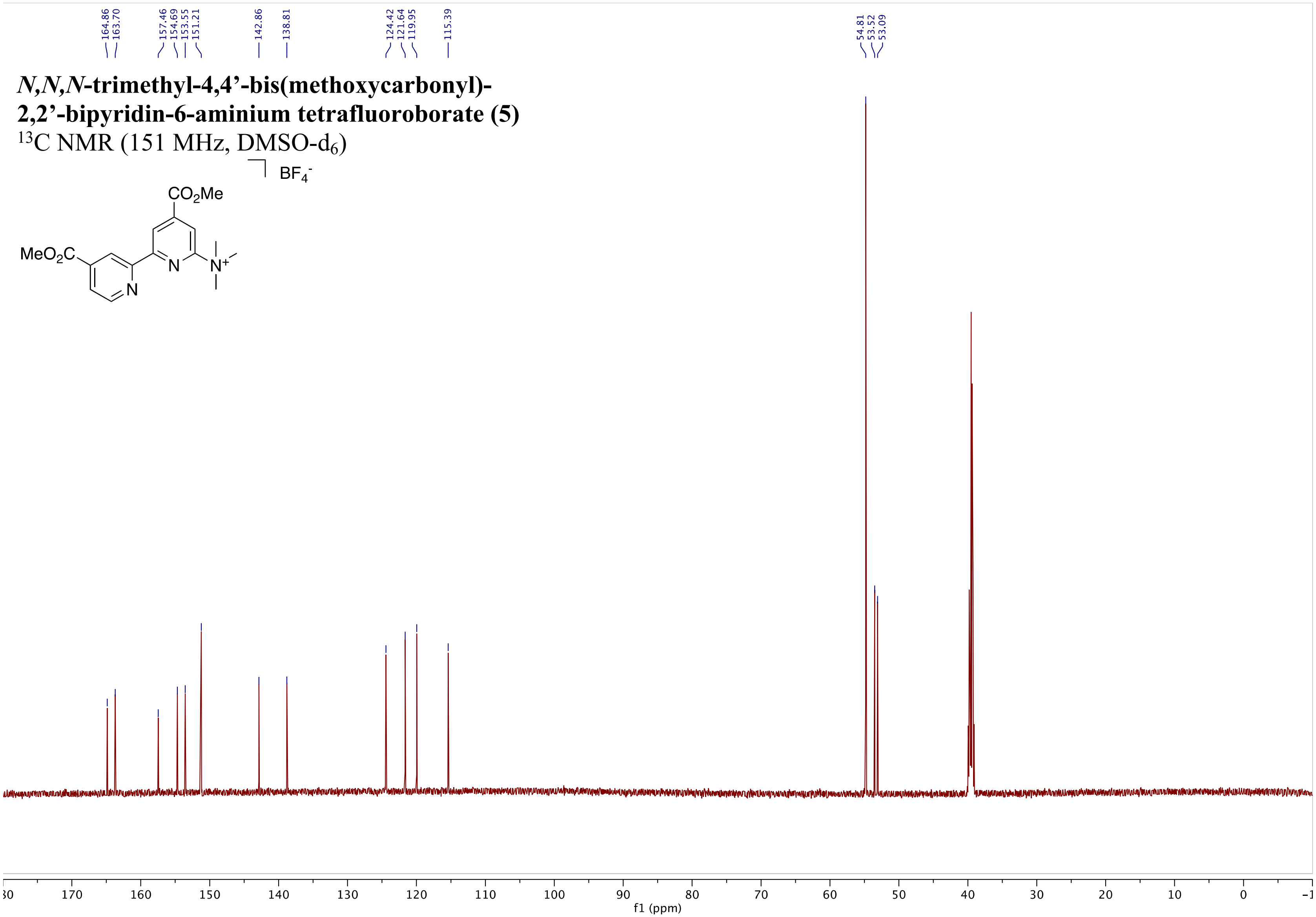

***N,N,N*-trimethyl-4,4'-bis(methoxycarbonyl)-2,2'-bipyridin-6-aminium tetrafluoroborate (5)**

^{19}F NMR (376 MHz, DMSO-d_6)

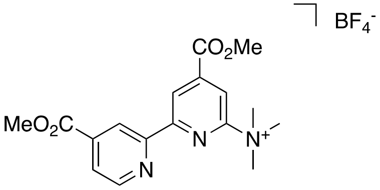

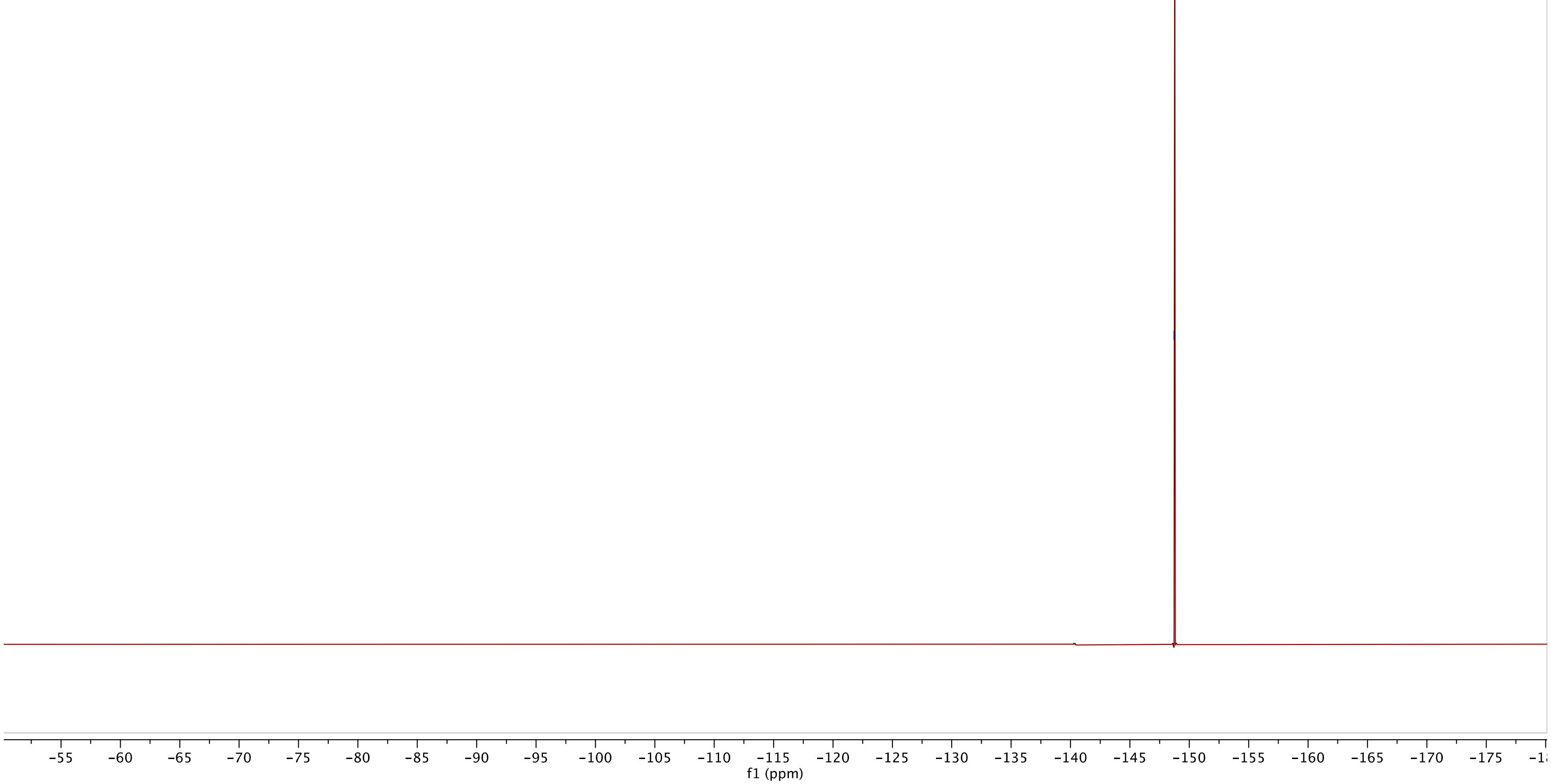

***N,N,N,N',N',N'*-hexamethyl-5,5'-dimethyl-2,2'-bipyridin-6,6'-diaminium bis(tetrafluoroborate) (6)**

^1H NMR (600 MHz, CD_3CN)

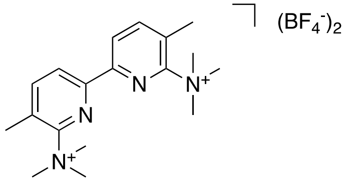

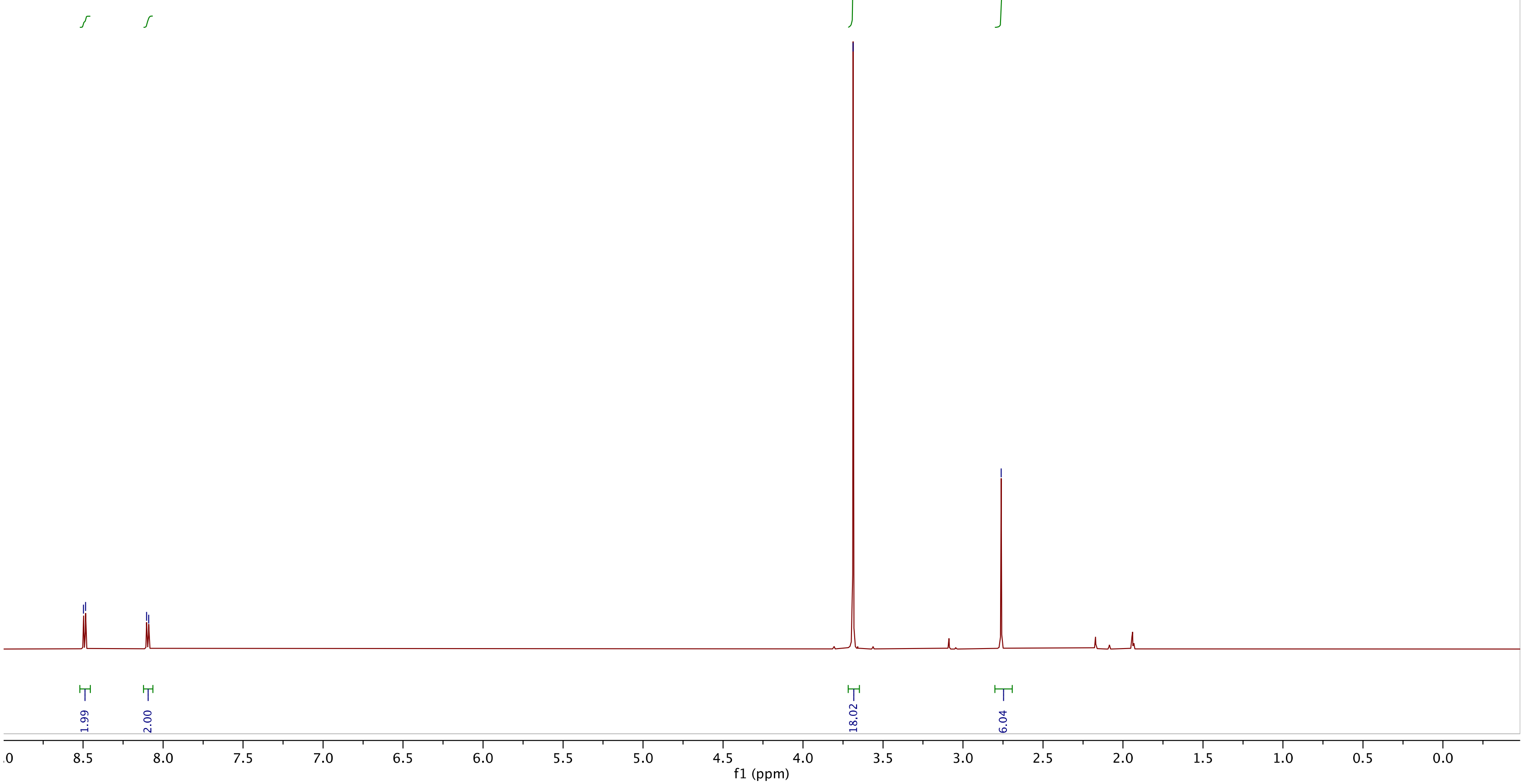

_N,N,N,N',N',N'_-hexamethyl-5,5'-dimethyl-2,2'-bipyridin-6,6'-diaminium bis(tetrafluoroborate) (6)

^{13}C NMR (151 MHz, CD_3CN)

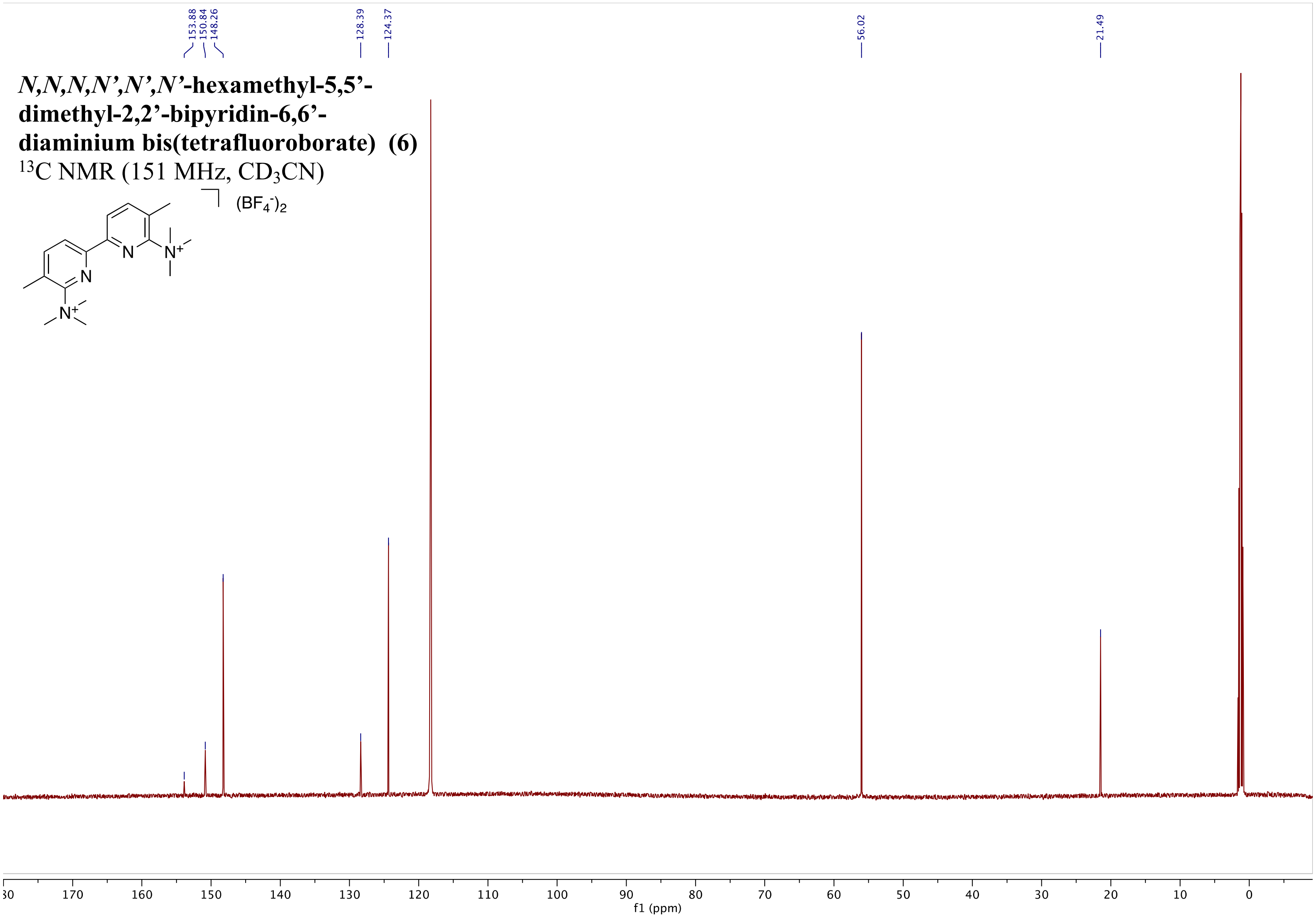

***N,N,N,N',N',N'*-hexamethyl-5,5'-dimethyl-2,2'-bipyridin-6,6'-diaminium bis(tetrafluoroborate) (6)**

^{19}F NMR (376 MHz, CD_3CN)

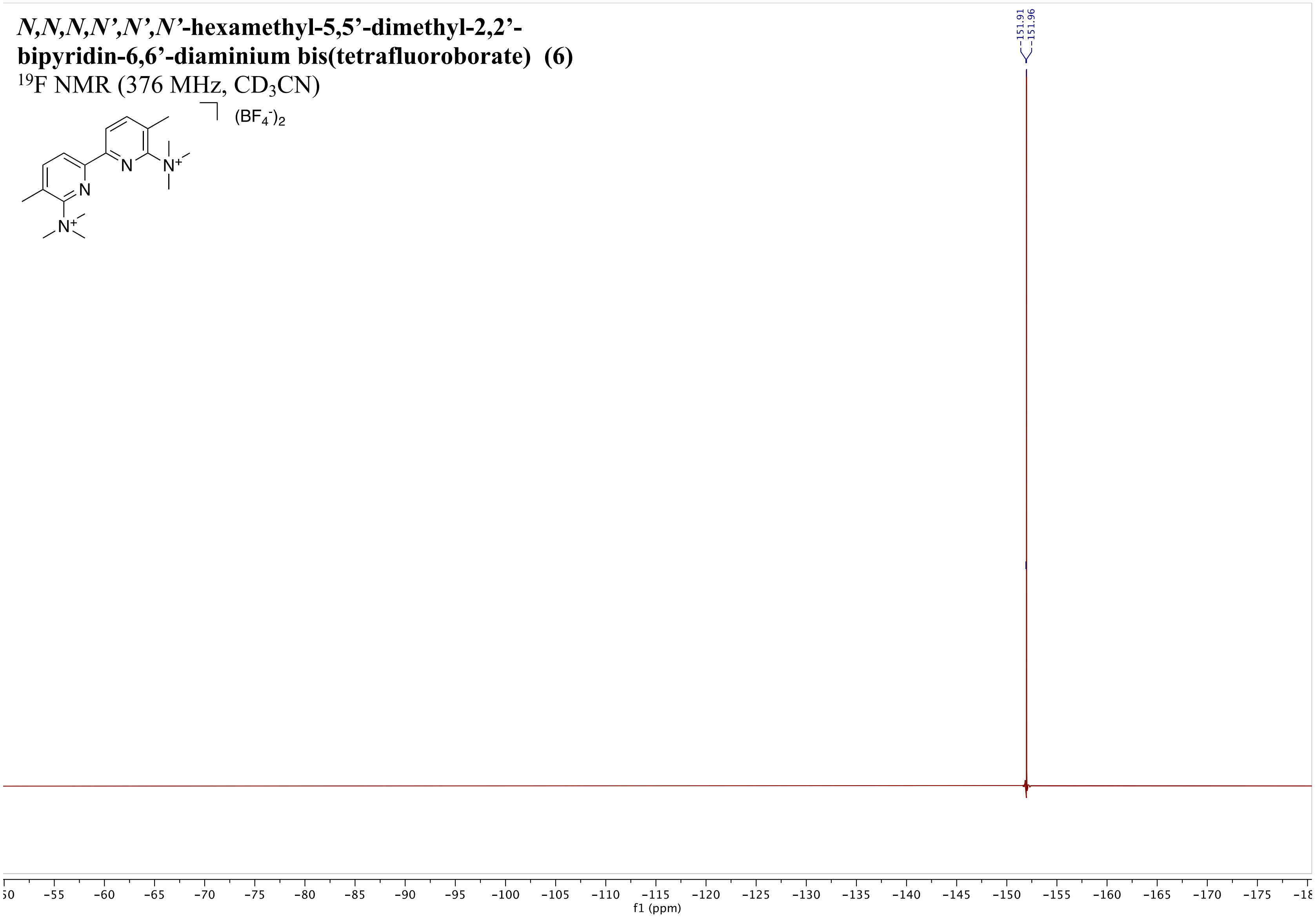

D$_8$-*N,N,N,N',N',N'*-hexamethyl-2,2'-bipyridin-6,6'-diaminium bis(tetrafluoroborate) (7)

^{1}H NMR (600 MHz, CD_3CN)

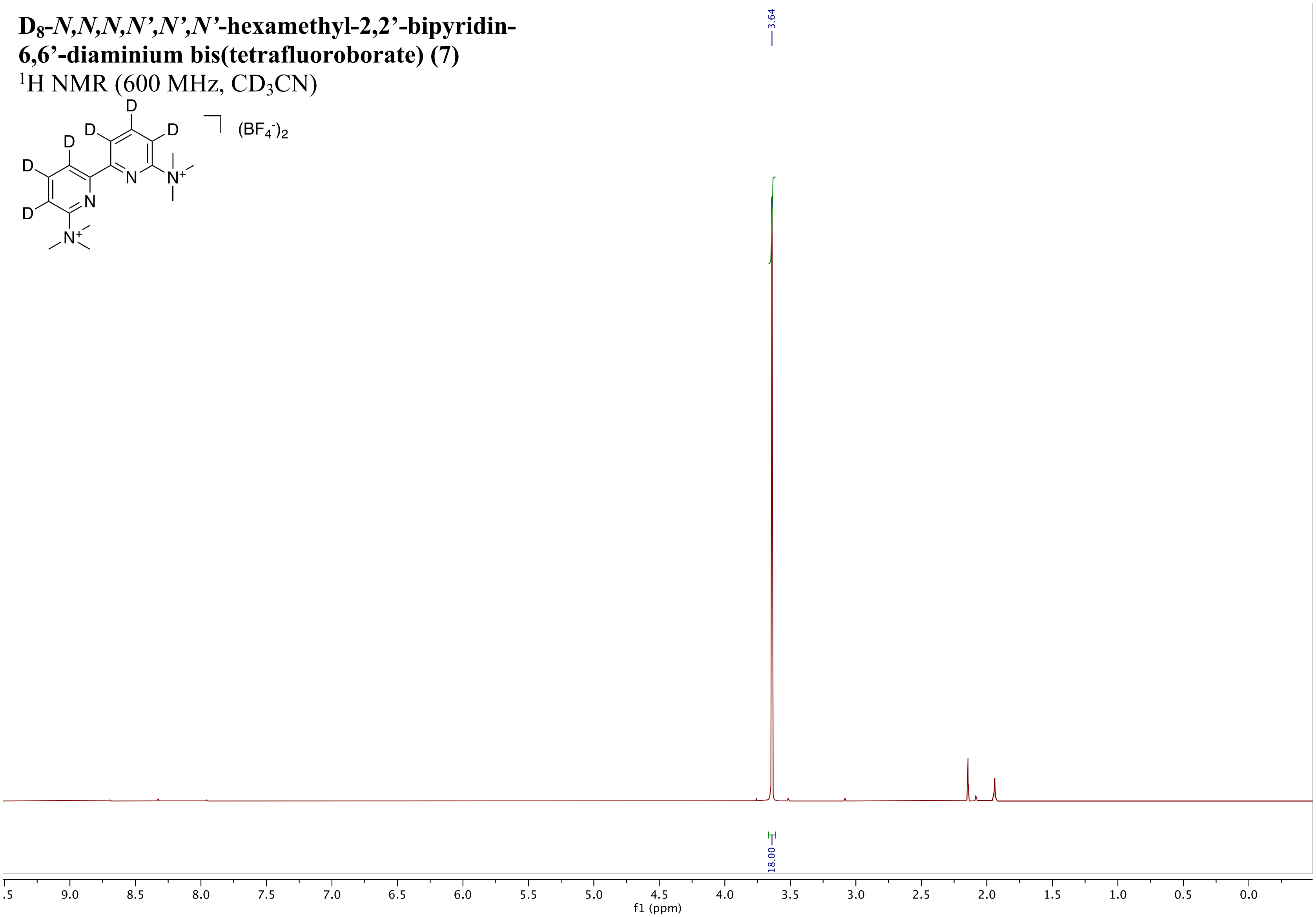

D$_8$-*N,N,N,N',N',N'*-hexamethyl-2,2'-bipyridin-6,6'-diaminium bis(tetrafluoroborate) (7)

^{2}H NMR (92 MHz, CD_3CN)

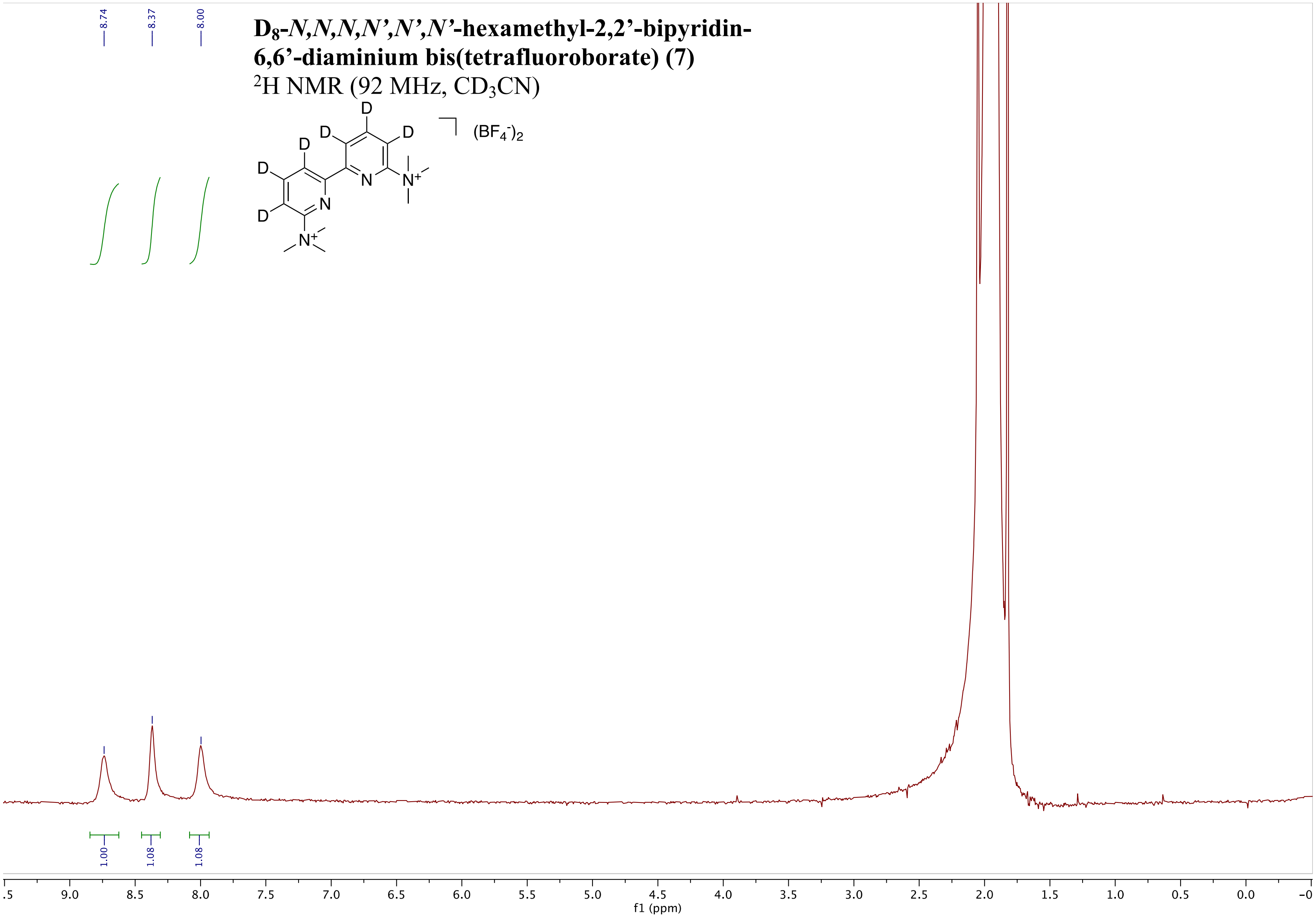

D_8-*N,N,N,N’,N’,N’*-hexamethyl-2,2’-bipyridin-6,6’-diaminium bis(tetrafluoroborate) (7)

^{13}C NMR (151 MHz, CD_3CN)

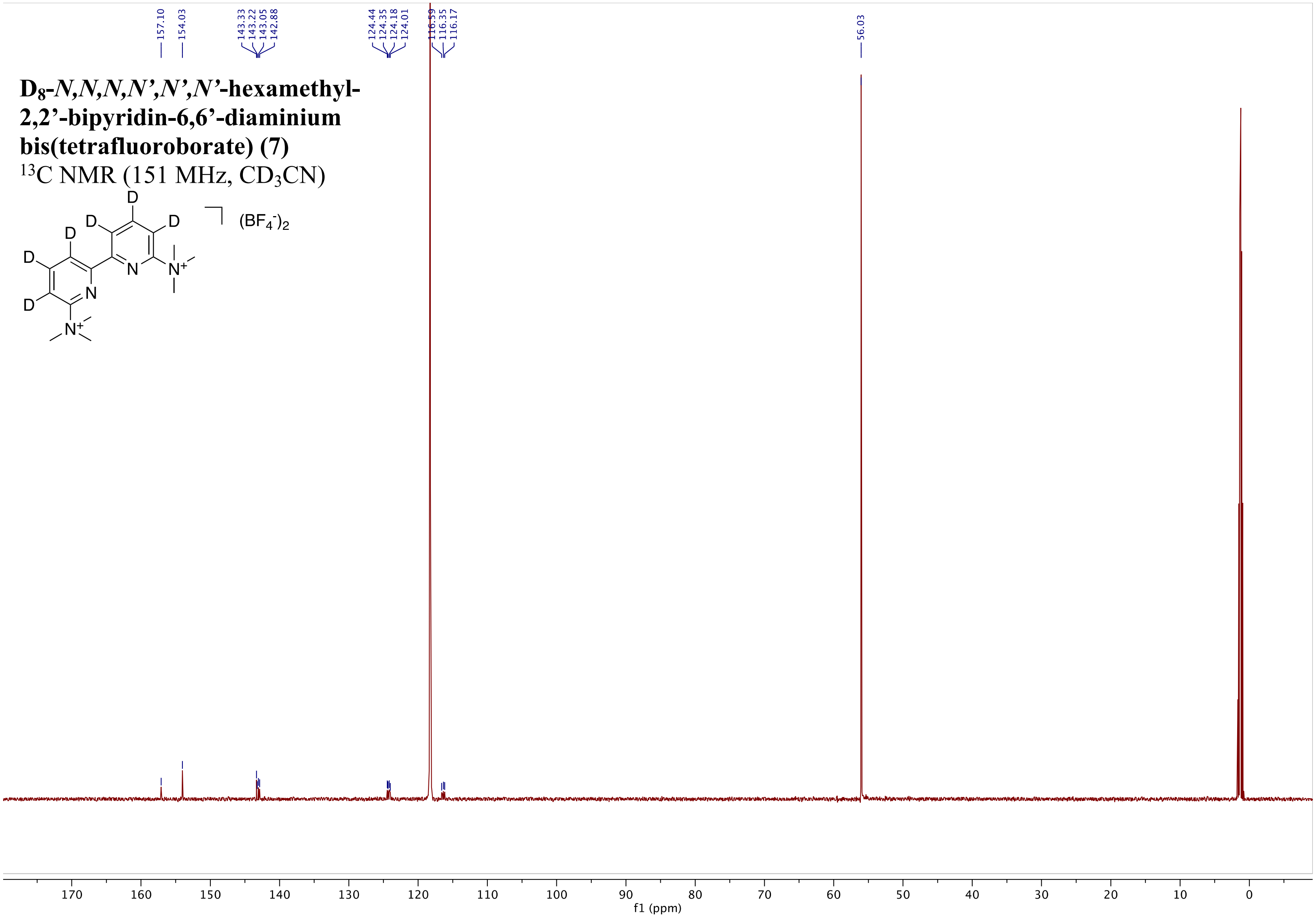

D$_8$-*N,N,N,N',N',N'*-hexamethyl-2,2'-bipyridin-6,6'-diaminium bis(tetrafluoroborate) (7)

^{13}C NMR (151 MHz, CD_3CN): Zoom

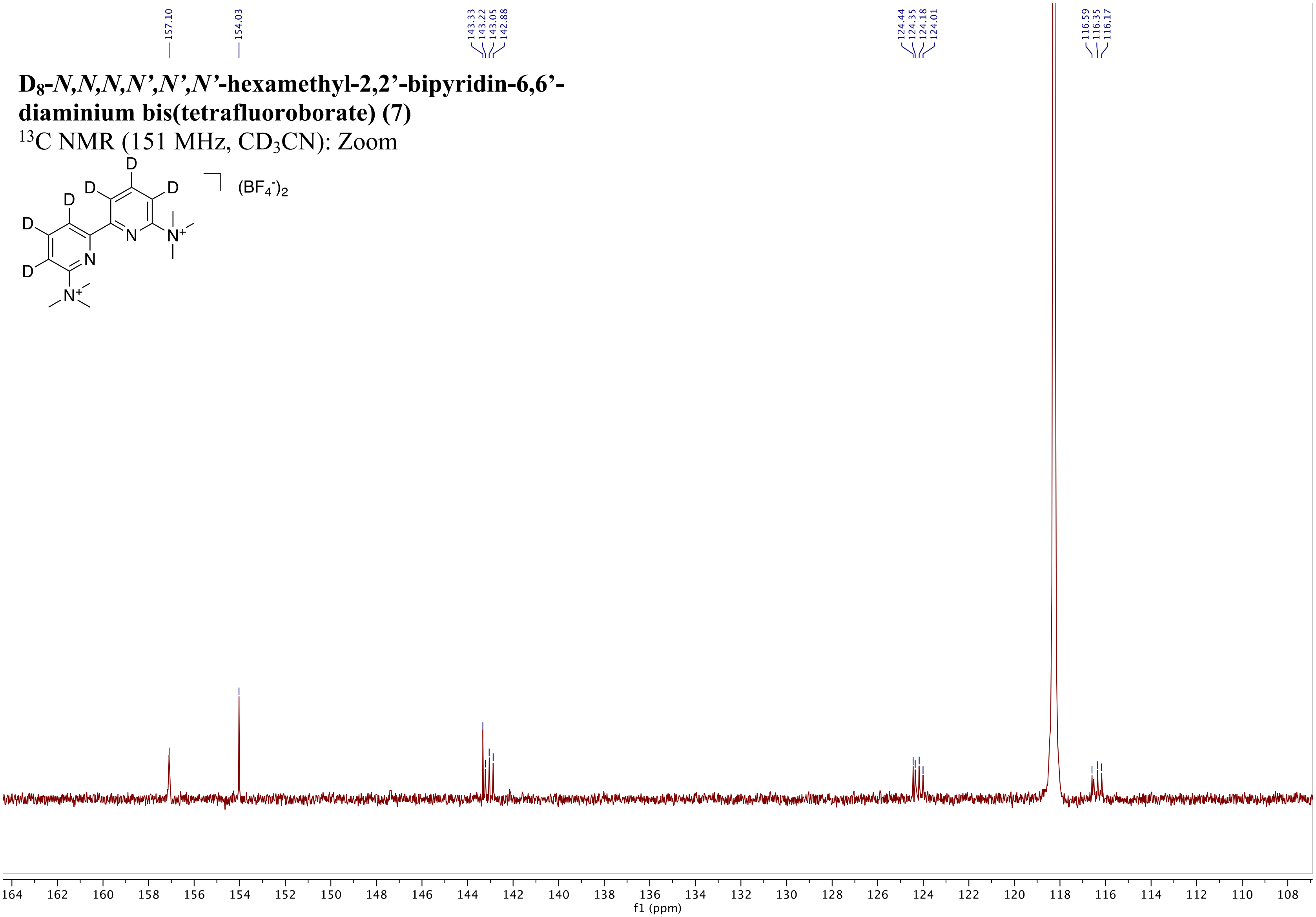

D$_8$-*N,N,N,N',N',N'*-hexamethyl-2,2'-bipyridin-6,6'-diaminium bis(tetrafluoroborate) (7)

^{19}F NMR (376 MHz, CD_3CN)

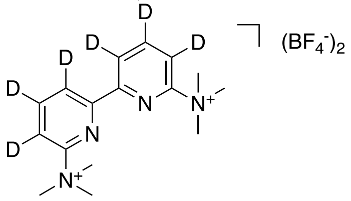

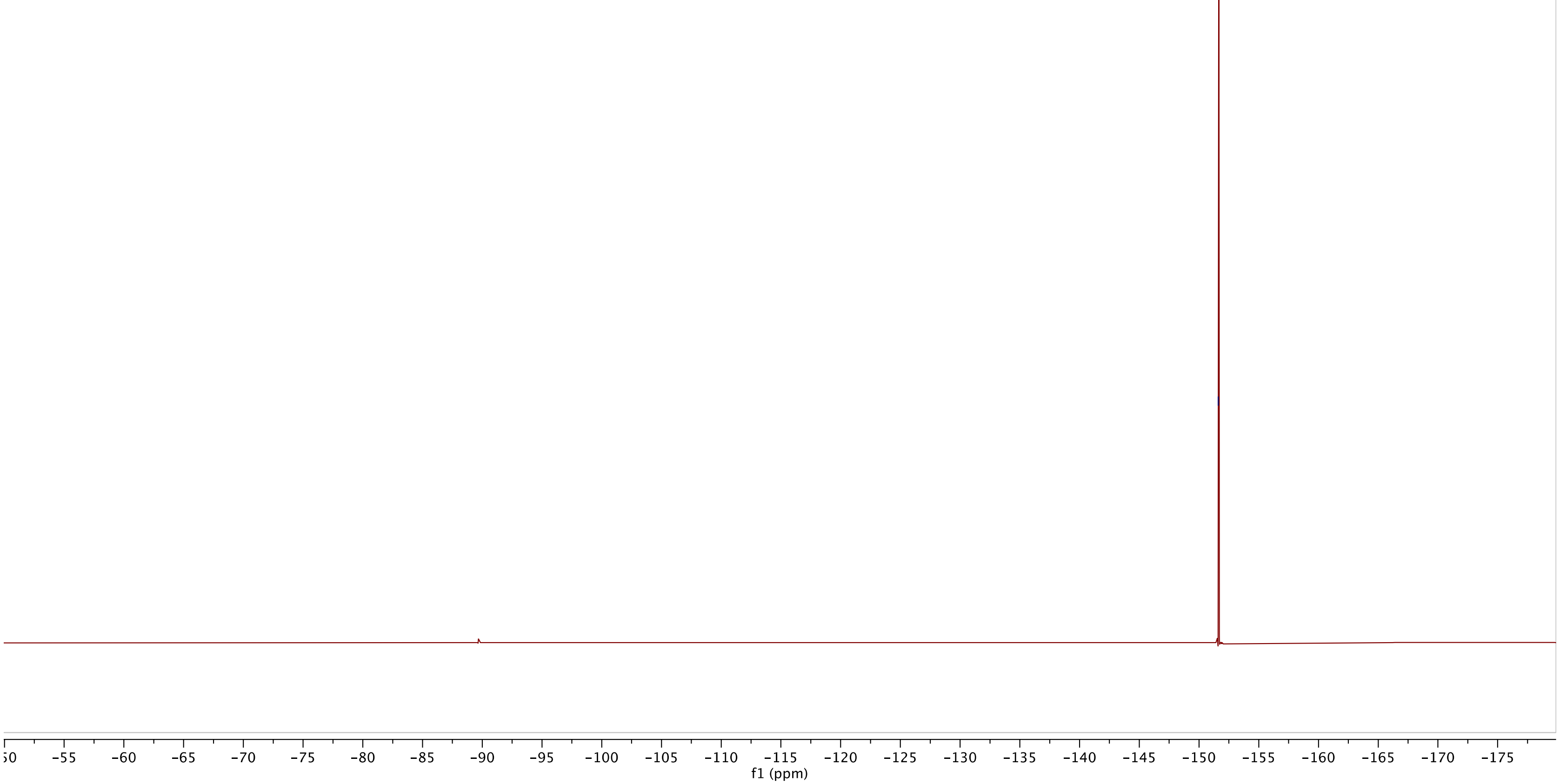

***N,N,N*-trimethyl-1,10-phenanthrolin-2-aminium tetrafluoroborate (8)**

^{1}H NMR (600 MHz, CD_3CN)

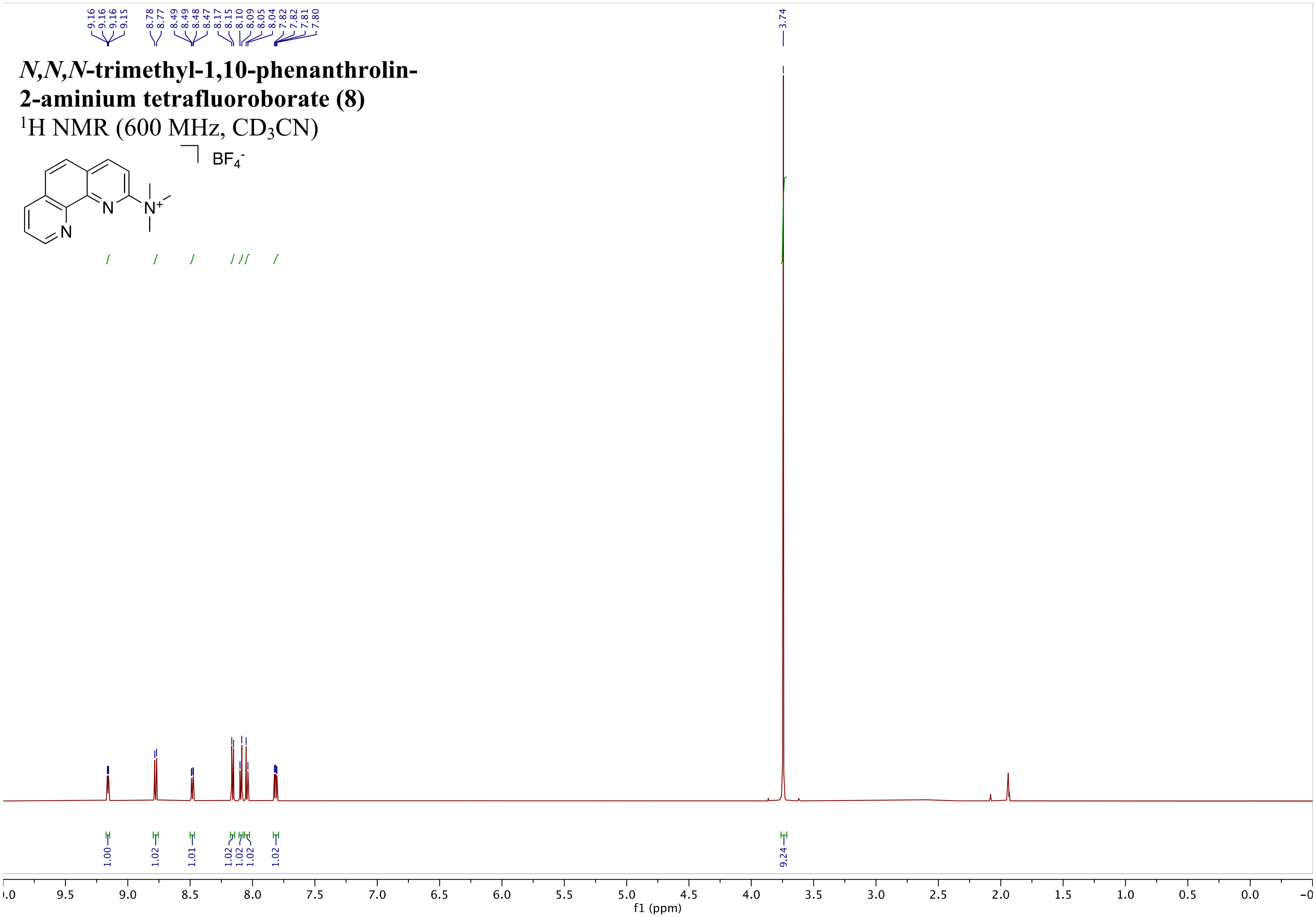

***N,N,N*-trimethyl-1,10-phenanthrolin-2-aminium tetrafluoroborate (8)**

^{13}C NMR (151 MHz, CD_3CN)

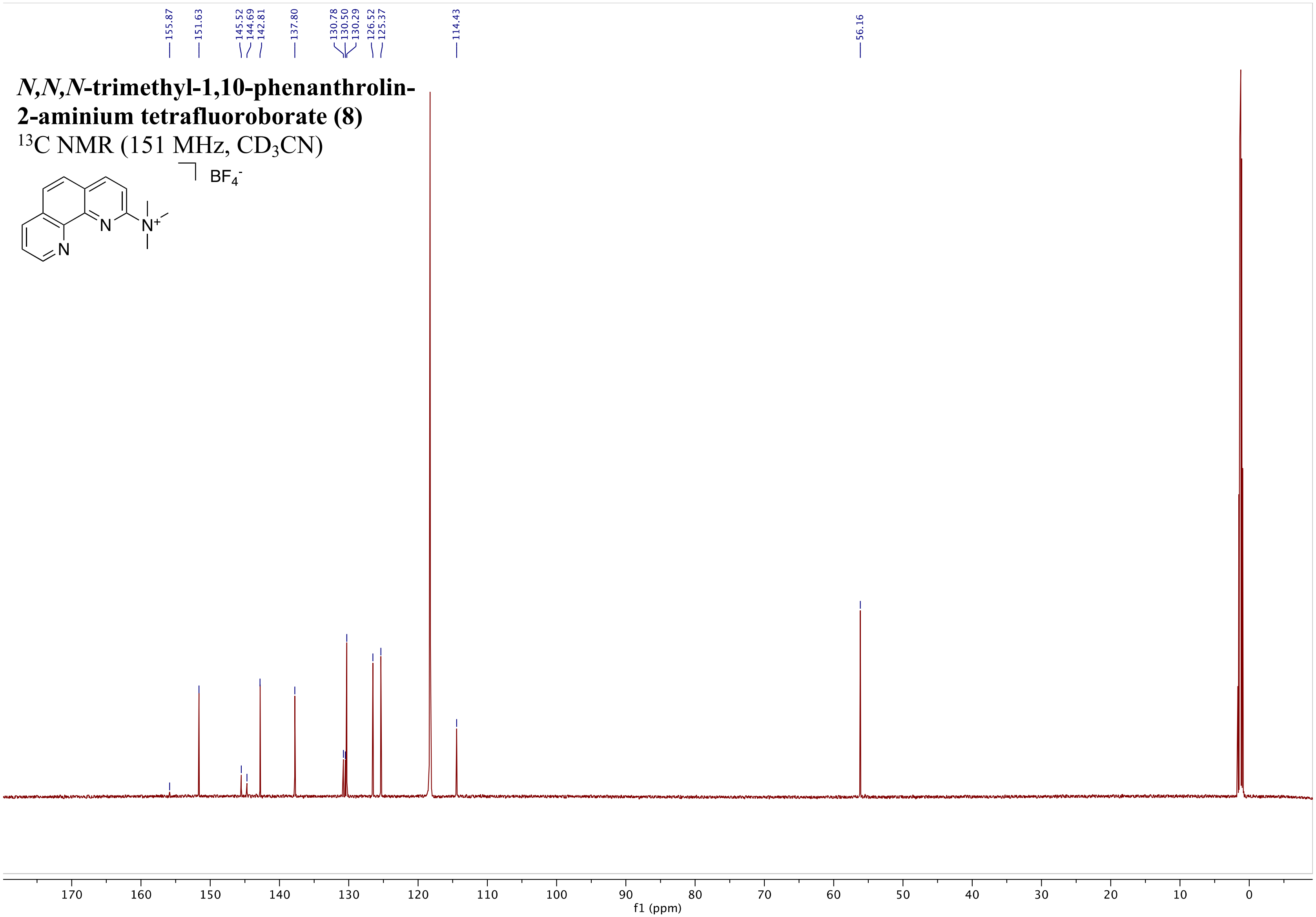

N,N,N-trimethyl-1,10-phenanthrolin-2-aminium tetrafluoroborate (8)

^{19}F NMR (376 MHz, CD_3CN)

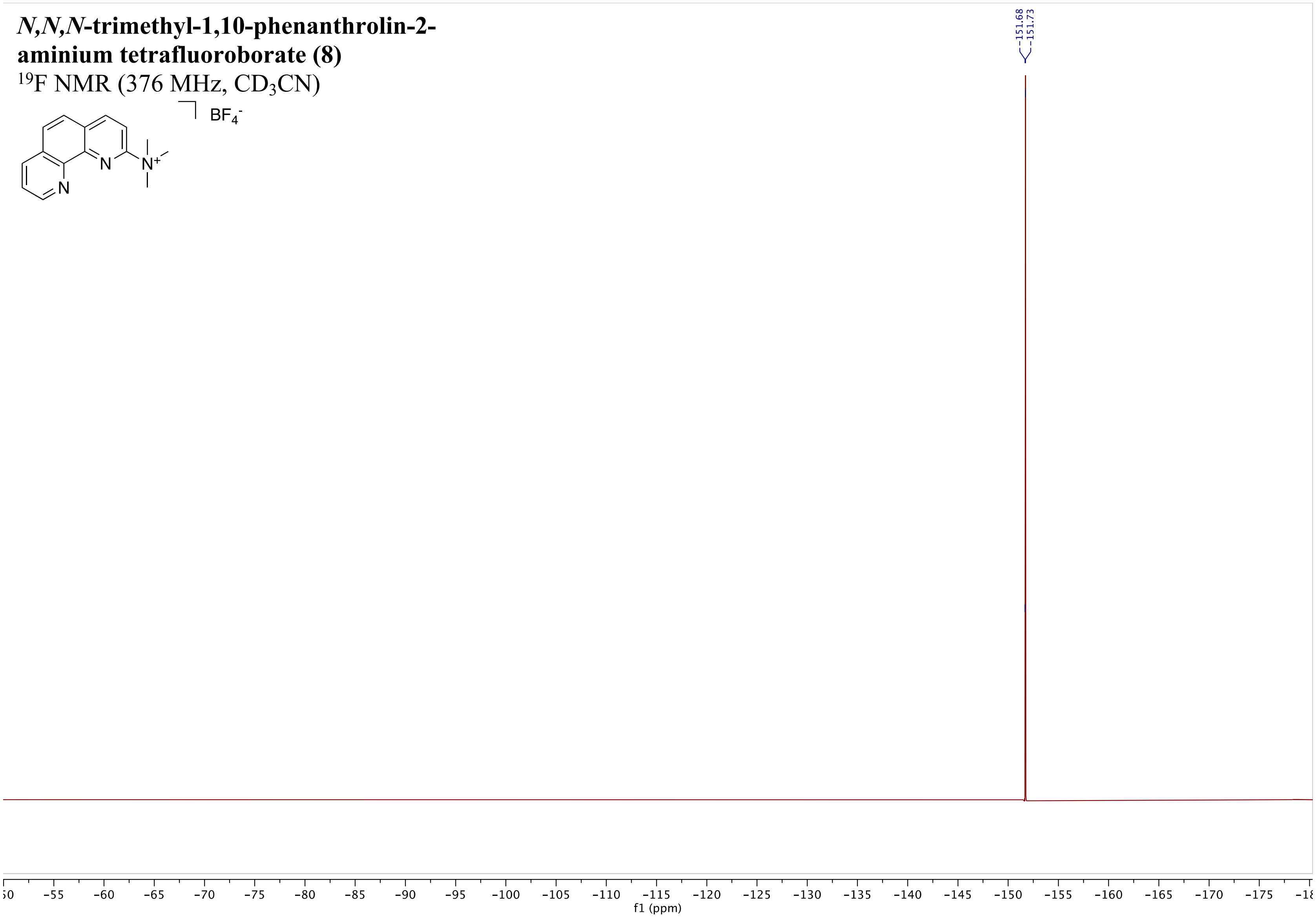

_N,N,N_-trimethyl-6-phenylpyridin-2-aminium tetrafluoroborate (9)

^{1}H NMR (600 MHz, CD_3CN)

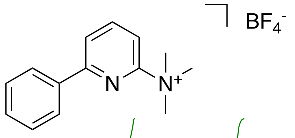

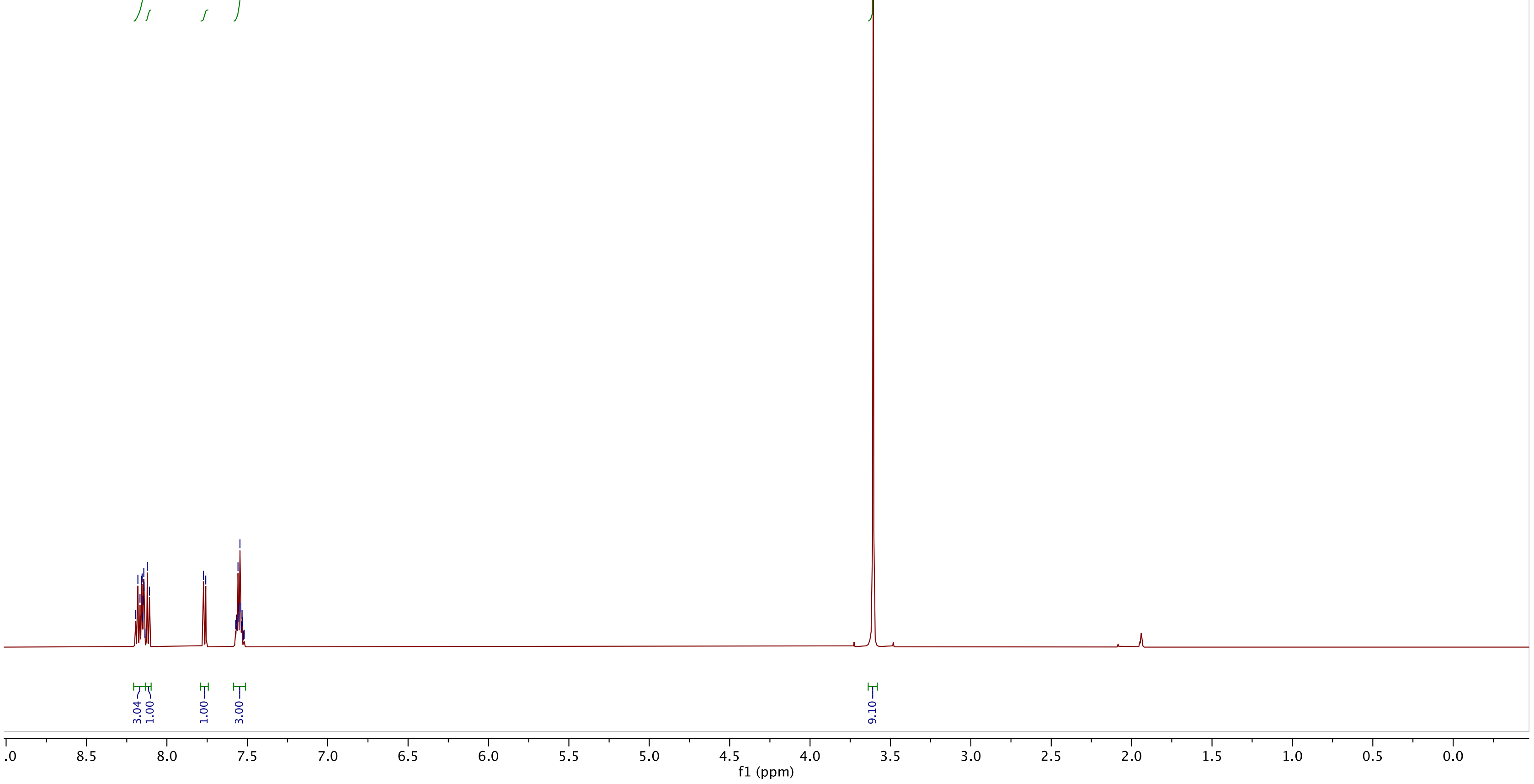

***N,N,N*-trimethyl-6-phenylpyridin-2-aminium tetrafluoroborate (9)**

^{13}C NMR (151 MHz, CD_3CN)

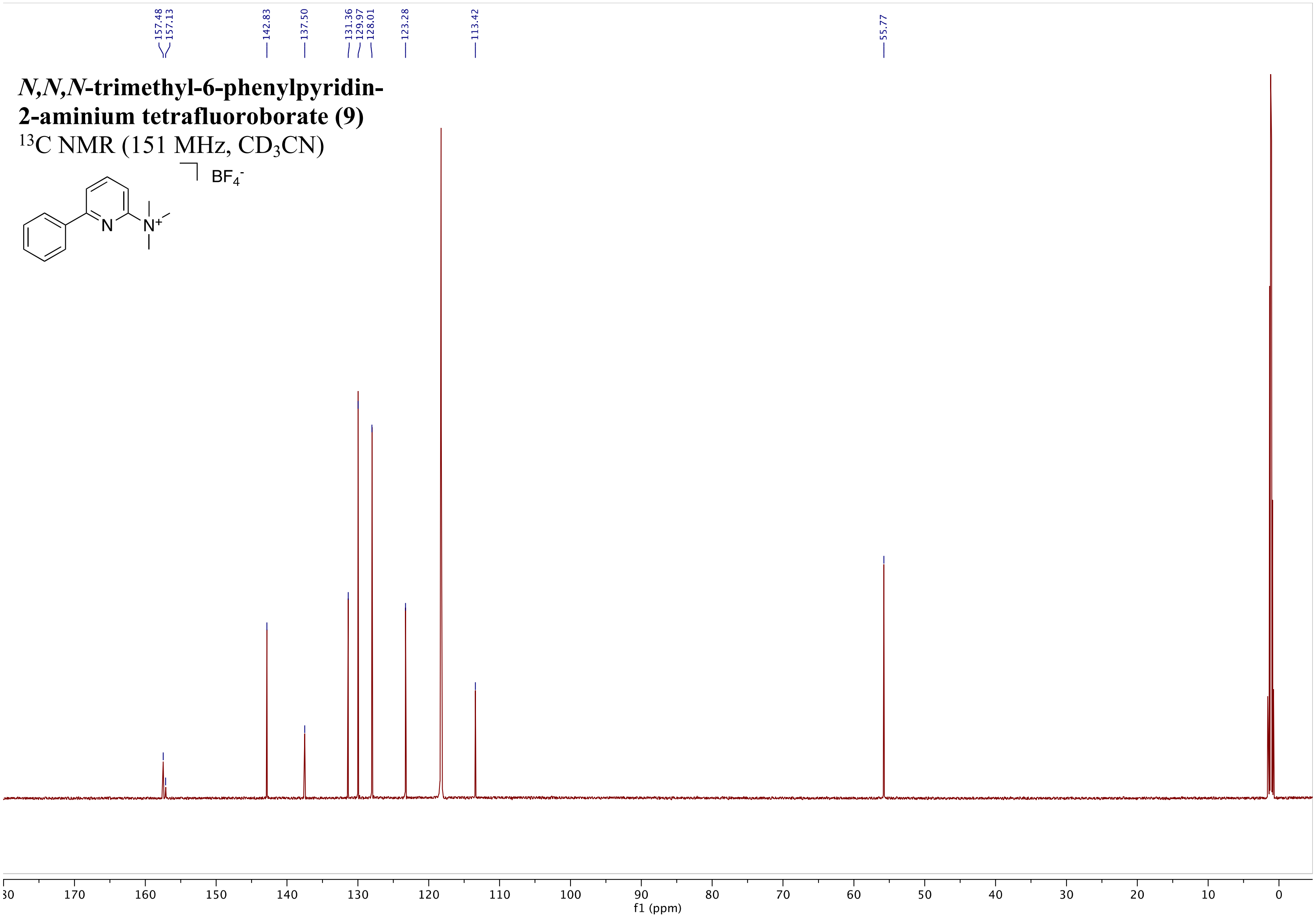

_N,N,N_-trimethyl-6-phenylpyridin-2-aminium tetrafluoroborate (9)

^{19}F NMR (565 MHz, CD_3CN)

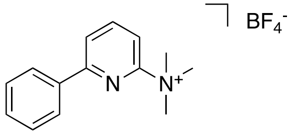

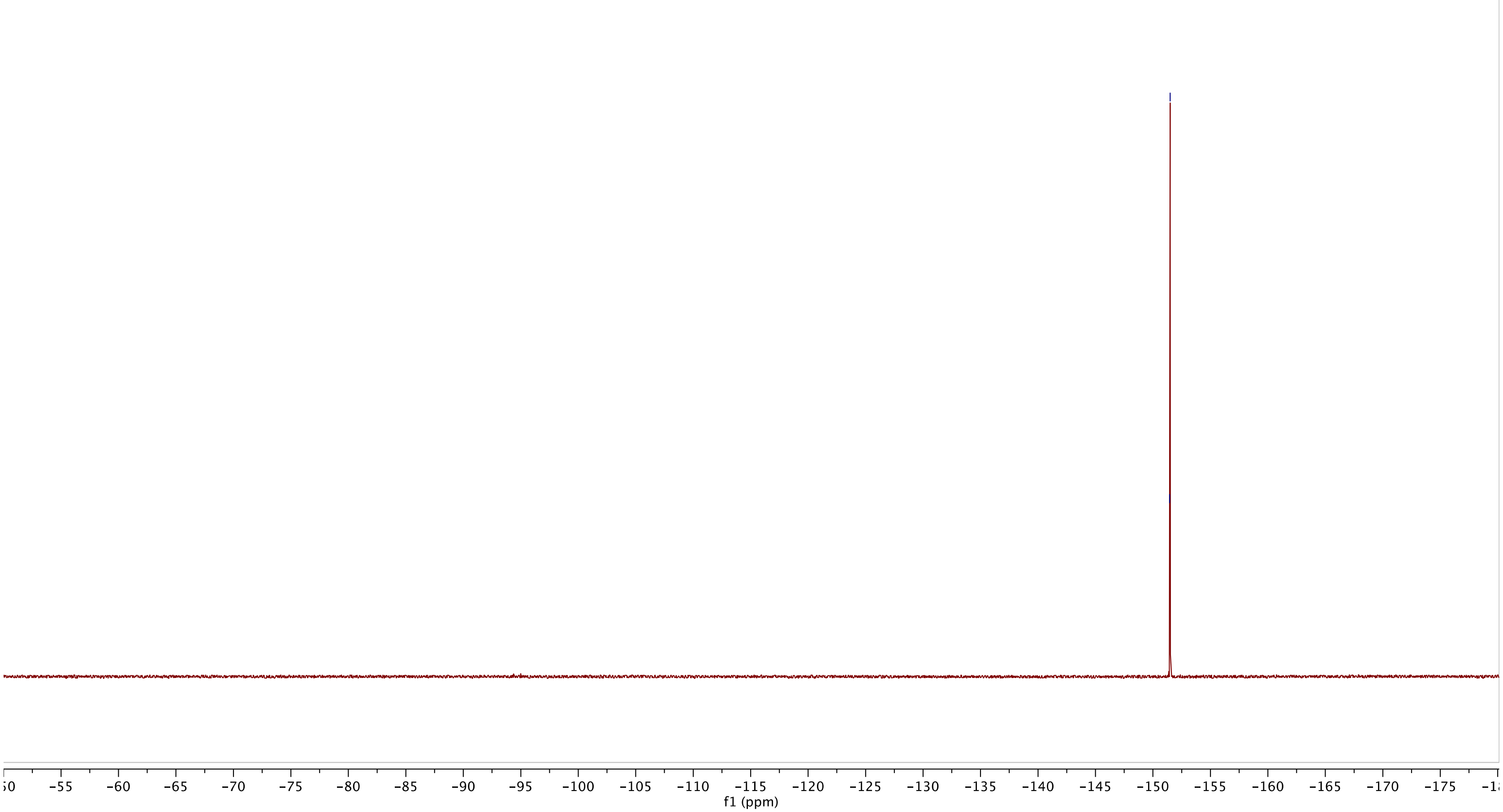

_N,N,N_-trimethyl-benzo[h]quinolin-2-aminium tetrafluoroborate (10)

^{1}H NMR (600 MHz, CD_3CN)

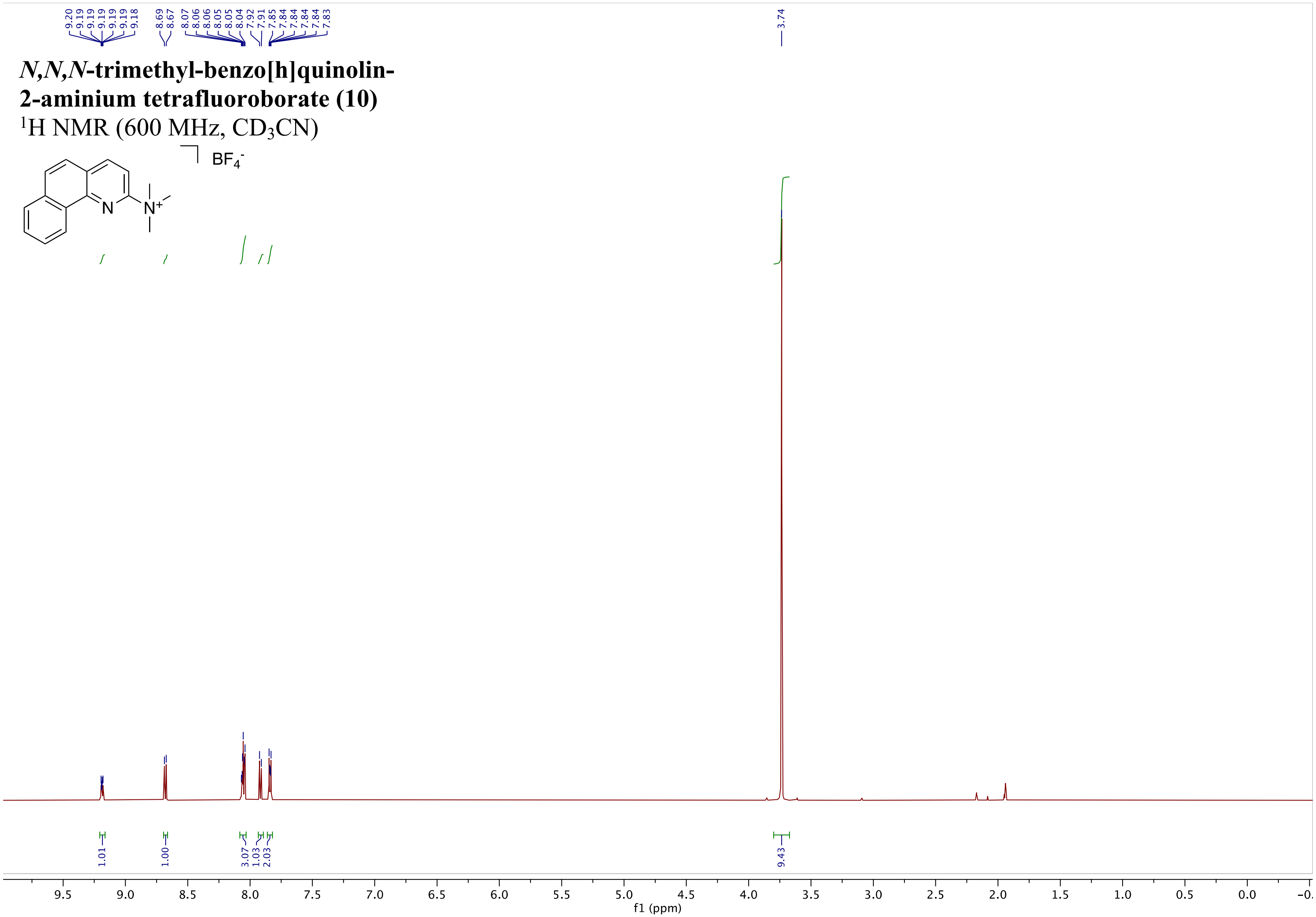

***N,N,N*-trimethyl-benzo[h]quinolin-2-aminium tetrafluoroborate (10)**

^{13}C NMR (151 MHz, CD_3CN)

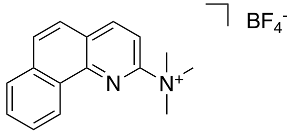

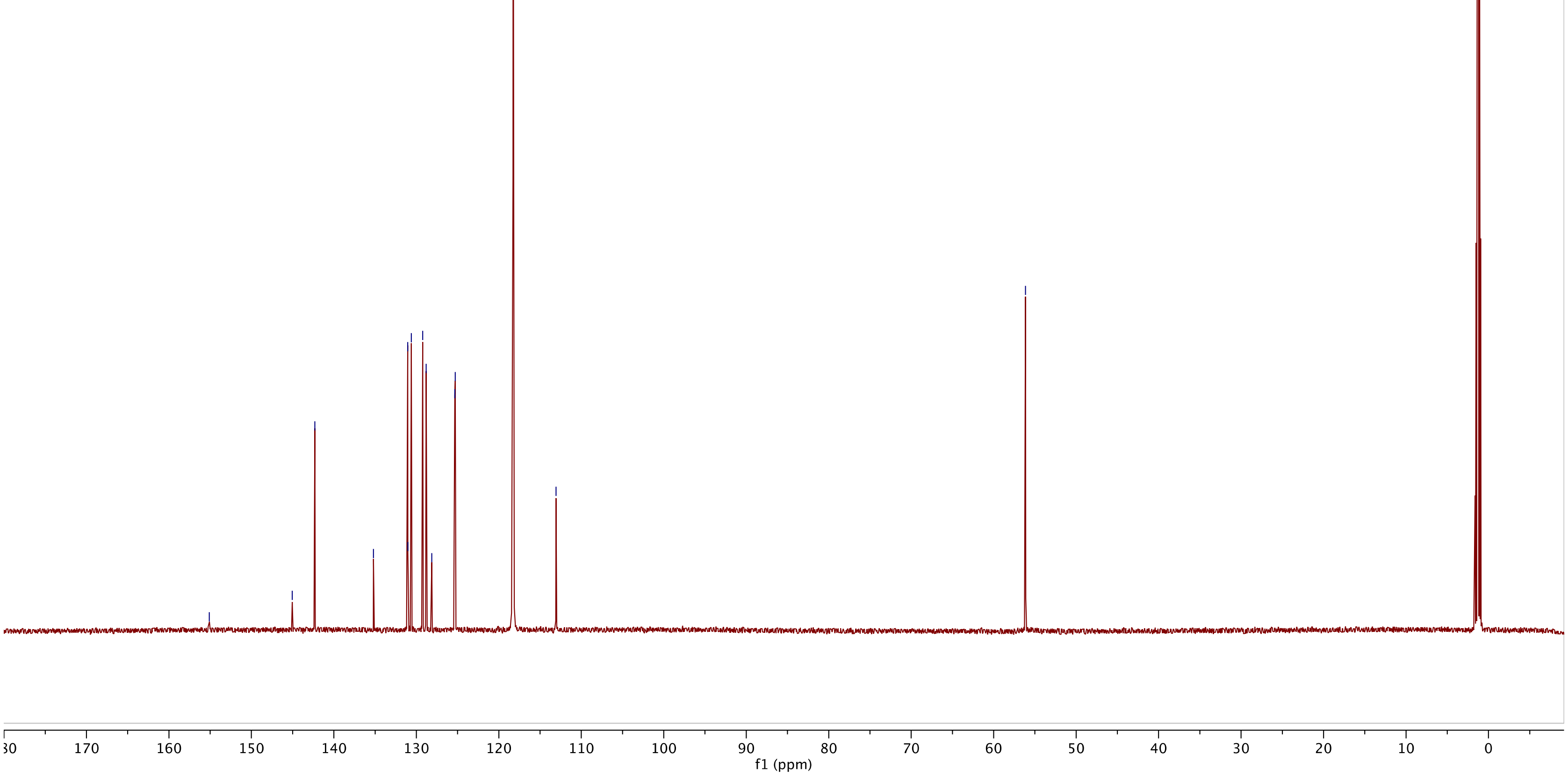

***N,N,N*-trimethyl-benzo[h]quinolin-2-aminium tetrafluoroborate (10)**

^{19}F NMR (376 MHz, CD_3CN)

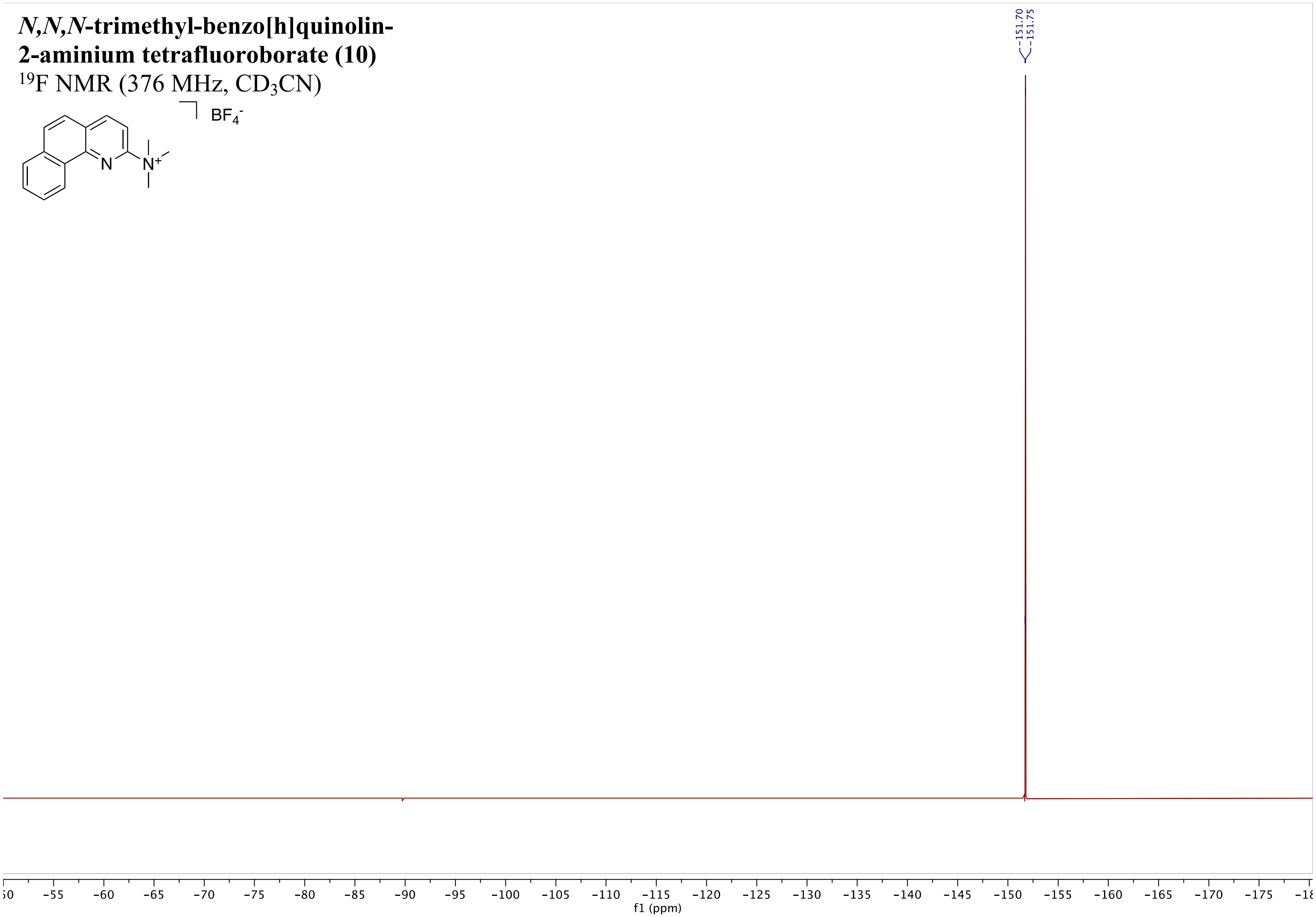

_N,N,N,N'',N'',N''_-hexamethyl-2,2':6',2"-terpyridin-6,6''-diaminium bis(tetrafluoroborate) (11)

^{1}H NMR (600 MHz, DMSO-d_6)

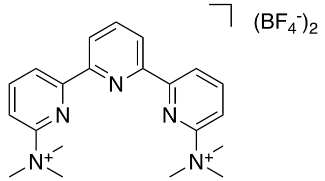

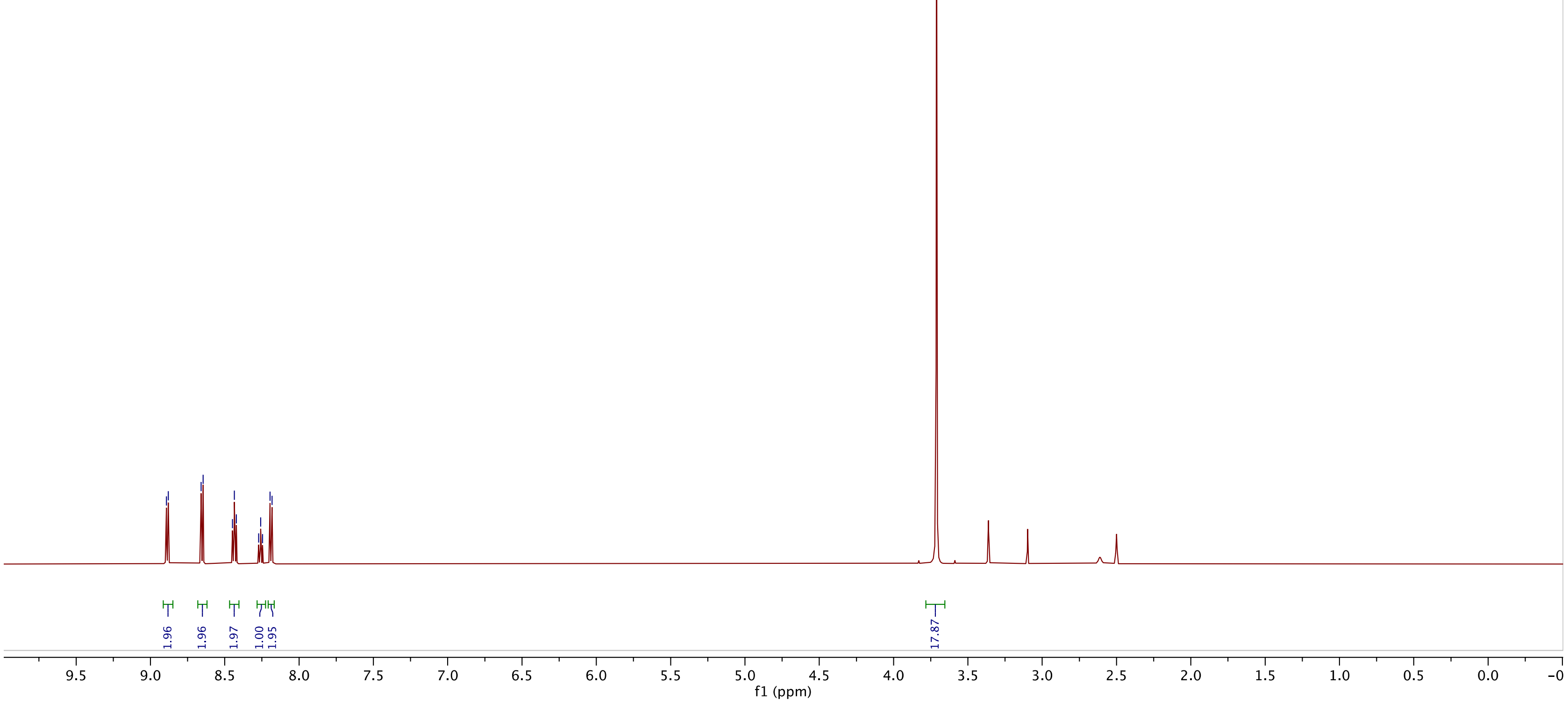

***N,N,N,N'',N'',N''*-hexamethyl-2,2':6',2”-terpyridin-6,6''-diaminium bis(tetrafluoroborate) (11)**

^{13}C NMR (151 MHz, DMSO-d_6)

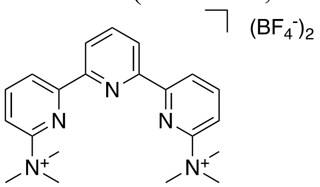

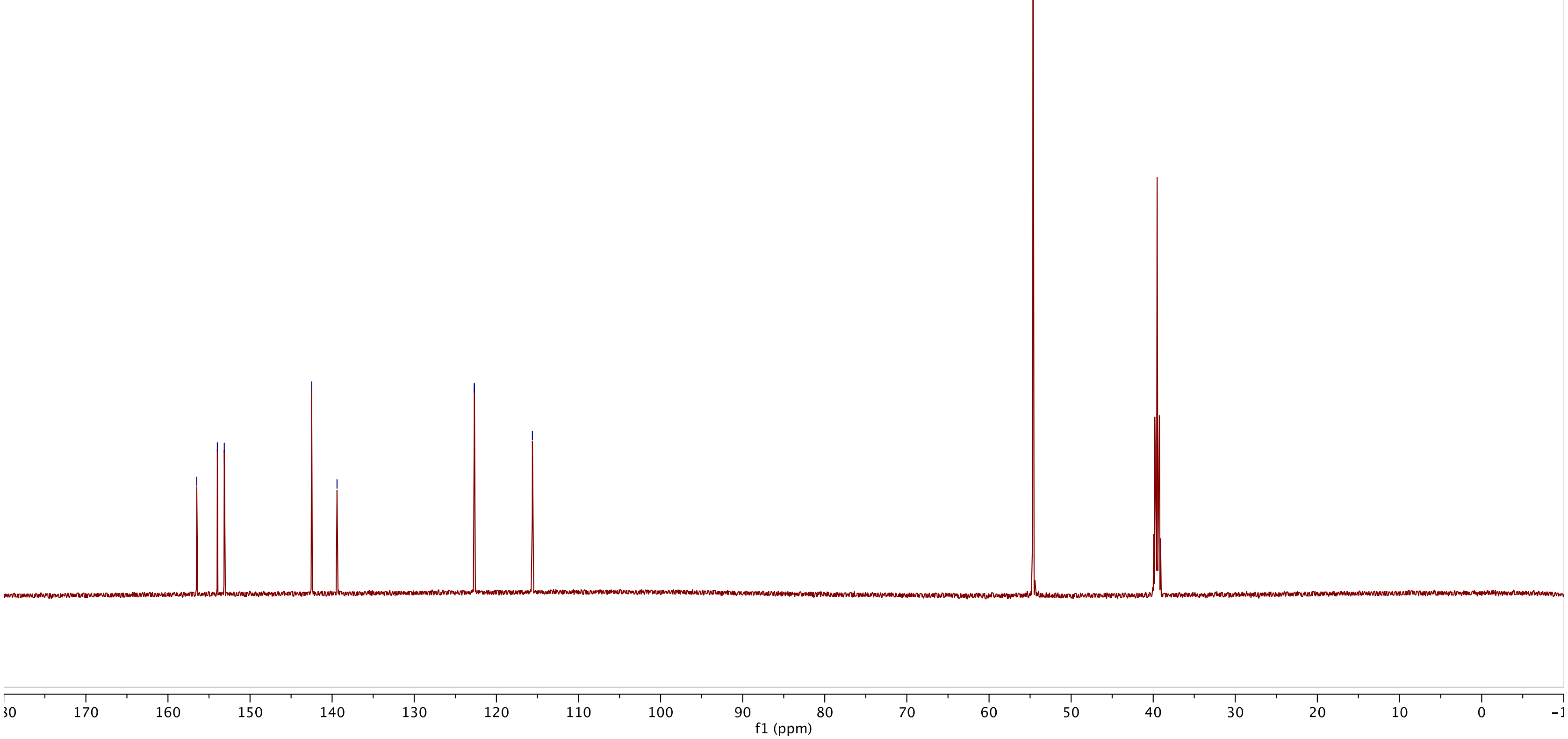

***N,N,N,N'',N'',N''*-hexamethyl-2,2':6',2"-terpyridin-6,6''-diaminium bis(tetrafluoroborate) (11)**

^{19}F NMR (376 MHz, DMSO-d$_6$)

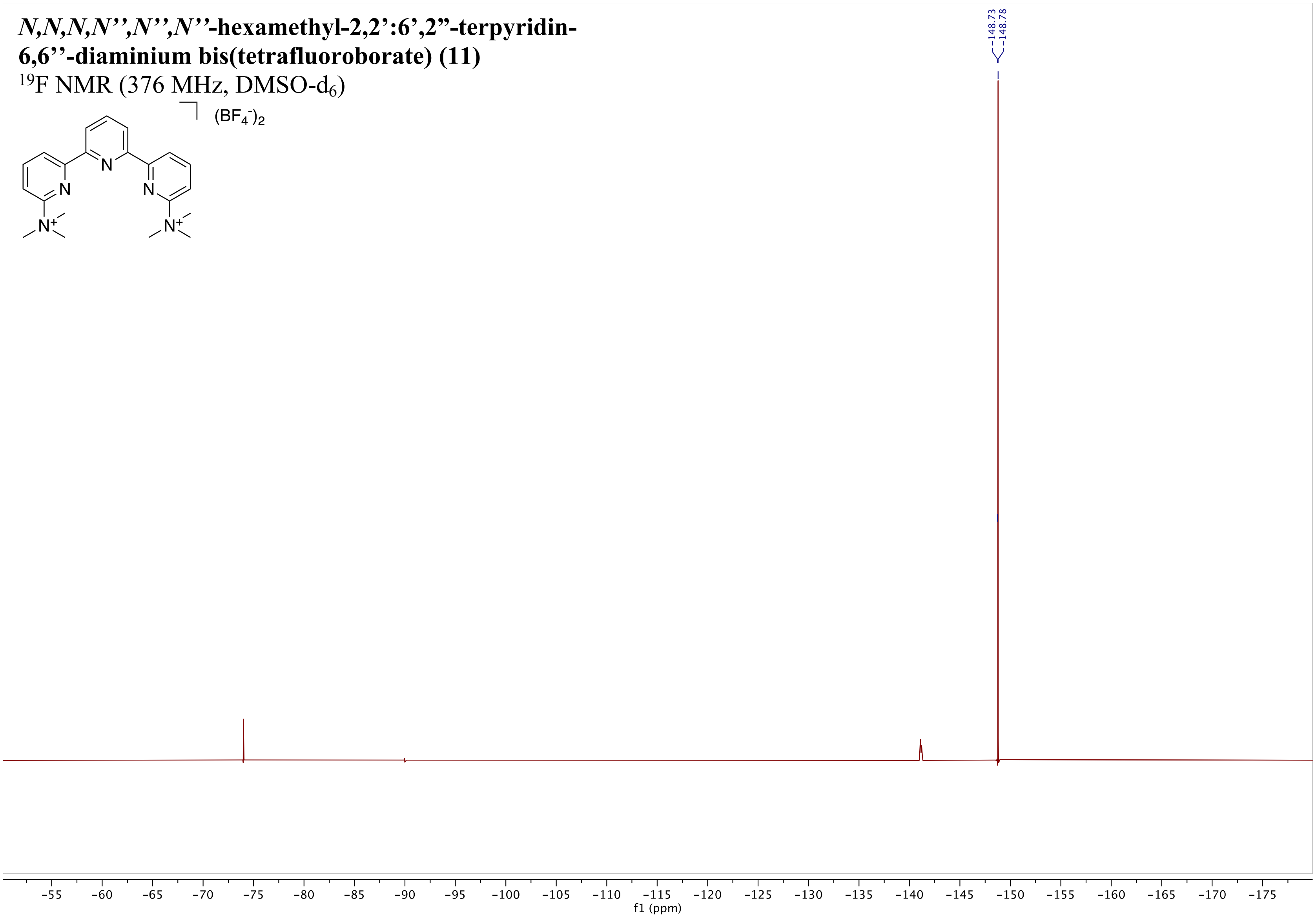

***N,N,N,N''',N''',N'''*-hexamethyl-2,2':6',2'':6'',2'''-quaterpyridin-6,6'''-diaminium bis(tetrafluoroborate) (12)**

^{1}H NMR (600 MHz, CD_3CN)

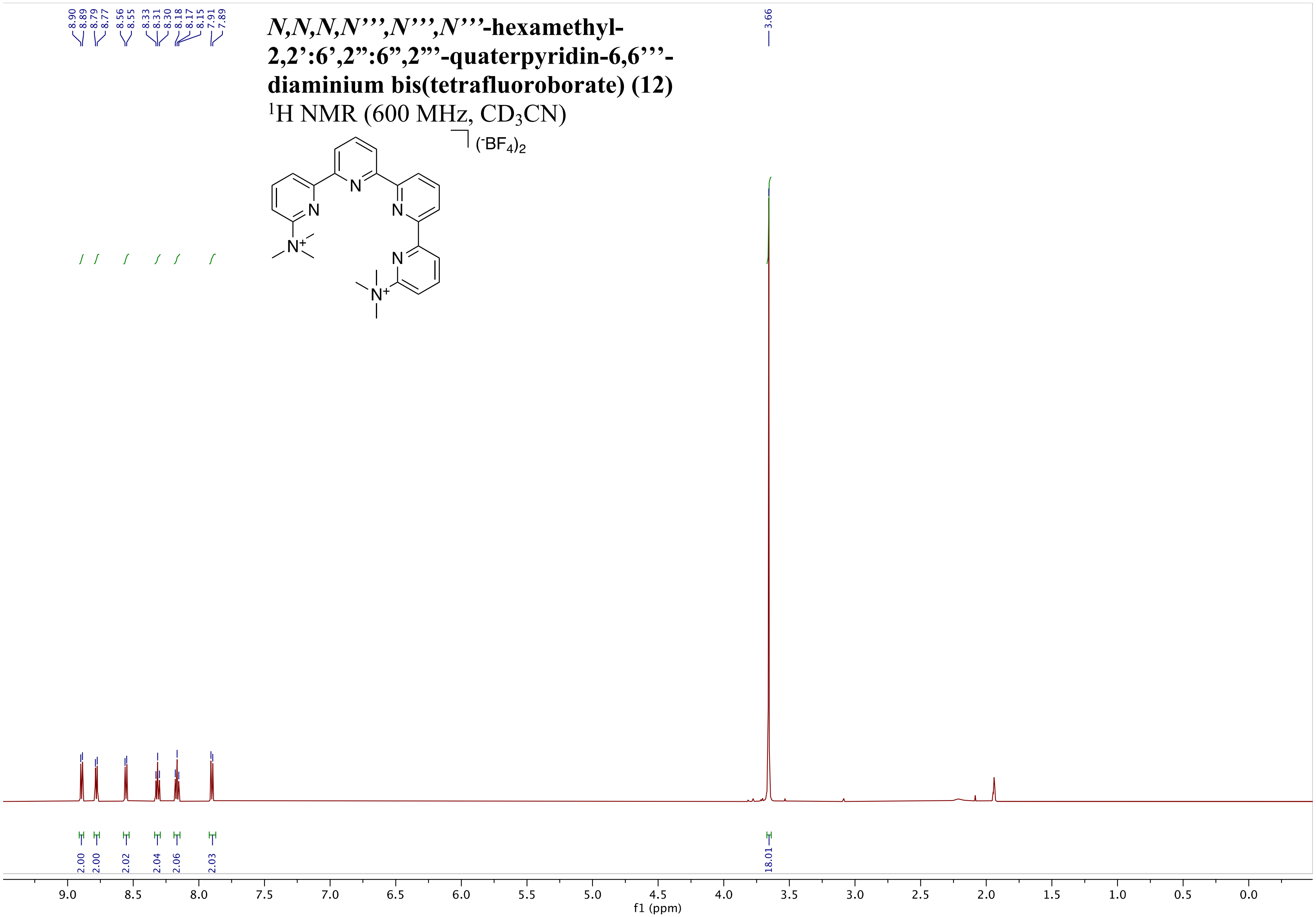

***N,N,N,N''',N''',N'''*-hexamethyl-2,2':6',2":6",2"'-quaterpyridin-6,6'''-diaminium bis(tetrafluoroborate) (12)**

^{13}C NMR (151 MHz, CD_3CN)

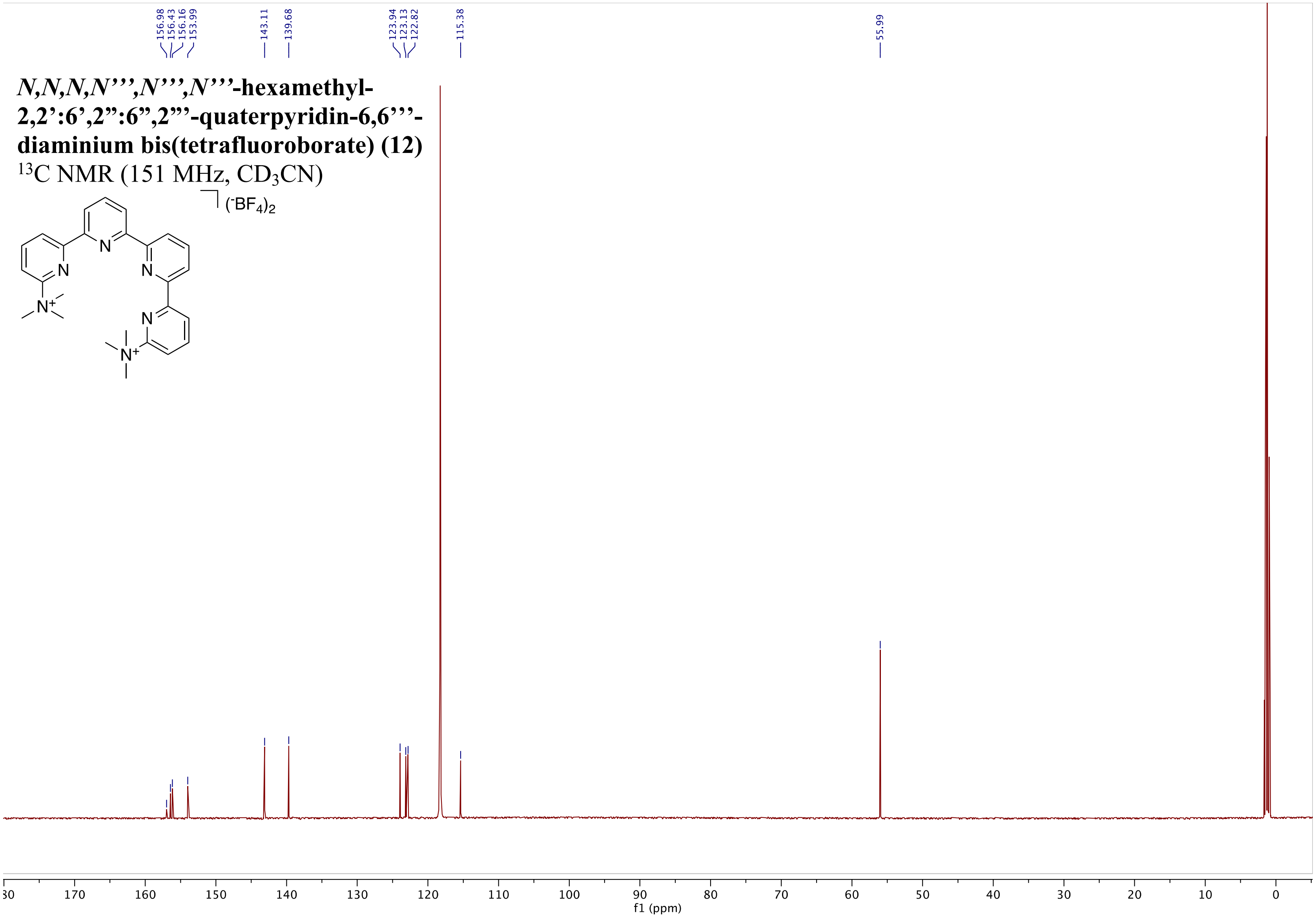

***N,N,N,N''',N''',N'''*-hexamethyl-2,2':6',2":6",2"'-quaterpyridin-6,6'''-diaminium bis(tetrafluoroborate) (12)**

^{19}F NMR (376 MHz, CD_3CN)

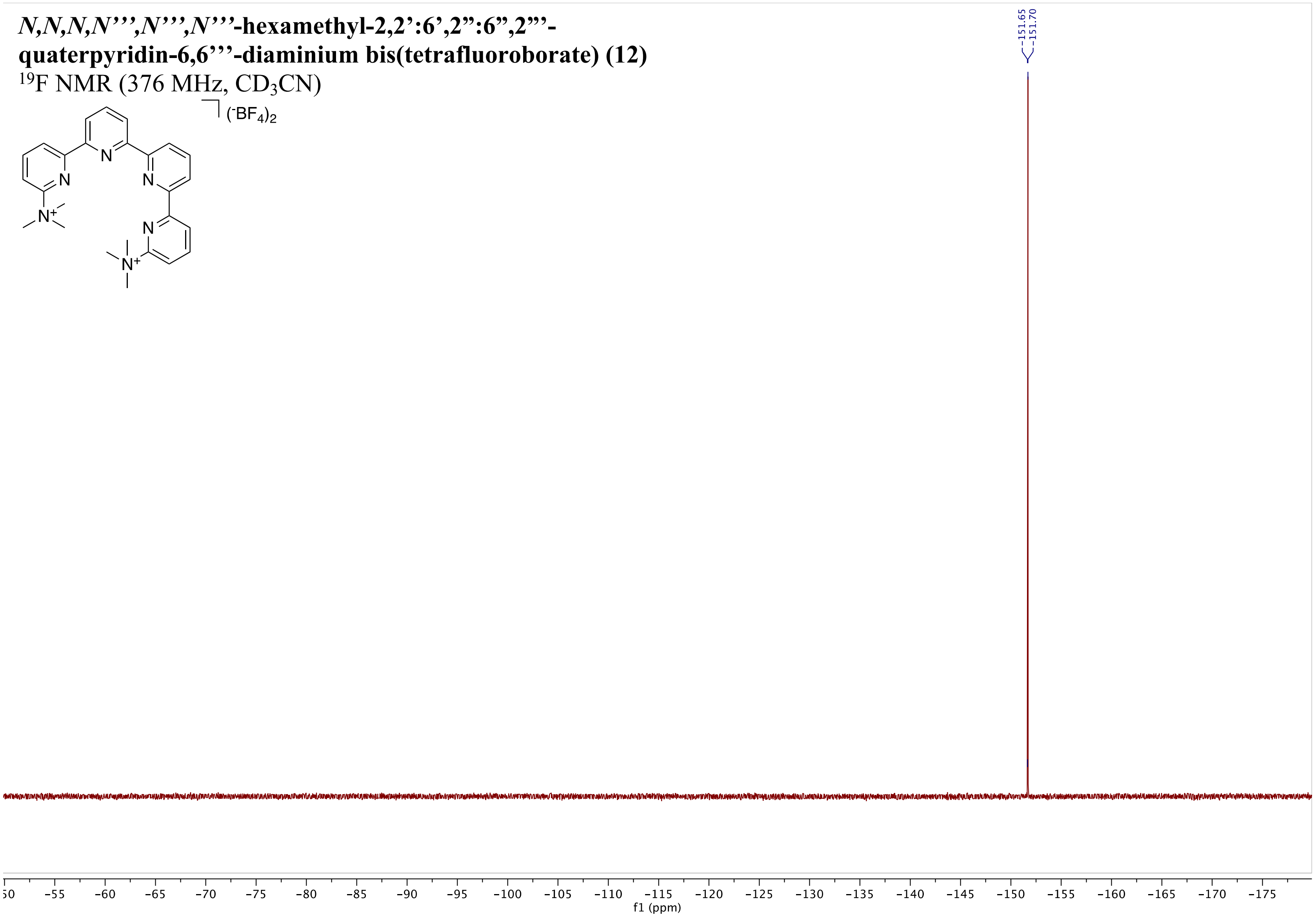

***P,P,P,P',P',P'*-hexa(*p*-tolyl)-4,4'-dimethyl-2,2'-bipyridin-6,6'-diphosphinium bis(trifluoroacetate) (13)**

^{1}H NMR (600 MHz, CD_3OD)

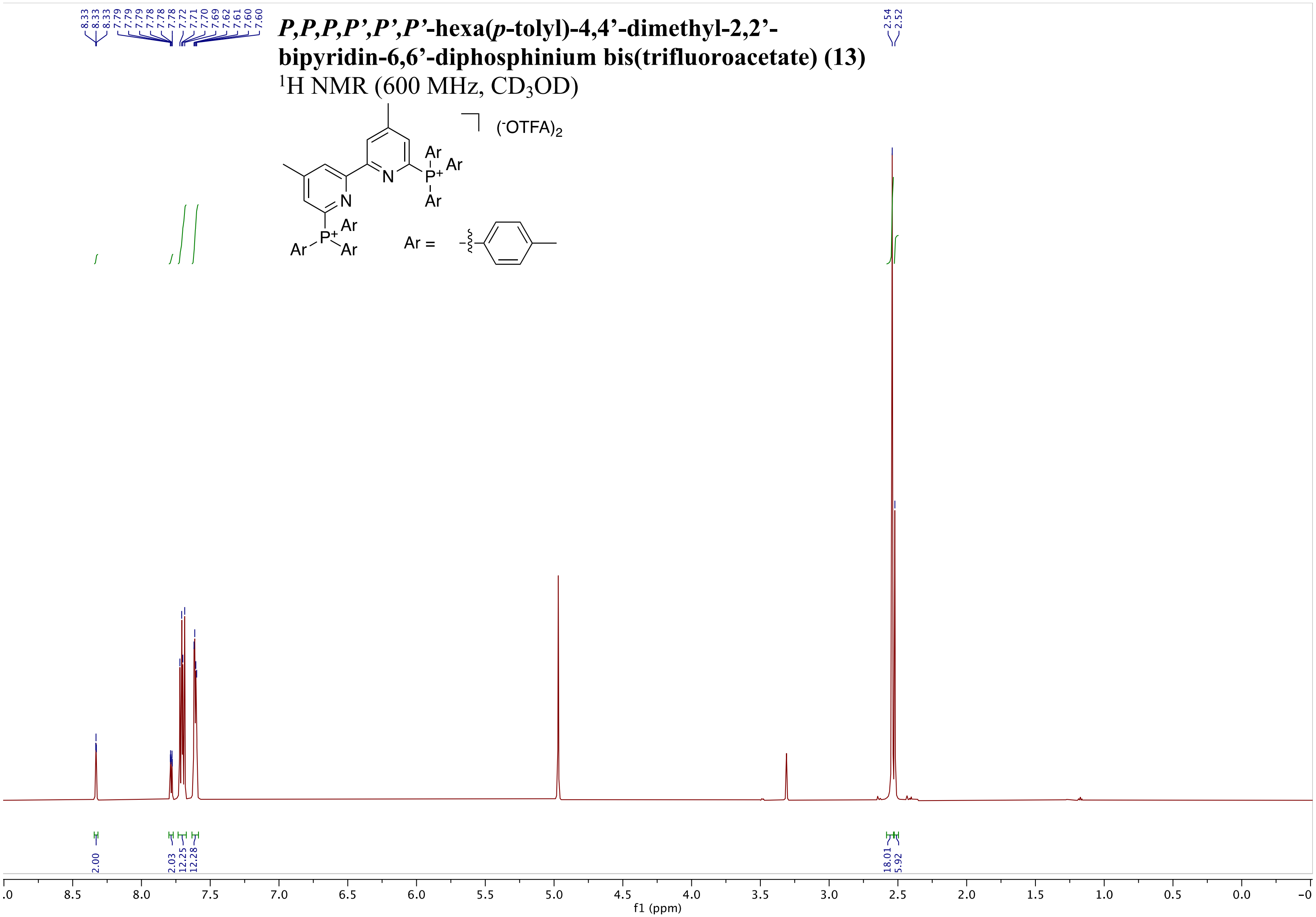

***P,P,P,P',P',P'*-hexa(*p*-tolyl)-4,4'-dimethyl-2,2'-bipyridin-6,6'-diphosphinium bis(trifluoroacetate) (13)**

^{13}C NMR (151 MHz, CD_3OD)

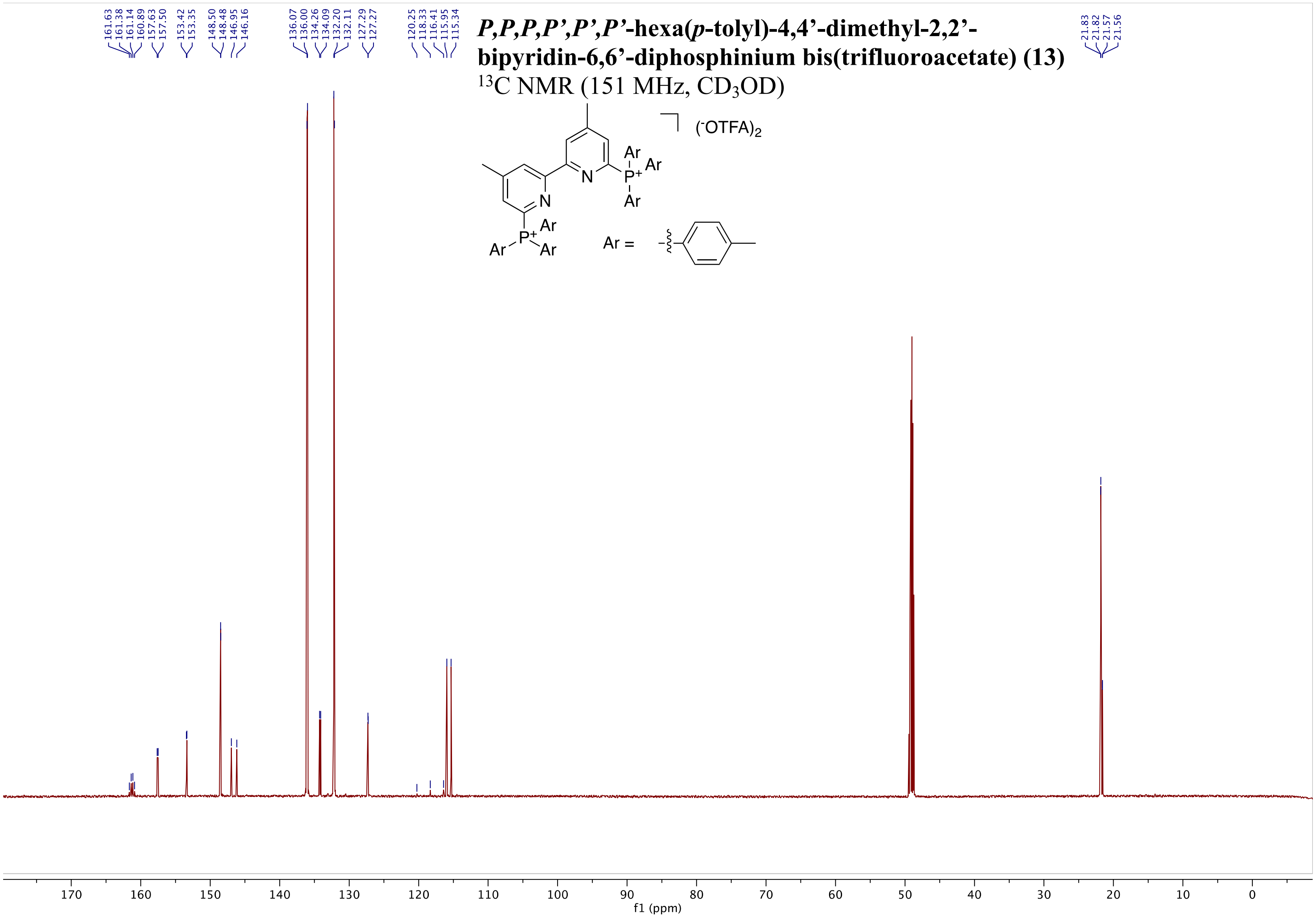

***P,P,P,P’,P’,P’*-hexa(*p*-tolyl)-4,4’-dimethyl-2,2’-bipyridin-6,6’-diphosphinium bis(trifluoroacetate) (13)**

^{19}F NMR (376 MHz, CD_3OD)

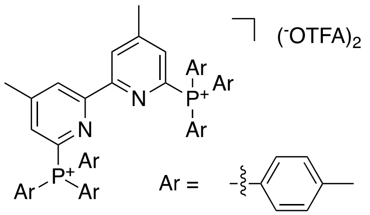

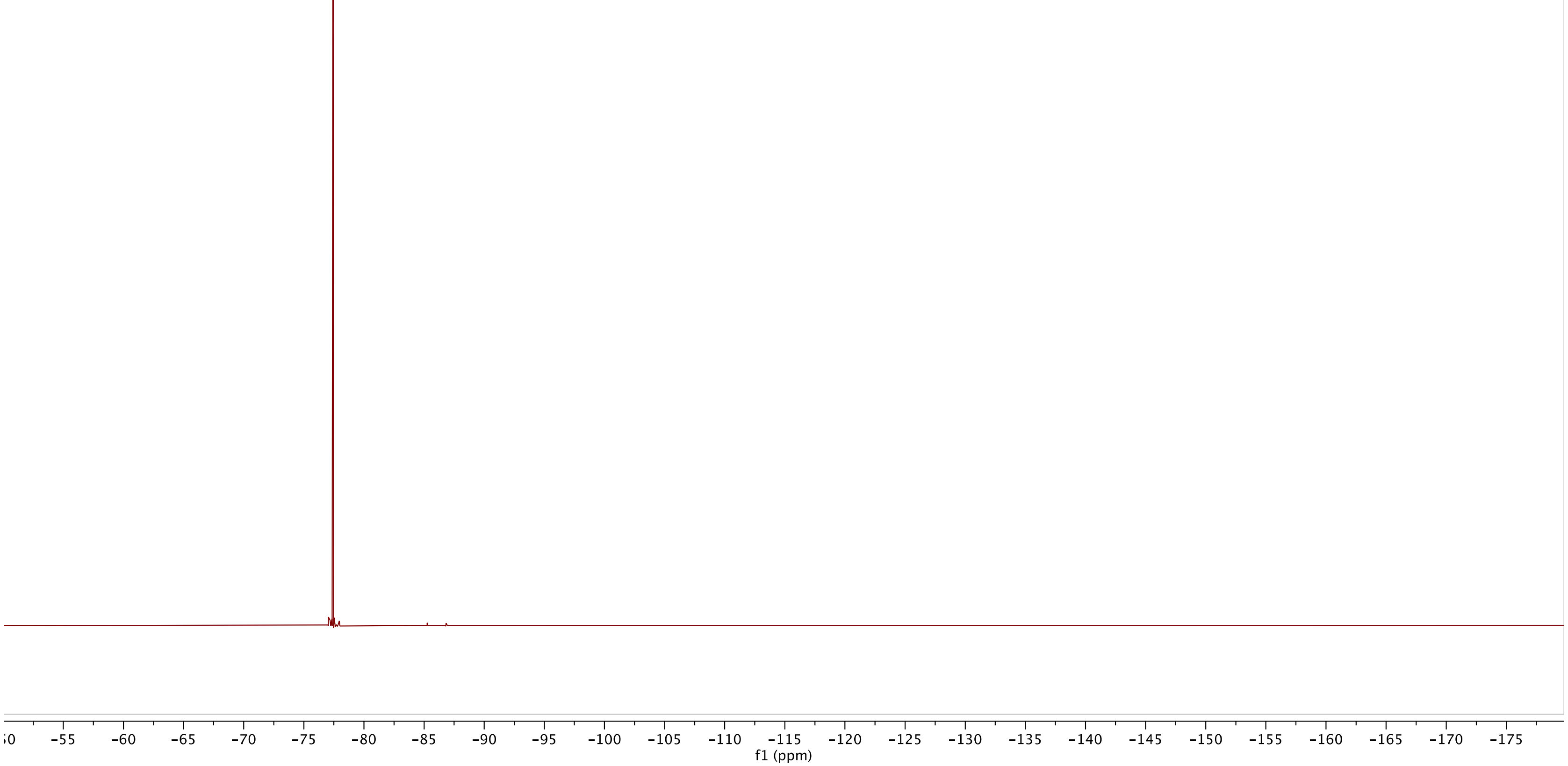

_P,P,P,P',P',P'_-hexa(_p_-tolyl)-4,4'-dimethyl-2,2'-bipyridin-6,6'-diphosphinium bis(trifluoroacetate) (13)

^{31}P NMR (162 MHz, CD_3OD)

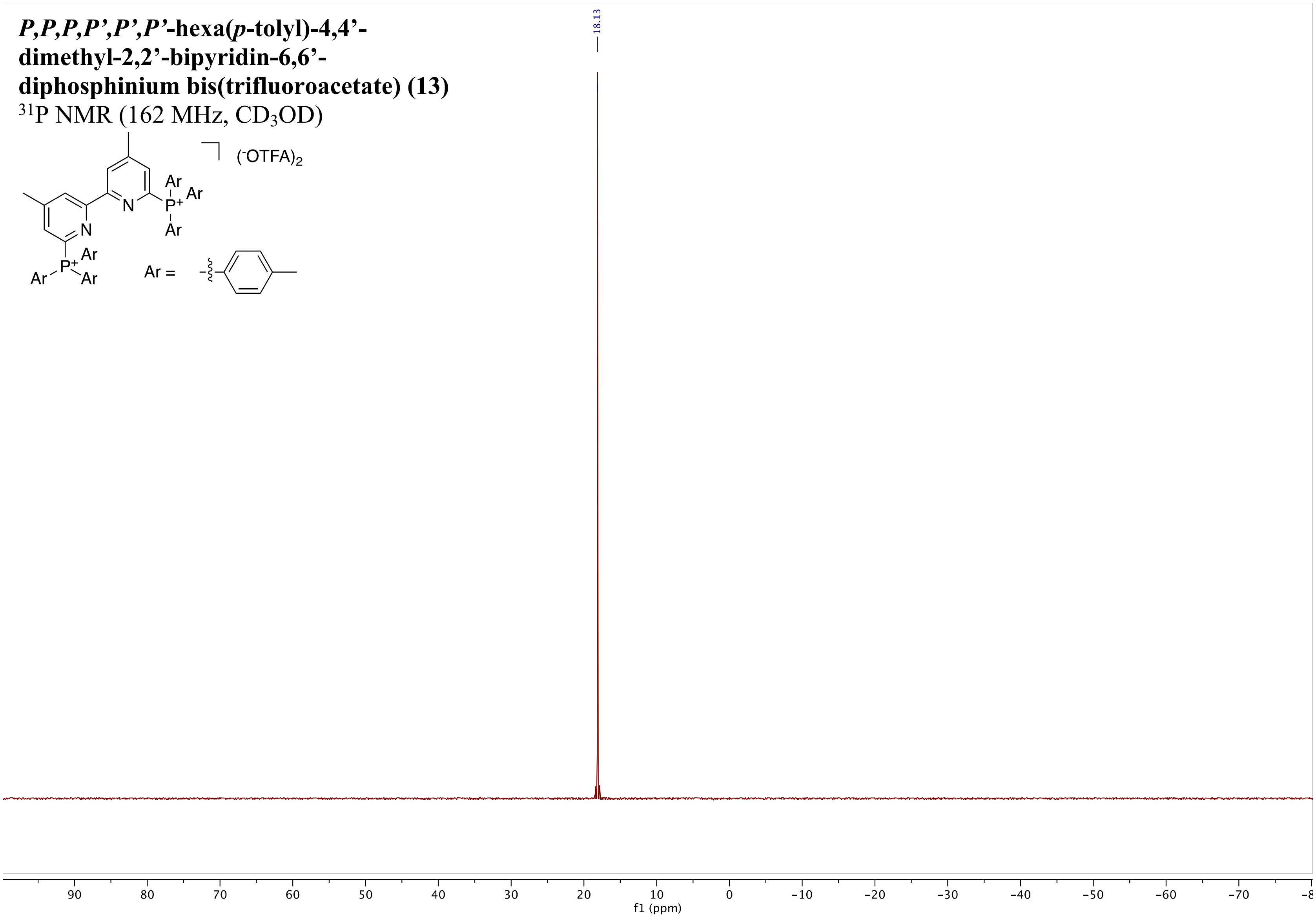

***P,P,P,P’,P’,P’*-hexa(*p*-tolyl)-6,6’-dimethyl-2,2’-bipyridin-4,4’-diphosphinium bis(trifluoroacetate) (14)**

^{1}H NMR (600 MHz, CD_3OD)

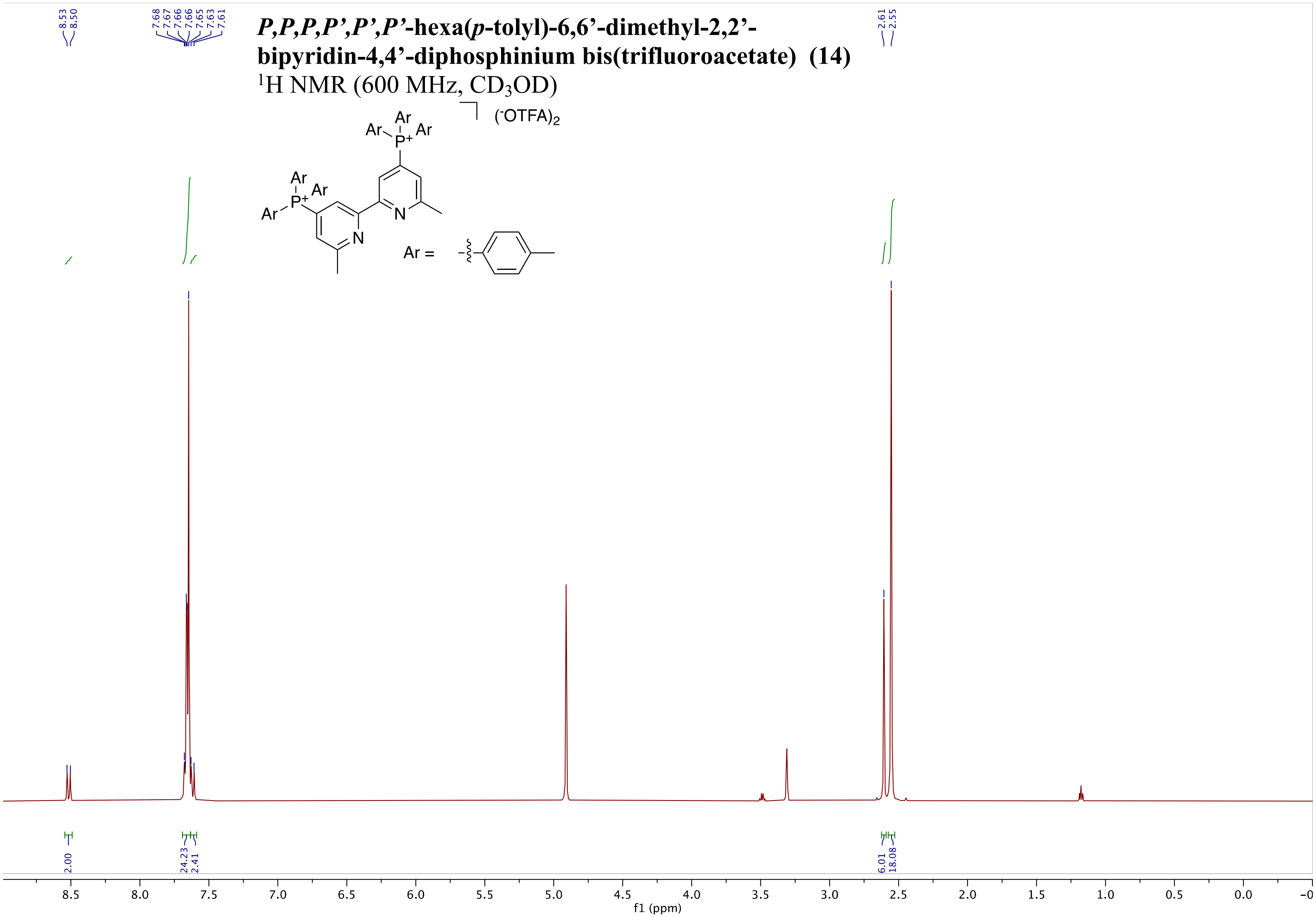

***P,P,P,P',P',P'*-hexa(*p*-tolyl)-6,6'-dimethyl-2,2'-bipyridin-4,4'-diphosphinium bis(trifluoroacetate) (14)**

^{13}C NMR (151 MHz, CD_3OD)

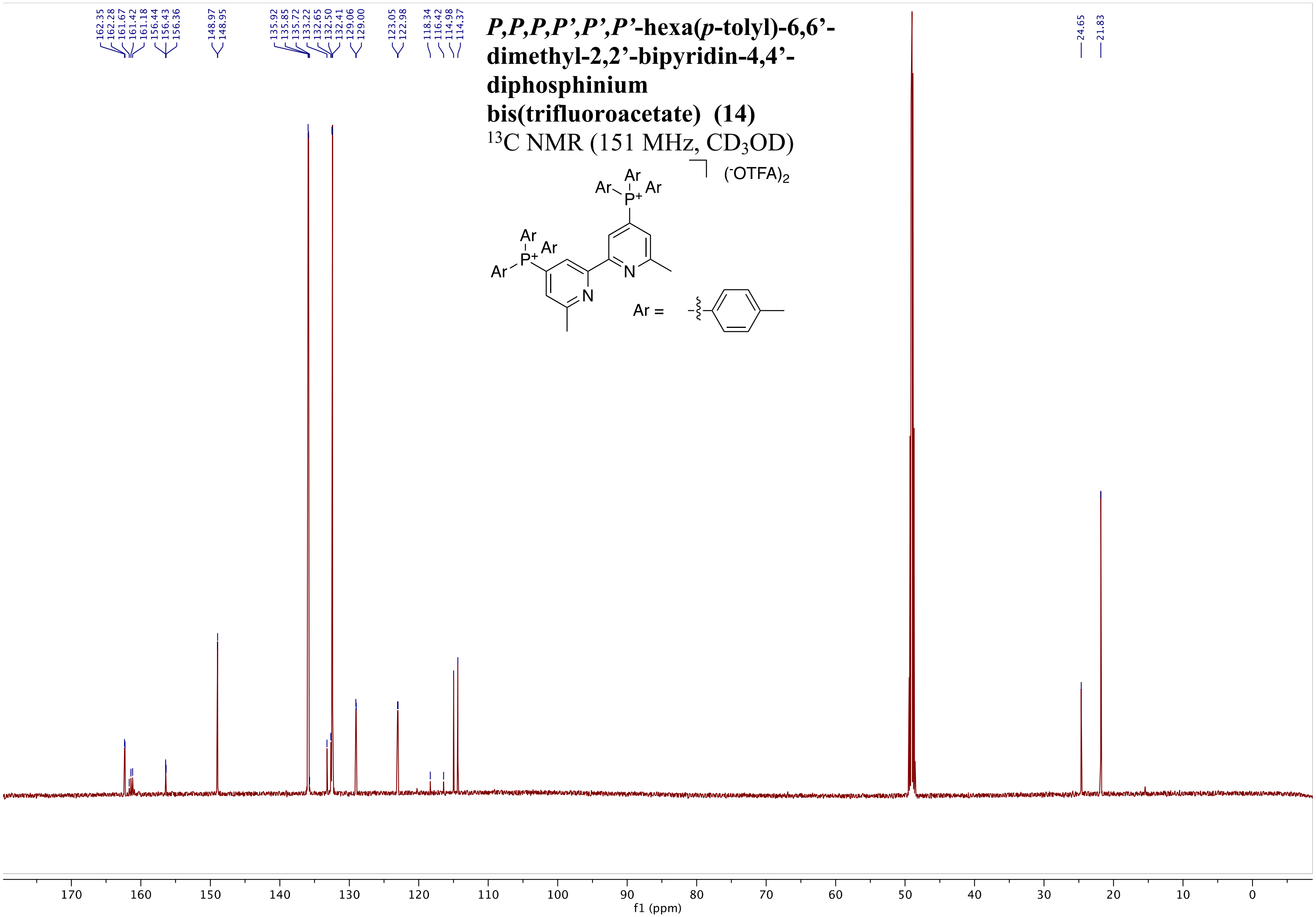

***P,P,P,P',P',P'*-hexa(*p*-tolyl)-6,6'-dimethyl-2,2'-bipyridin-4,4'-diphosphinium bis(trifluoroacetate) (14)**

^{19}F NMR (376 MHz, CD_3OD)

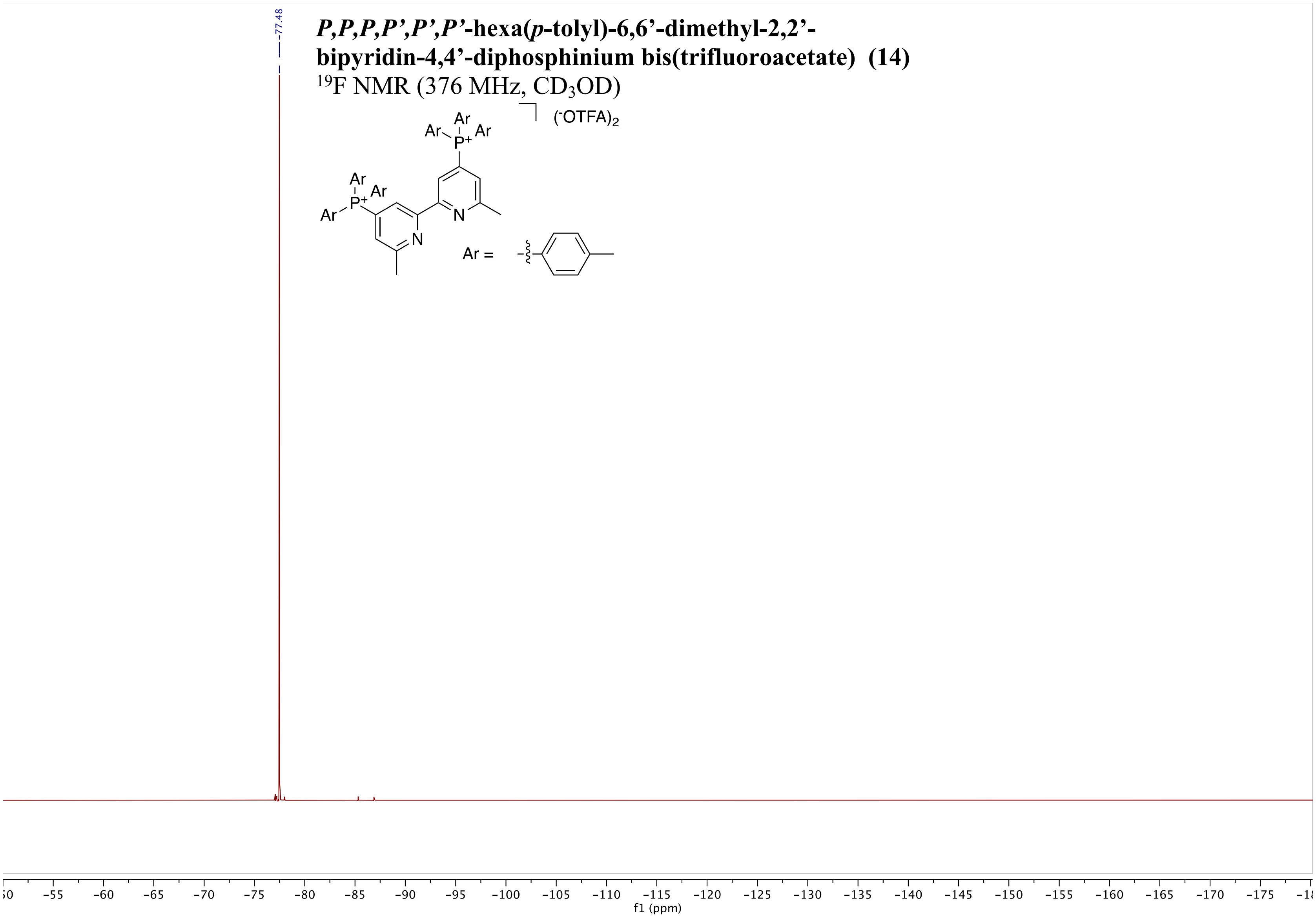

***P,P,P,P',P',P'*-hexa(*p*-tolyl)-6,6'-dimethyl-2,2'-bipyridin-4,4'-diphosphinium bis(trifluoroacetate) (14)**

^{31}P NMR (162 MHz, CD_3OD)

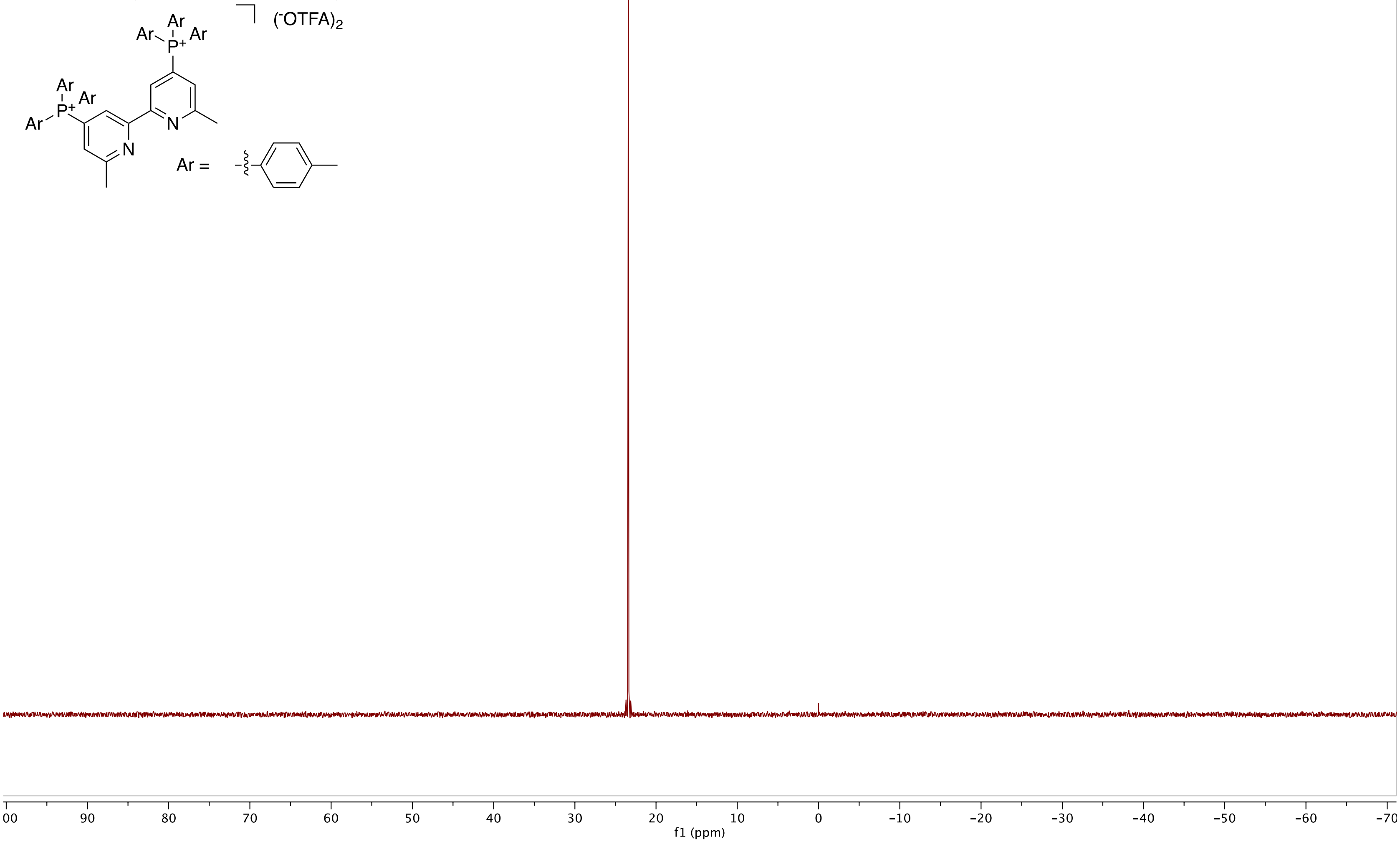

_P,P,P,P',P',P'_-hexaphenyl-4,4'-dimethoxy-2,2'-bipyridin-6,6'-diphosphinium bis(trifluoroacetate) (15)
^1H NMR (600 MHz, CD_3OD)

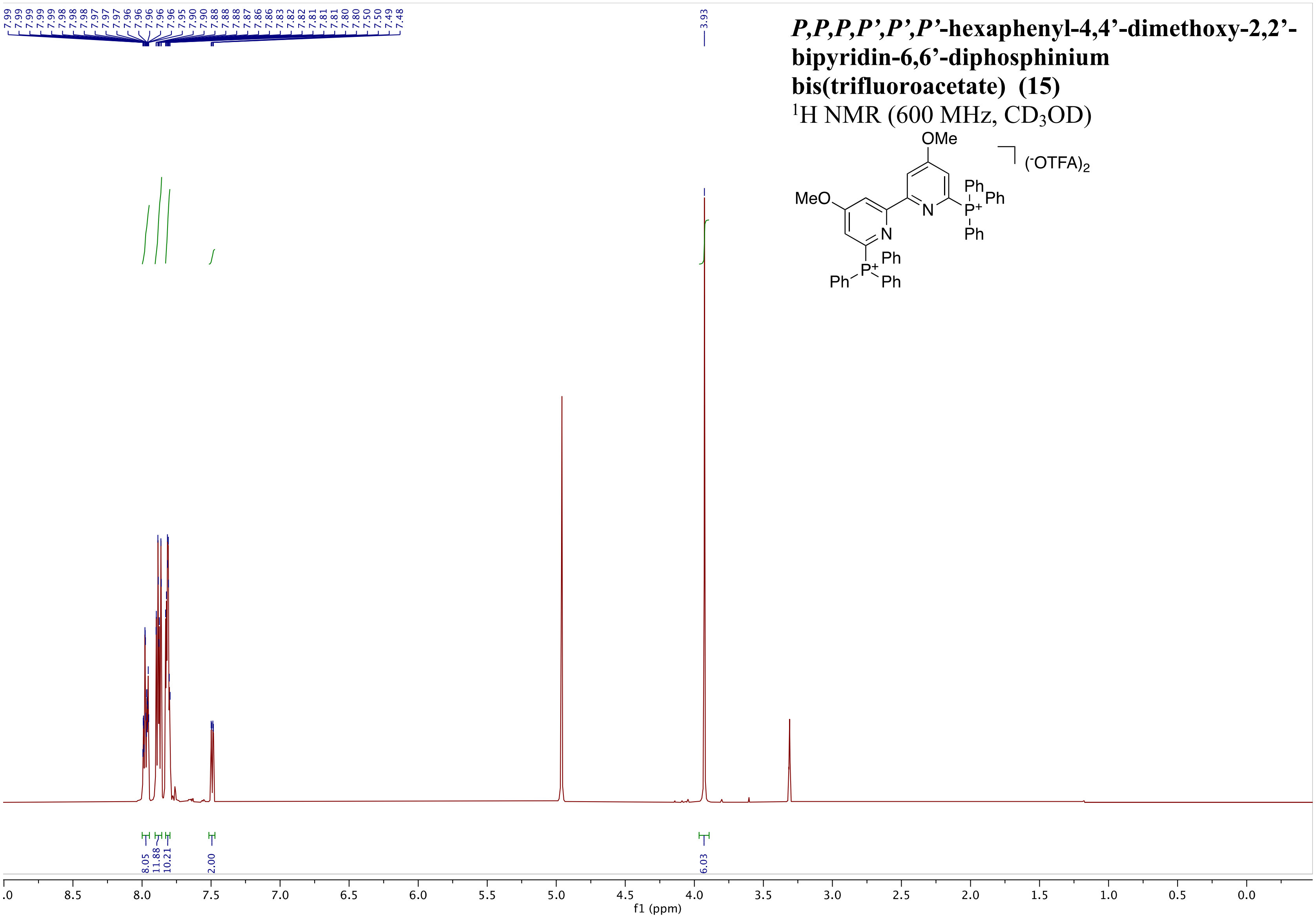

_P,P,P,P',P',P'_-hexaphenyl-4,4'-dimethoxy-2,2'-bipyridin-6,6'-diphosphinium bis(trifluoroacetate) (15)

^{13}C NMR (151 MHz, CD_3OD)

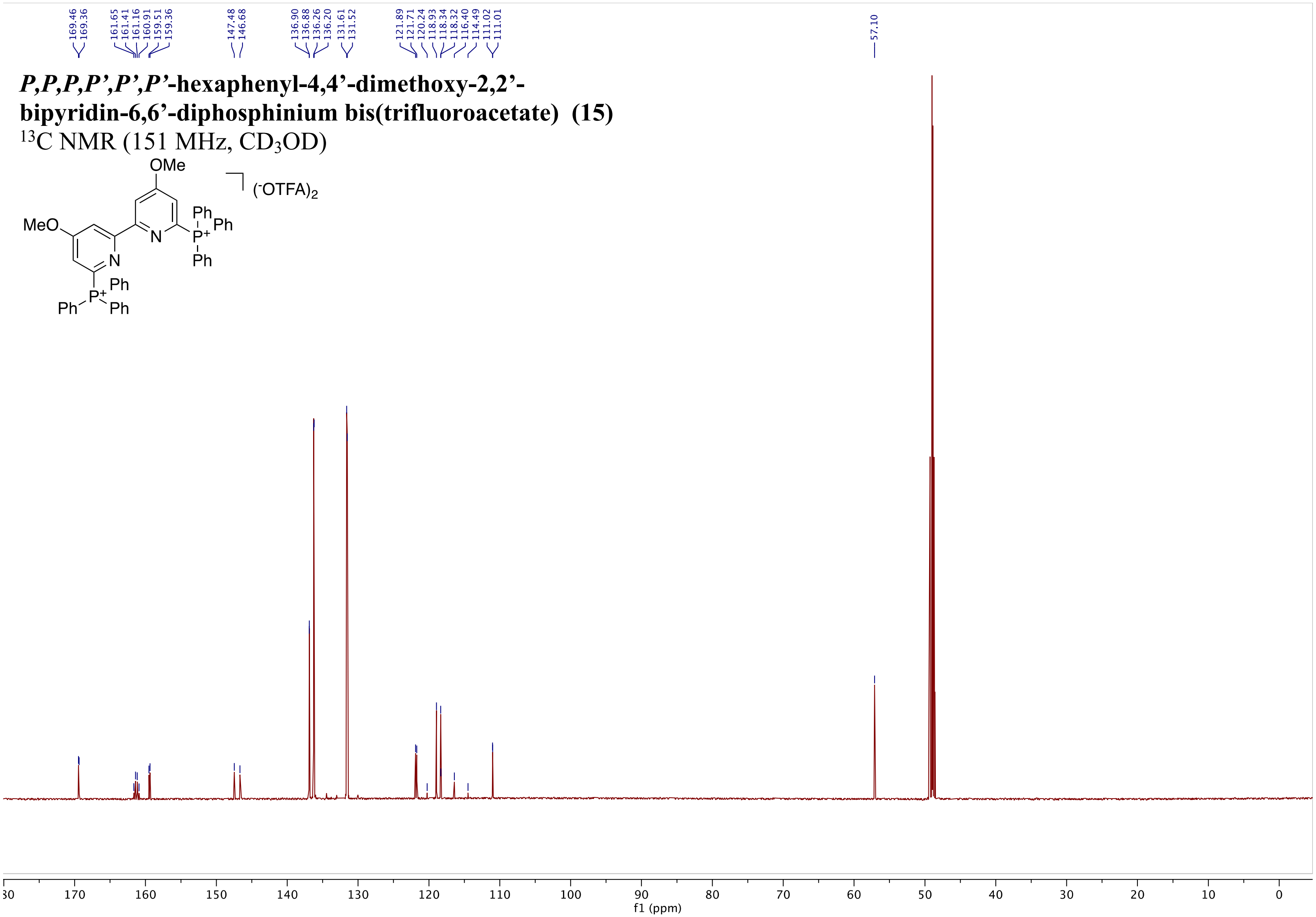

***P,P,P,P',P',P'*-hexaphenyl-4,4'-dimethoxy-2,2'-bipyridin-6,6'-diphosphinium bis(trifluoroacetate) (15)**

^{19}F NMR (376 MHz, CD_3OD)

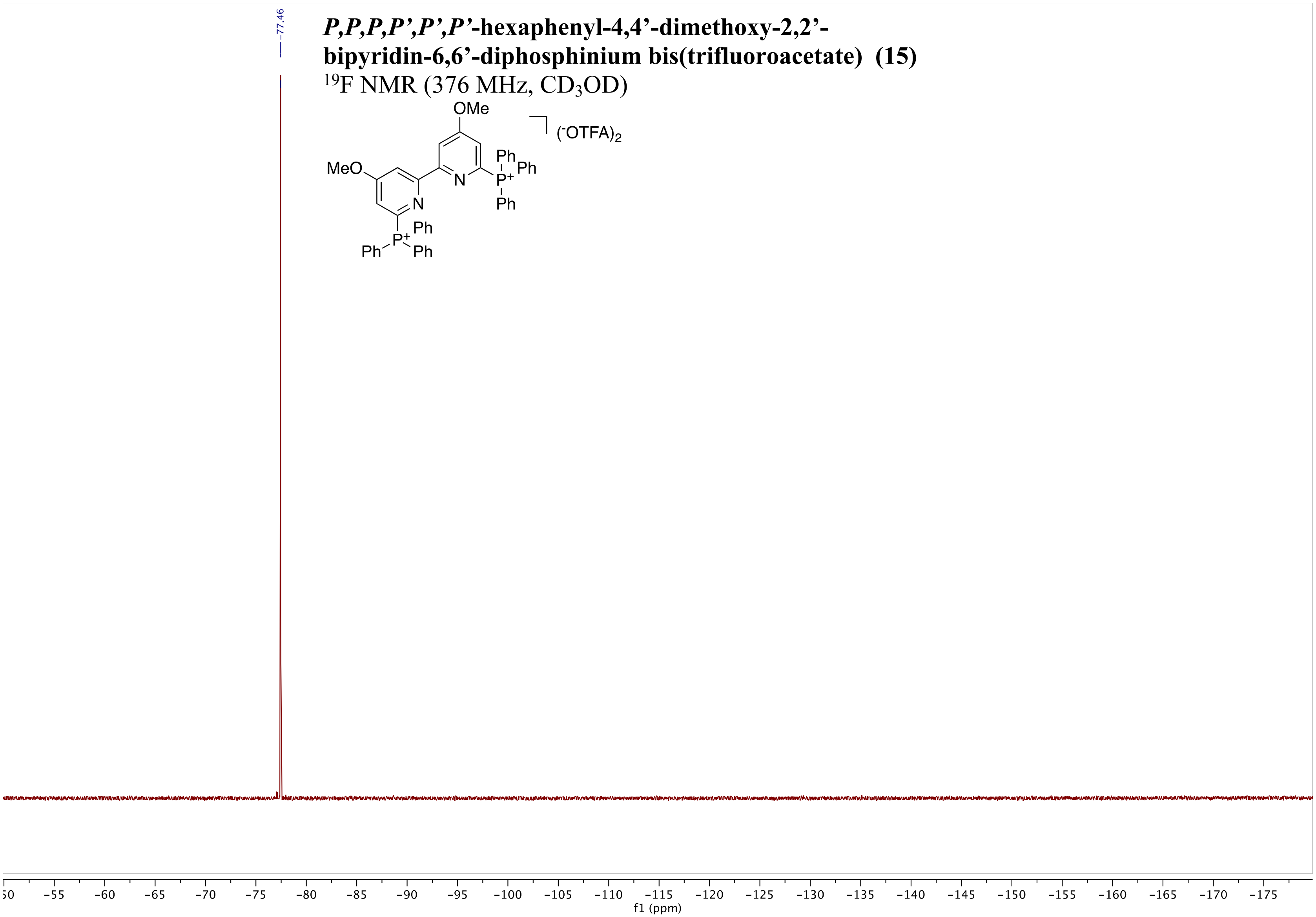

***P,P,P,P',P',P'*-hexaphenyl-4,4'-dimethoxy-2,2'-bipyridin-6,6'-diphosphinium bis(trifluoroacetate) (15)**

^{31}P NMR (162 MHz, CD_3OD)

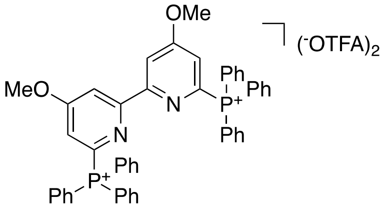

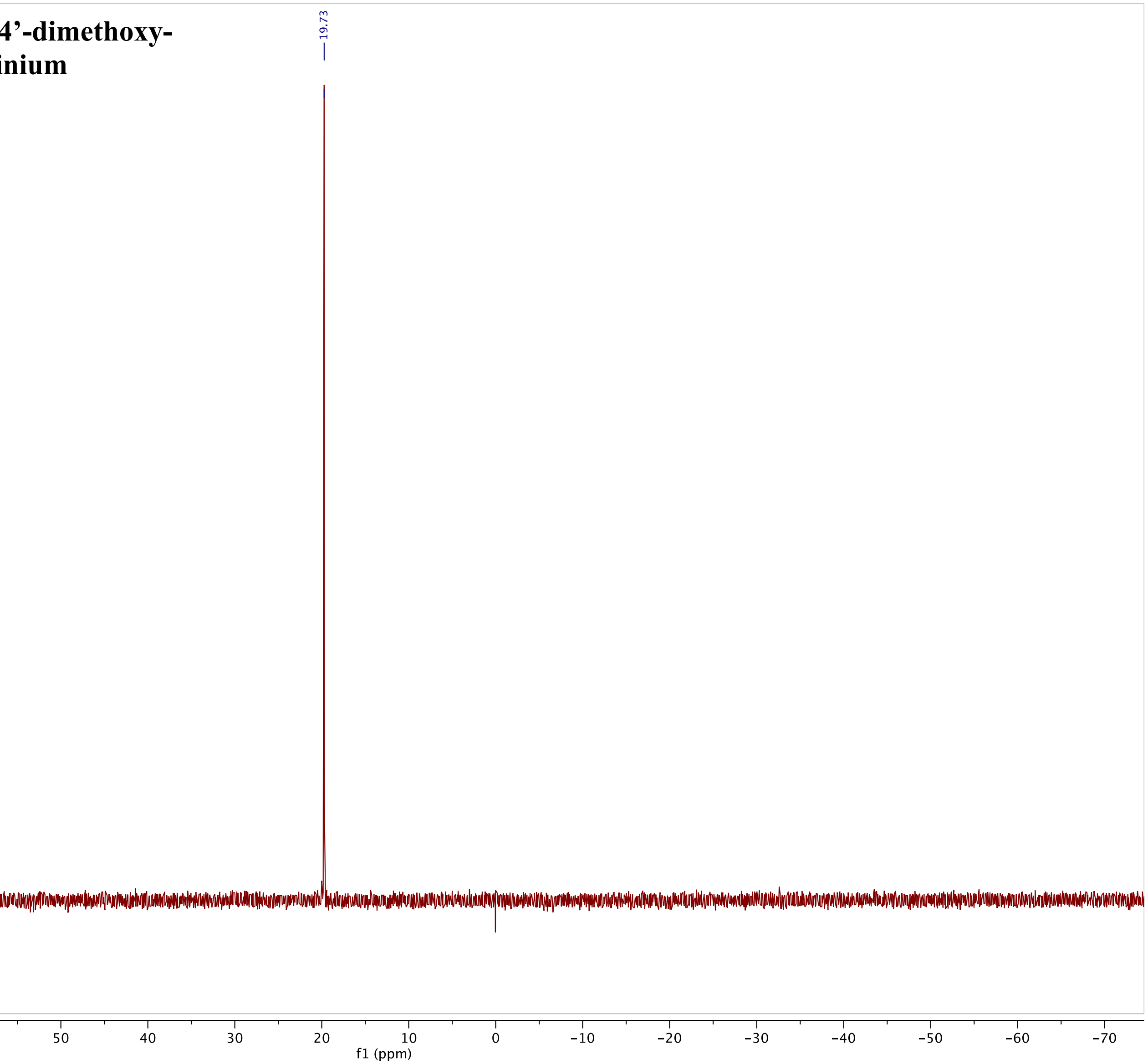

***P,P,P*-triphenyl-4,4'-dimethyl-2,2'-bipyridin-6-phosphinium trifluoroacetate (16)**

^{1}H NMR (600 MHz, CD_3OD)

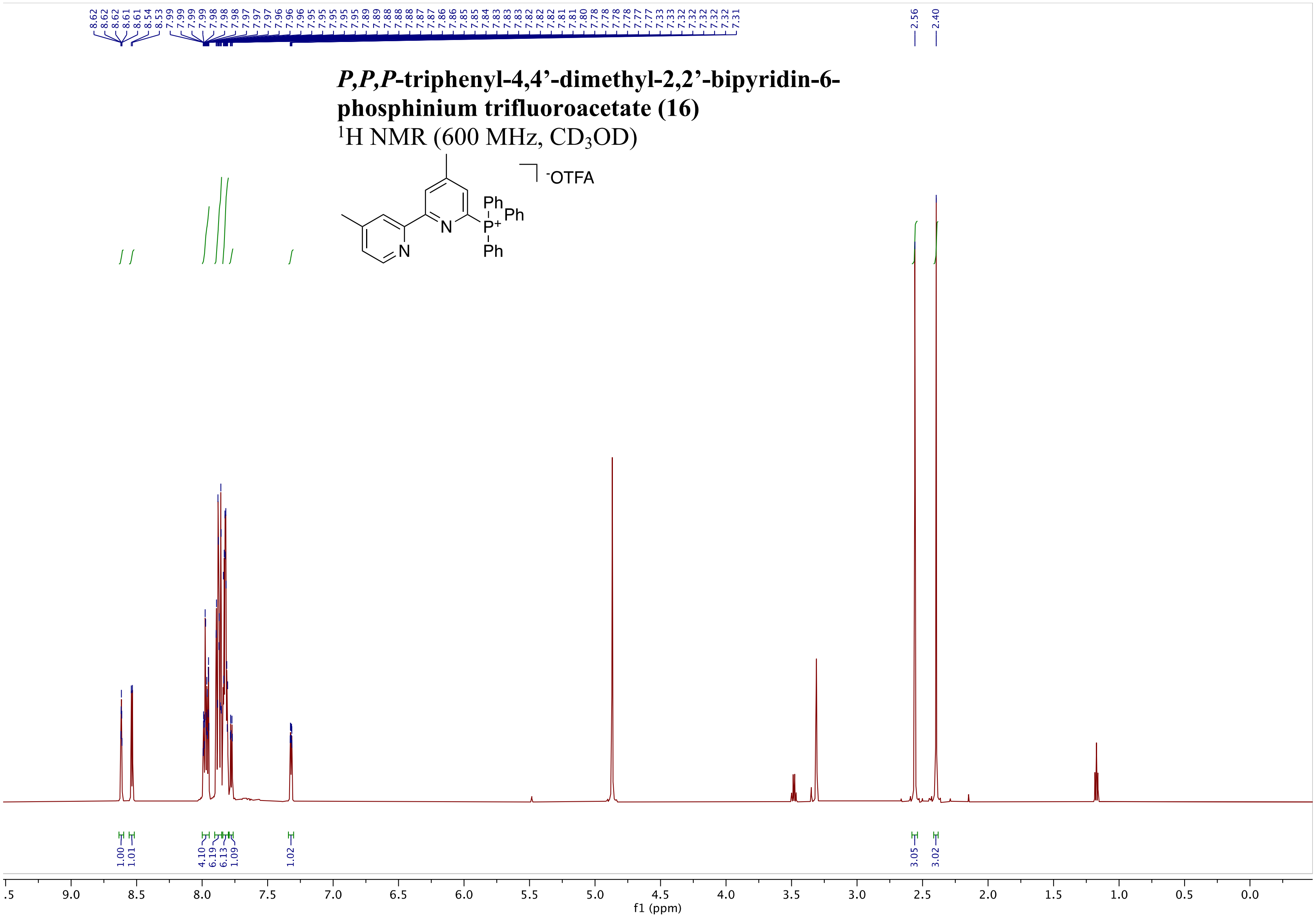

***P,P,P*-triphenyl-4,4’-dimethyl-2,2’-bipyridin-6-phosphinium trifluoroacetate (16)**

^{13}C NMR (151 MHz, CD_3OD)

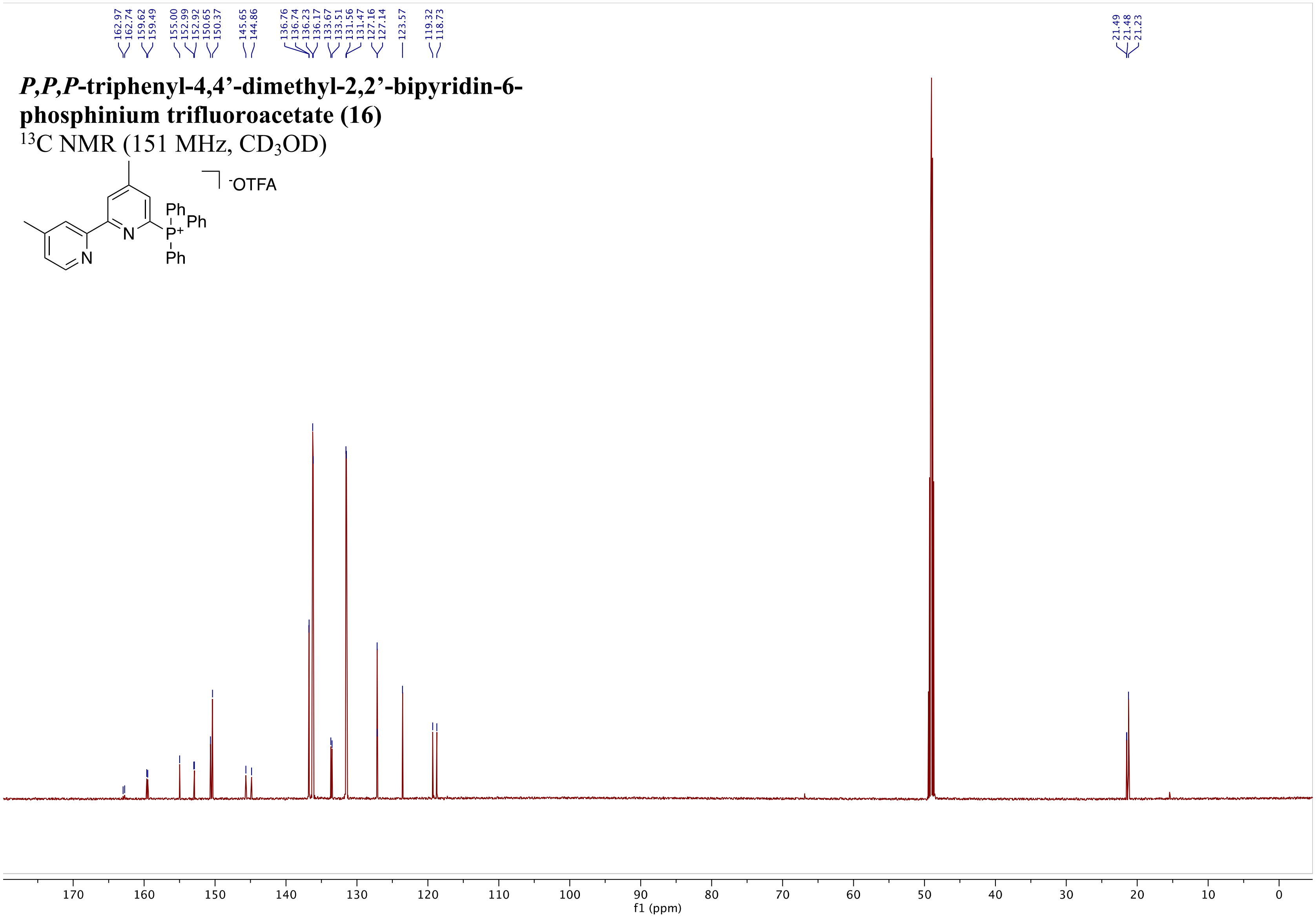

***P,P,P*-triphenyl-4,4'-dimethyl-2,2'-bipyridin-6-phosphinium trifluoroacetate (16)**

^{19}F NMR (376 MHz, CD_3OD)

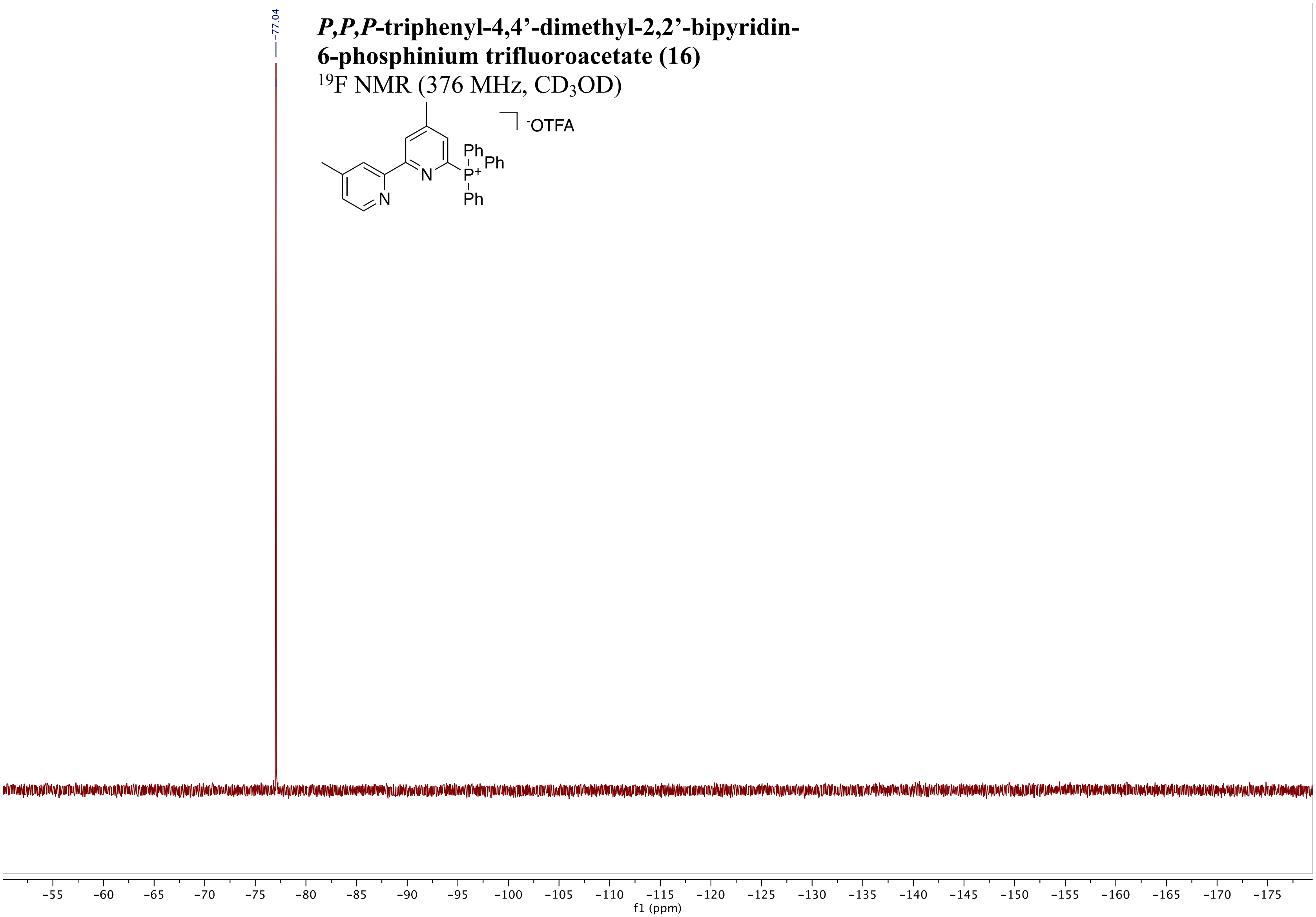

***P,P,P*-triphenyl-4,4'-dimethyl-2,2'-bipyridin-6-phosphinium trifluoroacetate (16)**

^{31}P NMR (162 MHz, CD_3OD)

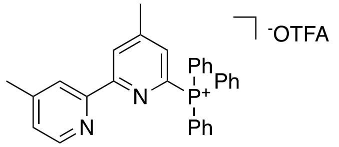

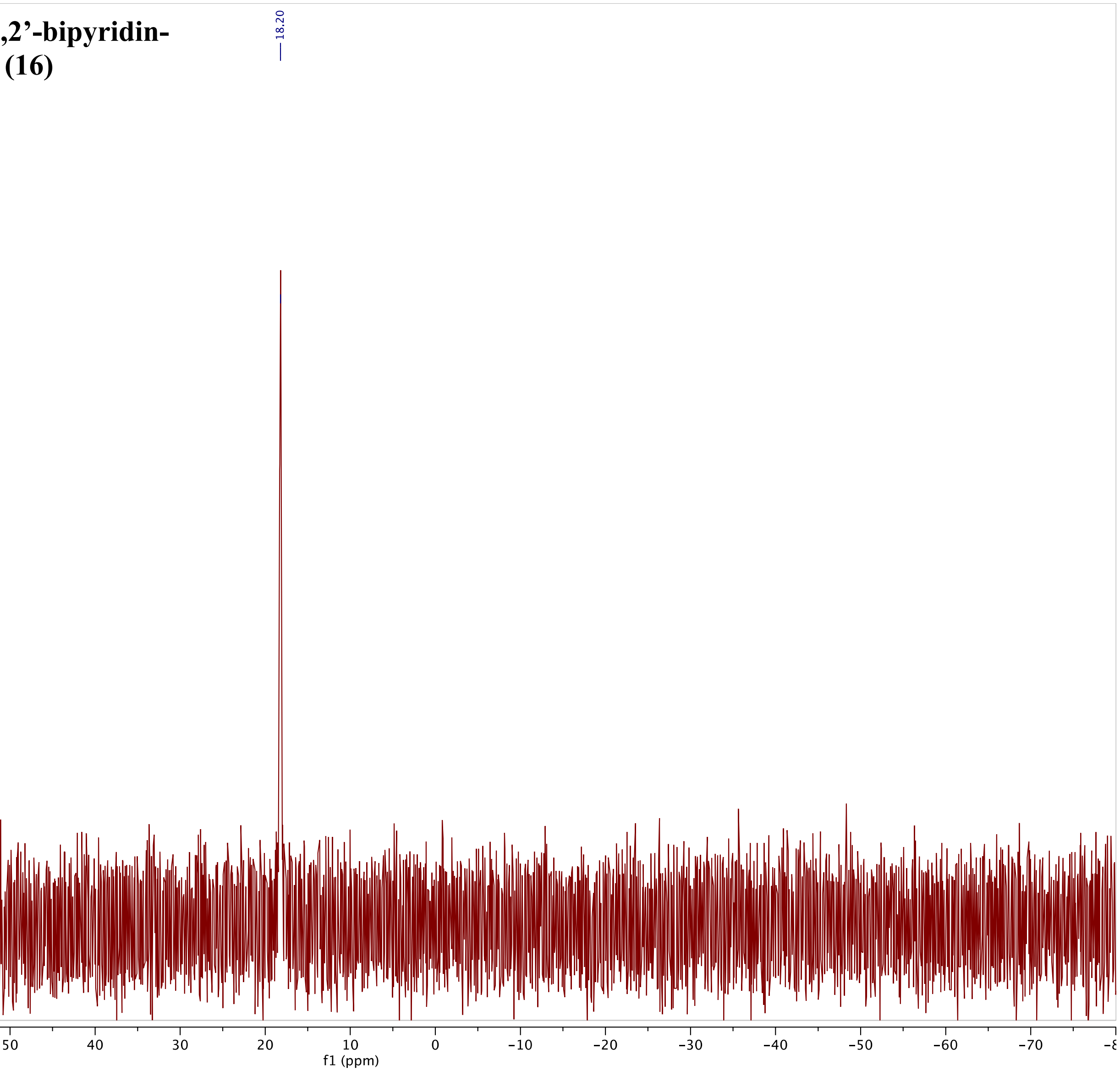

***P,P,P,P',P',P'*-hexa(*p*-tolyl)-4,4'-di-*tert*-butyl-2,2'-bipyridin-6,6'-diphosphinium bis(trifluoroacetate) (17)**

^{1}H NMR (600 MHz, CD_3OD)

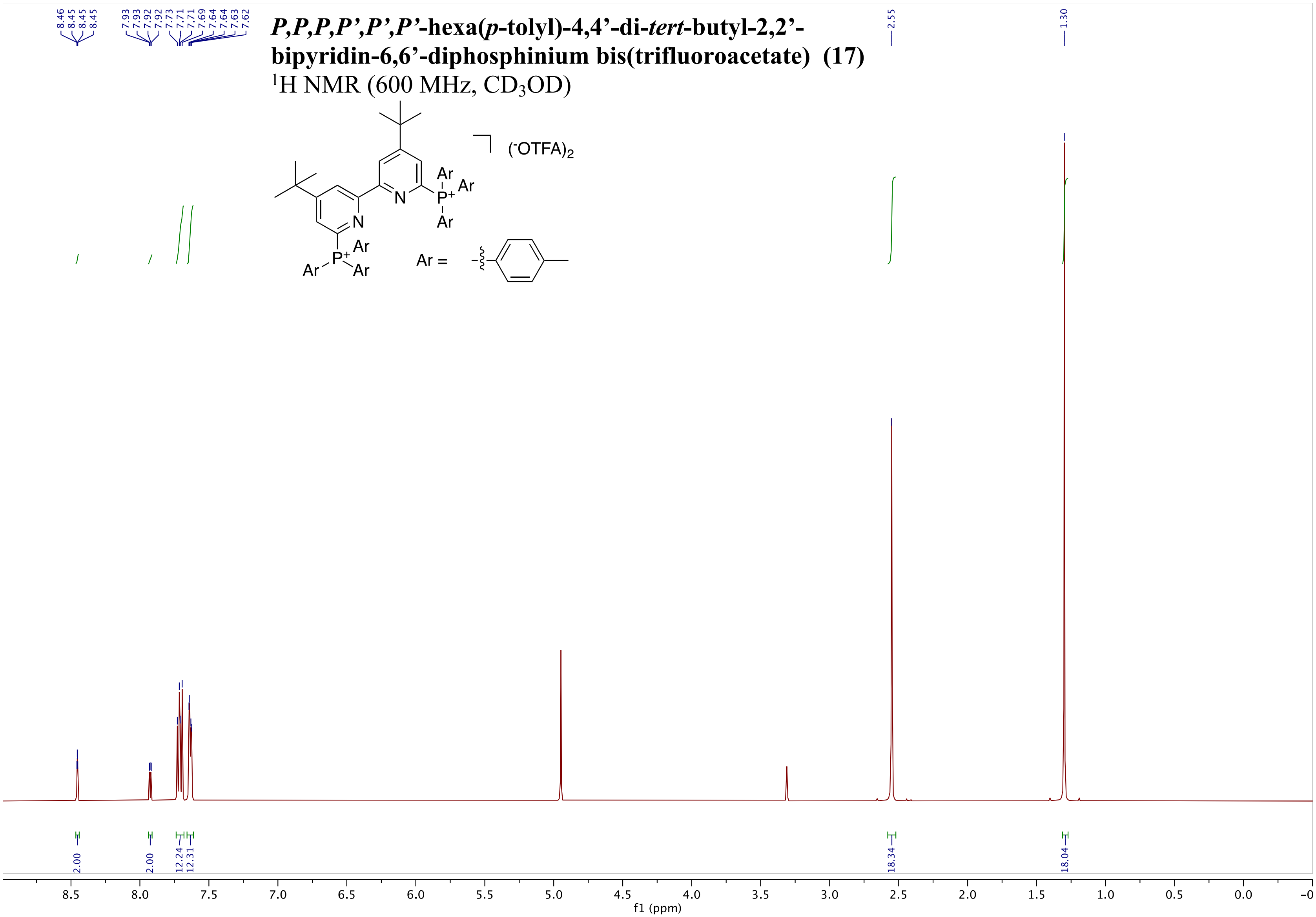

***P,P,P,P',P',P'*-hexa(*p*-tolyl)-4,4'-di-*tert*-butyl-2,2'-bipyridin-6,6'-diphosphinium bis(trifluoroacetate) (17)**

^{13}C NMR (151 MHz, CD_3OD)

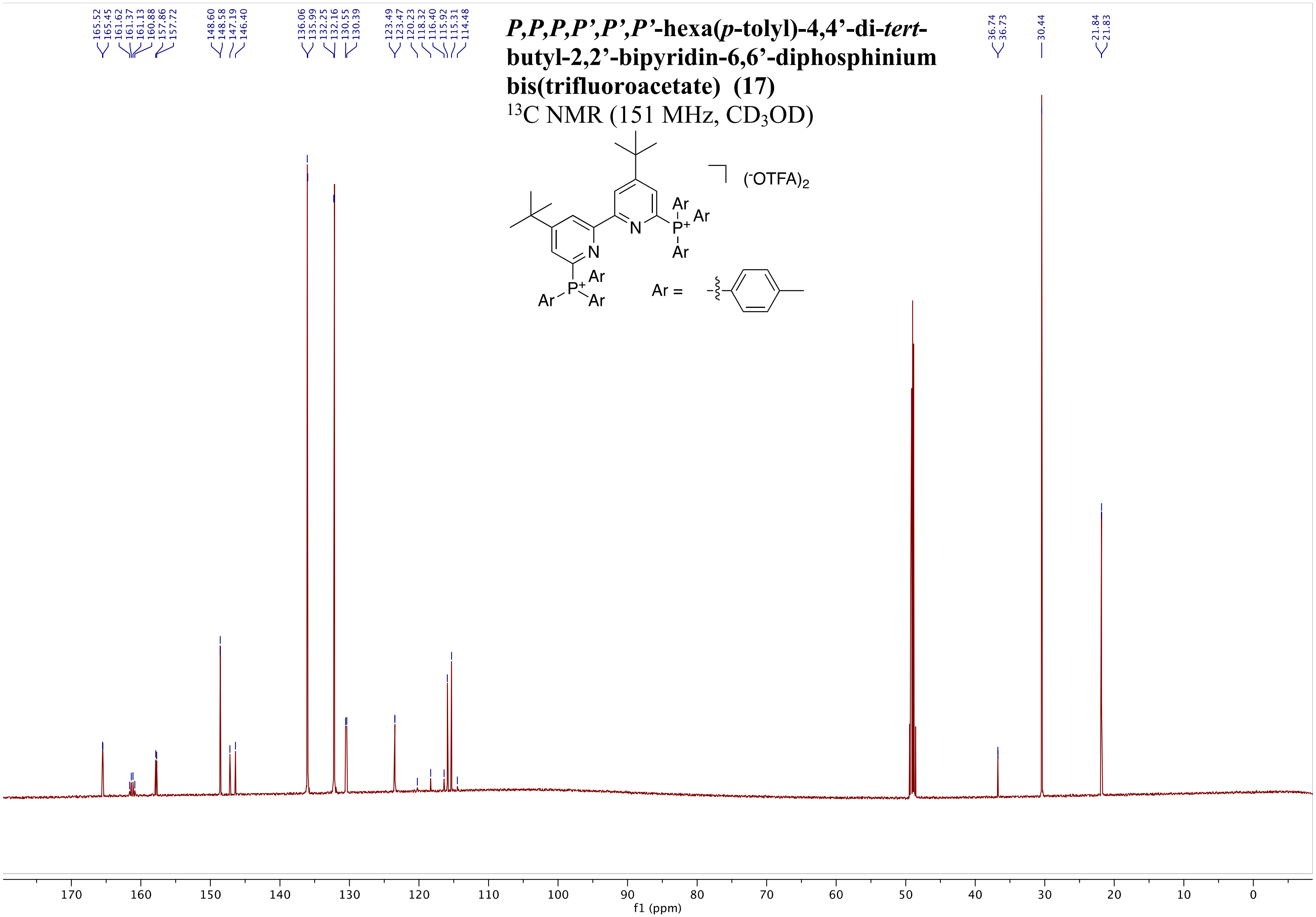

***P,P,P,P',P',P'*-hexa(*p*-tolyl)-4,4'-di-*tert*-butyl-2,2'-bipyridin-6,6'-diphosphinium bis(trifluoroacetate) (17)**

^{19}F NMR (376 MHz, CD_3OD)

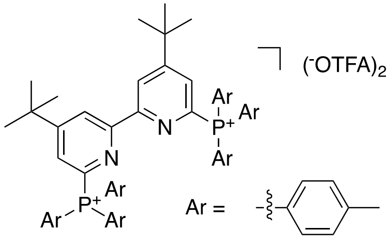

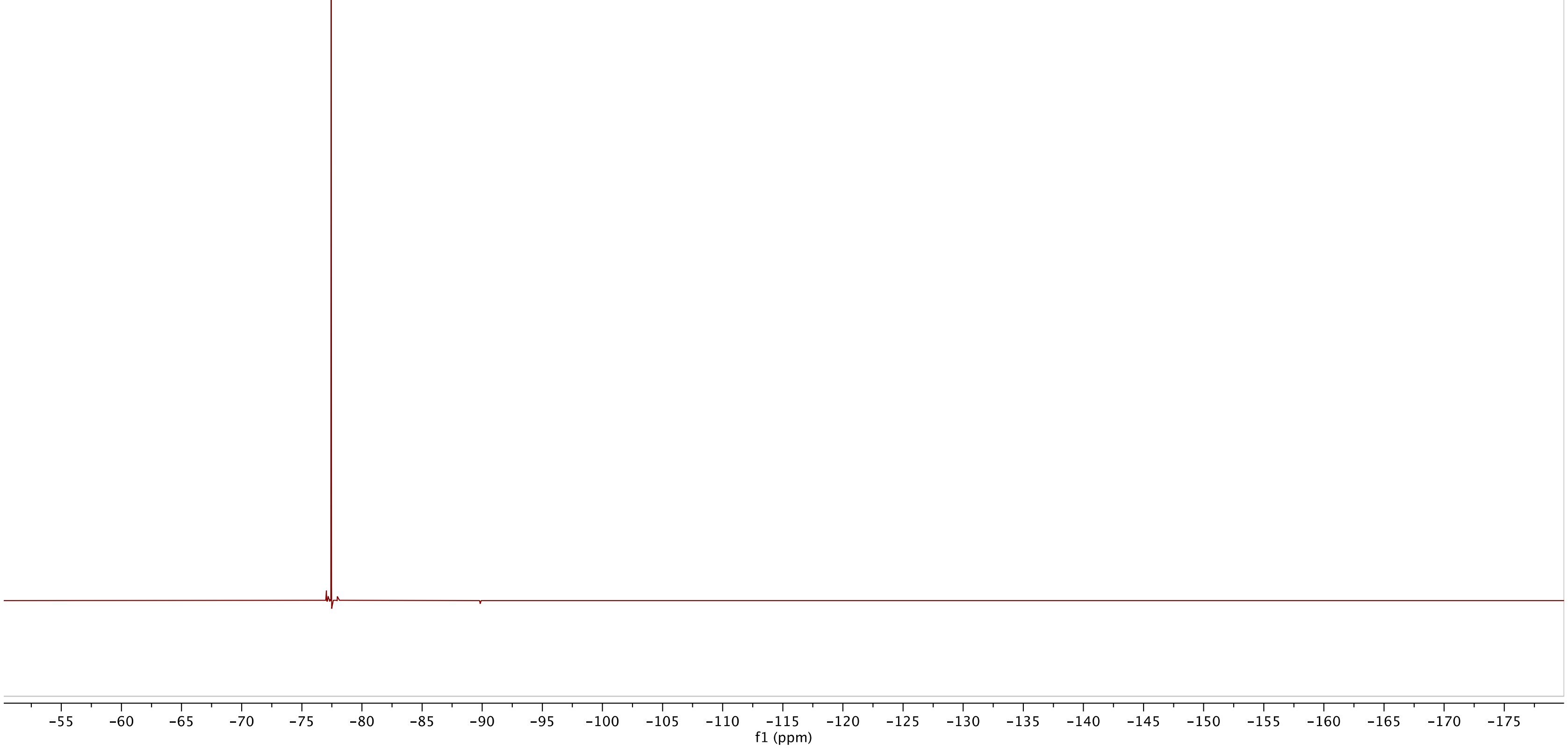

_P,P,P,P',P',P'_-hexa(_p_-tolyl)-4,4'-di-_tert_-butyl-2,2'-bipyridin-6,6'-diphosphinium bis(trifluoroacetate) (17)

^{31}P NMR (162 MHz, CD_3OD)

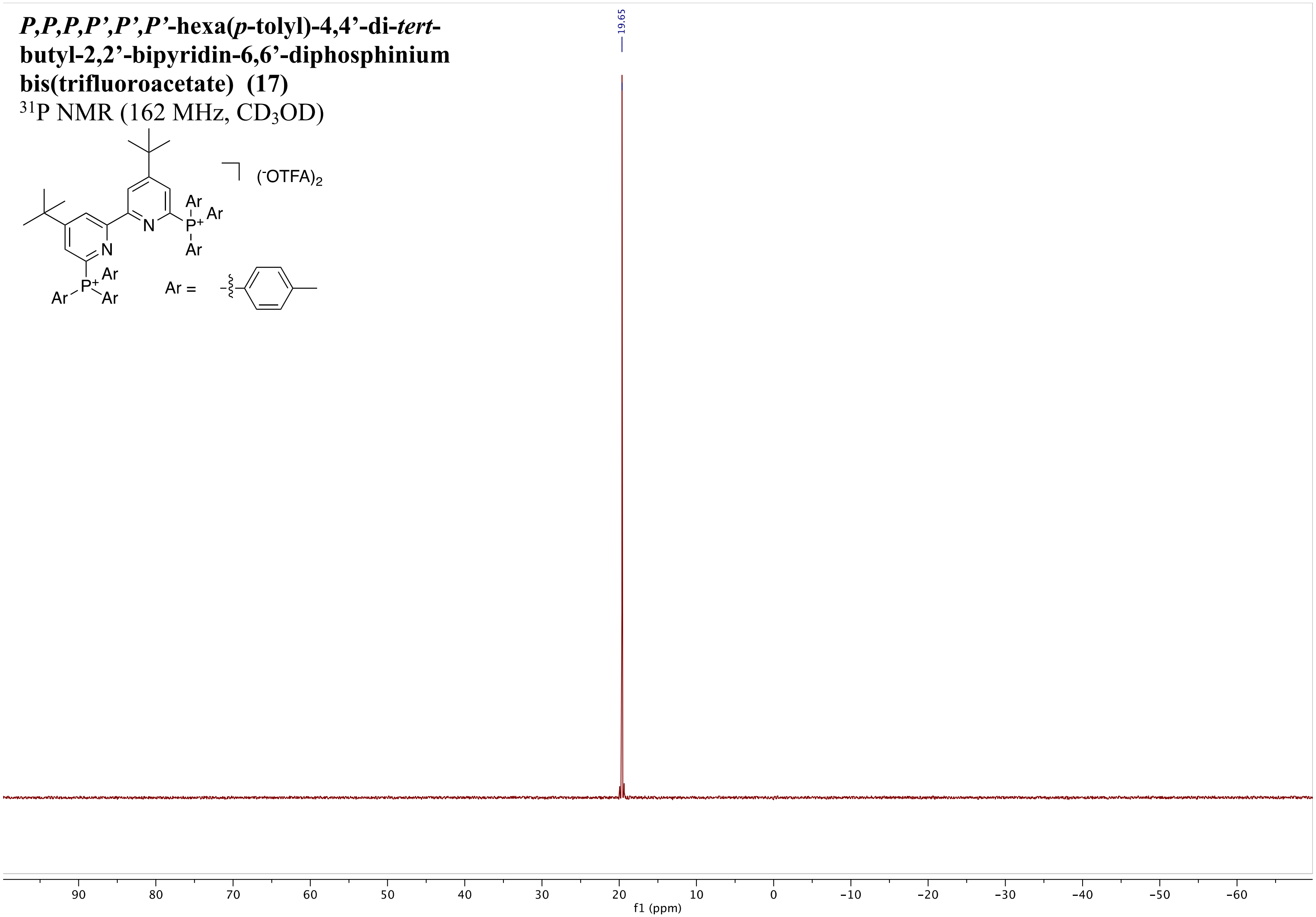

[2,2'-biquinolin]-4-yltriphenylphosphonium trifluoroacetate (18)

^{1}H NMR (600 MHz, CD_2Cl_2)

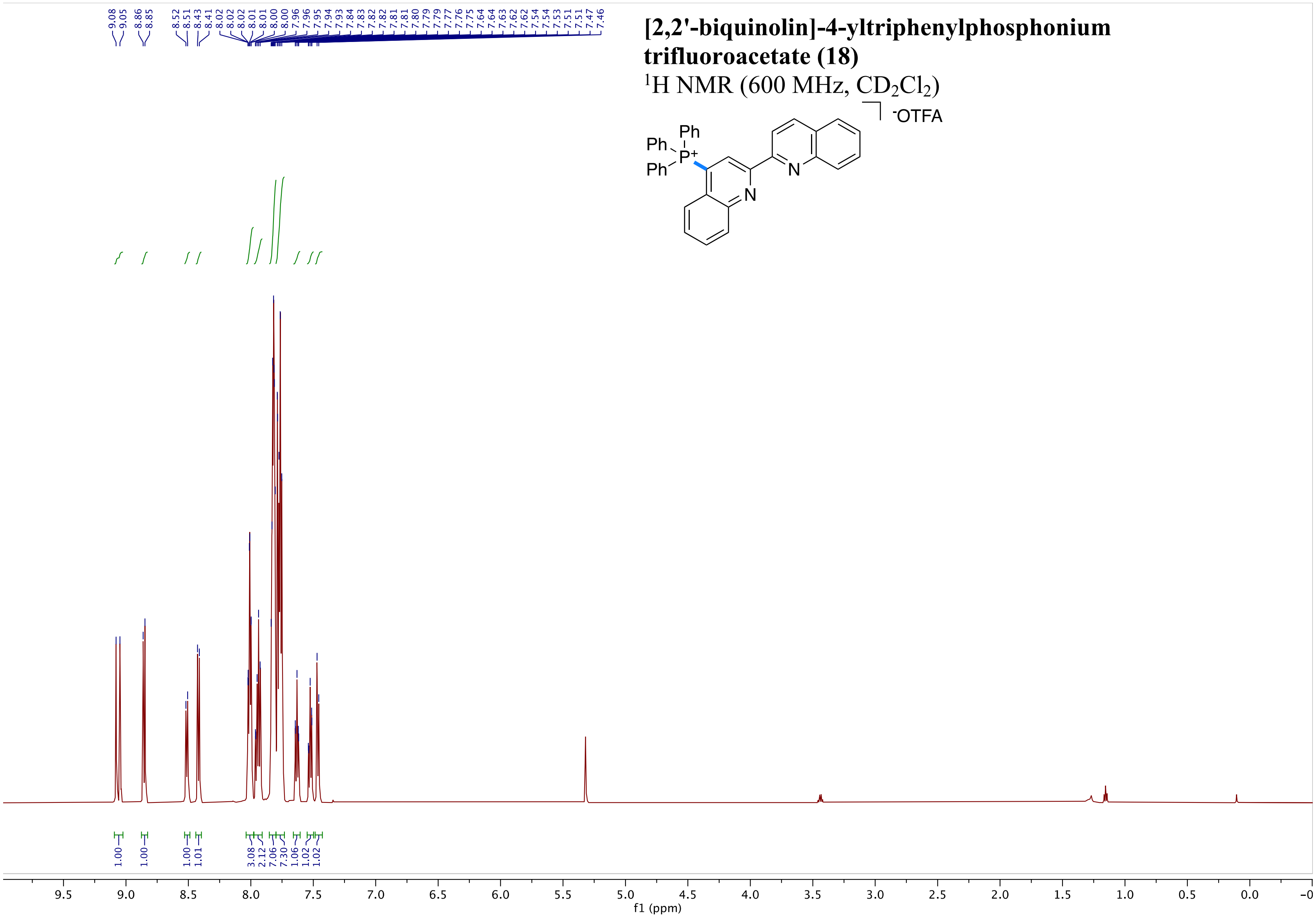

[2,2'-biquinolin]-4-yltriphenylphosphonium trifluoroacetate (18)
^{13}C NMR (151 MHz, CD_2Cl_2)

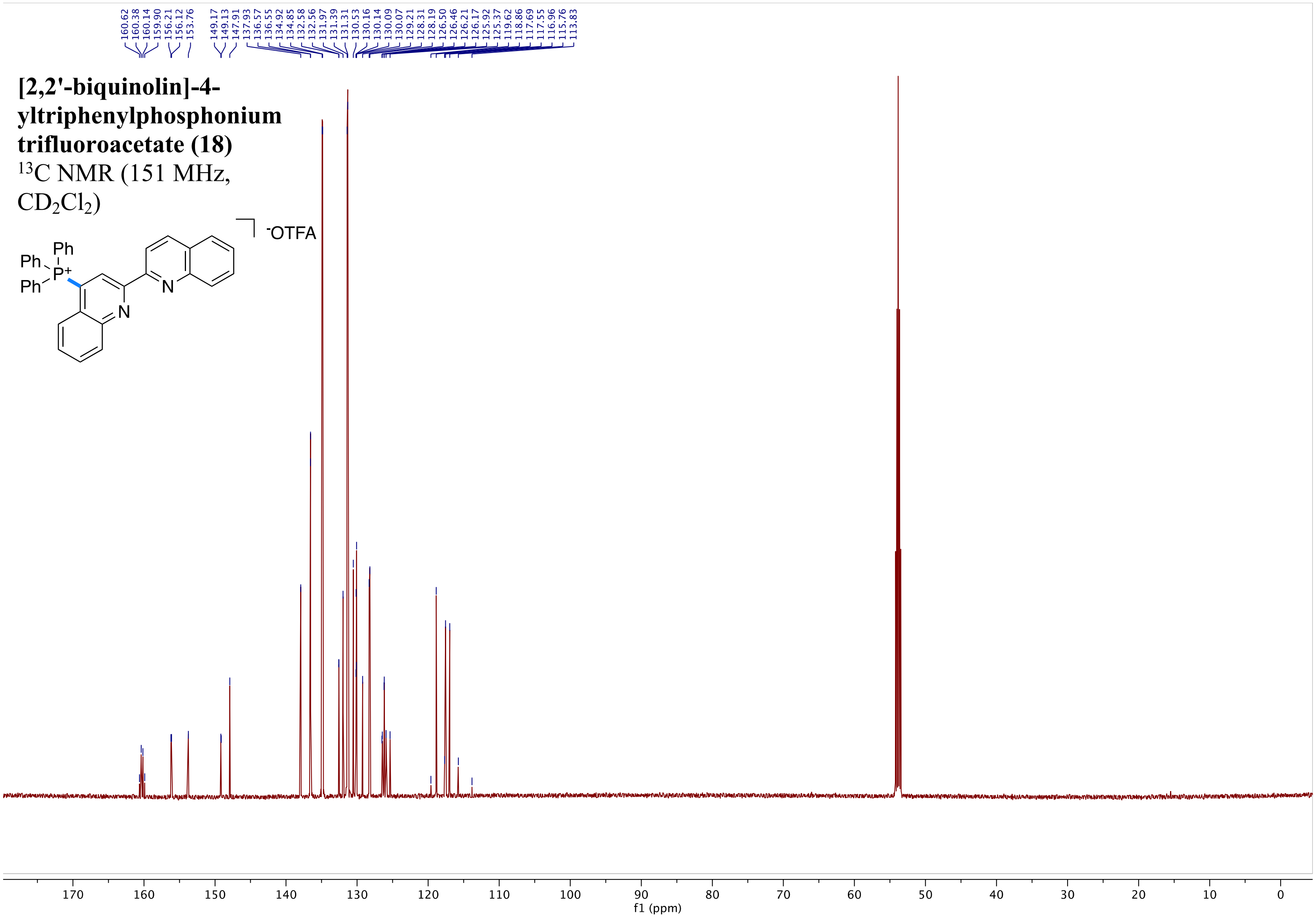

[2,2'-biquinolin]-4-yltriphenylphosphonium trifluoroacetate (18)

^{19}F NMR (376 MHz, CD_2Cl_2)

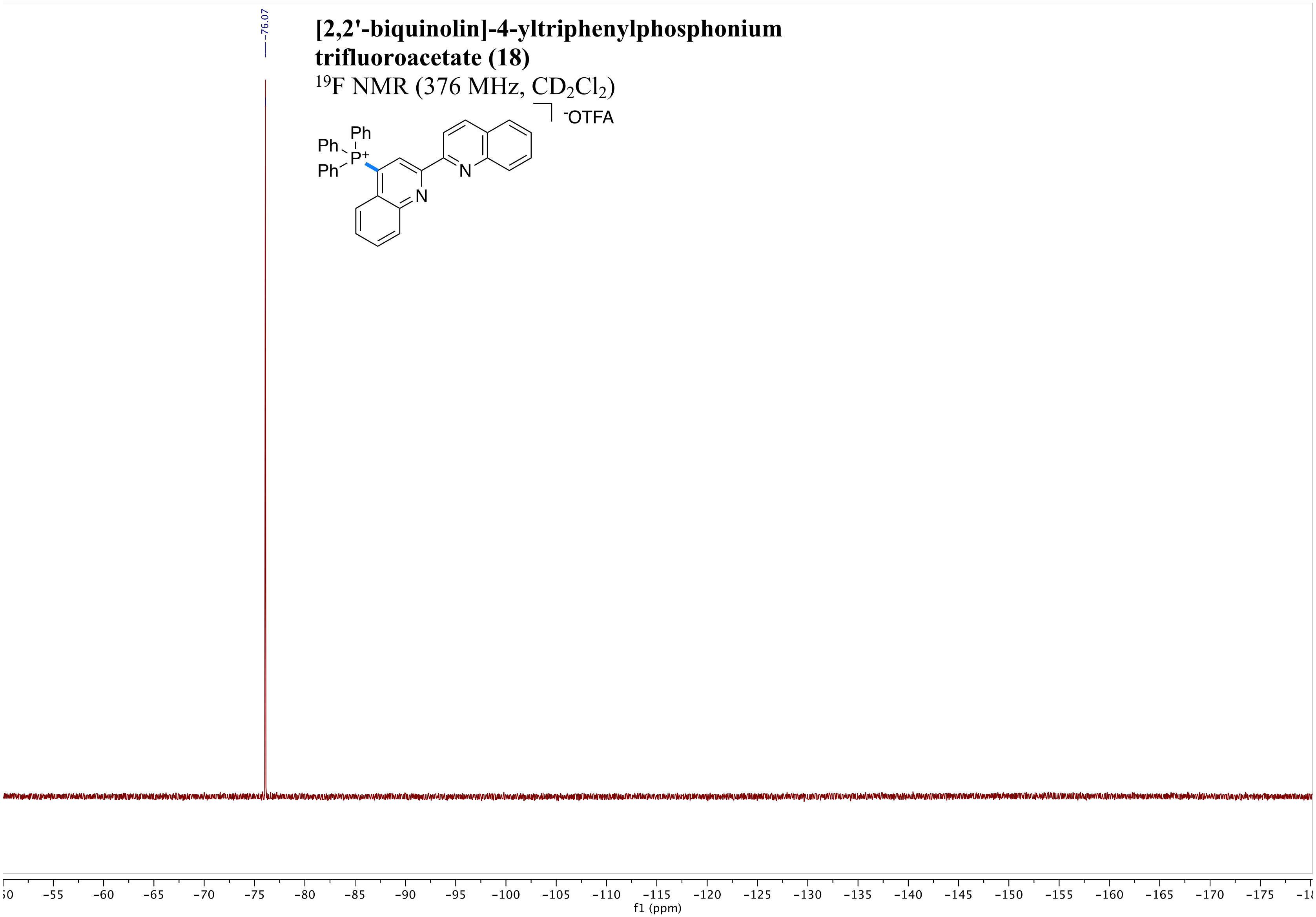

[2,2'-biquinolin]-4-yltriphenylphosphonium trifluoroacetate (18)

^{31}P NMR (162 MHz, CD_2Cl_2)

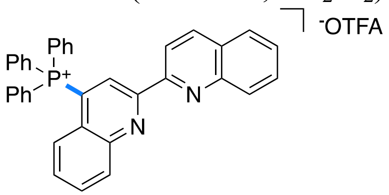

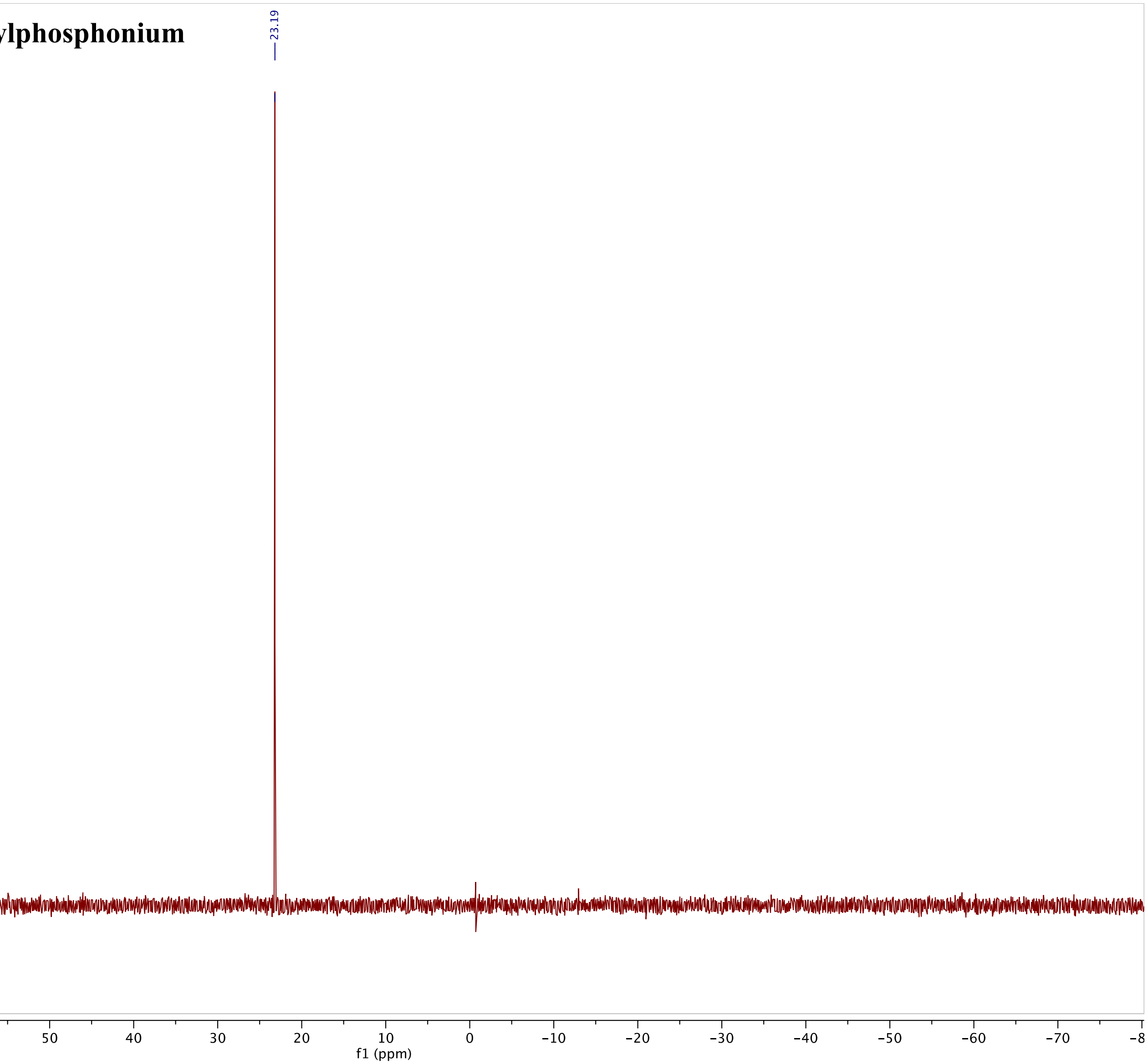

1,1'-dimethyl-[2,2'-bipyridine]-1,1'-diium bis(trifluoromethanesulfonate) (19)

^{1}H NMR (600 MHz, CD_3CN)

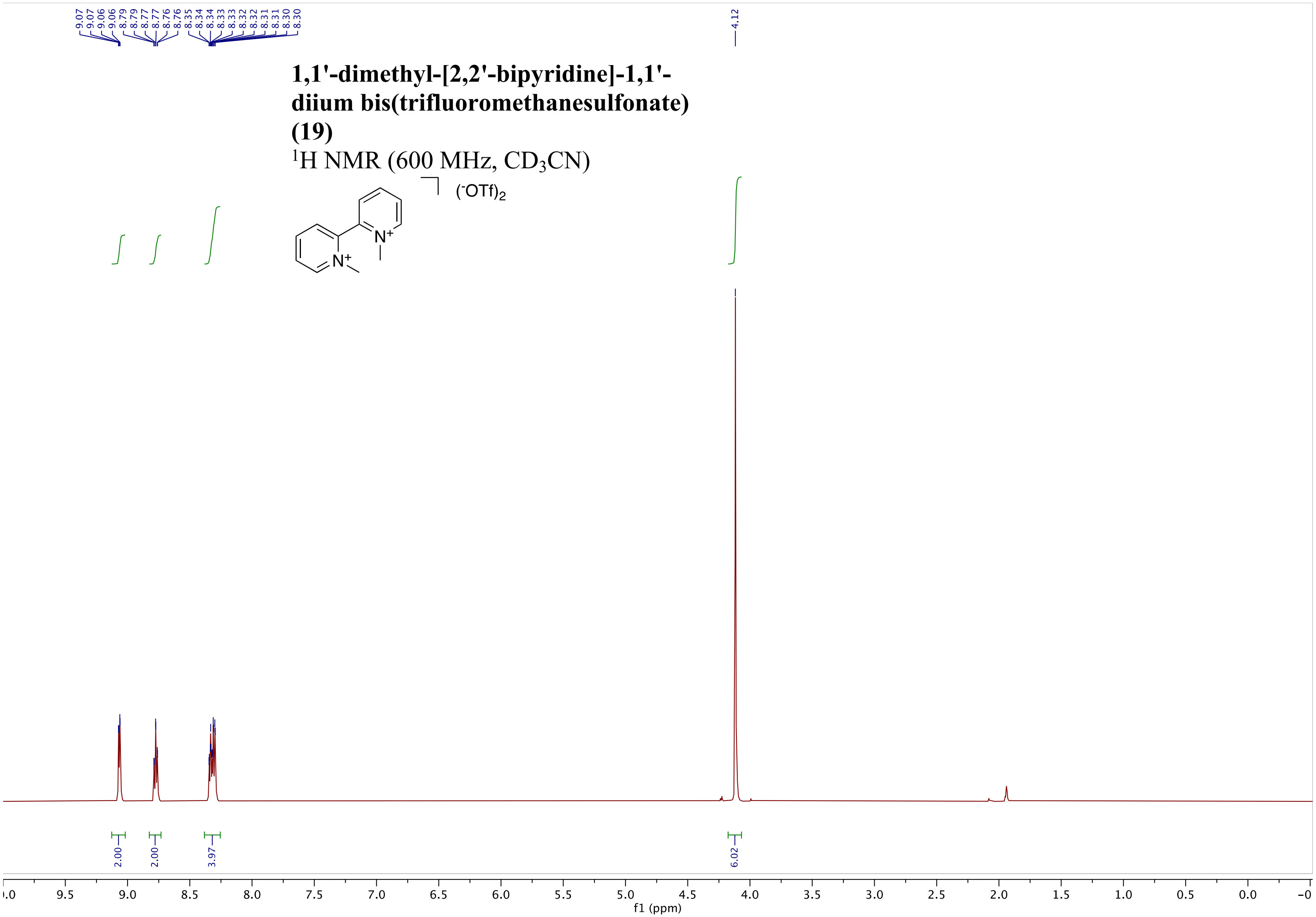

1,1'-dimethyl-[2,2'-bipyridine]-1,1'-diium bis(trifluoromethanesulfonate) (19)

^{13}C NMR (151 MHz, CD_3CN)

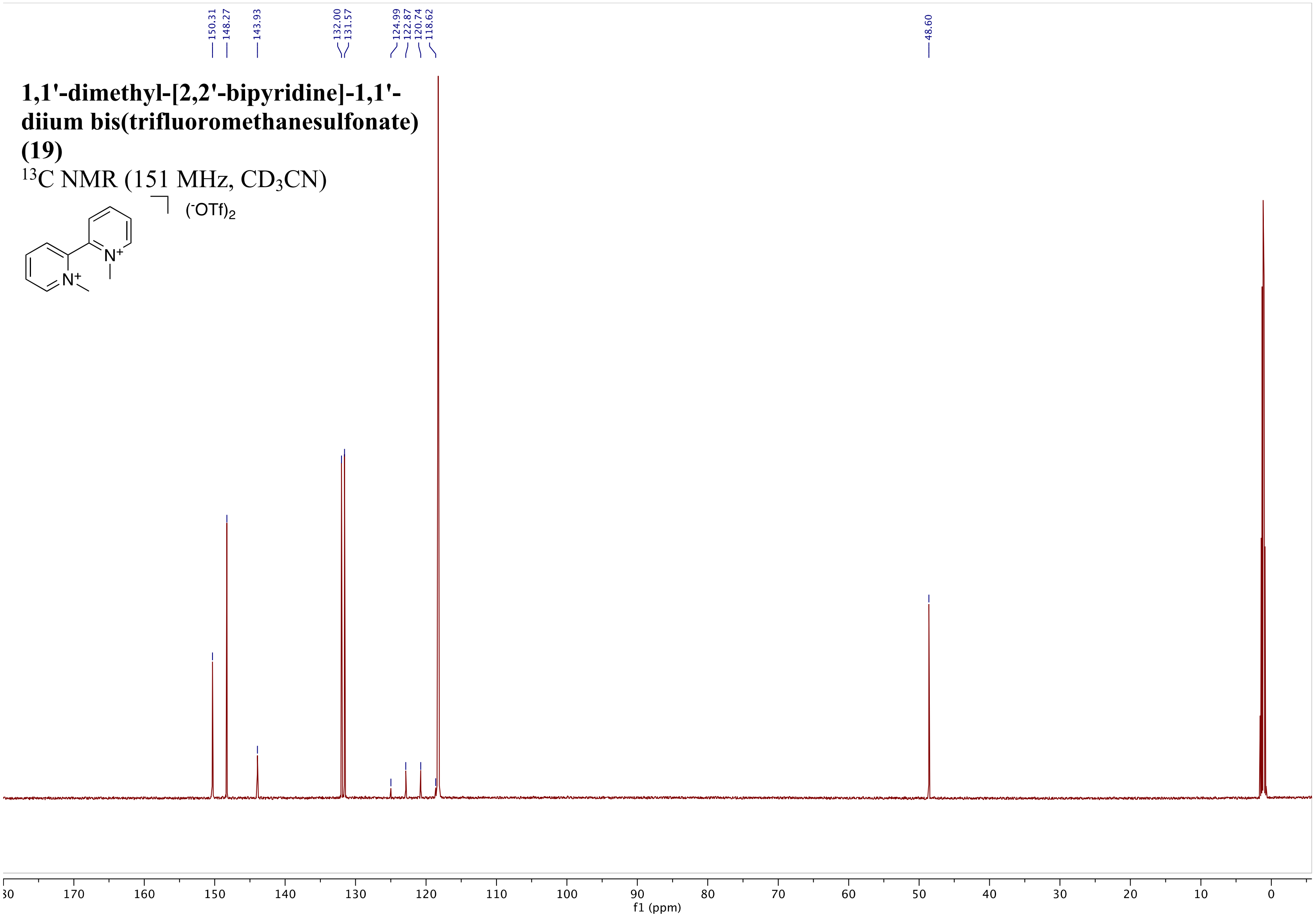

1,1'-dimethyl-[2,2'-bipyridine]-1,1'-diium bis(trifluoromethanesulfonate) (19)

^{19}F NMR (565 MHz, CD_3CN)

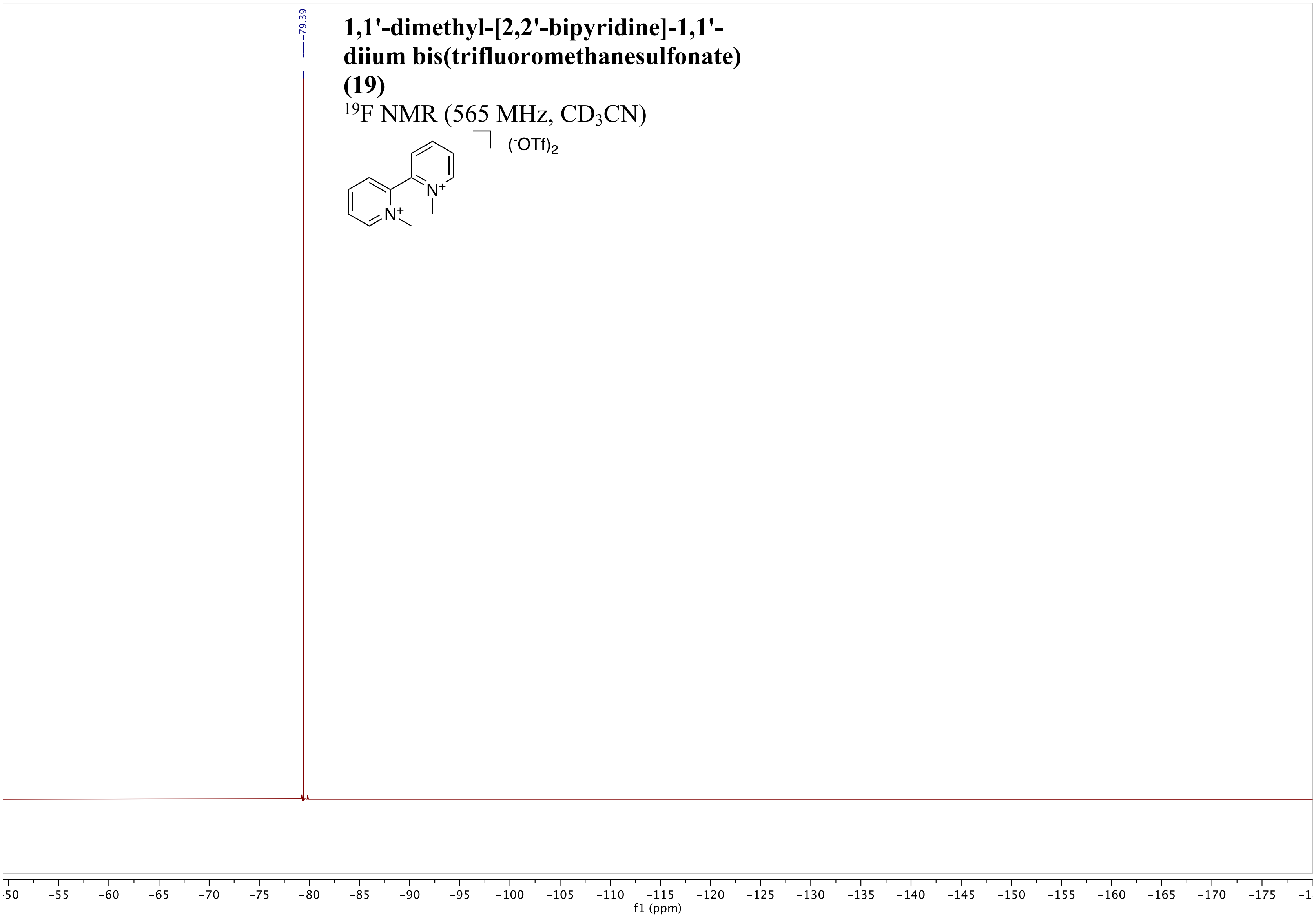

([2,2'-biquinolin]-4-yltriphenylphosphonium)chlororhenium tricarbonyl trifluoroacetate (20)

^{1}H NMR (600 MHz, CD_3CN)

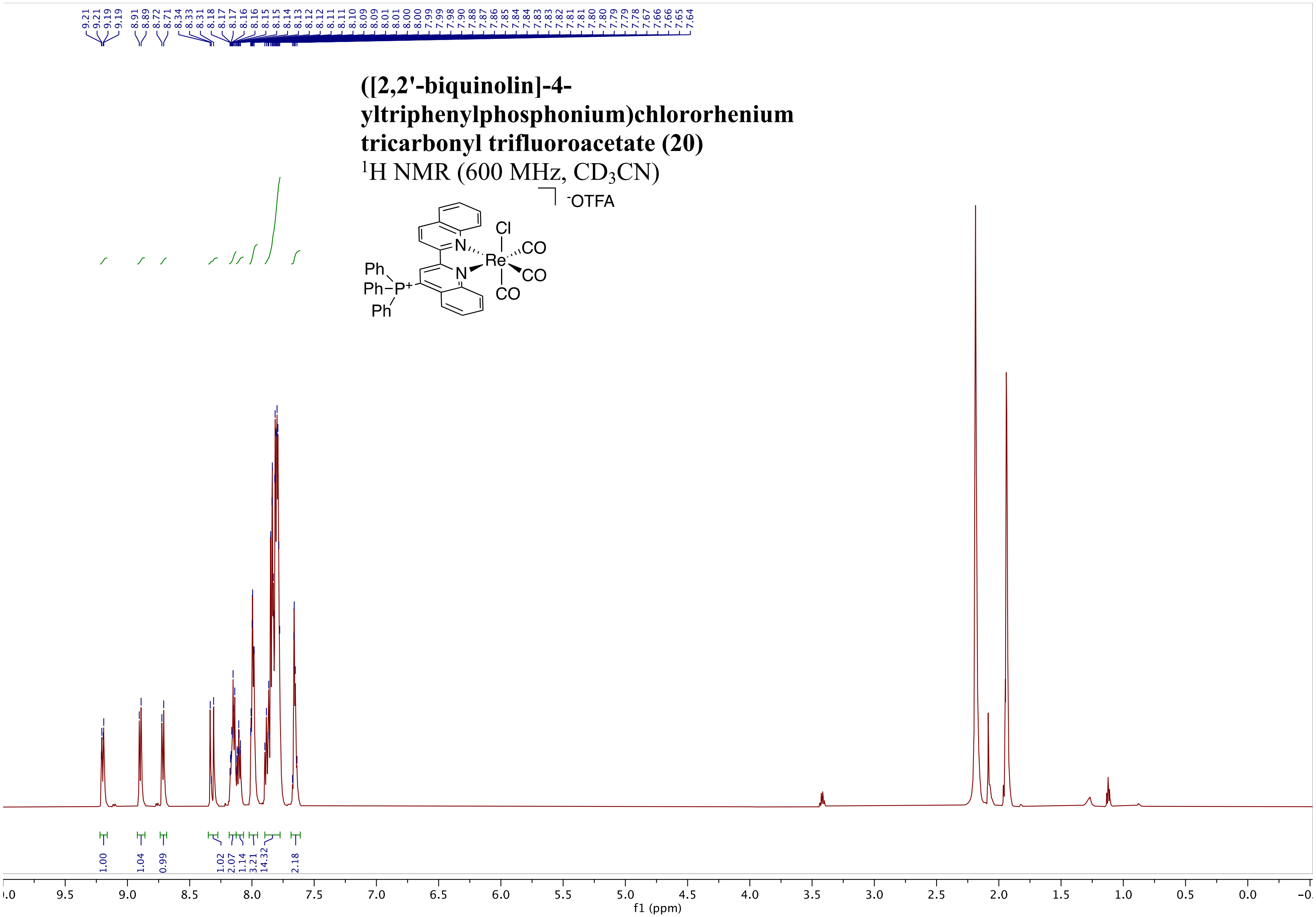

([2,2'-biquinolin]-4-yltriphenylphosphonium)chlororhenium tricarbonyl trifluoroacetate (20)

^{13}C NMR (151 MHz, CD_3CN)

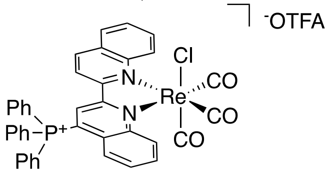

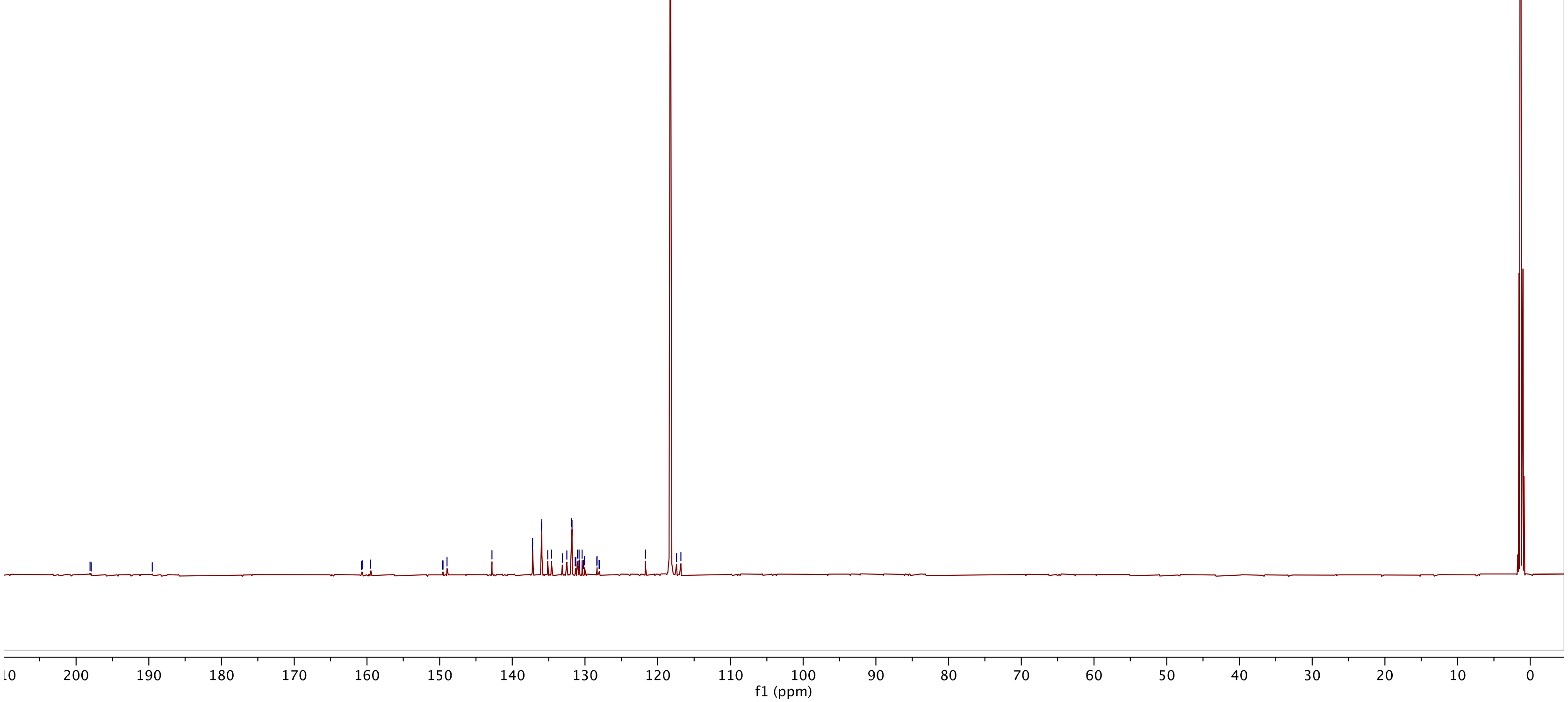

([2,2'-biquinolin]-4-yltriphenylphosphonium)chlororhenium tricarbonyl trifluoroacetate (20)

^{13}C NMR (151 MHz, CD_3CN): zoom

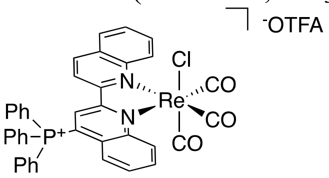

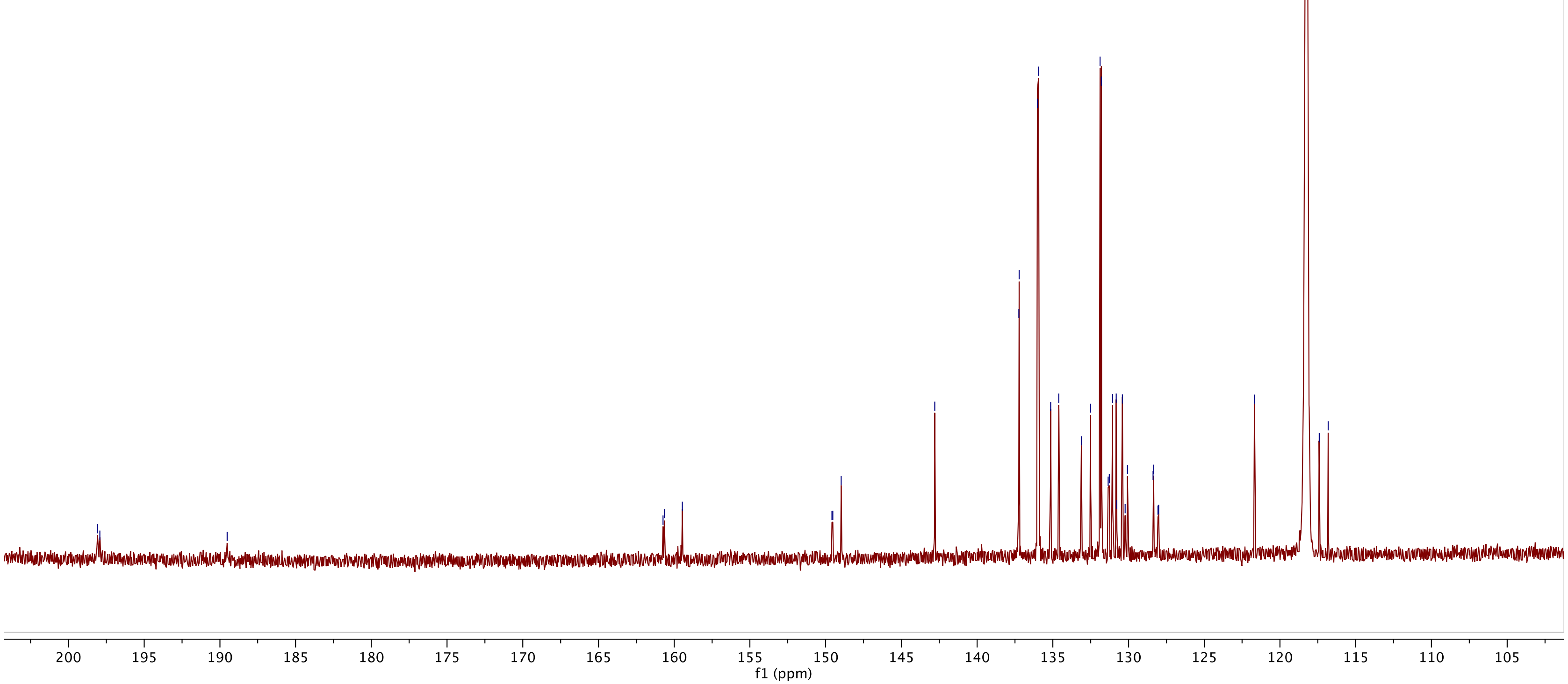

([2,2'-biquinolin]-4-yltriphenylphosphonium)chlororhenium tricarbonyl trifluoroacetate (20)

^{19}F NMR (565 MHz, CD_3CN)

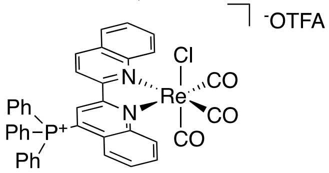

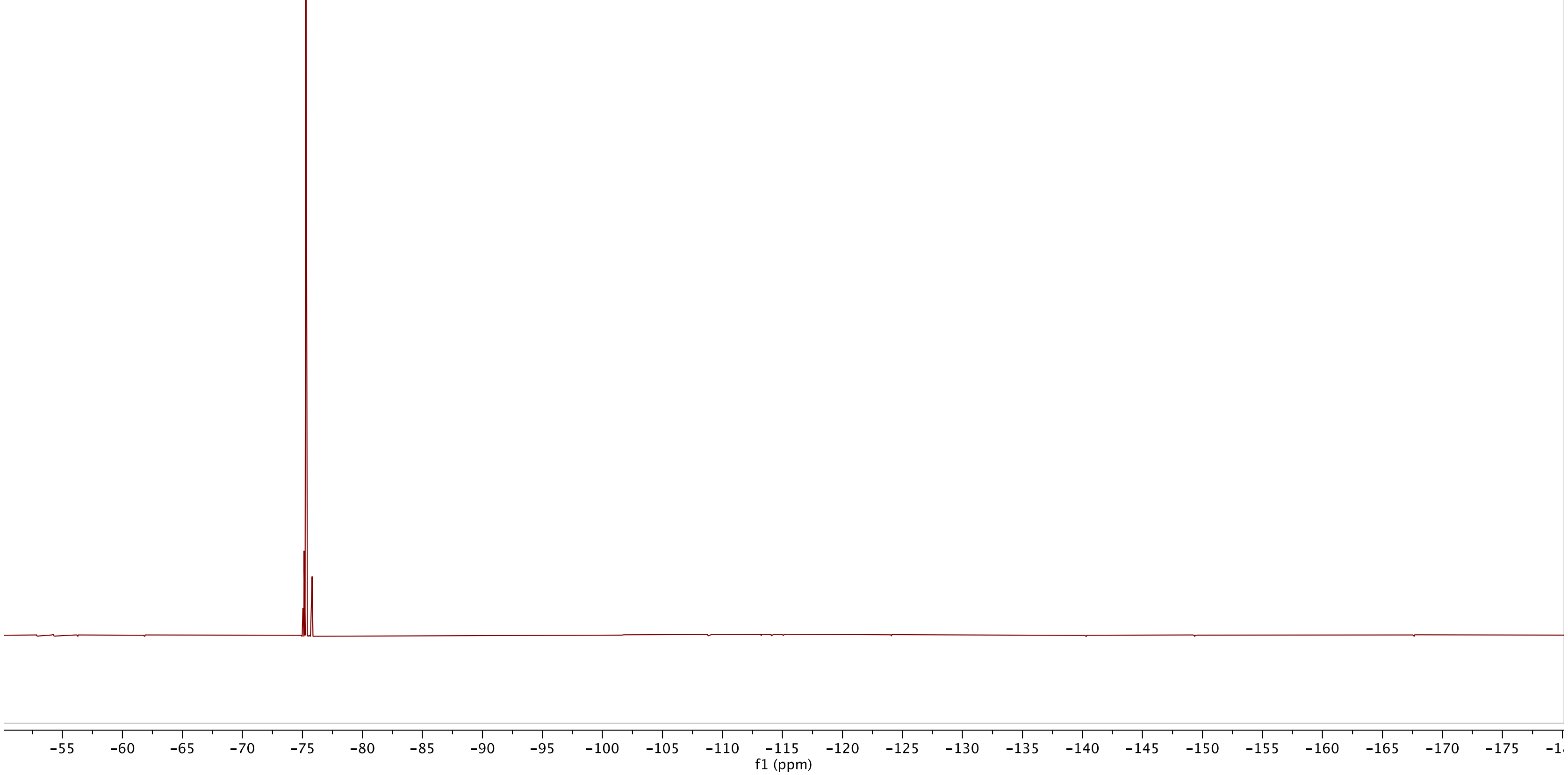

([2,2'-biquinolin]-4-yltriphenylphosphonium)chlororhenium tricarbonyl trifluoroacetate (20)
^{31}P NMR (243 MHz, CD_3CN)

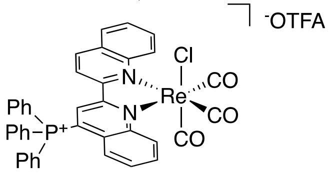

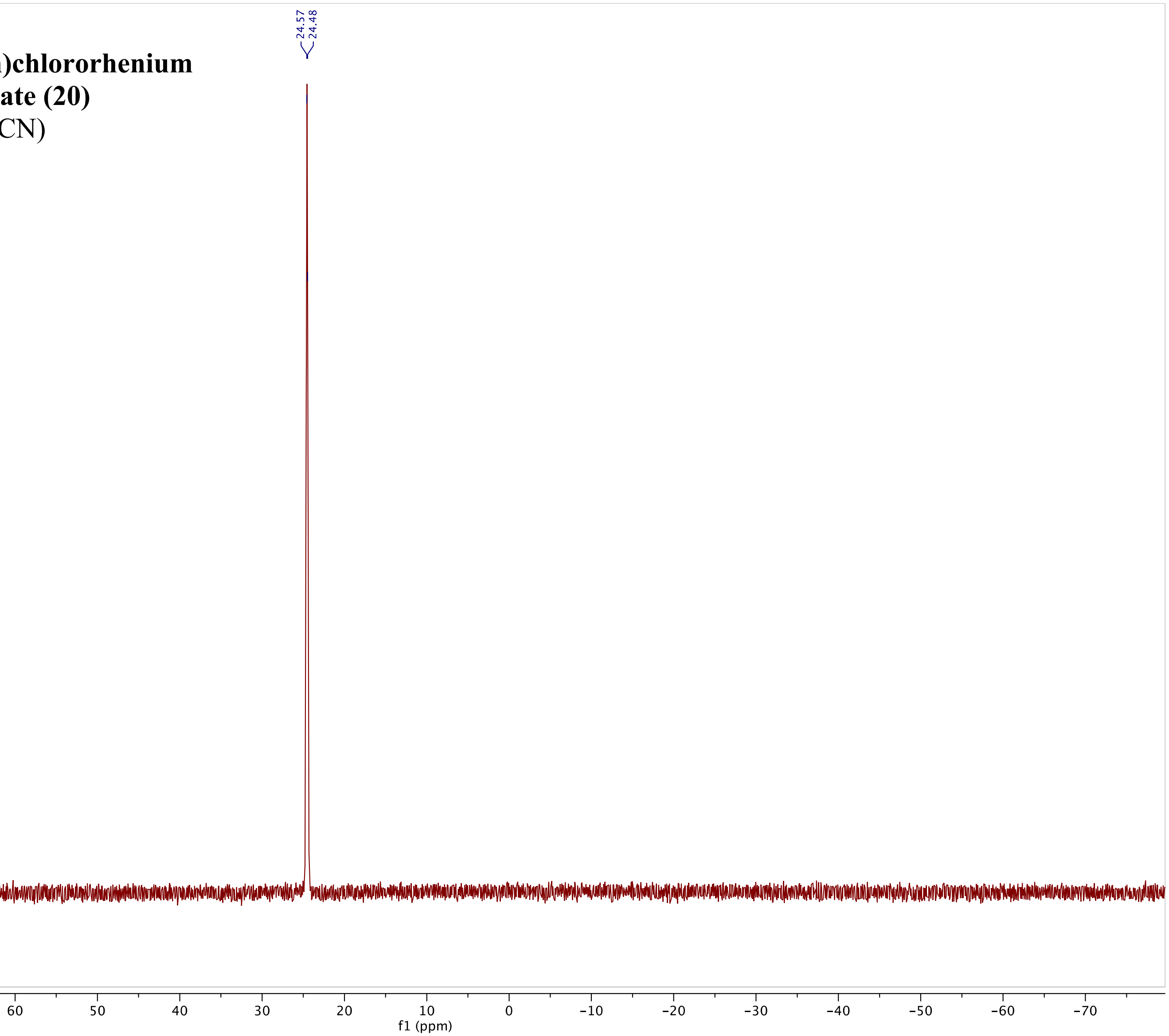

Supplement: SC-014-D3SC04864K-s001 [file SC-014-D3SC04864K-s001.pdf]
